# Combining the Ugi-azide multicomponent reaction and rhodium(III)-catalyzed annulation for the synthesis of tetrazole-isoquinolone/pyridone hybrids

**DOI:** 10.3762/bjoc.15.237

**Published:** 2019-10-16

**Authors:** Gerardo M Ojeda, Prabhat Ranjan, Pavel Fedoseev, Lisandra Amable, Upendra K Sharma, Daniel G Rivera, Erik V Van der Eycken

**Affiliations:** 1 Laboratory for Organic & Microwave-Assisted Chemistry (LOMAC), Department of Chemistry, University of Leuven (KU Leuven), Celestijnenlaan 200F, B-3001 Leuven, Belgium; 2 Center for Natural Product Research, Faculty of Chemistry, University of Havana, Zapata y G, 10400, La Habana, Cuba; 3 Peoples´ Friendship University of Russia (RUDN University) Miklukho-Maklaya Street 6, 117198 Moscow, Russia

**Keywords:** C–H activation, cyclization, isoquinolone, multicomponent reaction, tetrazole

## Abstract

An efficient sequence based on the Ugi-azide reaction and rhodium(III)-catalyzed intermolecular annulation has been established for the preparation of tetrazole-isoquinolone/pyridone hybrids. Several *N*-acylaminomethyltetrazoles were reacted with arylacetylenes to form the hybrid products in moderate to very good yields. The method relies on the capacity of the rhodium catalyst to promote C(sp^2^)–H activation in the presence of a suitable directing group. The Ugi-azide reaction provides broad molecular diversity and enables the introduction of the tetrazole moiety, which may further assist the catalytic reaction by coordinating the metal center. The scope of the isoquinolones is very wide and may be extended to the preparation of complex compounds having heterocyclic moieties such as pyridone, furan, thiophene and pyrrole, as well as the corresponding benzo-fused derivatives. The developed procedure is simple, reproducible and does not require inert conditions.

## Introduction

**Figure 1:**
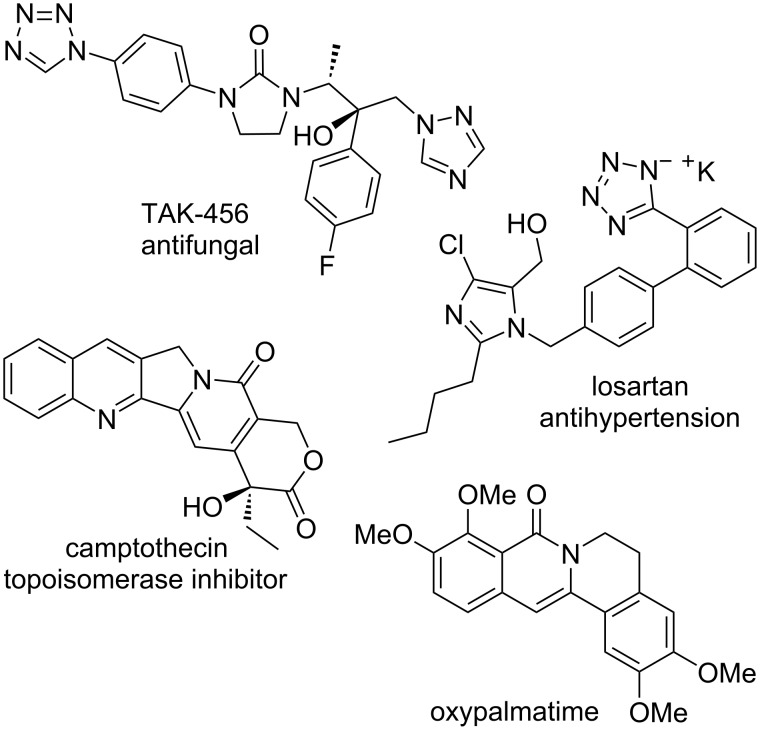
Bioactive molecules containing a tetrazole, pyridone or isoquinolone ring.

Pyridones and isoquinolones are relevant heterocyclic scaffolds present in numerous bioactive compounds and natural products [1–4]. Similarly, molecules containing a tetrazole ring exhibit a wide variety of pharmacological and antimicrobial properties [5–6], including analgesic, antihypertensive, anti-inflammatory, anticancer, antifungal and antimalarial activity (Figure 1). A key feature of the tetrazole ring is its bioisosteric character with the carboxylic acid and amide functional groups, which has been considered of interest for medicinal chemistry applications [7–8]. In recent years, the preparation of hybrid heterocyclic scaffolds including the tetrazole ring (either fused or linked to other heterocycles) has rendered potent bioactive compounds [9–13], which confirms the prospect of the tetrazole hybridization strategy in drug discovery.

**Scheme 1:**
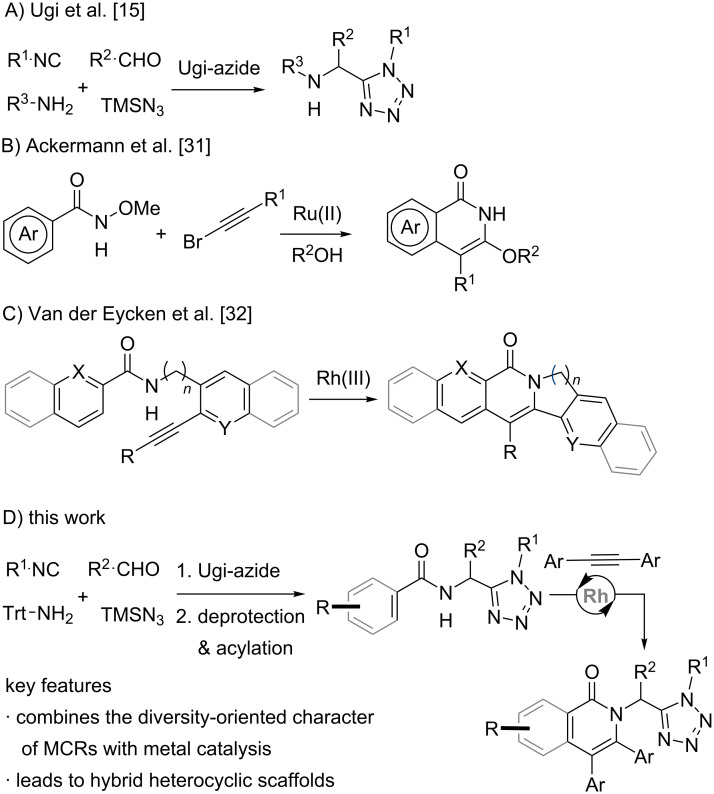
Approaches for the synthesis of tetrazoles and isoquinolones and their interplay as designed in this work.

Among the most versatile methods for obtaining tetrazoles are the Ugi-azide four-component reaction (Ugi-azide-4CR) [14–16] and the 1,3-dipolar cycloaddition of azides with (acyl)cyanides [17–18]. The Ugi-azide-4CR enables the incorporation of three different diversity-generating sites (Scheme 1A), a feature that has been exploited for the construction of libraries of tetrazole-based compounds with potential bioactivity [9–12,19]. A powerful approach for obtaining hybrid heterocyclic compounds including the tetrazole ring comprises an initial Ugi-azide-4CR followed by a cyclization step, involving some of the reactive functionalities previously installed in the multicomponent process [20–26]. However, the interplay between the multicomponent synthesis of tetrazoles and metal-catalyzed cyclization processes has remained underexploited [27]. Thus, we envisioned that the combination of an Ugi-azide-4CR with modern metal-catalyzed C–C bond-forming methods would enable access to structurally novel compounds featuring hybrid heterocycle platforms.

We focused on metal-catalyzed annulation approaches that could form isoquinolones and pyridones linked to a tetrazole ring, since such class of hybrid compounds is not described in the literature. Several protocols based on C–H activation or metal-catalyzed cyclizations are known to generate the isoquinolone and pyridone moieties and further assist their post-modifications, encompassing the use of catalysts based on complexes of Fe(III), Co(III), Ni(II), Cu(II), Ru(II), Rh(III), Ir(III), Pd(II), Ag(I) and Au(I) [28–45]. Limitations of the latter strategy, such as the poor reactivity and regioselectivity, can be overcome when a suitable directing group assists the reaction [46–47]. Traditional directing groups for C–H activation include amide, hydroxamide, carboxylate, pyridyl, quinolyl, carbonyl, ether, hydroxy, oxazolinyl and cyano [47]. For example, Ackermann et al. reported a Ru(II)-catalyzed synthesis of isoquinolones using an *N*-methoxyamide as directing group for the *ortho*-position and alkynyl bromide to achieve the regioselective cyclization (Scheme 1B) [31]. In parallel, our group has developed Ru(II) and Rh(III)-catalyzed inter- and intramolecular annulations of aromatic rings with alkynes using a secondary amide as directing group (Scheme 1C) [32,48]. In these protocols, the amide group plays a dual behavior of directing group and reaction center, as it participates in the final ring-closing reductive elimination. Herein, we report the synthesis of a new class of tetrazolo-isoquinolone/pyridone hybrids by means of a reaction sequence comprising an Ugi-azide-4CR and a Rh(III)-catalyzed annulation as key steps (Scheme 1D).

## Results and Discussion

**Scheme 2:**
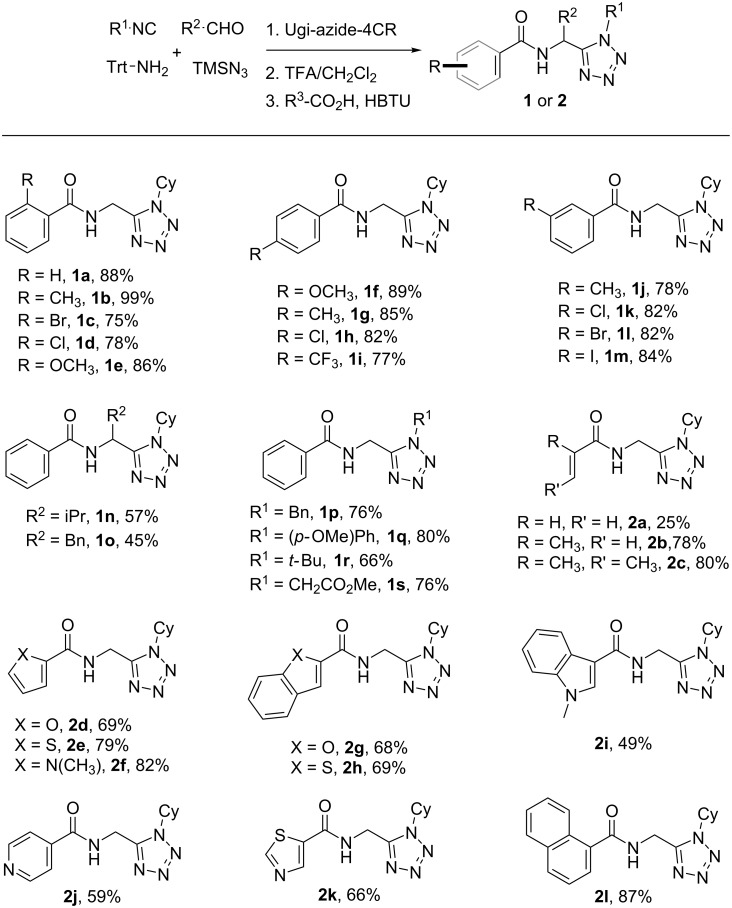
Scope of the Ugi-azide-4CR/deprotection/acylation sequence. Ugi-azide-4CR conducted at the 2.0 mmol scale with tritylamine (1.0 equiv), aldehyde (1.5 equiv), isocyanide (1.1 equiv) and TMSN_3_ (1.1 equiv) in EtOH at 100 °C (MW radiation, two cycles of 15 min each).

In previous works, our group has described a variety of synthetic approaches that combine the diversity-generating character of multicomponent reactions (MCRs) with the large synthetic scope of metal-catalyzed cyclization protocols [49–54]. In this sense, we envisioned that the application of modern Ru(II) or Rh(III)-catalyzed annulations on Ugi-azide-4CR-derived scaffolds could be a promising strategy to generate novel hybrid compounds. As shown in Scheme 2, the design of the tetrazolic substrate included the incorporation of an *N*-acylaminomethyl moiety enabling the further metal-catalyzed transformations mediated by a C–H activation process. The method comprises the initial Ugi-azide-4CR – in which several isocyanides and aldehydes were reacted in parallel with trimethylsilyl azide and tritylamine under microwave irradiation – followed by removal of the trityl group and acylation to afford the *N*-acylaminomethyltetrazoles **1a**–**s** and **2a**–**l**. The functionalized tetrazoles were obtained in moderate to excellent yields over three steps without purification of the intermediates. In this strategy, three different diversity sites could be generated, i.e., those derived from the isocyano and aldehyde components and a third one from the carboxylic acid used in the last acylation step. We sought to incorporate aryl or vinyl carboxylic acids to allow the subsequent reaction with alkynes based on the C(sp^2^)–H activation of these aryl or vinyl moieties.

As depicted in Table 1, we chose the tetrazolic substrate **1a** for the optimization of the catalytic addition of diphenylacetylene (**3a**), a process involving the metal-catalyzed *ortho*-C–H activation of the *N*-benzamidomethyltetrazole core followed by isoquinolone-ring formation to furnish **4a**.

**Table 1:** Optimization of the reaction conditions with model compound **1a**.^a^

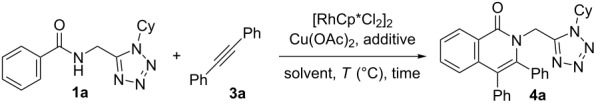					

Entry	Solvent	Additive	*T* (°C)	Time (h)	Yield (%)^b^

1	MeOH	CsOAc	120	12	59
2	THF	CsOAc	120	12	78
3	DMF	CsOAc	120	12	80
**4**	***t-*AmOH**	**CsOAc**	**120**	**12**	**90^c^**
5	*t-*AmOH	NaOAc	120	12	73
6	*t-*AmOH	–	120	12	14
7^d^	*t-*AmOH	CsOAc	120	12	48
8	*t-*AmOH	CsOAc	90	12	87
9	*t-*AmOH	CsOAc	130	12	70
10	*t-*AmOH	CsOAc	120	6	87
11	*t-*AmOH	CsOAc	120	24	80
12^e^	*t-*AmOH	CsOAc	120	12	84
13^f^	*t-*AmOH	CsOAc	120	12	90

^a^Unless otherwise stated, all reactions were carried out with **1a** (0.25 mmol, 1.0 equiv), **3a** (1.5 equiv), catalyst (5 mol %), oxidant (2.0 equiv), additive (0.5 equiv), in 1.5 mL of solvent (0.17 M) at the indicated temperature and reaction time. ^b^Yields were determined by quantitative ^1^H NMR using 3,4,6-trimethoxybenzaldehyde as internal standard. ^c^Isolated yield of 88% after column chromatography. ^d^No oxidant was added. ^e^Three equiv of alkyne were used. ^f^ One mol % of catalyst was used. Cy: cyclohexyl; Cp*: 1,2,3,4,5-pentamethylcyclopentadienyl.

We endeavored two catalytic systems based on ruthenium and rhodium, which in our laboratory have proven success in this type of cyclization [32,48]. First, the alkynylation protocol was attempted using the relatively cheap complex (*p*-cymene)ruthenium(II) chloride dimer, in the presence of copper(II) acetate as oxidant under conventional heating. Despite all effort put in this attempt, the isolated yields were in the range of 14–62%, with the highest yield achieved after 24 h of reaction using 10 mol % of catalyst (results not shown, see Supporting Information File 1). We next turned to rhodium catalysis, since despite the fact that rhodium-based catalysts are more expensive, they have proven to be very efficient using lower catalyst loadings compared to ruthenium [30,32,39,55]. Fortunately, the use of pentamethylcyclopentadienylrhodium(III) chloride dimer, [Cp*RhCl_2_]_2_, allowed reducing both the loading to 5 mol % and the reaction time to 12 h (Table 1). The addition of cesium acetate appeared to be required, probably to convert the pre-catalyst into the active catalyst. After some additional optimization varying the solvent (Table 1, entries 1–4), the isolated yield of **4a** was increased to 90% (Table 1, entry 4) with the use of *tert*-amyl alcohol (*t*-AmOH). Other experiments (Table 1, entries 5–7) proved the importance of both cesium acetate and copper(II) acetate for the reaction to proceed efficiently. The use of the nonhygroscopic and cheaper sodium acetate decreased the yield (Table 1, entry 5), whereas the absence of the acetate salt additive led to a very low reaction yield (Table 1, entry 6). Neither removal of the oxidant (Table 1, entry 7) nor further modification of the reaction conditions, i.e., time, temperature, equivalents, concentration, was beneficial for increasing the yield. Interestingly, the catalyst loading could be reduced to 1 mol % without affecting the reaction efficiency (Table 1, entry 13), although this result could not be reproduced for all the substrates employed in this work. Conventional heating at 120 °C was used, while no inert atmosphere was required.

**Scheme 3:**
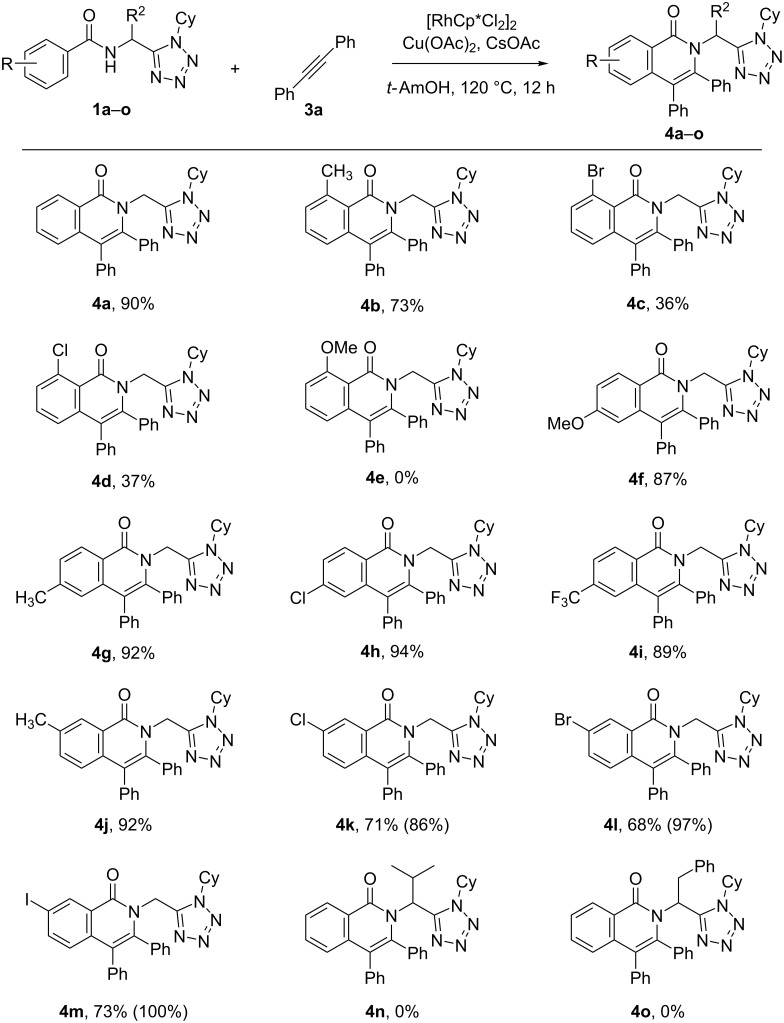
Influence of substituents R and R^2^ on the reaction outcome. For compounds **4k**–**m** the overall yield in parentheses refers to the mixture of regioisomers.

Initially, we sought to assess the scope of the *N*-benzamidomethyltetrazole bearing different substituents at the R and R^2^ position (see Scheme 1) in the Rh(III)-catalyzed reaction (Scheme 3). Thus, compounds **1a–o** were reacted with diphenylacetylene under the optimized catalytic conditions to furnish – eventually – the corresponding tetrazole/isoquinolone hybrids **4a–o**. We first fixed the R^2^ substituent as H (derived from paraformaldehyde as aldehyde component in the Ugi-azide-4CR, Scheme 1) to better evaluate the effect of the substituent in the phenyl ring. Interestingly, the presence of substituents in the *ortho-*position of the amide group (acting as a directing group) decreased the yields, with substrates bearing substituents Me, Br and Cl leading to moderate yields of compounds **4b**, **4c,** and **4d**, while a OMe substituent in this position no product **4e** was obtained. On the other hand, no significant effect was observed when the substituents were placed in the *meta*- or *para-*position (**4f**–**m**) of the amide group. It must be noted that for substrates bearing a halogen (**1k**–**m**) in the *meta*-position of the amide, a mixture of the two possible regioisomers of compounds **4k**, **4l** and **4m** was obtained. Fortunately, the major isomers (shown in Scheme 3) could be isolated as pure products corresponding to the less hindered isomer, in which the annulation took place in the *para*-position of the halogen. When a Me substituent was present in the *meta*-position, only a single regiosomer of compound **4j** was obtained. It is important to note that when R^2^ is different than H (i.e., iPr or Bn), no products (**4n** and **4o**) were formed, presumably due to the steric hindrance at the neighboring position of the amide and the tetrazole moiety.

**Scheme 4:**
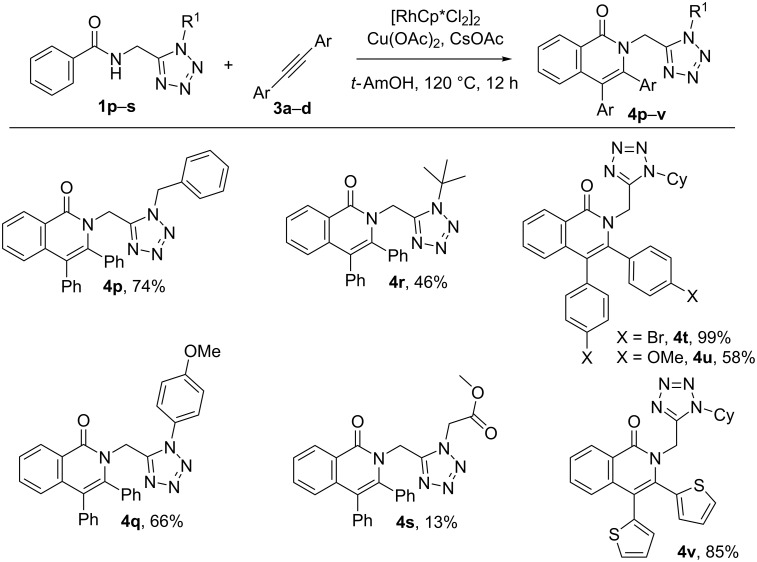
Influence of the alkyne and R^1^ substituent on the reaction outcome.

The effect of the R^1^ substituent at the tetrazole ring and the aryl substituents of the alkyne (Ar, Scheme 1) on the efficiency of the annulation reaction were also evaluated. As shown in Scheme 4, the system is tolerant to a variety of substituents R^1^ (originating from the isocyanide component in the Ugi-azide-4CR, see Scheme 1), with tetrazole/isoquinoline hybrids bearing a benzyl (**4p**), a *p*-methoxyphenyl (**4q**) or a *tert*-butyl (**4r**) group, respectively, being obtained in good yields. However, the presence of an ester functionality at the tetrazole ring (R^1^ = CH_2_CO_2_Me) led to a significant decrease in the yield of compound **4s** (see Scheme 4). A plausible explanation might be that the presence of an additional coordinating ester group may trap the catalyst in a stable complex. Substituted diphenylacetylenes led to good yields of compounds **4t** and **4u**, while 1,2-di(thiophen-2-yl)ethyne also rendered isoquinolone hybrid **4v** in a very good yield. However, the reaction with 1,2-di(pyridin-3-yl)ethyne was not successful, nor was the employment of aliphatic alkynes or the terminal phenylacetylene.

**Scheme 5:**
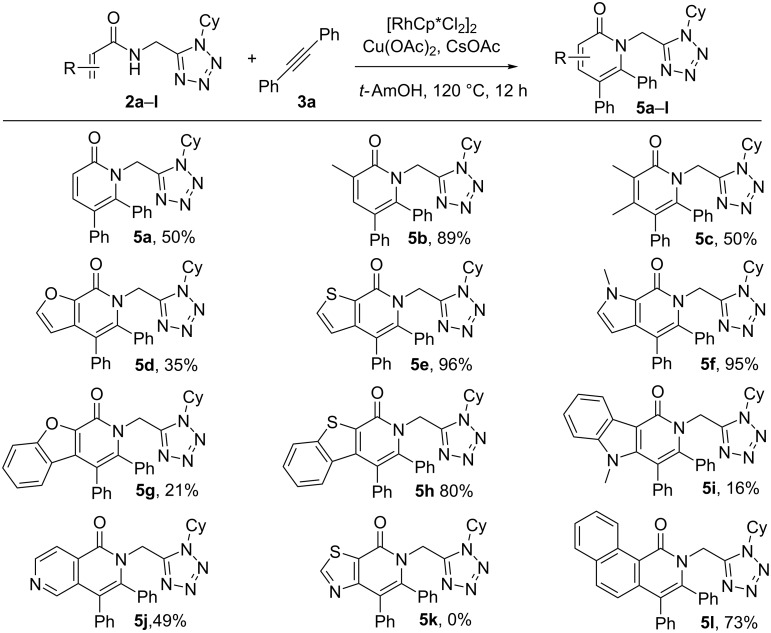
Scope of acrylic, heterocyclic and ring-fused *N*-acylaminomethyl tetrazole substrates.

Finally, aiming at producing tetrazole hybrids including other heterocyclic moieties, the reaction was explored with substrates bearing acyl groups other than benzoyl (Scheme 5). Compounds incorporating acrylamidomethyltetrazole fragments reacted with diphenylacetylene to form the tetrazole/pyridone hybrids **5a**–**c** in good yields. In addition, very complex hybrid compounds based on heterocyclic, fused aromatic rings, such as furan, benzofuran, pyrrole, benzopyrrole, thiophene, benzothiophene and naphthalene were also successfully synthesized (**5d–l**) in moderate to very good yields. The exceptions were the cases of indolopyridone **5i** and thiazolopyridone **5k**, the latter one could not be obtained even after many attempts.

**Scheme 6:**
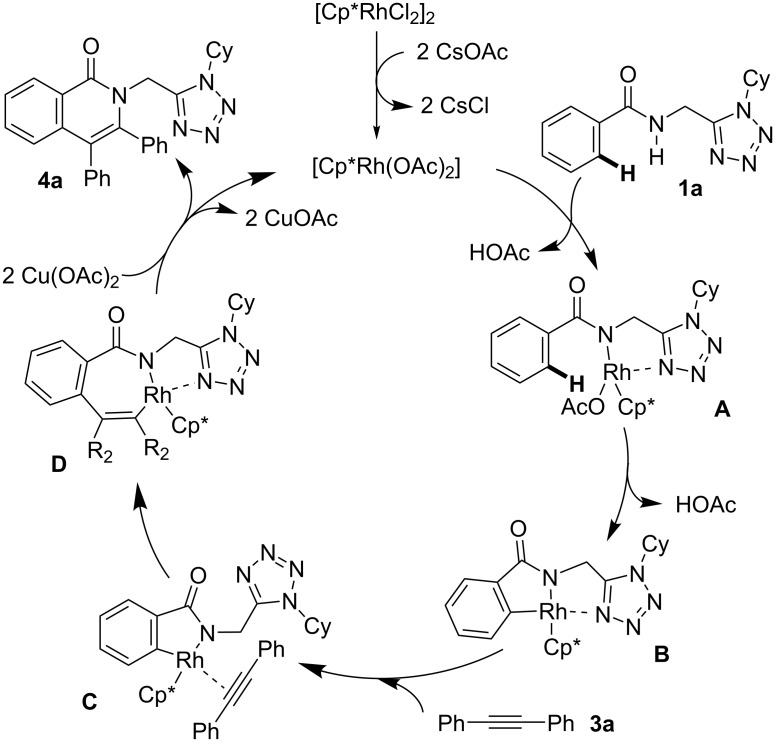
Proposed reaction mechanism using substrates **1a** and **3a**.

To explain the experimental observations of this work and based on literature reports [28,30,32,39,41–42], we propose a mechanism as depicted in Scheme 6. First, [Cp*RhCl_2_]_2_ undergoes ligand exchange with CsOAc to form in situ the active catalyst. This is followed by addition of the substrate **1a** via deprotonation of the amide to form complex **A**, in which it is very likely that a tetrazole nitrogen atom forms a dative bond with the metal center. The next step is the crucial C–H activation of the amide *ortho*-position leading to intermediate **B** with elimination of AcOH. There are examples in the literature supporting the chelation of metal centers by a tetrazole ring during the C–H activation processes, in which the tetrazole may also act as directing group [55–61]. The closely related triazole ring has also been used for the C–H modification of amino acids and peptides and the formation of isoquinolones; so it is expected that in intermediate **B** the substrate behaves as a tridentate ligand for the Rh(III) center [37,62–65]. However, such a complexation must be reversible to allow a further ligand exchange with the acetylene to form intermediate **C**. The subsequent migratory insertion furnishes the seven-membered metallacycle **D**. Finally, reductive elimination leads to compounds **4a** and the concomitant reoxidation of Rh(I) to Rh(III) by the Cu(II) salt completes the catalytic cycle.

## Conclusion

In conclusion, we have developed a versatile method for the preparation of a new family of tetrazole-isoquinolone/pyridone hybrids. The protocol relies on a Rh(III)-catalyzed addition of arylacetylenes to *N*-acylaminomethyltetrazoles derived from the Ugi-azide-4CR. The C–H activation mediated annulation proved to be successful with a variety of benzoyl, acryl and heterocyclic carboxamide moieties, while the *N*-alkyl substituent of the tetrazole ring also proved to show a wide substrate scope. Overall, the reaction sequence is easy to implement and does not require inert conditions. Based on the experimental results, we believe that not only the amido group but also the tetrazole ring takes part in the catalytic reaction mechanism by chelating Rh(III) upon activation of the amide *ortho*-position. This work illustrates the synthetic potential of combining isocyanide-based multicomponent reactions with metal-catalyzed transformations to generate structural diversity and complexity. In addition, the hybrid nature of the final compounds makes them attractive for possible medicinal applications.

## Experimental

### General information

All starting materials were purchased from commercial sources and used without additional purification. Noncommercial arylacetylenes **3c** and **3d** were synthesized according to a reported procedure [38]. ^1^H NMR and ^13^C NMR spectra were recorded on a 400 MHz or 600 MHz apparatus. Chemical shifts (δ) are reported in ppm relative to TMS (^1^H NMR) and to the solvent signal (^13^C NMR). ^13^C NMR peak assignment was accomplished from the DEPT-135 spectra. For thin-layer chromatography, analytical TLC plates (Alugram G/UV254 and 70–230 mesh silica gel (E. M. Merck) were used). Column chromatography was performed using silica gel (Merck, 60–120 mesh size). Solvents for chromatography were used as commercial without previous distillation. The proportion of chromatographic solvents is presented as volume:volume ratios. Isolated compounds were submitted to HRMS using an Agilent 6220A Time of Flight MSD spectrometer equipped with an ESI ionization source. IR spectra were recorded on a Bruker Alpha FTIR spectrometer. Only frequencies (ν, in cm^−1^) of the most relevant peaks are reported. Melting points ranges were recorded on a Reichert Thermovar apparatus and are uncorrected.

### General procedure A for the synthesis of *N*-acylaminomethyltetrazoles

**First step: synthesis of *N*-tritylaminomethyltetrazoles** [66]. Tritylamine (2.0 mmol, 1.0 equiv) and aldehyde (3.0 mmol, 1.5 equiv) components were mixed in EtOH (2.0 mL) in a sealed vial provided with a magnetic stirring bar. The reaction was heated at 100 °C under MW irradiation for 15 minutes. Then, the isocyanide (2.2 mmol, 1.1 equiv) component and azidotrimethylsilane (2.2 mmol, 1.1 equiv, 292 μL) were added into the reaction mixture and further heating followed at 100 °C under MW irradiation for 15 minutes. The solvent was removed under reduced pressure and the residue was used for the next step without any purification.

**Second step: removal of the trityl group** [66]*.* The *N*-trityl-protected α-aminotetrazole obtained in the previous step was dissolved in DCM (10 mL, 0.2 M) in a flask provided with a magnetic stirring bar. TFA (4.0 mmol, 2.0 equiv, 154 μL) was added dropwise at rt and the reaction was allowed to proceed for 1 min. The reaction mixture was concentrated under reduced pressure. The residue was dissolved with a mixture of heptane and EtOAc 1:1 and poured over a silica bed wetted with the same solvent mixture. The side product was washed out with heptane and EtOAc 1:1. The *N*-deprotected aminomethyltetrazole was collected with a mixture of MeOH and DCM 1:1. The solvent was removed under reduced pressure and the residue was used for the next step without any further purification.

**Third step: acylation of deprotected aminomethyltetrazoles.** The *N*-deprotected aminomethyltetrazole obtained in the previous step was dissolved in dried DMF (4.0 mL, 0.5 M) in a flask provided with a magnetic stirring bar. The carboxylic acid (2.0 mmol, 1.0 equiv) and TEA (10 mmol, 5.0 equiv, 1.39 mL) were added and the mixture was cooled to 0 °C. Then, HBTU (2.0 mmol, 1.0 equiv) was added portion-wise and the mixture allowed to slowly reach rt. Then DMAP (0.2 mmol, 10 mol %) was added and the reaction developed for 72 hours. The reaction mixture was poured over 5% aqueous HCl (20 mL) under vigorous stirring. Extractions with AcOEt (3 × 20 mL) followed. The organic layers were combined, washed with saturated aqueous NaHCO_3_ (3 × 20 mL), brine (20 mL) and dried over anhydrous Na_2_SO_4_. The solvent was removed under reduced pressure and the reaction crude was purified by LCC to afford the desired *N*-acylaminomethyltetrazole.

### General procedure B for the rhodium-catalyzed addition of diphenylacetylenes to acylated α-aminotetrazole

The *N*-acylated aminomethyltetrazole (0.25 mmol, 1.0 equiv), arylacetylene (0.375 mmol, 1.5 equiv), Cu(OAc)_2_ (0.5 mmol, 2.0 equiv), CsOAc (0.125 mmol, 0.5 equiv) and [RhCp*Cl_2_]_2_ (0.0125 mmol, 5 mol %) were suspended in *t-*AmOH (1.5 mL, 0.17 M) in a sealed tube containing a magnetic stirring bar. The mixture was reacted under conventional heating in an oil bath at 120 °C for 12 h. After completion of the reaction (checked by TLC), the mixture was diluted with AcOEt (10 mL), filtered through a Celite^®^ pad and washed with additional AcOEt (10 mL). Then the solvent was removed under reduced pressure and the reaction crude was purified by LCC to get the title compound.

## Supporting Information

File 1 Experimental procedures and compound characterization data.
