# Supplementary material for: Combining the Ugi-azide multicomponent reaction and rhodium(III)-catalyzed annulation for the synthesis of tetrazole-isoquinolone/pyridone hybrids
Source: Beilstein J Org Chem. 2019 Oct 16;15:2447–57. doi: 10.3762/bjoc.15.237 (PMC6808192; doi:10.3762/bjoc.15.237)
Supplement: File 1 — Experimental procedures and compound characterization data. [file Beilstein_J_Org_Chem-15-2447-s001.pdf]

BEILSTEIN JOURNAL OF ORGANIC CHEMISTRY

Supporting Information

for

Combining the Ugi-azide multicomponent reaction and rhodium(III)-catalyzed annulation for the synthesis of tetrazole-isoquinolone/pyridone hybrids

Gerardo M. Ojeda, Prabhat Ranjan, Pavel Fedoseev, Lisandra Amable, Upendra K. Sharma, Daniel G. Rivera and Erik V. Van der Eycken

Beilstein J. Org. Chem. **2019,** *15,* 2447–2457. doi:10.3762/bjoc.15.237

Experimental procedures and compound characterization data

License and Terms: This is a supporting information file under the terms of the Creative Commons Attribution License (http://creativecommons.org/licenses/by/4.0). Please note that the reuse, redistribution and reproduction in particular requires that the authors and source are credited.
The license is subject to the *Beilstein Journal of Organic Chemistry* terms and conditions: (https://www.beilstein-journals.org/bjoc)

Synthesis and characterization of intermediates and final compounds

*N-((1-Cyclohexyl-1H-tetrazol-5-yl)methyl)benzamide (**1**)*

$TrtNH_2$ (2.0 mmol, 519 mg) × 5, $(CH_2O)_n$ (3.0 mmol, 90.1 mg) × 5, CyNC (2.2 mmol, 240 mg, 274 μL) × 5, $TMSN_3$ (2.2 mmol, 253 mg, 292 μL) × 5, TFA (20.0 mmol, 2.28 g, 1.53 mL), benzoic acid (10 mmol, 1.22 g), TEA (50 mmol, 5.05 g, 6.97 mL), HBTU (10 mmol, 3.79 mg) and DMAP (1.0 mmol, 122 mg) were reacted according to general procedure A. Purification by LCC afforded compound **1a** as a white solid (2.50 g, 88%). R_f = 0.14 (heptane/AcOEt, 1:1); mp 136-138 °C; FT-IR (KBr) ν 3272 (N-H), 1659 (C=O); ^{1}H NMR (600 MHz, $CDCl_3$) δ 8.02 (t, *J* = 5.7 Hz, 1H), 7.91 – 7.84 (m, 2H), 7.51 (t, *J* = 7.4 Hz, 1H), 7.42 (t, *J* = 7.6 Hz, 2H), 4.95 (d, *J* = 5.8 Hz, 2H), 4.62 (tt, *J* = 11.4, 4.2 Hz, 1H), 2.05 – 1.91 (m, 6H), 1.76 (dt, *J* = 13.7, 3.4 Hz, 1H), 1.47 (dddd, *J* = 17.0, 13.2, 8.4, 3.7 Hz, 2H), 1.32 (dddd, *J* = 16.7, 13.1, 8.3, 3.7 Hz, 1H); ^{13}C {^{1}H} NMR (151 MHz, $CDCl_3$) δ 167.68 (C), 151.78 (C), 132.99 (C), 132.06 (CH), 128.65 (CH), 127.27 (CH), 58.16 (CH), 32.96 (CH_2), 32.53 (CH_2), 25.15 (CH_2), 24.83 (CH_2); HRMS (ESI/TOF) m/z: $[M + H]^+$ Calcd for $C_{15}H_{20}N_5O$ 286.1668, Found 286.1662.

*N-((1-Cyclohexyl-1H-tetrazol-5-yl)methyl)-2-methylbenzamide (**1b**)*

$TrtNH_2$ (2.0 mmol, 519 mg), $(CH_2O)_n$ (3.0 mmol, 90.1 mg), CyNC (2.2 mmol, 240 mg, 274 μL), $TMSN_3$ (2.2 mmol, 253 mg, 292 μL), TFA (4.0 mmol, 456 mg, 306 μL), *o*-methylbenzoic acid (2.0 mmol, 272 mg), TEA (10 mmol, 1.01 g, 1.39 mL), HBTU (2.0 mmol, 758 mg) and DMAP (0.2 mmol, 24.4 mg) were reacted according to general procedure A. Purification by LCC afforded compound **1b** as a light yellow solid (597 mg, 99%). R_f = 0.22 (heptane/AcOEt, 1:1); mp 135-138 °C; FT-IR (KBr) ν 3291 (N-H), 1645 (C=O); ^{1}H NMR (600 MHz, $CDCl_3$) δ 7.41 (d, *J* = 7.6 Hz, 1H), 7.34 (t, *J* = 7.6 Hz, 1H), 7.25 – 7.18 (m, 2H), 7.08 (t, *J* = 5.8 Hz, 1H), 4.90 (d, *J* = 5.8 Hz, 2H), 4.59 (ddd, *J* = 11.2, 6.8, 4.3 Hz, 1H), 2.44 (s, 3H), 2.09 – 1.95 (m, 6H), 1.83 – 1.78 (m, 1H), 1.56 – 1.46 (m, 2H), 1.39 – 1.30 (m, 1H); ^{13}C {^{1}H} NMR (151 MHz, $CDCl_3$) δ 170.09 (C), 151.32 (C), 136.54 (C), 134.58 (C), 131.27 (CH), 130.57 (CH), 126.95 (CH), 125.90 (CH), 58.13 (CH), 32.96 (CH_2), 32.56 (CH_2), 25.19 (CH_2), 24.81 (CH_2); 19.89 (CH_3); HRMS (ESI/TOF) m/z: $[M + H]^+$ Calcd for $C_{16}H_{22}N_5O$ 300.1819, Found 300.1819.

*2-Bromo-N-((1-cyclohexyl-1H-tetrazol-5-yl)methyl)benzamide, (**1c**)*

TrtNH$_2$ (2.0 mmol, 519 mg), $(CH_2O)_n$ (3.0 mmol, 90.1 mg), CyNC (2.2 mmol, 240 mg, 274 μL), TMSN$_3$ (2.2 mmol, 253 mg, 292 μL), TFA (4.0 mmol, 456 mg, 306 μL), *o*-bromobenzoic acid (2.0 mmol, 330 mg), TEA (10 mmol, 1.01 g, 1.39 mL), HBTU (2.0 mmol, 758 mg) and DMAP (0.2 mmol, 24.4 mg) were reacted according to general procedure A. Purification by LCC afforded compound **1c** as a salmon-colored solid (549 mg, 75%). R_f = 0.28 (heptane/AcOEt, 1:2); mp 164-166 °C; FT-IR (KBr) ν 3249 (N-H), 1659 (C=O); ^{1}H NMR (600 MHz, $CDCl_3$) δ 7.60 (d, *J* = 8.0 Hz, 1H), 7.53 (dd, *J* = 7.7, 1.8 Hz, 1H), 7.37 (t, *J* = 7.5 Hz, 1H), 7.33 – 7.27 (m, 2H), 4.94 (d, *J* = 5.8 Hz, 2H), 4.62 (tt, *J* = 11.5, 4.1 Hz, 1H), 2.11 – 1.94 (m, 6H), 1.83 – 1.78 (m, 1H), 1.52 (qt, *J* = 13.2, 3.9 Hz, 2H), 1.35 (ddt, *J* = 16.6, 13.1, 6.6 Hz, 1H); ^{13}C {^{1}H} NMR (151 MHz, $CDCl_3$) δ 167.87 (C), 150.92 (C), 136.34 (C), 133.57 (CH), 131.83 (CH), 129.47 (CH), 127.66 (CH), 119.38 (C), 58.20 (CH), 33.00 (CH_2), 32.62 (CH_2), 25.20 (CH_2), 24.82 (CH_2); HRMS (ESI/TOF) m/z: $[M + H]^+$ Calcd for $C_{15}H_{19}BrN_5O$ 364.0768, Found 364.0756.

2-Chloro-N-((1-cyclohexyl-1H-tetrazol-5-yl)methyl)benzamide ***(1d)***

TrtNH$_2$ (2.0 mmol, 519 mg), $(CH_2O)_n$ (3.0 mmol, 90.1 mg), CyNC (2.2 mmol, 240 mg, 274 μL), TMSN$_3$ (2.2 mmol, 253 mg, 292 μL), TFA (4.0 mmol, 456 mg, 306 μL), *o*-chlorobenzoic acid (2.0 mmol, 313 mg), TEA (10 mmol, 1.01 g, 1.39 mL), HBTU (2.0 mmol, 758 mg) and DMAP (0.2 mmol, 24.4 mg) were reacted according to general procedure A. Purification by LCC afforded compound **1d** as a white solid (496 mg, 78%). R_f = 0.28 (heptane/AcOEt, 1:2); mp 161-164 °C; FT-IR (KBr) ν 3253 (N-H), 1661 (C=O); ^{1}H NMR (600 MHz, $CDCl_3$) δ 7.64 (d, *J* = 7.6 Hz, 1H), 7.47 – 7.37 (m, 3H), 7.36 – 7.31 (m, 1H), 4.95 (d, *J* = 5.7 Hz, 2H), 4.60 (tt, *J* = 11.4, 4.3 Hz, 1H), 2.10 – 1.93 (m, 6H), 1.82 – 1.30 (m, 1H), 1.51 (dtd, *J* = 17.0, 13.2, 4.1 Hz, 2H), 1.35 (dddd, *J* = 17.0, 13.4, 8.5, 3.8 Hz, 1H); ^{13}C {^{1}H} NMR (151 MHz, $CDCl_3$) δ 166.80 (C), 150.98 (C), 133.71 (C), 131.89 (CH); 130.90 (C), 130.44 (CH), 129.98 (CH), 127.18 (CH), 58.18 (CH), 32.97 (CH_2), 32.70 (CH_2), 25.19 (CH_2), 24.82 (CH_2); HRMS (ESI/TOF) m/z: $[M + H]^+$ Calcd for $C_{15}H_{19}ClN_5O$ 320.1273, Found 320.1267.

N-((1-Cyclohexyl-1H-tetrazol-5-yl)methyl)-2-methoxybenzamide ***(1e)***

TrtNH$_2$ (2.0 mmol, 519 mg), $(CH_2O)_n$ (3.0 mmol, 90.1 mg), CyNC (2.2 mmol, 240 mg, 274 μL), TMSN$_3$ (2.2 mmol, 253 mg, 292 μL), TFA (4.0 mmol, 456 mg, 306 μL), *o*-anisic acid (2.0 mmol, 304 mg), TEA (10 mmol, 1.01 g, 1.39 mL), HBTU (2.0 mmol, 758 mg) and DMAP (0.2 mmol, 24.4 mg) were reacted according to general procedure A. Purification by LCC afforded compound

1e as a white solid (544 mg, 86%). R_f = 0.24 (heptane/AcOEt, 1:2); mp 119-122 °C; FT-IR (KBr) ν 3342 (N-H), 1636 (C=O); ^{1}H NMR (400 MHz, $CDCl_3$) δ 8.64 (t, *J* = 5.9 Hz, 1H), 8.20 (dd, *J* = 7.8, 1.9 Hz, 1H), 7.57 – 7.40 (m, 1H), 7.09 (t, *J* = 7.6 Hz, 1H), 6.99 (d, *J* = 8.4 Hz, 1H), 4.97 (d, *J* = 5.7 Hz, 2H), 4.66 – 4.52 (m, 1H), 3.98 (s, 3H), 2.05 – 1.87 (m, 6H), 1.75 (dt, *J* = 13.0, 3.4 Hz, 1H), 1.54 – 1.39 (m, 2H), 1.32 (tt, *J* = 12.8, 3.4 Hz, 1H); ^{13}C {^{1}H} NMR (101 MHz, $CDCl_3$) δ 165.52 (C), 157.70 (C), 151.70 (C), 133.58 (CH), 132.31 (CH_2), 121.33 (CH), 120.10 (C), 111.44 (CH), 58.00 (CH), 56.07 (CH_3), 32.93 (CH_2), 32.51 (CH_2), 25.18 (CH_2), 24.85 (CH_2); HRMS (ESI/TOF) m/z: $[M + H]^+$ Calcd for $C_{16}H_{22}N_5O_2$ 316.1768, Found 316.1773.

*N-((1-Cyclohexyl-1H-tetrazol-5-yl)methyl)-4-methoxybenzamide (**1f**)*

$TrtNH_2$ (2.0 mmol, 519 mg), $(CH_2O)_n$ (3.0 mmol, 90.1 mg), CyNC (2.2 mmol, 240 mg, 274 μL), $TMSN_3$ (2.2 mmol, 253 mg, 292 μL), TFA (4.0 mmol, 456 mg, 306 μL), *p*-anisic acid (2.0 mmol, 304 mg), TEA (10 mmol, 1.01 g, 1.39 mL), HBTU (2.0 mmol, 758 mg) and DMAP (0.2 mmol, 24.4 mg) were reacted according to general procedure A. Purification by LCC afforded compound **1f** as a white solid (563 mg, 89%). R_f = 0.22 (heptane/AcOEt, 1:2); mp 171-174 °C; FT-IR (KBr) ν 3300 (N-H), 1643 (C=O); ^{1}H NMR (400 MHz, DMSO-*d*6) δ 7.94 (t, *J* = 5.8 Hz, 1H), 7.85 (d, *J* = 8.4 Hz, 2H), 6.88 (d, *J* = 8.5 Hz, 2H), 4.92 (d, *J* = 5.8 Hz, 2H), 4.61 (tt, *J* = 11.1, 4.4 Hz, 1H), 3.83 (s, 3H), 2.07 – 1.88 (m, 6H), 1.75 (dt, *J* = 13.1, 3.5 Hz, 1H), 1.46 (qt, *J* = 12.9, 3.9 Hz, 2H), 1.31 (tt, *J* = 13.1, 3.6 Hz, 1H); ^{13}C {^{1}H} NMR (101 MHz, DMSO-*d*6) δ 167.17 (C), 162.62 (C), 151.99 (C), 129.19 (CH), 125.26 (C), 113.84 (CH), 58.14 (CH), 55.41 (CH_3), 32.97 (CH_2), 32.48 (CH_2), 25.16 (CH_2), 24.85 (CH_2); HRMS (ESI/TOF) m/z: $[M + H]^+$ Calcd for $C_{16}H_{22}N_5O_2$ 316.1768, Found 316.1763.

*N-((1-Cyclohexyl-1H-tetrazol-5-yl)methyl)-4-methylbenzamide (**1g**)*

$TrtNH_2$ (4.0 mmol, 1.04 g), $(CH_2O)_n$ (6.0 mmol, 180 mg), CyNC (4.4 mmol, 480 mg, 548 μL), $TMSN_3$ (4.4 mmol, 506 mg, 584 μL), TFA (8.0 mmol, 912 mg, 612 μL), *p*-toluic acid (4.0 mmol, 608 mg), TEA (20 mmol, 2.02 g, 2.78 mL), HBTU (4.0 mmol, 1.52 g) and DMAP (0.4 mmol, 48.8 mg) were reacted according to general procedure A. Purification by LCC afforded compound **1g** as a white solid (1.02 g, 85%). R_f = 0.20 (heptane/AcOEt, 1:1); mp 172-174 °C; FT-IR (KBr) ν 3356 (N-H), 1650 (C=O); ^{1}H NMR (400 MHz, $CDCl_3$) δ 7.89 (t, *J* = 5.8 Hz, 1H), 7.76 (d, *J* = 7.9 Hz, 2H), 7.20 (d, *J* = 7.9 Hz, 2H), 4.93 (d, *J* = 5.8 Hz, 2H), 4.60 (tt, *J* = 10.7, 4.5 Hz, 1H), 2.37 (s, 3H), 2.03 – 1.96 (m, 3H), 1.96 – 1.86 (m, 3H), 1.75 (dt, *J* = 13.4, 3.3 Hz, 1H), 1.46 (qt, *J* = 12.8,

4.0 Hz, 2H), 1.31 (tt, *J* = 12.9, 3.5 Hz, 1H); ^{13}C {^{1}H} NMR (151 MHz, $CDCl_3$) δ 167.60 (C), 151.83 (C), 142.59 (C), 130.17 (C), 129.31 (CH), 127.26 (CH), 58.12(CH), 32.95 (CH_2), 32.48 (CH_2), 25.15 (CH_2), 24.84 (CH_2), 21.47 (CH_3); HRMS (ESI/TOF) m/z: $[M + H]^+$ Calcd for $C_{16}H_{22}N_5O$ 300.1819, Found 300.1812.

*4-Chloro-N-((1-cyclohexyl-1H-tetrazol-5-yl)methyl)benzamide (**1h**)*

$TrtNH_2$ (2.0 mmol, 519 mg), $(CH_2O)_n$ (3.0 mmol, 90.1 mg), CyNC (2.2 mmol, 240 mg, 274 µL), $TMSN_3$ (2.2 mmol, 253 mg, 292 µL), TFA (4.0 mmol, 456 mg, 306 µL), *p*-chlorobenzoic acid (2.0 mmol, 313 mg), TEA (10 mmol, 1.01 g, 1.39 mL), HBTU (2.0 mmol, 758 mg) and DMAP (0.2 mmol, 24.4 mg) were reacted according to general procedure A. Purification by LCC afforded compound **1h** as a white solid (523 mg, 82%). R_f = 0.24 (heptane/AcOEt, 1:1); mp 180-182 °C; FT-IR (KBr) ν 3341 (N-H), 1655 (C=O); ^{1}H NMR (400 MHz, DMSO-*d*6) δ 9.37 (t, *J* = 5.8 Hz, 1H), 7.90 (d, *J* = 8.1 Hz, 2H), 7.57 (d, *J* = 8.2 Hz, 2H), 4.83 (d, *J* = 5.5 Hz, 2H), 4.61 (tt, *J* = 11.4, 3.9 Hz, 1H), 1.99 (dt, *J* = 9.7, 4.3 Hz, 2H), 1.79 (qd, *J* = 12.0, 11.6, 3.5 Hz, 4H), 1.67 (d, *J* = 12.8 Hz, 1H), 1.39 (qd, *J* = 13.6, 11.9, 6.8 Hz, 2H), 1.32 – 1.21 (m, 1H); ^{13}C {^{1}H} NMR (101 MHz, DMSO-*d*6) δ 165.95 (C), 152.77 (C), 137.03 (C), 132.55 (C), 129.68 (CH), 129.04 (CH), 57.18 (CH), 32.92 (CH_2), 32.84 (CH_2), 25.16 (CH_2), 25.00 (CH_2); HRMS (ESI/TOF) m/z: $[M + H]^+$ Calcd for $C_{15}H_{19}ClN_5O$ 320.1273, Found 320.1258.

*N-((1-Cyclohexyl-1H-tetrazol-5-yl)methyl)-4-(trifluoromethyl)benzamide (**1i**)*

$TrtNH_2$ (2.0 mmol, 519 mg), $(CH_2O)_n$ (3.0 mmol, 90.1 mg), CyNC (2.2 mmol, 240 mg, 274 µL), $TMSN_3$ (2.2 mmol, 253 mg, 292 µL), TFA (4.0 mmol, 456 mg, 306 µL), 4-trifluoromethylbenzoic acid (2.0 mmol, 380 mg), TEA (10 mmol, 1.01 g, 1.39 mL), HBTU (2.0 mmol, 758 mg) and DMAP (0.2 mmol, 24.4 mg) were reacted according to general procedure A. Purification by LCC afforded compound **1i** as a white solid (547 mg, 77%). R_f = 0.28 (heptane/AcOEt, 1:1); mp 171-174 °C; FT-IR (KBr) ν 3321 (N-H), 1659 (C=O); ^{1}H NMR (400 MHz, DMSO-*d*6) δ 9.54 (t, *J* = 5.6 Hz, 1H), 8.08 (d, *J* = 8.0 Hz, 2H), 7.88 (d, *J* = 8.1 Hz, 2H), 4.87 (d, *J* = 5.6 Hz, 2H), 4.62 (tt, *J* = 11.3, 4.0 Hz, 1H), 2.06 – 1.96 (m, 2H), 1.89 – 1.74 (m, 4H), 1.71 – 1.63 (m, 1H), 1.39 (qd, *J* = 12.0, 10.5, 6.7 Hz, 2H), 1.26 (tt, *J* = 12.5, 2.9 Hz, 1H); ^{13}C {^{1}H} NMR (151 MHz, DMSO-*d*6) δ 165.85 (C), 152.67 (C), 137.55 (C), 132.00 (q, *J* = 31.9 Hz, CF_3), 128.68 (CH), 127.03 (C), 125.97 (q, *J* = 3.8 Hz, CH), 125.22 (C), 123.41 (C), 121.61 (C); 57.19 (CH), 32.91 (CH_2), 25.14 (CH_2),

24.98 (CH_2); ^{19}F {^{13}C} NMR (376 MHz, $CDCl_3$) δ -56.71; HRMS (ESI/TOF) m/z: $[M + H]^+$ Calcd for $C_{16}H_{19}F_3N_5O$ 354.1536, Found 354.1520.

*N-((1-Cyclohexyl-1H-tetrazol-5-yl)methyl)-3-methylbenzamide (**1j**)*

$TrtNH_2$ (2.0 mmol, 519 mg), $(CH_2O)_n$ (3.0 mmol, 90.1 mg), CyNC (2.2 mmol, 240 mg, 274 µL), $TMSN_3$ (2.2 mmol, 253 mg, 292 µL), TFA (4.0 mmol, 456 mg, 306 µL), *m*-toluic acid (2.0 mmol, 272 mg), TEA (10 mmol, 1.01 g, 1.39 mL), HBTU (2.0 mmol, 758 mg) and DMAP (0.2 mmol, 24.4 mg) were reacted according to general procedure A. Purification by LCC afforded compound **1j** as a white solid (467 mg, 78%). R_f = 0.24 (heptane/AcOEt, 1:1); mp 122-124 °C; FT-IR (KBr) ν 3276 (N-H), 1635 (C=O); ^1H NMR (400 MHz, $CDCl_3$) δ 7.94 (t, *J* = 5.8 Hz, 1H), 7.67 (s, 1H), 7.66 – 7.60 (m, 1H), 7.29 (d, *J* = 7.0 Hz, 2H), 4.93 (d, *J* = 5.8 Hz, 2H), 4.61 (tt, *J* = 11.0, 4.5 Hz, 1H), 2.35 (s, 3H), 2.00 (dd, *J* = 11.7, 3.5 Hz, 3H), 1.97 – 1.88 (m, 3H), 1.76 (dt, *J* = 13.2, 3.3 Hz, 1H), 1.47 (qt, *J* = 12.9, 4.0 Hz, 2H), 1.32 (tt, *J* = 12.8, 3.5 Hz, 1H); ^{13}C {^1H} NMR (151 MHz, $CDCl_3$) δ 167.86 (C), 151.78 (C), 138.53 (C), 132.94 (C), 132.81 (CH), 128.53 (CH), 127.93 (CH), 124.25 (CH), 58.15 (CH), 32.96 (CH_2), 32.54 (CH_2), 25.16 (CH_2), 24.84 (CH_2), 21.27 (CH_3); HRMS (ESI/TOF) m/z: $[M + H]^+$ Calcd for $C_{16}H_{22}N_5O$ 300.1819, Found 300.1812.

*3-Chloro-N-((1-cyclohexyl-1H-tetrazol-5-yl)methyl)benzamide (**1k**)*

$TrtNH_2$ (4.0 mmol, 1.04 g), $(CH_2O)_n$ (6.0 mmol, 180 mg), CyNC (4.4 mmol, 480 mg, 548 µL), $TMSN_3$ (4.4 mmol, 506 mg, 584 µL), TFA (8.0 mmol, 912 mg, 612 µL), *m*-chlorobenzoic acid (4.0 mmol, 626 mg), TEA (20 mmol, 2.02 g, 2.78 mL), HBTU (4.0 mmol, 1.52 g) and DMAP (0.4 mmol, 48.8 mg) were reacted according to general procedure A. Purification by LCC afforded compound **1k** as a light yellow solid (1.05 g, 82%). R_f = 0.22 (heptane/AcOEt, 1:1); mp 140-143 °C; FT-IR (KBr) ν 3268 (N-H), 1658 (C=O); ^1H NMR (400 MHz, $CDCl_3$) δ 8.21 (t, *J* = 5.8 Hz, 1H), 7.85 (d, *J* = 1.9 Hz, 1H), 7.73 (d, *J* = 7.7 Hz, 1H), 7.46 (dd, *J* = 8.0, 2.0 Hz, 1H), 7.33 (t, *J* = 7.9 Hz, 1H), 4.92 (d, *J* = 5.8 Hz, 2H), 4.64 – 4.53 (m, 1H), 2.03 (td, *J* = 11.2, 10.3, 4.5 Hz, 3H), 1.99 – 1.90 (m, 3H), 1.77 (dt, *J* = 13.2, 3.3 Hz, 1H), 1.48 (qt, *J* = 12.8, 4.1 Hz, 2H), 1.33 (tt, *J* = 12.7, 3.4 Hz, 1H); ^{13}C {^1H} NMR (151 MHz, $CDCl_3$) δ 166.36 (C), 151.67 (C), 134.88 (C), 134.64 (C), 132.09 (CH), 129.94 (CH), 127.78 (CH), 125.19 (CH), 58.29 (CH), 32.95 (CH_2), 32.68 (CH_2), 25.17 (CH_2), 24.81 (CH_2); HRMS (ESI/TOF) m/z: $[M + H]^+$ Calcd for $C_{15}H_{19}ClN_5O$ 320.1273, Found 320.1266.

*3-Bromo-N-((1-cyclohexyl-1H-tetrazol-5-yl)methyl)benzamide (**1l**)*

$TrtNH_2$ (4.0 mmol, 1.04 g), $(CH_2O)_n$ (6.0 mmol, 180 mg), CyNC (4.4 mmol, 480 mg, 548 μL), $TMSN_3$ (4.4 mmol, 506 mg, 584 μL), TFA (8.0 mmol, 912 mg, 612 μL), *m*-bromobenzoic acid (4.0 mmol, 804 mg), TEA (20 mmol, 2.02 g, 2.78 mL), HBTU (4.0 mmol, 1.52 g) and DMAP (0.4 mmol, 48.8 mg) were reacted according to general procedure A. Purification by LCC afforded compound **1l** as a light yellow solid (1.20 g, 82%). R_f = 0.22 (heptane/AcOEt, 1:1); mp 131-134 °C; FT-IR (KBr) ν 3237 (N-H), 1636 (C=O); ^{1}H NMR (400 MHz, $CDCl_3$) δ 8.23 (t, *J* = 5.8 Hz, 1H), 8.00 (s, 1H), 7.78 (d, *J* = 7.8 Hz, 1H), 7.61 (dd, *J* = 7.9, 1.8 Hz, 1H), 7.26 (t, *J* = 7.9 Hz, 1H), 4.92 (d, *J* = 5.8 Hz, 2H), 4.59 (tt, *J* = 10.4, 4.6 Hz, 1H), 2.07 – 1.92 (m, 6H), 1.77 (dt, *J* = 13.0, 3.5 Hz, 1H), 1.48 (qt, *J* = 12.7, 4.1 Hz, 2H), 1.33 (tt, *J* = 12.8, 3.5 Hz, 1H); ^{13}C {^{1}H} NMR (151 MHz, $CDCl_3$) δ 166.25 (C), 151.68 (C), 135.00 (CH), 134.81 (C), 130.67 (CH), 130.18 (CH), 125.67 (CH), 122.86 (C), 58.29 (CH), 32.95 (CH_2), 32.67 (CH_2), 25.17 (CH_2), 24.81 (CH_2); HRMS (ESI/TOF) m/z: $[M + H]^+$ Calcd for $C_{15}H_{19}BrN_5O$ 364.0768, Found 364.0762.

*N-((1-Cyclohexyl-1H-tetrazol-5-yl)methyl)-3-iodobenzamide (**1m**)*

$TrtNH_2$ (4.0 mmol, 1.04 g), $(CH_2O)_n$ (6.0 mmol, 180 mg), CyNC (4.4 mmol, 480 mg, 548 μL), $TMSN_3$ (4.4 mmol, 506 mg, 584 μL), TFA (8.0 mmol, 912 mg, 612 μL), *m*-iodobenzoic acid (4.0 mmol, 992 mg), TEA (20 mmol, 2.02 g, 2.78 mL), HBTU (4.0 mmol, 1.52 g) and DMAP (0.4 mmol, 48.8 mg) were reacted according to general procedure A. Purification by LCC afforded compound **1m** as a light yellow solid (1.38 g, 84%). R_f = 0.22 (heptane/AcOEt, 1:1); mp 141-146 °C; FT-IR (KBr) ν 3240 (N-H), 1633 (C=O); ^{1}H NMR (400 MHz, $CDCl_3$) δ 8.18 (s, 1H), 8.08 (t, *J* = 5.8 Hz, 1H), 7.81 (t, *J* = 8.3 Hz, 2H), 7.13 (t, *J* = 7.8 Hz, 1H), 4.91 (d, *J* = 5.7 Hz, 2H), 4.58 (tt, *J* = 10.2, 4.7 Hz, 1H), 2.11 – 1.90 (m, 6H), 1.80 – 1.74 (m, 1H), 1.48 (dddd, *J* = 16.6, 12.8, 9.9, 4.2 Hz, 2H), 1.34 (tt, *J* = 12.7, 3.4 Hz, 1H); ^{13}C {^{1}H} NMR (151 MHz, $CDCl_3$) δ 166.11 (C), 151.60 (C), 140.94 (CH), 136.43 (CH), 134.78 (C), 130.28 (CH), 126.25 (CH), 94.34 (C), 58.30 (CH), 32.96 (CH_2), 32.71 (CH_2), 25.18 (CH_2), 24.82 (CH_2); HRMS (ESI/TOF) m/z: $[M + H]^+$ Calcd for $C_{15}H_{19}IN_5O$ 411.0631, Found 412.0611.

*N-(1-(1-Cyclohexyl-1H-tetrazol-5-yl)-2-methylpropyl)benzamide (**1n**)*

$TrtNH_2$ (2.0 mmol, 519 mg), isobutyraldehyde (3.0 mmol, 216 mg, 274 μL), CyNC (2.2 mmol, 240 mg, 274 μL), $TMSN_3$ (2.2 mmol, 253 mg, 292 μL), TFA (4.0 mmol, 456 mg, 306 μL), benzoic acid (2.0 mmol, 244 mg), TEA (10 mmol, 1.01 g, 1.39 mL), HBTU (2.0 mmol, 758 mg) and DMAP (0.2 mmol, 24.4 mg) were reacted according to general procedure A. Purification by LCC

afforded compound **1n** as a white solid (370 mg, 57%). R_f = 0.20 (heptane/AcOEt, 3:1); mp 207-209 °C; FT-IR (KBr) ν 3307 (N-H), 1649 (C=O); ^{1}H NMR (400 MHz, $CDCl_3$) δ 7.80 (d, *J* = 7.6 Hz, 3H), 7.46 (t, *J* = 7.4 Hz, 1H), 7.36 (t, *J* = 7.6 Hz, 2H), 5.25 (t, *J* = 9.1 Hz, 1H), 4.63 (tt, *J* = 11.7, 4.0 Hz, 1H), 2.42 (dq, *J* = 9.2, 6.5 Hz, 1H), 2.11 (ddt, *J* = 12.1, 8.3, 3.9 Hz, 2H), 2.04 – 1.88 (m, 4H), 1.82 – 1.73 (m, 1H), 1.49 (dtp, *J* = 16.2, 7.8, 3.8 Hz, 2H), 1.35 (tt, *J* = 12.9, 3.5 Hz, 1H), 1.15 (d, *J* = 6.7 Hz, 3H), 0.78 (d, *J* = 6.6 Hz, 3H); ^{13}C {^{1}H} NMR (151 MHz, $CDCl_3$) δ 167.54 (C), 154.64 (C), 148.47 (C), 133.29 (C), 133.14 (CH), 131.94 (CH), 130.06 (CH), 128.54 (CH), 128.35 (CH), 128.12 (CH), 127.91 (CH), 127.27 (CH), 126.62 (CH), 58.03 (CH), 49.29 (CH), 33.21 (CH_2), 33.16 (CH_2), 32.40 (CH), 25.33 (CH_2), 25.25 (CH_2), 24.87 (CH_2), 19.50 (CH_3), 19.03 (CH_3); HRMS (ESI/TOF) m/z: $[M + H]^+$ Calcd for $C_{18}H_{26}N_5O$ 328.2132, Found 328.2127.

*N-(1-(1-Cyclohexyl-1H-tetrazol-5-yl)-2-phenylethyl)benzamide (**1o**)*

$TrtNH_2$ (2.0 mmol, 519 mg), phenylacetaldehyde (3.0 mmol, 360 mg, 348 μL), CyNC (2.2 mmol, 240 mg, 274 μL), $TMSN_3$ (2.2 mmol, 253 mg, 292 μL), TFA (4.0 mmol, 456 mg, 306 μL), benzoic acid (2.0 mmol, 244 mg), TEA (10 mmol, 1.01 g, 1.39 mL), HBTU (2.0 mmol, 758 mg) and DMAP (0.2 mmol, 24.4 mg) were reacted according to general procedure A. Purification by LCC afforded compound **1o** as a yellow solid (335 mg, 45%). R_f = 0.18 (heptane/AcOEt, 3:1); mp 196-198 °C; FT-IR (KBr) ν 3313 (N-H), 1654 (C=O); ^{1}H NMR (400 MHz, $CDCl_3$) δ 8.02 (p, *J* = 8.6 Hz, 1H), 7.81 (d, *J* = 7.6 Hz, 2H), 7.50 (t, *J* = 7.4 Hz, 1H), 7.39 (t, *J* = 7.6 Hz, 2H), 7.21 – 7.13 (m, 3H), 6.98 – 6.88 (m, 2H), 5.66 (ddd, *J* = 10.6, 8.2, 5.2 Hz, 1H), 3.96 (tt, *J* = 8.3, 3.8 Hz, 1H), 3.51 (dd, *J* = 12.9, 5.3 Hz, 1H), 3.31 (dd, *J* = 13.0, 10.6 Hz, 1H), 2.05 (d, *J* = 12.4 Hz, 1H), 1.90 – 1.56 (m, 5H), 1.43 – 1.28 (m, 1H), 1.27 – 1.03 (m, 2H), 0.87 (d, *J* = 12.4 Hz, 1H); ^{13}C {^{1}H} NMR (151 MHz, $CDCl_3$) δ 167.36 (C), 154.50 (C), 135.51 (C), 133.14 (C), 132.02 (CH), 130.10 (CH), 129.26 (CH), 128.84 (CH), 128.61 (CH), 128.40 (CH), 128.12 (CH), 127.90 (CH), 127.40 (CH), 127.36 (CH), 126.60 (CH), 58.06 (CH), 46.18 (CH), 41.11 (CH_2), 32.99 (CH_2), 32.19 (CH_2), 25.22 (CH_2), 24.79 (CH_2); HRMS (ESI/TOF) m/z: $[M + H]^+$ Calcd for $C_{22}H_{26}N_5O$ 376.2059, Found 376.2125.

*N-((1-Benzyl-1H-tetrazol-5-yl)methyl)benzamide (**1p**)*

$TrtNH_2$ (4.0 mmol, 1.04 g), $(CH_2O)_n$ (6.0 mmol, 180 mg), BnNC (4.4 mmol, 516 mg, 536 μL), $TMSN_3$ (4.4 mmol, 506 mg, 584 μL), TFA (8.0 mmol, 912 mg, 612 μL), benzoic acid (4.0 mmol, 488 mg), TEA (20 mmol, 2.02 g, 2.78 mL), HBTU (4.0 mmol, 1.52 g) and DMAP (0.4 mmol,

48.8 mg) were reacted according to general procedure A. Purification by LCC afforded compound **1p** as a light yellow solid (892 mg, 76%). R_f = 0.20 (heptane/AcOEt, 1:1); mp 105 °C; FT-IR (KBr) ν 3289 (N-H), 1635 (C=O); ^{1}H NMR (600 MHz, $CDCl_3$) δ 8.11 (t, *J* = 5.9 Hz, 1H), 7.82 – 7.78 (m, 2H), 7.51 – 7.46 (m, 1H), 7.37 (t, *J* = 7.7 Hz, 2H), 7.35 – 7.29 (m, 5H), 5.82 (s, 2H), 4.83 (d, *J* = 5.9 Hz, 2H); ^{13}C {^{1}H} NMR (151 MHz, $CDCl_3$) δ 167.95 (C), 152.85 (C), 133.55 (C), 132.76 (C), 132.10 (CH), 129.20 (CH), 128.89 (CH), 128.60 (CH), 127.80 (CH), 127.30 (CH), 51.21 (CH_2), 32.41 (CH_2); HRMS (ESI/TOF) m/z: $[M + H]^+$ Calcd for $C_{16}H_{16}N_5O$ 294.1349, Found 294.1352.

*N-((1-(4-Methoxyphenyl)-1H-tetrazol-5-yl)methyl)benzamide (**1q**)*

$TrtNH_2$ (4.0 mmol, 1.04 g), $(CH_2O)_n$ (6.0 mmol, 180 mg), *p*-methoxyphenylisocyanide (4.4 mmol, 586 mg), $TMSN_3$ (4.4 mmol, 506 mg, 584 μL), TFA (8.0 mmol, 912 mg, 612 μL), benzoic acid (4.0 mmol, 488 mg), TEA (20 mmol, 2.02 g, 2.78 mL), HBTU (4.0 mmol, 1.52 g) and DMAP (0.4 mmol, 48.8 mg) were reacted according to general procedure A. Purification by LCC afforded compound **1q** as a light brown solid (992 mg, 80%). R_f = 0.24 (heptane/AcOEt, 1:2); mp 133-134 °C; FT-IR (KBr) ν 3290 (N-H), 1635 (C=O); ^{1}H NMR (300 MHz, $CDCl_3$) δ 8.02 (t, *J* = 5.5 Hz, 1H), 7.79 – 7.71 (m, 2H), 7.55 – 7.39 (m, 3H), 7.38 – 7.29 (m, 2H), 7.08 – 7.00 (m, 2H), 4.85 (d, *J* = 5.5 Hz, 2H), 3.86 (s, 3H); ^{13}C {^{1}H} NMR (75 MHz, $CDCl_3$) δ 167.68 (C), 161.17 (C), 153.00 (C), 132.83 (C), 131.90 (CH), 128.42 (CH), 127.20 (CH), 126.49 (CH), 125.81 (C), 115.10 (CH), 55.64 (CH_3), 33.88 (CH_2); HRMS (ESI/TOF) m/z: Not found.

*N-((1-(tert-Butyl)-1H-tetrazol-5-yl)methyl)benzamide (**1r**)*

$TrtNH_2$ (2.0 mmol, 519 mg), $(CH_2O)_n$ (3.0 mmol, 90.1 mg), *t*BuNC (2.2 mmol, 183 mg, 249 μL), $TMSN_3$ (2.2 mmol, 253 mg, 292 μL), TFA (4.0 mmol, 456 mg, 306 μL), benzoic acid (2.0 mmol, 244 mg), TEA (10 mmol, 1.01 g, 1.39 mL), HBTU (2.0 mmol, 758 mg) and DMAP (0.2 mmol, 24.4 mg) were reacted according to general procedure A. Purification by LCC afforded compound **1r** as a white oil (344 mg, 66%) that solidifies upon standing. R_f = 0.26 (heptane/AcOEt, 1:2); mp 80-81 °C; FT-IR (KBr) ν 3265 (N-H), 1656 (C=O); ^{1}H NMR (400 MHz, $CDCl_3$) δ 8.00 (t, *J* = 5.1 Hz, 1H), 7.88 – 7.79 (m, 2H), 7.51 – 7.44 (m, 1H), 7.38 (dd, *J* = 8.4, 7.0 Hz, 2H), 5.01 (d, *J* = 5.1 Hz, 2H), 1.77 (s, 9H); ^{13}C {^{1}H} NMR (101 MHz, $CDCl_3$) δ 167.56 (C), 151.76 (C), 133.05 (C), 131.95 (CH), 128.58 (CH), 127.23 (CH), 61.57 (C), 35.84 (CH_2), 29.54(CH_3); HRMS (ESI/TOF) m/z: $[M + H]^+$ Calcd for $C_{13}H_{18}N_5O$ 260.1506, Found 260.1514.

*Methyl 2-(5-(benzamidomethyl)-1H-tetrazol-1-yl)acetate **(1s)***

$TrtNH_2$ (2.0 mmol, 519 mg) × 5, $(CH_2O)_n$ (3.0 mmol, 90.1 mg) × 5, *t*BuNC (2.2 mmol, 183 mg, 249 μL) × 5, $TMSN_3$ (2.2 mmol, 253 mg, 292 μL) × 5, TFA (20.0 mmol, 2.28 g, 1.53 mL), benzoic acid (10 mmol, 1.22 g), TEA (50 mmol, 5.05 g, 6.97 mL), HBTU (10 mmol, 3.79 g) and DMAP (1.0 mmol, 122 mg) were reacted according to general procedure A. Purification by LCC afforded compound **1s** as a light brown oil (2.10 g, 76%) that solidifies upon standing. R_f = 0.24 (heptane/AcOEt, 1:2); mp 119-120 °C; FT-IR (KBr) ν 3286 (N-H), 1750 (C=O), 1632 (C=O); ^1H NMR (300 MHz, $CDCl_3$) δ 8.29 (t, *J* = 6.1 Hz, 1H), 7.86 – 7.80 (m, 2H), 7.53 – 7.44 (m, 1H), 7.38 (tt, *J* = 6.6, 1.6 Hz, 2H), 5.59 (s, 2H), 4.90 (d, *J* = 6.1 Hz, 2H), 3.71 (s, 3H); ^{13}C {^1H} NMR (75 MHz, $CDCl_3$) δ 168.20 (C), 166.52 (C), 153.97 (C), 132.49 (CH), 128.58 (CH), 127.71 (C), 127.32 (CH), 53.41 (CH_3), 48.47 (CH_2), 32.12 (CH_2); HRMS (ESI/TOF) m/z: Not found.

*N-((1-Cyclohexyl-1H-tetrazol-5-yl)methyl)acrylamide **(2a)***

$TrtNH_2$ (2.0 mmol, 519 mg), $(CH_2O)_n$ (3.0 mmol, 90.1 mg), CyNC (2.2 mmol, 240 mg, 274 μL), $TMSN_3$ (2.2 mmol, 253 mg, 292 μL), TFA (4.0 mmol, 456 mg, 306 μL), acrylic acid (2.0 mmol, 144 mg, 137 μL), TEA (10 mmol, 1.01 g, 1.39 mL), HBTU (2.0 mmol, 758 mg) and DMAP (0.2 mmol, 24.4 mg) were reacted according to general procedure A. Purification by LCC afforded compound **2a** as a white yellowish solid (117 mg, 25%). R_f = 0.16 (heptane/AcOEt, 1:3); mp 128-130 °C; FT-IR (KBr) ν 3326 (N-H), 1661 (C=O); ^1H NMR (400 MHz, $CDCl_3$) δ 7.73 (t, *J* = 6.1 Hz, 1H), 6.41 – 6.22 (m, 2H), 5.72 (dd, *J* = 9.8, 1.7 Hz, 1H), 4.81 (d, *J* = 5.8 Hz, 2H), 4.55 (tt, *J* = 9.8, 4.8 Hz, 1H), 2.04 – 1.90 (m, 6H), 1.81 – 1.73 (m, 1H), 1.47 (dddd, *J* = 16.0, 12.6, 9.3, 3.3 Hz, 2H), 1.37 – 1.25 (m, 1H); ^{13}C {^1H} NMR (151 MHz, $CDCl_3$) δ 165.95 (C), 151.63 (C), 129.83 (CH), 127.92 (CH_2), 58.27 (CH), 32.90 (CH_2), 31.96 (CH_2), 25.12 (CH_2), 24.80 (CH_2); HRMS (ESI/TOF) m/z: $[M + H]^+$ Calcd for $C_{11}H_{18}N_5O$ 236.1506, Found 236.1516.

*N-((1-Cyclohexyl-1H-tetrazol-5-yl)methyl)methacrylamide **(2b)***

$TrtNH_2$ (2.0 mmol, 519 mg), $(CH_2O)_n$ (3.0 mmol, 90.1 mg), CyNC (2.2 mmol, 240 mg, 274 μL), $TMSN_3$ (2.2 mmol, 253 mg, 292 μL), TFA (4.0 mmol, 456 mg, 306 μL), methacrylic acid (2.0 mmol, 172 mg, 170 μL), TEA (10 mmol, 1.01 g, 1.39 mL), HBTU (2.0 mmol, 758 mg) and DMAP (0.2 mmol, 24.4 mg) were reacted according to general procedure A. Purification by LCC afforded compound **2b** as a white sticky solid (391 mg, 78%) that crystalizes upon standing. R_f = 0.18 (heptane/AcOEt, 1:2); mp 92-93 °C; FT-IR (KBr) ν 3307 (N-H), 1663 (C=O); ^1H NMR (400 MHz,

$CDCl_3$) δ 7.51 (s, 1H), 5.85 (s, 1H), 5.42 (s, 1H), 4.80 (s, 2H), 4.53 (s, 1H), 2.14 – 1.67 (m, 10H), 1.54 – 1.25 (m, 3H); ^{13}C {^{1}H} NMR (151 MHz, $CDCl_3$) δ 168.48 (C), 151.68 (C), 138.75 (C), 121.21 (CH_2), 58.04 (CH), 32.93 (CH_2), 32.23 (CH_2), 25.13 (CH_2), 24.82 (CH_2), 18.55 (CH_3); HRMS (ESI/TOF) m/z: $[M + H]^+$ Calcd for $C_{12}H_{20}N_5O$ 250.1662, Found 250.1665.

*(E)-N-((1-Cyclohexyl-1H-tetrazol-5-yl)methyl)-2-methylbut-2-enamide (**2c**)*

$TrtNH_2$ (4.0 mmol, 1.04 g), $(CH_2O)_n$ (6.0 mmol, 180 mg), CyNC (4.4 mmol, 480 mg, 548 μL), $TMSN_3$ (4.4 mmol, 506 mg, 584 μL), TFA (8.0 mmol, 912 mg, 612 μL), (E)-2-methylbut-2-enoic acid (4.0 mmol, 400 mg), TEA (20 mmol, 2.02 g, 2.78 mL), HBTU (4.0 mmol, 1.52 g) and DMAP (0.4 mmol, 48.8 mg) were reacted according to general procedure A. Purification by LCC afforded compound **2c** as a light yellow solid (842 mg, 80%). R_f = 0.22 (heptane/AcOEt, 1:2); mp 114-117 °C; FT-IR (KBr) ν 3310 (N-H), 1629 (C=O); ^{1}H NMR (400 MHz, $CDCl_3$) δ 7.11 (t, *J* = 5.8 Hz, 1H), 6.55 (q, *J* = 7.0 Hz, 1H), 4.79 (d, *J* = 5.8 Hz, 2H), 4.52 (tt, *J* = 9.9, 5.4 Hz, 1H), 2.03 – 1.90 (m, 6H), 1.86 (s, 3H), 1.81 – 1.71 (m, 4H (*J* = 7.2 Hz)), 1.47 (qt, *J* = 12.9, 3.8 Hz, 2H), 1.33 (tt, *J* = 12.7, 3.4 Hz, 1H); ^{13}C {^{1}H} NMR (151 MHz, $CDCl_3$) δ 169.36 (C), 151.74 (C), 132.61 (CH), 130.73 (C), 58.00 (CH), 32.92 (CH_2), 32.36 (CH_2), 25.14 (CH_2), 24.83 (CH_2), 14.04 (CH_3), 12.28 (CH_3); HRMS (ESI/TOF) m/z: $[M + H]^+$ Calcd for $C_{13}H_{22}N_5O$ 264.1819, Found 264.1818.

*N-((1-Cyclohexyl-1H-tetrazol-5-yl)methyl)furan-2-carboxamide (**2d**)*

$TrtNH_2$ (2.0 mmol, 519 mg), $(CH_2O)_n$ (3.0 mmol, 90.1 mg), CyNC (2.2 mmol, 240 mg, 274 μL), $TMSN_3$ (2.2 mmol, 253 mg, 292 μL), TFA (4.0 mmol, 456 mg, 306 μL), 2-furoic acid (2.0 mmol, 224 mg), TEA (10 mmol, 1.01 g, 1.39 mL), HBTU (2.0 mmol, 758 mg) and DMAP (0.2 mmol, 24.4 mg) were reacted according to general procedure A. Purification by LCC afforded compound **2d** as a white solid (377 mg, 69%). R_f = 0.18 (heptane/AcOEt, 1:2); mp 144-146 °C; FT-IR (KBr) ν 3299 (N-H), 1645 (C=O); ^{1}H NMR (400 MHz, $CDCl_3$) δ 7.70 (d, *J* = 6.0 Hz, 1H), 7.44 (d, *J* = 1.6 Hz, 1H), 7.15 (d, *J* = 3.5 Hz, 1H), 6.49 (dd, *J* = 3.6, 1.7 Hz, 1H), 4.92 (d, *J* = 5.9 Hz, 2H), 4.56 (tt, *J* = 10.2, 4.8 Hz, 1H), 2.08 – 1.88 (m, 6H), 1.76 (dt, *J* = 13.1, 3.4 Hz, 1H), 1.47 (qt, *J* = 12.9, 4.0 Hz, 2H), 1.33 (tt, *J* = 12.6, 3.4 Hz, 1H); ^{13}C {^{1}H} NMR (101 MHz, $CDCl_3$) δ 158.54 (C), 151.50 (C), 146.74 (C), 144.84 (CH), 115.24 (CH), 112.17 (CH), 58.29 (CH), 32.93 (CH_2), 31.76 (CH_2), 25.14 (CH_2), 24.81 (CH_2); HRMS (ESI/TOF) m/z: $[M + H]^+$ Calcd for $C_{13}H_{18}N_5O_2$ 276.1455, Found 276.1577.

*N-((1-Cyclohexyl-1H-tetrazol-5-yl)methyl)thiophene-2-carboxamide (**2e**)*

TrtNH$_2$ (2.0 mmol, 519 mg), $(CH_2O)_n$ (3.0 mmol, 90.1 mg), CyNC (2.2 mmol, 240 mg, 274 μL), TMSN$_3$ (2.2 mmol, 253 mg, 292 μL), TFA (4.0 mmol, 456 mg, 306 μL), 2-thiophenecarboxylic acid (2.0 mmol, 256 mg), TEA (10 mmol, 1.01 g, 1.39 mL), HBTU (2.0 mmol, 758 mg) and DMAP (0.2 mmol, 24.4 mg) were reacted according to general procedure A. Purification by LCC afforded compound **2e** as a white solid (463 mg, 79%). R_f = 0.16 (heptane/AcOEt, 1:1); mp 143-144 °C; FT-IR (KBr) ν 3310 (N-H), 1632 (C=O); ^{1}H NMR (400 MHz, $CDCl_3$) δ 8.35 (t, *J* = 5.9 Hz, 1H), 7.73 (d, *J* = 3.7 Hz, 1H), 7.48 (d, *J* = 5.3 Hz, 1H), 7.03 (dd, *J* = 5.0, 3.8 Hz, 1H), 4.92 (d, *J* = 5.9 Hz, 2H), 4.62 (tt, *J* = 11.2, 4.2 Hz, 1H), 2.09 – 1.85 (m, 7H), 1.75 (dt, *J* = 13.0, 3.4 Hz, 1H), 1.46 (qt, *J* = 13.0, 3.8 Hz, 2H), 1.31 (dtd, *J* = 18.7, 9.4, 8.1, 4.7 Hz, 1H); ^{13}C {^{1}H} NMR (151 MHz, $CDCl_3$) δ 162.31 (C), 151.91 (C), 137.70 (C), 130.97 (CH), 129.05 (CH), 127.94 (CH), 58.23 (CH), 32.97 (CH_2), 32.31 (CH_2), 25.12 (CH_2), 24.84 (CH_2); HRMS (ESI/TOF) m/z: $[M + H]^+$ Calcd for $C_{13}H_{18}N_5OS$ 292.1227, Found 292.1228.

*N-((1-Cyclohexyl-1H-tetrazol-5-yl)methyl)-1-methyl-1H-pyrrole-2-carboxamide (**2f**)*

TrtNH$_2$ (2.0 mmol, 519 mg), $(CH_2O)_n$ (3.0 mmol, 90.1 mg), CyNC (2.2 mmol, 240 mg, 274 μL), TMSN$_3$ (2.2 mmol, 253 mg, 292 μL), TFA (4.0 mmol, 456 mg, 306 μL), 1-methyl-1H-pyrrole-2-carboxylic acid (2.0 mmol, 250 mg), TEA (10 mmol, 1.01 g, 1.39 mL), HBTU (2.0 mmol, 758 mg) and DMAP (0.2 mmol, 24.4 mg) were reacted according to general procedure A. Purification by LCC afforded compound **2f** as a yellow solid (476 mg, 82%). R_f = 0.18 (heptane/AcOEt, 1:1); mp 143-147 °C; FT-IR (KBr) ν 3242 (N-H), 1657 (C=O); ^{1}H NMR (400 MHz, $CDCl_3$) δ 7.35 (t, *J* = 6.0 Hz, 1H), 6.77 (dd, *J* = 4.0, 1.7 Hz, 1H), 6.73 (dd, *J* = 2.2, 2.0 Hz, 1H), 6.07 (dd, *J* = 4.0, 2.6 Hz, 1H), 4.86 (d, *J* = 6.0 Hz, 2H), 4.65 – 4.52 (m, 1H), 3.92 (s, 3H), 2.06 – 1.87 (m, 6H), 1.75 (dt, *J* = 13.1, 3.4 Hz, 1H), 1.45 (qt, *J* = 12.5, 4.0 Hz, 2H), 1.32 (tt, *J* = 12.7, 3.3 Hz, 1H); ^{13}C {^{1}H} NMR (101 MHz, $CDCl_3$) δ 161.77 (C), 151.93 (C), 128.68 (CH), 124.38 (C), 113.20 (CH), 107.65 (CH), 58.04(CH), 36.77 (CH_3), 32.94 (CH_2), 31.67 (CH_2), 25.18 (CH_2), 24.84 (CH_2); HRMS (ESI/TOF) m/z: $[M + H]^+$ Calcd for $C_{14}H_{21}N_6O$ 289.1771, Found 289.1494.

*N-((1-Cyclohexyl-1H-tetrazol-5-yl)methyl)benzofuran-2-carboxamide (**2g**)*

TrtNH$_2$ (2.0 mmol, 519 mg), $(CH_2O)_n$ (3.0 mmol, 90.1 mg), CyNC (2.2 mmol, 240 mg, 274 μL), TMSN$_3$ (2.2 mmol, 253 mg, 292 μL), TFA (4.0 mmol, 456 mg, 306 μL), benzofuran-2-carboxylic acid (2.0 mmol, 324 mg), TEA (10 mmol, 1.01 g, 1.39 mL), HBTU (2.0 mmol, 758 mg) and DMAP (0.2 mmol, 24.4 mg) were reacted according to general procedure A. Purification by LCC

afforded compound **2g** as a white solid (443 mg, 68%). R_f = 0.26 (heptane/AcOEt, 1:1); mp 200-201 °C; FT-IR (KBr) ν 3326 (N-H), 1648 (C=O); ^{1}H NMR (400 MHz, DMSO-*d*6) δ 9.54 (t, *J* = 5.8 Hz, 1H), 7.79 (d, *J* = 7.8 Hz, 1H), 7.67 (d, *J* = 8.3 Hz, 1H), 7.63 (s, 1H), 7.49 (t, *J* = 7.7 Hz, 1H), 7.35 (t, *J* = 7.5 Hz, 1H), 4.85 (d, *J* = 5.8 Hz, 2H), 4.63 (tt, *J* = 11.5, 4.0 Hz, 1H), 2.09 – 1.96 (m, 2H), 1.82 (dd, *J* = 13.2, 8.0 Hz, 4H), 1.70 – 1.62 (m, 1H), 1.40 (dtd, *J* = 14.5, 11.1, 10.6, 3.5 Hz, 2H), 1.26 (tt, *J* = 12.7, 3.2 Hz, 1H); ^{13}C {^{1}H} NMR (151 MHz, DMSO-*d*6) δ 158.89 (C), 154.80 (C), 152.59 (C), 148.75 (C), 127.59 (CH), 127.46 (C), 124.27 (CH), 123.39 (CH), 112.28 (CH), 110.65 (CH), 57.17 (CH), 32.91 (CH_2), 32.41 (CH_2), 25.14 (CH_2), 25.00 (CH_2); HRMS (ESI/TOF) m/z: $[M + H]^+$ Calcd for $C_{17}H_{20}N_5O_2$ 326.1611, Found 326.1606.

*N-((1-Cyclohexyl-1H-tetrazol-5-yl)methyl)benzo[b]thiophene-2-carboxamide (**2h**)*

$TrtNH_2$ (2.0 mmol, 519 mg), $(CH_2O)_n$ (3.0 mmol, 90.1 mg), CyNC (2.2 mmol, 240 mg, 274 μL), $TMSN_3$ (2.2 mmol, 253 mg, 292 μL), TFA (4.0 mmol, 456 mg, 306 μL), thianaphthene-2-carboxylic acid (2.0 mmol, 356 mg), TEA (10 mmol, 1.01 g, 1.39 mL), HBTU (2.0 mmol, 758 mg) and DMAP (0.2 mmol, 24.4 mg) were reacted according to general procedure A. Purification by LCC afforded compound **2h** as a white solid (469 mg, 69%). R_f = 0.24 (heptane/AcOEt, 1:1); mp 162-164 °C; FT-IR (KBr) ν 3313 (N-H), 1638 (C=O); ^{1}H NMR (400 MHz, $CDCl_3$) δ 8.00 (t, *J* = 5.9 Hz, 1H), 7.90 – 7.82 (m, 2H), 7.54 – 7.46 (m, 1H), 7.45 – 7.36 (m, 2H), 4.94 (d, *J* = 5.8 Hz, 2H), 4.61 (tt, *J* = 11.0, 4.5 Hz, 1H), 2.06 – 1.88 (m, 6H), 1.76 (dt, *J* = 13.1, 3.3 Hz, 1H), 1.47 (qt, *J* = 12.8, 4.0 Hz, 2H), 1.32 (tt, *J* = 12.8, 3.4 Hz, 1H); ^{13}C {^{1}H} NMR (151 MHz, DMSO-*d*6) δ 162.33 (C), 152.67 (C), 140.79 (C), 139.50 (C), 139.08 (C), 126.93 (CH), 126.06 (CH), 125.84 (CH), 125.47 (CH), 123.30 (CH), 57.25 (CH), 32.94 (CH_2), 32.79 (CH_2), 25.17 (CH_2), 24.99 (CH_2); HRMS (ESI/TOF) m/z: $[M + H]^+$ Calcd for $C_{17}H_{20}N_5OS$ 342.1383, Found 342.1389.

*N-((1-Cyclohexyl-1H-tetrazol-5-yl)methyl)-1-methyl-1H-indole-3-carboxamide (**2i**)*

$TrtNH_2$ (2.0 mmol, 519 mg), $(CH_2O)_n$ (3.0 mmol, 90.1 mg), CyNC (2.2 mmol, 240 mg, 274 μL), $TMSN_3$ (2.2 mmol, 253 mg, 292 μL), TFA (4.0 mmol, 456 mg, 306 μL), 1-methylindole-3-carboxylic acid (2.0 mmol, 356 mg), TEA (10 mmol, 1.01 g, 1.39 mL), HBTU (2.0 mmol, 758 mg) and DMAP (0.2 mmol, 24.4 mg) were reacted according to general procedure A. Purification by LCC afforded compound **2i** as a white solid (330 mg, 49%). R_f = 0.14 (heptane/AcOEt, 1:2); mp 203-206 °C; FT-IR (KBr) ν 3282 (N-H), 1619 (C=O); ^{1}H NMR (400 MHz, $CDCl_3$) δ 8.66 (t, *J* = 5.8 Hz, 1H), 8.15 (d, *J* = 7.8 Hz, 1H), 8.02 (s, 1H), 7.49 (d, *J* = 8.1 Hz, 1H), 7.20 (dt, *J* = 24.9,

7.3 Hz, 2H), 4.83 (d, J = 5.7 Hz, 2H), 4.76 – 4.64 (m, 1H), 3.83 (s, 3H), 2.07 – 1.94 (m, 2H), 1.87 – 1.72 (m, 4H), 1.65 (d, J = 12.8 Hz, 1H), 1.46 – 1.31 (m, 2H), 1.24 (tt, J = 12.3, 3.3 Hz, 1H); ^{13}C {^{1}H} NMR (101 MHz, $CDCl_3$) δ 169.52 (C), 158.12 (C), 141.92 (C), 137.44 (CH), 131.59 (C), 127.31 (CH), 126.19 (CH), 126.06 (CH), 115.56 (CH), 113.83 (C), 61.88 (CH), 38.27 (CH_3), 37.72 (CH_2), 36.70 (CH_2), 29.93 (CH_2), 29.77 (CH_2); HRMS (ESI/TOF) m/z: $[M + H]^+$ Calcd for $C_{18}H_{23}N_6O$ 339.1928, Found 339.1922.

*N-((1-Cyclohexyl-1H-tetrazol-5-yl)methyl)isonicotinamide (**2j**)*

$TrtNH_2$ (2.0 mmol, 519 mg), $(CH_2O)_n$ (3.0 mmol, 90.1 mg), CyNC (2.2 mmol, 240 mg, 274 μL), $TMSN_3$ (2.2 mmol, 253 mg, 292 μL), TFA (4.0 mmol, 456 mg, 306 μL), isonicotinic acid (2.0 mmol, 246 mg), TEA (10 mmol, 1.01 g, 1.39 mL), HBTU (2.0 mmol, 758 mg) and DMAP (0.2 mmol, 24.4 mg) were reacted according to general procedure A. Purification by LCC afforded compound **2j** as a white solid (336 mg, 59%). R_f = 0.20 (AcOEt/MeOH, 20:1); mp 151-152 °C; FT-IR (KBr) ν 3254 (N-H), 1645 (C=O); ^{1}H NMR (400 MHz, DMSO-*d*6) δ 9.60 (t, J = 5.7 Hz, 1H), 8.76 (d, J = 5.1 Hz, 2H), 7.78 (d, J = 5.2 Hz, 1H), 4.87 (d, J = 5.6 Hz, 2H), 4.61 (tt, J = 11.3, 3.9 Hz, 1H), 2.05 – 1.96 (m, 2H), 1.80 (qd, J = 12.0, 11.3, 3.8 Hz, 4H), 1.71 – 1.63 (m, 1H), 1.38 (qd, J = 12.0, 10.4, 6.8 Hz, 2H), 1.31 – 1.21 (m, 1H); ^{13}C {^{1}H} NMR (151 MHz, DMSO-*d*6) δ 165.52 (C), 152.56 (C), 150.87 (CH), 140.74 (C), 121.63 (CH), 57.21 (CH), 32.91 (CH_2), 32.87 (CH_2), 25.14 (CH_2), 24.99 (CH_2); HRMS (ESI/TOF) m/z: $[M + H]^+$ Calcd for $C_{14}H_{19}N_6O$ 287.1615, Found 287.1613.

*N-((1-Cyclohexyl-1H-tetrazol-5-yl)methyl)thiazole-5-carboxamide (**2k**)*

$TrtNH_2$ (2.0 mmol, 519 mg), $(CH_2O)_n$ (3.0 mmol, 90.1 mg), CyNC (2.2 mmol, 240 mg, 274 μL), $TMSN_3$ (2.2 mmol, 253 mg, 292 μL), TFA (4.0 mmol, 456 mg, 306 μL), thiazole-5-carboxylic acid (2.0 mmol, 258 mg), TEA (10 mmol, 1.01 g, 1.39 mL), HBTU (2.0 mmol, 758 mg) and DMAP (0.2 mmol, 24.4 mg) were reacted according to general procedure A. Purification by LCC afforded compound **2k** as a light yellow solid (384 mg, 66%). R_f = 0.20 (heptane/AcOEt, 1:10); mp 159-162 °C; FT-IR (KBr) ν 3235 (N-H), 1632 (C=O); ^{1}H NMR (400 MHz, DMSO-*d*6) δ 9.53 (t, J = 5.7 Hz, 1H), 9.27 (s, 1H), 8.51 (s, 1H), 4.84 (d, J = 5.7 Hz, 2H), 4.58 (tt, J = 11.3, 3.9 Hz, 1H), 1.99 (dt, J = 10.2, 4.4 Hz, 2H), 1.81 (td, J = 10.0, 8.4, 3.6 Hz, 4H), 1.73 – 1.62 (m, 1H), 1.38 (qd, J = 13.3, 11.8, 6.7 Hz, 2H), 1.26 (tt, J = 13.0, 3.2 Hz, 1H); ^{13}C {^{1}H} NMR (151 MHz, DMSO-*d*6) δ 160.66 (C), 159.01 (CH), 152.57 (C), 144.68 (CH), 134.87 (C), 57.24 (CH), 32.91 (CH_2),

32.65 (CH_2), 25.14 (CH_2), 24.98 (CH_2); HRMS (ESI/TOF) m/z: $[M + H]^+$ Calcd for $C_{12}H_{17}N_6OS$ 293.1179, Found 293.1189.

*N-((1-Cyclohexyl-1H-tetrazol-5-yl)methyl)-1-naphthamide (**2l**)*

$TrtNH_2$ (2.0 mmol, 519 mg), $(CH_2O)_n$ (3.0 mmol, 90.1 mg), CyNC (2.2 mmol, 240 mg, 274 µL), $TMSN_3$ (2.2 mmol, 253 mg, 292 µL), TFA (4.0 mmol, 456 mg, 306 µL), 1-naphthoic acid (2.0 mmol, 344 mg), TEA (10 mmol, 1.01 g, 1.39 mL), HBTU (2.0 mmol, 758 mg) and DMAP (0.2 mmol, 24.4 mg) were reacted according to general procedure A. Purification by LCC afforded compound **2l** as a white solid (583 mg, 87%). R_f = 0.24 (heptane/AcOEt, 1:1); mp 249-250 °C; FT-IR (KBr) ν 3297 (N-H), 1646 (C=O); ^{1}H NMR (400 MHz, $CDCl_3$) δ 9.33 (t, *J* = 5.8 Hz, 1H), 8.30 – 8.18 (m, 1H), 8.03 (d, *J* = 8.2 Hz, 1H), 8.01 – 7.95 (m, 1H), 7.67 – 7.50 (m, 4H), 4.90 (d, *J* = 5.7 Hz, 2H), 4.68 (tt, *J* = 11.5, 4.0 Hz, 1H), 2.14 – 2.01 (m, 2H), 1.92 – 1.77 (m, 4H), 1.68 (d, *J* = 13.0 Hz, 1H), 1.43 (qt, *J* = 13.1, 2.7 Hz, 2H), 1.28 (tt, *J* = 13.0, 3.4 Hz, 1H); ^{13}C {^{1}H} NMR (151 MHz, DMSO-*d*6) δ 169.34 (C), 152.81 (C), 133.98 (C), 133.62 (C), 130.79 (CH), 130.17 (C), 128.74 (CH), 127.29 (CH), 126.80 (CH), 125.96 (CH), 125.69 (CH), 125.42 (CH), 57.19 (CH), 32.97 (CH_2), 32.78 (CH_2), 25.18 (CH_2), 25.03 (CH_2); HRMS (ESI/TOF) m/z: $[M + H]^+$ Calcd for $C_{19}H_{22}N_5O$ 336.1819, Found 336.1828.

Synthesis of tetrazole-isoquinolone/pyridone hybrids

*2-((1-Cyclohexyl-1H-tetrazol-5-yl)methyl)-3,4-diphenylisoquinolin-1(2H)-one (**4a**)*

Compound **1a** (0.25 mmol, 71.3 mg), diphenylacetylene (**3a**, 0.375 mmol, 66.8 mg), $Cu(OAc)_2$ (0.5 mmol, 90.8 mg), CsOAc (0.125 mmol, 24.0 mg) and $[RhCp^*Cl_2]_2$ (0.0125 mmol, 7.7 mg) were reacted according to general procedure B. Purification by LCC afforded compound **4a** as a white solid (104 mg, 90%). R_f = 0.26 (heptane/AcOEt, 2:1); mp 214-219 °C; FT-IR (KBr) ν 1651 (C=O), 1590 (C-H), 704 (C-H); ^{1}H NMR (400 MHz, $CDCl_3$) δ 8.46 (d, *J* = 7.8 Hz, 1H), 7.56 (t, *J* = 7.6 Hz, 1H), 7.49 (t, *J* = 7.5 Hz, 1H), 7.29 (dt, *J* = 8.6, 3.5 Hz, 2H), 7.24 – 7.05 (m, 9H), 5.22 (s, 2H), 4.19 (ddt, *J* = 10.9, 8.2, 4.4 Hz, 1H), 2.02 – 1.83 (m, 6H), 1.48 – 1.18 (m, 4H); ^{13}C {^{1}H} NMR (151 MHz, $CDCl_3$) δ 162.31 (C), 150.84 (C), 140.27 (C), 137.54 (C), 136.10 (C), 134.00 (C), 132.77 (CH), 131.41 (CH), 130.50 (CH), 128.70 (CH), 128.21 (CH), 127.99 (CH), 127.89 (CH), 127.01 (CH), 125.75 (CH), 124.63 (C), 120.12 (C), 58.03 (CH), 39.48 (CH_2), 32.58 (CH_2), 25.18 (CH_2), 24.84 (CH_2); HRMS (ESI/TOF) m/z: $[M + H]^+$ Calcd for $C_{29}H_{28}N_5O$ 462.2288, Found 462.2270.

2-((1-Cyclohexyl-1H-tetrazol-5-yl)methyl)-8-methyl-3,4-diphenylisoquinolin-1(2H)-one ***(4b)***

Compound **1b** (0.25 mmol, 74.8 mg), diphenylacetylene (**3a**, 0.375 mmol, 66.8 mg), $Cu(OAc)_2$ (0.5 mmol, 90.8 mg), CsOAc (0.125 mmol, 24.0 mg) and $[RhCp^*Cl_2]_2$ (0.0125 mmol, 7.7 mg) were reacted according to general procedure II during 24 h. Purification by LCC afforded compound **4b** as a light yellow solid (87 mg, 73%). R_f = 0.33 (heptane/AcOEt, 2:1); mp 192-193 °C; FT-IR (KBr) ν 1641 (C=O), 1595 (C-H), 696 (C-H); ^{1}H NMR (600 MHz, $CDCl_3$) δ 7.39 (t, *J* = 7.8 Hz, 1H), 7.29 – 7.26 (m, 1H), 7.26 – 7.23 (m, 2H), 7.22 – 7.15 (m, 6H), 7.13 – 7.08 (m, 2H), 7.00 (d, *J* = 8.1 Hz, 1H), 5.21 (s, 2H), 4.18 (tt, *J* = 9.7, 4.6 Hz, 1H), 2.95 (s, 3H), 1.95 – 1.85 (m, 6H), 1.73 (dd, *J* = 9.6, 6.5 Hz, 1H), 1.42 – 1.29 (m, 3H); ^{13}C {^{1}H} NMR (151 MHz, $CDCl_3$) δ 162.88 (C), 150.97 (C), 142.01 (C), 140.17 (C), 139.26 (C), 136.85 (C), 134.28 (C), 131.84 (CH), 131.51 (CH), 130.34 (CH), 130.23 (CH), 128.51 (CH), 128.14 (CH), 127.98 (CH), 126.86 (CH), 124.11 (CH), 123.30 (C), 120.02 (C), 57.93 (CH), 39.29 (CH_2), 32.56 (CH_2), 25.18 (CH_2), 24.84 (CH_2), 24.20 (CH_3); HRMS (ESI/TOF) m/z: $[M + H]^+$ Calcd for $C_{30}H_{30}N_5O$ 476.2445, Found 476.2427.

8-Bromo-2-((1-cyclohexyl-1H-tetrazol-5-yl)methyl)-3,4-diphenylisoquinolin-1(2H)-one ***(4c)***

Compound **1c** (0.25 mmol, 91.1 mg), diphenylacetylene (**3a**, 0.375 mmol, 66.8 mg), $Cu(OAc)_2$ (0.5 mmol, 90.8 mg), CsOAc (0.125 mmol, 24.0 mg) and $[RhCp^*Cl_2]_2$ (0.0125 mmol, 7.7 mg) were reacted according to general procedure B during 24 h. Purification by LCC afforded compound **4c** as a yellow solid (49 mg, 36%). R_f = 0.29 (heptane/AcOEt, 1:1); mp 195-197 °C; FT-IR (KBr) ν 1644 (C=O), 1599 (C-H), 697 (C-H);^{1}H NMR (400 MHz, $CDCl_3$) δ 8.47 (dd, *J* = 8.0, 1.4 Hz, 1H), 7.57 (td, *J* = 8.0, 7.6, 1.5 Hz, 1H), 7.53 – 7.47 (m, 1H), 7.29 (dd, *J* = 6.7, 2.9 Hz, 2H), 7.22 – 7.17 (m, 6H), 7.13 – 7.09 (m, 2H), 5.22 (s, 2H), 4.25 – 4.13 (m, 1H), 1.99 – 1.87 (m, 6H), 1.43 – 1.25 (m, 4H); ^{13}C {^{1}H} NMR (101 MHz, $CDCl_3$) δ 162.33 (C), 150.80 (C), 140.23 (C), 137.53 (C), 136.09 (C), 134.00 (C), 132.77 (CH), 131.41 (CH), 130.50 (CH), 128.69 (CH), 128.21 (CH), 127.98 (CH), 127.89 (CH), 127.03 (CH), 126.99 (CH), 125.74 (CH), 124.61 (C), 120.15 (C), 58.04 (CH), 39.45 (CH_2), 32.58 (CH_2), 25.19 (CH_2), 24.83 (CH_2); HRMS (ESI/TOF) m/z: $[M + H]^+$ Calcd for $C_{29}H_{27}BrN_5O$ 540.1394, Found 540.1405.

8-Chloro-2-((1-cyclohexyl-1H-tetrazol-5-yl)methyl)-3,4-diphenylisoquinolin-1(2H)-one ***(4d)***

Compound **1d** (0.25 mmol, 80.0 mg), diphenylacetylene (**3a**, 0.375 mmol, 66.8 mg), $Cu(OAc)_2$ (0.5 mmol, 90.8 mg), CsOAc (0.125 mmol, 24.0 mg) and $[RhCp^*Cl_2]_2$ (0.0125 mmol, 7.7 mg)

were reacted according to general procedure B during 24 h. Purification by LCC afforded compound **4d** as a yellow solid (46 mg, 37%). R_f = 0.26 (heptane/AcOEt, 1:1); mp 210-212 °C; FT-IR (KBr) ν 1646 (C=O), 1599 (C-H), 698 (C-H); ^{1}H NMR (400 MHz, $CDCl_3$) δ 8.47 (dd, *J* = 8.0, 1.4 Hz, 1H), 7.60 – 7.54 (m, 1H), 7.50 (t, *J* = 7.5 Hz, 1H), 7.29 (dd, *J* = 7.0, 2.8 Hz, 2H), 7.21 – 7.16 (m, 6H), 7.13 – 7.08 (m, 2H), 5.22 (s, 2H), 4.27 – 4.13 (m, 1H), 2.01 – 1.86 (m, 6H), 1.43 – 1.25 (m, 4H); ^{13}C {^{1}H} NMR (101 MHz, $CDCl_3$) δ 162.33 (C), 150.80 (C), 140.23 (C), 137.53 (C), 136.10 (C), 134.00 (C), 132.77 (CH), 131.41 (CH), 130.50 (CH), 128.69 (CH), 128.21 (CH), 127.98 (CH), 127.90 (CH), 127.03 (CH), 126.99 (CH), 125.74 (CH), 124.62 (C), 120.15 (C), 58.05 (CH), 39.45 (CH_2), 32.59 (CH_2), 25.20 (CH_2), 24.84 (CH_2); HRMS (ESI/TOF) m/z: Not found.

*2-((1-Cyclohexyl-1H-tetrazol-5-yl)methyl)-8-methoxy-3,4-diphenylisoquinolin-1(2H)-one (**4e**)*

Compound **1e** (0.25 mmol, 78.9 mg), diphenylacetylene (**3a**, 0.375 mmol, 66.8 mg), $Cu(OAc)_2$ (0.5 mmol, 90.8 mg), CsOAc (0.125 mmol, 24.0 mg) and $[RhCp^*Cl_2]_2$ (0.0125 mmol, 7.7 mg) were reacted according to general procedure B. The desired product could not be obtained.

*2-((1-Cyclohexyl-1H-tetrazol-5-yl)methyl)-6-methoxy-3,4-diphenylisoquinolin-1(2H)-one (**4f**)*

Compound **1f** (0.25 mmol, 78.9 mg), diphenylacetylene (**3a**, 0.375 mmol, 66.8 mg), $Cu(OAc)_2$ (0.5 mmol, 90.8 mg), CsOAc (0.125 mmol, 24.0 mg) and $[RhCp^*Cl_2]_2$ (0.0125 mmol, 7.7 mg) were reacted according to general procedure B. Purification by LCC afforded compound **4f** as a light yellow solid (107 mg, 87%). R_f = 0.18 (heptane/AcOEt, 2:1); mp 215-216 °C; FT-IR (KBr) ν 1648 (C=O), 1604 (C-H), 714 (C-H); ^{1}H NMR (400 MHz, $CDCl_3$) δ 8.39 (d, *J* = 8.9 Hz, 1H), 7.28 (dd, *J* = 6.5, 2.8 Hz, 2H), 7.23 – 7.13 (m, 6H), 7.12 – 7.09 (m, 2H), 7.06 (dd, *J* = 8.9, 2.5 Hz, 1H), 6.53 (d, *J* = 2.4 Hz, 1H), 5.20 (s, 2H), 4.25 – 4.11 (m, 1H), 3.69 (s, 3H), 2.00 – 1.83 (m, 6H), 1.72 (d, *J* = 12.0 Hz, 1H), 1.39 – 1.25 (m, 3H); ^{13}C {^{1}H} NMR (151 MHz, $CDCl_3$) δ 163.15 (C), 161.95 (C), 150.93 (C), 140.91 (C), 139.70 (C), 136.17 (C), 134.13 (C), 131.36 (CH), 130.42 (CH), 130.11 (CH), 128.64 (CH), 128.18 (CH), 128.01 (CH), 127.00 (CH), 119.83 (C), 118.54 (C), 115.78 (CH), 107.56 (CH), 58.00 (CH), 55.30 (CH_3), 39.27 (CH_2), 32.58 (CH_2), 25.19 (CH_2), 24.84 (CH_2); HRMS (ESI/TOF) m/z: $[M + H]^+$ Calcd for $C_{30}H_{30}N_5O_2$ 492.2394, Found 492.2377.

*2-((1-Cyclohexyl-1H-tetrazol-5-yl)methyl)-6-methyl-3,4-diphenylisoquinolin-1(2H)-one (**4g**)*

Compound **1g** (0.25 mmol, 74.8 mg), diphenylacetylene (**3a**, 0.375 mmol, 66.8 mg), $Cu(OAc)_2$ (0.5 mmol, 90.8 mg), CsOAc (0.125 mmol, 24.0 mg) and $[RhCp^*Cl_2]_2$ (0.0125 mmol, 7.7 mg)

were reacted according to general procedure B. Purification by LCC afforded compound **4g** as a light yellow solid (109 mg, 92%). R_f = 0.16 (heptane/AcOEt, 3:1); mp 212-214 °C; FT-IR (KBr) ν 1641 (C=O), 1603 (C-H), 699 (C-H); ^{1}H NMR (600 MHz, $CDCl_3$) δ 8.37 (d, *J* = 8.2 Hz, 1H), 7.34 (dd, *J* = 8.3, 1.7 Hz, 1H), 7.30 (dd, *J* = 7.2, 2.4 Hz, 2H), 7.24 – 7.16 (m, 6H), 7.15 – 7.10 (m, 2H), 6.96 (s, 1H), 5.23 (s, 2H), 4.22 (tt, *J* = 11.1, 4.1 Hz, 1H), 2.37 (s, 3H), 2.01 – 1.88 (m, 6H), 1.76 – 1.72 (m, 1H), 1.39 (qd, *J* = 13.4, 6.8 Hz, 2H), 1.34 – 1.28 (m, 1H); ^{13}C {^{1}H} NMR (151 MHz, $CDCl_3$) δ 162.28 (C), 150.88 (C), 143.50 (C), 140.31 (C), 137.62 (C), 136.24 (C), 134.13 (C), 131.45 (CH), 130.52 (CH), 128.64 (CH), 128.61 (CH), 128.17 (CH), 127.95 (CH), 127.91 (CH), 126.92 (CH), 125.41 (CH), 122.45 (C), 119.99 (C), 58.01 (CH), 39.35 (CH_2), 32.59 (CH_2), 25.20 (CH_2), 24.85 (CH_2), 22.04 (CH_3); HRMS (ESI/TOF) m/z: $[M + H]^+$ Calcd for $C_{30}H_{30}N_5O$ 476.2445, Found 476.2425.

*6-Chloro-2-((1-cyclohexyl-1H-tetrazol-5-yl)methyl)-3,4-diphenylisoquinolin-1(2H)-one (**4h**)*

Compound **1h** (0.25 mmol, 79.8 mg), diphenylacetylene (**3a**, 0.375 mmol, 66.8 mg), $Cu(OAc)_2$ (0.5 mmol, 90.8 mg), CsOAc (0.125 mmol, 24.0 mg) and $[RhCp^*Cl_2]_2$ (0.0125 mmol, 7.7 mg) were reacted according to general procedure B. Purification by LCC afforded compound **4h** as a yellow solid (117 mg, 94%). R_f = 0.20 (heptane/AcOEt, 3:1); mp 241-243 °C; FT-IR (KBr) ν 1656 (C=O), 1598 (C-H), 709 (C-H); ^{1}H NMR (400 MHz, $CDCl_3$) δ 8.39 (d, *J* = 8.6 Hz, 1H), 7.44 (dd, *J* = 8.7, 2.0 Hz, 1H), 7.28 (dd, *J* = 7.3, 2.4 Hz, 2H), 7.25 – 7.12 (m, 7H), 7.11 – 7.05 (m, 2H), 5.19 (s, 2H), 4.25 – 4.09 (m, 1H), 2.01 – 1.82 (m, 6H), 1.75 – 1.67 (m, 1H), 1.46 – 1.27 (m, 3H); ^{13}C {^{1}H} NMR (101 MHz, $CDCl_3$) δ 161.71 (C), 150.61 (C), 141.73 (C), 139.52 (C), 138.89 (C), 135.35 (C), 133.69 (C), 131.29 (CH), 130.29 (CH), 129.74 (CH), 128.86 (CH), 128.28 (CH), 128.20 (CH), 127.56 (CH), 127.31 (CH), 125.08 (CH), 122.96 (C), 119.24 (C), 58.08 (CH), 39.52 (CH_2), 32.56 (CH_2), 25.17 (CH_2), 24.81 (CH_2); HRMS (ESI/TOF) m/z: $[M + H]^+$ Calcd for $C_{29}H_{27}ClN_5O$ 496.1899, Found 496.1877.

*2-((1-Cyclohexyl-1H-tetrazol-5-yl)methyl)-3,4-diphenyl-6-(trifluoromethyl)isoquinolin-1(2H)-one (**4i**)*

Compound **1i** (0.25 mmol, 88.3 mg), diphenylacetylene (**3a**, 0.375 mmol, 66.8 mg), $Cu(OAc)_2$ (0.5 mmol, 90.8 mg), CsOAc (0.125 mmol, 24.0 mg) and $[RhCp^*Cl_2]_2$ (0.0125 mmol, 7.7 mg) were reacted according to general procedure B. Purification by LCC afforded compound **4i** as a light yellow solid (118 mg, 89%). R_f = 0.20 (heptane/AcOEt, 3:1); mp 165-169 °C; FT-IR (KBr)

ν 1650 (C=O), 693 (C-H);^{1}H NMR (400 MHz, $CDCl_3$) δ 8.58 (d, *J* = 8.4 Hz, 1H), 7.70 (d, *J* = 8.4 Hz, 1H), 7.45 (s, 1H), 7.33 – 7.27 (m, 2H), 7.26 – 7.16 (m, 6H), 7.13 – 7.07 (m, 2H), 5.21 (s, 2H), 4.23 – 4.12 (m, 1H), 2.00 – 1.84 (m, 6H), 1.77 – 1.69 (m, 1H), 1.45 – 1.28 (m, 3H); ^{13}C {^{1}H} NMR (101 MHz, $CDCl_3$) δ 161.53 (C), 150.48 (C), 141.90 (C), 137.67 (C), 135.03 (C), 134.36 (q, *J* = 32.6 Hz, CF_3), 133.53 (C), 131.25 (CH), 130.30 (CH), 129.07 (CH), 128.98 (CH), 128.35 (C), 128.30 (CH), 127.50 (CH), 126.69 (C), 122.96 (q, *J* = 3.8 Hz, CH), 119.83 (C), 58.15 (CH), 39.69 (CH_2), 32.57 (CH_2), 25.19 (CH_2), 24.81 (CH_2); ^{19}F {^{13}C} NMR (376 MHz, $CDCl_3$) δ -63.04; HRMS (ESI/TOF) m/z: $[M + H]^+$ Calcd for $C_{30}H_{27}F_3N_5O$ 530.2162, Found 530.2139.

*2-((1-Cyclohexyl-1H-tetrazol-5-yl)methyl)-7-methyl-3,4-diphenylisoquinolin-1(2H)-one **(4j)***

Compound **1j** (0.50 mmol, 150 mg), diphenylacetylene (**3a**, 0.750 mmol, 134 mg), $Cu(OAc)_2$ (1.0 mmol, 182 mg), CsOAc (0.250 mmol, 48.0 mg) and $[RhCp^*Cl_2]_2$ (0.0250 mmol, 15.4 mg) were reacted according to general procedure B. Purification by LCC afforded compound **4j** as a light yellow solid (219 mg, 92%). R_f = 0.30 (heptane/AcOEt, 2:1); mp 229-231 °C; FT-IR (KBr) ν 1643 (C=O), 728 (C-H); ^{1}H NMR (400 MHz, $CDCl_3$) δ 8.26 (d, *J* = 1.9 Hz, 1H), 7.38 (dd, *J* = 8.3, 1.9 Hz, 1H), 7.30 (dd, *J* = 6.6, 2.8 Hz, 2H), 7.17 (td, *J* = 8.8, 7.6, 5.4 Hz, 6H), 7.12 – 7.06 (m, 3H), 5.21 (s, 2H), 4.21 (tt, *J* = 11.2, 4.4 Hz, 1H), 2.47 (s, 3H), 2.02 – 1.85 (m, 6H), 1.73 (d, *J* = 12.0 Hz, 1H), 1.41 – 1.25 (m, 3H); ^{13}C {^{1}H} NMR (101 MHz, $CDCl_3$) δ 162.30 (C), 150.87 (C), 139.27 (C), 137.20 (C), 136.27 (C), 135.24 (C), 134.23 (CH), 134.10 (C), 131.39 (CH), 130.62 (CH), 128.61 (CH), 128.17 (CH), 127.92 (CH), 127.44 (CH), 126.91 (CH), 125.72 (CH), 124.50 (C), 120.10 (C), 58.02 (CH), 39.45 (CH_2), 32.59 (CH_2), 25.20 (CH_2), 24.85 (CH_2), 21.32 (CH_3); HRMS (ESI/TOF) m/z: $[M + H]^+$ Calcd for $C_{30}H_{30}N_5O$ 476.2445, Found 476.2430.

*7-Chloro-2-((1-cyclohexyl-1H-tetrazol-5-yl)methyl)-3,4-diphenylisoquinolin-1(2H)-one **(4k)***

Compound **1k** (0.50 mmol, 160 mg), diphenylacetylene (**3a**, 0.750 mmol, 134 mg), $Cu(OAc)_2$ (1.0 mmol, 182 mg), CsOAc (0.250 mmol, 48.0 mg) and $[RhCp^*Cl_2]_2$ (0.0250 mmol, 15.4 mg) were reacted according to general procedure B. Purification by LCC afforded compound **4k** as a light yellow solid (175 mg, 71%). R_f = 0.32 (heptane/AcOEt, 2:1); mp 107-116 °C; FT-IR (KBr) ν 1648 (C=O), 1596 (C-H), 699 (C-H); ^{1}H NMR (600 MHz, $CDCl_3$) δ 8.45 (d, J = 2.3 Hz, 1H), 7.51 (dd, J = 8.8, 2.3 Hz, 1H), 7.34 – 7.29 (m, 2H), 7.25 – 7.18 (m, 6H), 7.15 (d, J = 8.7 Hz, 1H), 7.13 – 7.08 (m, 2H), 5.21 (s, 2H), 4.21 (ddd, J = 11.3, 7.1, 4.3 Hz, 1H), 2.02 – 1.87 (m, 6H), 1.75 (d, J = 12.8 Hz, 1H), 1.45 – 1.35 (m, 2H), 1.32 (tt, J = 13.0, 2.9 Hz, 1H); ^{13}C {^{1}H} NMR (151 MHz,

$CDCl_3$) δ 161.30 (C), 150.58 (C), 140.60 (C), 135.95 (C), 135.65 (C), 133.69 (C), 133.16 (CH), 131.30 (CH), 130.43 (CH), 128.85 (CH), 128.29 (CH), 128.13 (CH), 127.51 (CH), 127.30 (CH), 127.23 (CH), 125.71 (C), 119.64 (C), 58.12 (CH), 39.60 (CH_2), 32.59 (CH_2), 25.21 (CH_2), 24.83 (CH_2); HRMS (ESI/TOF) m/z: $[M + H]^+$ Calcd for $C_{29}H_{27}ClN_5O$ 496.1899, Found 496.1877.

*7-Bromo-2-((1-cyclohexyl-1H-tetrazol-5-yl)methyl)-3,4-diphenylisoquinolin-1(2H)-one (**4l**)*

Compound **1l** (0.50 mmol, 182 mg), diphenylacetylene (**3a**, 0.750 mmol, 134 mg), $Cu(OAc)_2$ (1.0 mmol, 182 mg), CsOAc (0.250 mmol, 48.0 mg) and $[RhCp^*Cl_2]_2$ (0.0250 mmol, 15.4 mg) were reacted according to general procedure B. Purification by LCC afforded compound **4l** as a light yellow solid (367 mg, 68%). R_f = 0.40 (heptane/AcOEt, 2:1); mp 207-210 °C; FT-IR (KBr) ν 1646 (C=O), 1590 (C-H), 714 (C-H); ^1H NMR (400 MHz, $CDCl_3$) δ 8.60 (d, J = 2.1 Hz, 1H), 7.63 (dd, J = 8.7, 2.2 Hz, 1H), 7.29 (dd, J = 7.3, 2.4 Hz, 2H), 7.23 – 7.15 (m, 6H), 7.08 (dd, J = 8.8, 2.8 Hz, 3H), 5.19 (s, 2H), 4.18 (dd, J = 9.9, 5.5 Hz, 1H), 1.99 – 1.85 (m, 6H), 1.78 – 1.70 (m, 1H), 1.43 – 1.26 (m, 3H); $^{13}C\{^1H\}$ NMR (101 MHz, $CDCl_3$) δ 161.16 (C), 150.56 (C), 140.78 (C), 136.27 (C), 135.88 (CH), 135.58 (C), 133.69 (C), 131.29 (CH), 130.43 (CH), 130.38 (CH), 128.85 (CH), 128.28 (CH), 128.12 (CH), 127.61 (CH), 127.22 (CH), 125.94 (C), 121.05 (C), 119.68 (C), 58.11 (CH), 39.60 (CH_2), 32.58 (CH_2), 25.20 (CH_2), 24.82 (CH_2); HRMS (ESI/TOF) m/z: $[M + H]^+$ Calcd for $C_{29}H_{27}BrN_5O$ 540.1394, Found 540.1381.

*2-((1-Cyclohexyl-1H-tetrazol-5-yl)methyl)-7-iodo-3,4-diphenylisoquinolin-1(2H)-one (**4m**)*

Compound **1m** (0.50 mmol, 206 mg), diphenylacetylene (**3a**, 0.750 mmol, 134 mg), $Cu(OAc)_2$ (1.0 mmol, 182 mg), CsOAc (0.250 mmol, 48.0 mg) and $[RhCp^*Cl_2]_2$ (0.0250 mmol, 15.4 mg) were reacted according to general procedure B. Purification by LCC afforded compound **4m** as a light yellow solid (214 mg, 73%). R_f = 0.36 (heptane/AcOEt, 2:1); mp 198-204 °C; FT-IR (KBr) ν 1651 (C=O), 1589 (C-H), 698 (C-H); ^1H NMR (400 MHz, $CDCl_3$) δ 8.80 (d, J = 1.7 Hz, 1H), 7.82 (dd, J = 8.6, 1.8 Hz, 1H), 7.33 – 7.27 (m, 2H), 7.23 – 7.13 (m, 6H), 7.10 – 7.05 (m, 2H), 6.91 (d, J = 8.5 Hz, 1H), 5.18 (s, 2H), 4.18 (dd, J = 9.9, 5.6 Hz, 1H), 2.02 – 1.85 (m, 6H), 1.74 (d, J = 11.2 Hz, 1H), 1.44 – 1.27 (m, 3H); $^{13}C\{^1H\}$ NMR (101 MHz, $CDCl_3$) δ 160.94 (C), 150.57 (C), 141.42 (CH), 141.02 (C), 136.65 (CH), 135.52 (C), 133.71 (C), 131.28 (CH), 130.36 (CH), 128.85 (CH), 128.27 (CH), 128.11 (CH), 127.52 (CH), 127.21 (CH), 126.03 (C), 119.73 (C), 92.04 (C), 58.11 (CH), 39.60 (CH_2), 32.58 (CH_2), 25.20 (CH_2), 24.82 (CH_2); HRMS (ESI/TOF) m/z: $[M + H]^+$ Calcd for $C_{29}H_{27}IN_5O$ 588.1257, Found 588.1232.

2-(1-(1-Cyclohexyl-1H-tetrazol-5-yl)-2-methylpropyl)-3,4-diphenylisoquinolin-1(2H)-one ***(4n)***

Compound **1n** (0.25 mmol, 81.9 mg), diphenylacetylene (**3a**, 0.375 mmol, 66.8 mg), $Cu(OAc)_2$ (0.5 mmol, 90.8 mg), CsOAc (0.125 mmol, 24.0 mg) and $[RhCp^*Cl_2]_2$ (0.0125 mmol, 7.7 mg) were reacted according to general procedure B. The desired product could not be obtained.

2-(1-(1-Cyclohexyl-1H-tetrazol-5-yl)-2-phenylethyl)-3,4-diphenylisoquinolin-1(2H)-one ***(4o)***

Compound **1o** (0.25 mmol, 93.9 mg), diphenylacetylene (**3a**, 0.375 mmol, 66.8 mg), $Cu(OAc)_2$ (0.5 mmol, 90.8 mg), CsOAc (0.125 mmol, 24.0 mg) and $[RhCp^*Cl_2]_2$ (0.0125 mmol, 7.7 mg) were reacted according to general procedure B. The desired product could not be obtained.

2-((1-Benzyl-1H-tetrazol-5-yl)methyl)-3,4-diphenylisoquinolin-1(2H)-one ***(4p)***

Compound **1p** (0.25 mmol, 73.3 mg), diphenylacetylene (**3a**, 0.375 mmol, 66.8 mg), $Cu(OAc)_2$ (0.5 mmol, 90.8 mg), CsOAc (0.125 mmol, 24.0 mg) and $[RhCp^*Cl_2]_2$ (0.0125 mmol, 7.7 mg) were reacted according to general procedure B. Purification by LCC afforded compound **4p** as a light yellow solid (87 mg, 74%). R_f = 0.18 (heptane/AcOEt, 2:1); mp 199-201 °C; FT-IR (KBr) ν 1637 (C=O), 1594 (C-H), 698 (C-H); ^{1}H NMR (400 MHz, $CDCl_3$) δ 8.46 (dd, *J* = 7.9, 1.5 Hz, 1H), 7.53 (dtd, *J* = 22.5, 7.3, 1.4 Hz, 2H), 7.25 (ddt, *J* = 10.0, 5.4, 2.7 Hz, 5H), 7.21 – 7.12 (m, 7H), 7.12 – 7.08 (m, 2H), 7.07 – 7.02 (m, 2H), 5.67 (s, 2H), 5.05 (s, 2H); ^{13}C {^{1}H} NMR (101 MHz, $CDCl_3$) δ 162.40 (C), 151.76 (C), 140.14 (C), 137.49 (C), 136.00 (C), 133.71 (C), 133.49 (C), 132.82 (CH), 131.33 (CH), 130.52 (CH), 129.14 (CH), 128.75 (CH), 128.72 (CH), 128.17 (CH), 127.96 (CH), 127.76 (CH), 127.37 (CH), 127.08 (CH), 126.99 (CH), 125.76 (CH), 124.53 (C), 120.24 (C), 51.03 (CH_2), 39.04 (CH_2); HRMS (ESI/TOF) m/z: $[M + H]^+$ Calcd for $C_{30}H_{24}N_5O$ 470.1975, Found 470.1955.

2-((1-(4-Methoxyphenyl)-1H-tetrazol-5-yl)methyl)-3,4-diphenylisoquinolin-1(2H)-one ***(4q)***

Compound **1q** (0.25 mmol, 77.3 mg), diphenylacetylene (**3a**, 0.375 mmol, 66.8 mg), $Cu(OAc)_2$ (0.5 mmol, 90.8 mg), CsOAc (0.125 mmol, 24.0 mg) and $[RhCp^*Cl_2]_2$ (0.0125 mmol, 7.7 mg) were reacted according to general procedure B. Purification by LCC afforded compound **4q** as a yellow solid (80 mg, 66%). R_f = 0.16 (heptane/AcOEt, 2:1); mp 227 °C; FT-IR (KBr) ν 1641 (C=O), 1595 (C-H), 697 (C-H); ^{1}H NMR (400 MHz, $CDCl_3$) δ 8.45 (dd, *J* = 7.9, 1.4 Hz, 1H), 7.60 – 7.47 (m, 2H), 7.35 – 7.29 (m, 2H), 7.27 – 7.23 (m, 2H), 7.18 (td, *J* = 7.4, 5.2 Hz, 7H), 7.13 – 7.08 (m, 2H), 7.00 – 6.95 (m, 2H), 5.19 (s, 2H), 3.84 (s, 3H); ^{13}C {^{1}H} NMR (101 MHz, $CDCl_3$)

δ 162.32 (C), 161.01 (C), 152.29 (C), 140.12 (C), 137.52 (C), 136.06 (C), 134.02 (C), 132.75 (CH), 131.39 (CH), 130.29 (CH), 128.71 (CH), 128.25 (CH), 127.99 (CH), 127.88 (CH), 127.01 (CH), 126.59 (CH), 125.91 (C), 125.72(CH), 124.62 (C), 120.10 (C), 114.83 (CH), 55.65 (CH_3), 40.14 (CH_2); HRMS (ESI/TOF) m/z: Not found.

*2-((1-(tert-Butyl)-1H-tetrazol-5-yl)methyl)-3,4-diphenylisoquinolin-1(2H)-one (**4r**)*

Compound **1r** (0.25 mmol, 64.8 mg), diphenylacetylene (**3a,** 0.375 mmol, 66.8 mg), $Cu(OAc)_2$ (0.5 mmol, 90.8 mg), CsOAc (0.125 mmol, 24.0 mg) and $[RhCp^*Cl_2]_2$ (0.0125 mmol, 7.7 mg) were reacted according to general procedure B. Purification by LCC afforded compound **4r** as a yellow solid (50 mg, 46%). R_f = 0.33 (heptane/AcOEt, 1:1); mp 269-272 °C; FT-IR (KBr) ν 1650 (C=O), 1601 (C-H), 699 (C-H); ^{1}H NMR (400 MHz, $CDCl_3$) δ 8.51 (dd, *J* = 8.0, 1.5 Hz, 1H), 7.57 (ddd, *J* = 8.3, 7.1, 1.6 Hz, 1H), 7.51 (td, *J* = 7.6, 7.1, 1.3 Hz, 1H), 7.23 – 7.07 (m, 11H), 5.38 (s, 2H), 1.52 (s, 9H); ^{13}C {^{1}H} NMR (101 MHz, $CDCl_3$) δ 162.38(C), 151.31 (C), 140.07 (C), 137.64 (C), 136.12 (C), 134.18 (C), 132.70 (CH), 131.43 (CH), 130.19 (CH), 128.59 (CH), 128.16 (CH), 128.08 (CH), 127.95 (CH), 126.96 (CH), 126.94 (CH), 125.70 (CH), 124.74 (C), 119.97 (C), 60.67 (C), 41.75 (CH_2), 29.24 (CH_3); HRMS (ESI/TOF) m/z: $[M + H]^+$ Calcd for $C_{27}H_{26}N_5O$ 436.2132, Found 436.2121.

*Methyl 2-(5-((1-oxo-3,4-diphenylisoquinolin-2(1H)-yl)methyl)-1H-tetrazol-1-yl)acetatE (**4s**)*

Compound **1s** (0.25 mmol, 68.8 mg), diphenylacetylene (**3a**, 0.375 mmol, 66.8 mg), $Cu(OAc)_2$ (0.5 mmol, 90.8 mg), CsOAc (0.125 mmol, 24.0 mg) and $[RhCp^*Cl_2]_2$ (0.0125 mmol, 7.7 mg) were reacted according to general procedure B. Purification by LCC afforded compound **4s** as a yellow solid (15 mg, 13%). R_f = 0.27 (heptane/AcOEt, 1:1); mp 206-208 °C; FT-IR (KBr) ν 1753 (C=O), 1646 (C=O), 1599 (C-H), 696 (C-H); ^{1}H NMR (400 MHz, $CDCl_3$) δ 8.39 (dd, *J* = 7.9, 1.5 Hz, 1H), 7.56 (dd, *J* = 8.0, 1.5 Hz, 1H), 7.53 – 7.47 (m, 1H), 7.41 (dd, *J* = 6.6, 2.9 Hz, 2H), 7.25 (d, *J* = 2.1 Hz, 2H), 7.24 – 7.14 (m, 5H), 7.13 – 7.08 (m, 2H), 5.58 (s, 2H), 5.19 (s, 2H), 3.65 (s, 3H); ^{13}C {^{1}H} NMR (101 MHz, $CDCl_3$) δ 166.58 (C), 162.51 (C), 152.62 (C), 140.48 (C), 137.56 (C), 136.01 (C), 133.55 (C), 132.94 (CH), 131.35 (CH), 130.84 (CH), 128.94 (CH), 128.32 (CH), 128.02 (CH), 127.56 (CH), 127.13 (CH), 127.07 (CH), 125.85 (CH), 124.26 (C), 120.56 (C), 53.06 (CH_3), 48.34 (CH_2), 38.89 (CH_2); HRMS (ESI/TOF) m/z: Not found.

*3,4-Bis(4-bromophenyl)-2-((1-cyclohexyl-1H-tetrazol-5-yl)methyl)isoquinolin-1(2H)-one (**4t**)*

Compound **1a** (0.25 mmol, 71.3 mg), 1,2-bis(4-bromophenyl)ethyne (**3b**, 0.375 mmol, 126 mg), $Cu(OAc)_2$ (0.5 mmol, 90.8 mg), CsOAc (0.125 mmol, 24.0 mg) and $[RhCp^*Cl_2]_2$ (0.0125 mmol, 7.7 mg) were reacted according to general procedure B. Purification by LCC afforded compound **4t** as a yellow solid (154 mg, 99%). R_f = 0.14 (heptane/AcOEt, 3:1); mp 234-235 °C; FT-IR (KBr) ν 1651 (C=O), 1600 (C-H); ^{1}H NMR (600 MHz, $CDCl_3$) δ 8.46 (dd, *J* = 8.0, 1.5 Hz, 1H), 7.63 – 7.58 (m, 1H), 7.56 – 7.52 (m, 1H), 7.43 – 7.37 (m, 4H), 7.28 – 7.23 (m, 2H), 7.14 (d, *J* = 8.2 Hz, 1H), 7.01 (d, *J* = 8.3 Hz, 2H), 5.16 (s, 2H), 4.31 (tt, *J* = 11.3, 4.0 Hz, 1H), 2.07 – 2.02 (m, 2H), 2.00 – 1.92 (m, 4H), 1.77 (d, *J* = 13.2 Hz, 1H), 1.49 – 1.39 (m, 2H), 1.37 – 1.31 (m, 1H); ^{13}C {^{1}H} NMR (151 MHz, $CDCl_3$) δ 162.13 (C), 150.60 (C), 139.22 (C), 136.98 (C), 134.84 (C), 133.04 (CH), 132.97 (CH), 132.61 (C), 132.12 (CH), 131.75 (CH), 131.54 (CH), 128.00 (CH), 127.45 (CH), 125.48 (CH), 124.66 (C), 123.55 (C), 121.59 (C), 119.11 (C), 58.24 (CH), 39.37 (CH_2), 32.68 (CH_2), 25.24 (CH_2), 24.85 (CH_2); HRMS (ESI/TOF) m/z: $[M + H]^+$ Calcd for $C_{29}H_{26}Br_2N_5O$ 618.0500, Found 618.0478.

*2-((1-Cyclohexyl-1H-tetrazol-5-yl)methyl)-3,4-bis(4-methoxyphenyl)isoquinolin-1(2H)-one (**4u**)*

Compound **1a** (0.25 mmol, 71.3 mg), 1,2-bis(4-methoxyphenyl)ethyne (**3c**, 0.375 mmol, 79.9 mg), $Cu(OAc)_2$ (0.5 mmol, 90.8 mg), CsOAc (0.125 mmol, 24.0 mg) and $[RhCp^*Cl_2]_2$ (0.0125 mmol, 7.7 mg) were reacted according to general procedure B. Purification by LCC afforded compound **4u** as a yellow solid (68 mg, 58%). R_f = 0.14 (heptane/AcOEt, 2:1); mp 181-184 °C; FT-IR (KBr) ν 1649 (C=O), 1607 (C-H); ^{1}H NMR (400 MHz, $CDCl_3$) δ 8.44 (dd, *J* = 8.1, 1.4 Hz, 1H), 7.59 – 7.52 (m, 1H), 7.47 (td, *J* = 7.6, 7.2, 1.2 Hz, 1H), 7.24 – 7.16 (m, 3H), 7.04 – 6.98 (m, 2H), 6.78 – 6.73 (m, 2H), 6.73 – 6.67 (m, 2H), 5.21 (s, 2H), 4.22 (tt, *J* = 11.4, 4.4 Hz, 1H), 3.76 (s, 3H), 3.72 (s, 3H), 2.02 – 1.87 (m, 6H), 1.73 (d, *J* = 12.2 Hz, 1H), 1.46 – 1.29 (m, 3H); ^{13}C {^{1}H} NMR (101 MHz, $CDCl_3$) δ 162.41 (C), 159.43 (C), 158.27 (C), 150.94 (C), 140.34 (C), 137.92 (C), 132.66 (CH), 132.41 (CH), 132.21 (CH), 131.74 (CH), 128.50 (C), 127.81 (CH), 126.85 (CH), 126.44 (C), 125.74 (CH), 124.58 (C), 120.06 (C), 115.71 (CH), 113.60 (CH), 113.47 (CH), 58.03 (CH), 55.43 (CH_3), 55.11 (CH_3), 39.40 (CH_2), 32.63 (CH_2), 25.20 (CH_2), 24.85 (CH_2); HRMS (ESI/TOF) m/z: $[M + H]^+$ Calcd for $C_{31}H_{32}N_5O_3$ 522.2500, Found 522.2475.

*2-((1-Cyclohexyl-1H-tetrazol-5-yl)methyl)-3,4-di(thiophen-2-yl)isoquinolin-1(2H)-one (**4v**)*

Compound **1a** (0.25 mmol, 71.3 mg), 1,2-di(thiophen-2-yl)ethyne **3d** (0.375 mmol, 79.9 mg), $Cu(OAc)_2$ (0.5 mmol, 90.8 mg), CsOAc (0.125 mmol, 24.0 mg) and $[RhCp^*Cl_2]_2$ (0.0125 mmol,

7.7 mg) were reacted according to general procedure B. Purification by LCC afforded compound **4v** as a brown solid (72 mg, 85%). R_f = 0.24 (heptane/AcOEt, 2:1); mp 248-253 °C; FT-IR (KBr) ν 1655 (C=O), 1601 (C-H); ^{1}H NMR (400 MHz, $CDCl_3$) δ 8.43 (d, *J* = 7.9 Hz, 1H), 7.68 – 7.59 (m, 1H), 7.53 (t, *J* = 7.5 Hz, 1H), 7.41 (d, *J* = 8.1 Hz, 1H), 7.29 (dd, *J* = 10.5, 5.2 Hz, 2H), 7.17 (d, *J* = 3.5 Hz, 1H), 6.94 (dd, *J* = 5.1, 3.6 Hz, 1H), 6.89 (q, *J* = 3.1, 2.6 Hz, 2H), 5.31 (s, 2H), 4.37 – 4.19 (m, 1H), 2.14 – 1.84 (m, 6H), 1.49 – 1.21 (m, 4H); ^{13}C {^{1}H} NMR (101 MHz, $CDCl_3$) δ 162.19 (C), 150.78 (C), 137.40 (C), 136.40 (C), 135.16 (C), 133.55 (C), 133.09 (CH), 131.68 (CH), 129.98 (CH), 128.43 (CH), 127.85 (CH), 127.79 (CH), 126.94 (CH), 126.74 (CH), 126.55 (CH), 125.90 (CH), 124.81 (C), 115.81 (C), 58.16 (CH), 39.63 (CH_2), 32.65 (CH_2), 25.23 (CH_2), 24.84 (CH_2); HRMS (ESI/TOF) m/z: $[M + H]^+$ Calcd for $C_{25}H_{24}N_5OS_2$ 474.1417, Found 474.1394.

*1-((1-Cyclohexyl-1H-tetrazol-5-yl)methyl)-5,6-diphenylpyridin-2(1H)-one (**5a**)*

Compound **2a** (0.25 mmol, 55.8 mg), diphenylacetylene (**3a**, 0.375 mmol, 66.8 mg), $Cu(OAc)_2$ (0.5 mmol, 90.8 mg), CsOAc (0.125 mmol, 24.0 mg) and $[RhCp^*Cl_2]_2$ (0.0125 mmol, 7.7 mg) were reacted according to general procedure B. Purification by LCC afforded compound **5a** as a light yellow solid (52 mg, 50%). R_f = 0.16 (heptane/AcOEt, 1:1); mp 230-235 °C; FT-IR (KBr) ν 1666 (C=O), 694 (C-H); ^{1}H NMR (400 MHz, $CDCl_3$) δ 7.52 (d, *J* = 9.4 Hz, 1H), 7.43 – 7.36 (m, 2H), 7.28 (d, *J* = 8.2 Hz, 3H), 7.13 (d, *J* = 6.4 Hz, 3H), 6.99 (dd, *J* = 7.2, 2.3 Hz, 2H), 6.67 (d, *J* = 9.3 Hz, 1H), 5.14 (s, 2H), 4.32 (ddd, *J* = 11.2, 8.8, 4.4 Hz, 1H), 2.03 – 1.88 (m, 7H), 1.74 (d, *J* = 12.7 Hz, 1H), 1.42 – 1.30 (m, 3H); ^{13}C {^{1}H} NMR (151 MHz, $CDCl_3$) δ 162.13 (C), 150.39 (C), 146.46 (C), 143.01 (CH), 137.96 (C), 133.24 (C), 130.37 (CH), 129.49 (CH), 129.35 (CH), 128.60 (CH), 128.00, 126.74 (CH), 121.61 (C), 119.15 (CH), 58.15 (CH), 39.52 (CH_2), 32.69 (CH_2), 29.70 (CH_2), 25.25 (CH_2), 24.86 (CH_2); HRMS (ESI/TOF) m/z: $[M + H]^+$ Calcd for $C_{25}H_{26}N_5O$ 412.2132, Found 412.2115.

*1-((1-Cyclohexyl-1H-tetrazol-5-yl)methyl)-3-methyl-5,6-diphenylpyridin-2(1H)-one (**5b**)*

Compound **2b** (0.25 mmol, 62.3 mg), diphenylacetylene (**3a**, 0.375 mmol, 66.8 mg), $Cu(OAc)_2$ (0.5 mmol, 90.8 mg), CsOAc (0.125 mmol, 24.0 mg) and $[RhCp^*Cl_2]_2$ (0.0125 mmol, 7.7 mg) were reacted according to general procedure B. Purification by LCC afforded compound **5b** as a light yellow solid (94 mg, 89%). R_f = 0.14 (heptane/AcOEt, 2:1); mp 122-126 °C; FT-IR (KBr) ν 1637 (C=O), 701 (C-H); ^{1}H NMR (400 MHz, $CDCl_3$) δ 7.41 (s, 1H), 7.39 – 7.33 (m, 2H), 7.30 – 7.21 (m, 3H), 7.15 – 7.06 (m, 3H), 6.99 (dd, *J* = 7.5, 2.0 Hz, 2H), 5.16 (s, 2H), 4.26 (tt, *J* = 11.7,

4.3 Hz, 1H), 2.20 (s, 3H), 2.04 – 1.89 (m, 6H), 1.44 – 1.24 (m, 4H). ^{13}C {^{1}H} NMR (101 MHz, $CDCl_3$) δ 162.43 (C), 150.70 (C), 143.64 (C), 140.38 (CH), 138.29 (C), 133.52 (C), 130.49 (CH), 129.51 (CH), 129.14 (CH), 128.51 (CH), 128.11 (C), 127.89 (CH), 126.54 (CH), 121.10 (C), 58.05 (CH), 39.88 (CH_2), 32.59 (CH_2), 25.18 (CH_2), 24.84 (CH_2), 17.11 (CH_3); HRMS (ESI/TOF) m/z: $[M + H]^+$ Calcd for $C_{26}H_{28}N_5O$ 426.2288, Found 426.2273.

*1-((1-Cyclohexyl-1H-tetrazol-5-yl)methyl)-3,4-dimethyl-5,6-diphenylpyridin-2(1H)-one (**5c**)*

Compound **2c** (0.25 mmol, 65.9 mg), diphenylacetylene (**3a**, 0.375 mmol, 66.8 mg), $Cu(OAc)_2$ (0.5 mmol, 90.8 mg), CsOAc (0.125 mmol, 24.0 mg) and $[RhCp^*Cl_2]_2$ (0.0125 mmol, 7.7 mg) were reacted according to general procedure B. Purification by LCC afforded compound **5c** as a light yellow solid (55 mg, 50%). R_f = 0.14 (heptane/AcOEt, 2:1); mp 175-177 °C; FT-IR (KBr) ν 1644 (C=O), 695 (C-H); ^{1}H NMR (600 MHz, $CDCl_3$) δ 7.28 – 7.22 (m, 2H), 7.19 – 7.12 (m, 5H), 7.10 (d, J = 7.2 Hz, 1H), 6.98 (d, J = 7.4 Hz, 2H), 5.12 (s, 2H), 4.24 (tt, J = 11.3, 4.1 Hz, 1H), 2.21 (s, 3H), 2.02 – 1.97 (m, 2H), 1.94 (s, 3H), 1.93 – 1.89 (m, 3H), 1.74 (d, J = 13.5 Hz, 1H), 1.44 – 1.25 (m, 4H); ^{13}C {^{1}H} NMR (151 MHz, $CDCl_3$) δ 161.94 (C), 150.81 (C), 146.78 (C), 142.85 (C), 137.64 (C), 133.93 (C), 130.83 (CH), 130.11 (CH), 128.61 (CH), 128.13 (CH), 127.83 (CH), 126.68 (CH), 125.12 (C), 123.25 (C), 58.00 (CH), 39.99 (CH_2), 32.57 (CH_2), 25.18 (CH_2), 24.85 (CH_2), 18.46 (CH_3), 13.40 (CH_3); HRMS (ESI/TOF) m/z: $[M + H]^+$ Calcd for $C_{27}H_{30}N_5O$ 440.2445, Found 440.2429.

*6-((1-Cyclohexyl-1H-tetrazol-5-yl)methyl)-4,5-diphenylfuro[2,3-c]pyridin-7(6H)-one (**5d**)*

Compound **2d** (0.25 mmol, 68.8 mg), diphenylacetylene (**3a**, 0.375 mmol, 66.8 mg), $Cu(OAc)_2$ (0.5 mmol, 90.8 mg), CsOAc (0.125 mmol, 24.0 mg) and $[RhCp^*Cl_2]_2$ (0.0125 mmol, 7.7 mg) were reacted according to general procedure B during 24 h. Purification by LCC afforded compound **5d** as a light yellow solid (39 mg, 35%). R_f = 0.22 (heptane/AcOEt, 1:1); mp 207-210 °C; FT-IR (KBr) ν 1685 (C=O), 703 (C-H); ^{1}H NMR (400 MHz, $CDCl_3$) δ 7.75 (d, J = 1.9 Hz, 1H), 7.38 – 7.33 (m, 2H), 7.28 – 7.22 (m, 3H), 7.17 (d, J = 7.4 Hz, 3H), 7.11 – 7.06 (m, 2H), 6.50 (d, J = 1.9 Hz, 1H), 5.26 (s, 2H), 4.27 (ddd, J = 11.2, 6.9, 4.3 Hz, 1H), 2.02 – 1.86 (m, 6H), 1.78 – 1.68 (m, 1H), 1.46 – 1.27 (m, 3H); ^{13}C {^{1}H} NMR (151 MHz, $CDCl_3$) δ 152.98 (C), 150.60 (C), 148.72 (CH), 141.78 (C), 140.99 (C), 135.68 (C), 134.63 (C), 133.58 (C), 130.95 (CH), 130.13 (CH), 129.08 (CH), 128.42 (CH), 128.05 (CH), 127.05 (CH), 116.24 (C), 107.68 (CH), 58.14 (CH), 39.15 (CH_2), 32.65(CH_2), 25.23 (CH_2), 24.86 (CH_2); HRMS (ESI/TOF) m/z: Not found.

*6-((1-Cyclohexyl-1H-tetrazol-5-yl)methyl)-4,5-diphenylthieno[2,3-c]pyridin-7(6H)-one (**5e**)*

Compound **2e** (0.25 mmol, 72.8 mg), diphenylacetylene (**3a**, 0.375 mmol, 66.8 mg), $Cu(OAc)_2$ (0.5 mmol, 90.8 mg), CsOAc (0.125 mmol, 24.0 mg) and $[RhCp^*Cl_2]_2$ (0.0125 mmol, 7.7 mg) were reacted according to general procedure B. Purification by LCC afforded compound **5e** as a light yellow solid (112 mg, 96%). R_f = 0.14 (heptane/AcOEt, 2:1); mp 206-208 °C; ^{1}H NMR (400 MHz, $CDCl_3$) δ 7.66 (d, *J* = 5.2 Hz, 1H), 7.34 (dd, *J* = 7.4, 2.1 Hz, 2H), 7.26 – 7.14 (m, 6H), 7.14 – 7.08 (m, 2H), 6.92 (d, *J* = 5.2 Hz, 1H), 5.25 (s, 2H), 4.29 – 4.17 (m, 1H), 2.04 – 1.84 (m, 6H), 1.73 (d, *J* = 12.1 Hz, 1H), 1.41 – 1.25 (m, 3H); ^{13}C {^{1}H} NMR (101 MHz, $CDCl_3$) δ 158.23 (C), 150.70 (C), 146.52 (C), 141.68 (C), 136.48 (C), 133.65 (C), 133.60 (CH), 130.71 (CH), 130.56 (CH), 128.94 (CH), 128.38 (C), 128.32 (CH), 127.98 (CH), 127.06 (CH), 125.09 (CH), 118.89 (C), 58.10 (CH), 39.36 (CH_2), 32.61 (CH_2), 25.20 (CH_2), 24.84 (CH_2); HRMS (ESI/TOF) m/z: [M + H]$^+$ Calcd for $C_{27}H_{26}N_5OS$ 468.1852, Found 468.1847.

*6-((1-Cyclohexyl-1H-tetrazol-5-yl)methyl)-1-methyl-4,5-diphenyl-1,6-dihydro-7H-pyrrolo[2,3-c]pyridin-7-one (**5f**)*

Compound **2f** (0.25 mmol, 72.1 mg), diphenylacetylene (**3a**, 0.375 mmol, 66.8 mg), $Cu(OAc)_2$ (0.5 mmol, 90.8 mg), CsOAc (0.125 mmol, 24.0 mg) and $[RhCp^*Cl_2]_2$ (0.0125 mmol, 7.7 mg) were reacted according to general procedure B. Purification by LCC afforded compound **5f** as a light yellow solid (110 mg, 95%). R_f = 0.22 (heptane/AcOEt, 3:1); mp 223-225 °C; FT-IR (KBr) ν 1654 (C=O), 703 (C-H); ^{1}H NMR (400 MHz, $CDCl_3$) δ 7.25 – 7.04 (m, 10H), 7.00 (d, *J* = 2.8 Hz, 1H), 6.04 (d, *J* = 2.8 Hz, 1H), 5.26 (s, 2H), 4.18 (s, 3H), 4.03 (dq, *J* = 9.7, 6.3, 5.2 Hz, 1H), 1.97 – 1.78 (m, 6H), 1.70 (d, *J* = 9.2 Hz, 1H), 1.29 (qt, *J* = 11.2, 7.8, 6.6 Hz, 3H); ^{13}C {^{1}H} NMR (151 MHz, $CDCl_3$) δ 155.52 (C), 151.29 (C), 136.81 (C), 136.42 (C), 134.54 (C), 132.76 (C), 131.95 (CH), 131.08 (CH), 130.41 (CH), 128.46 (CH), 128.16 (CH), 127.73 (CH), 126.50 (CH), 121.56 (C), 117.60 (C), 102.63 (CH), 57.87 (CH), 38.72 (CH_2), 35.97 (CH_3), 32.50 (CH_2), 25.16 (CH_2), 24.82 (CH_2); HRMS (ESI/TOF) m/z: [M + H]$^+$ Calcd for $C_{28}H_{29}N_6O$ 465.2397, Found 465.2385.

*2-((1-Cyclohexyl-1H-tetrazol-5-yl)methyl)-3,4-diphenylbenzofuro[2,3-c]pyridin-1(2H)-one (**5g**)*

Compound **2g** (0.25 mmol, 81.3 mg), diphenylacetylene (**3a**, 0.375 mmol, 66.8 mg), $Cu(OAc)_2$ (0.5 mmol, 90.8 mg), CsOAc (0.125 mmol, 24.0 mg) and $[RhCp^*Cl_2]_2$ (0.0125 mmol, 7.7 mg) were reacted according to general procedure B during 18 h. Purification by LCC afforded

compound **5g** as a white solid (26 mg, 21%). R_f = 0.12 (heptane/AcOEt, 2:1); mp 263-265 °C; FT-IR (KBr) ν 1676 (C=O), 706 (C-H); ^{1}H NMR (400 MHz, $CDCl_3$) δ 7.65 (d, *J* = 8.4 Hz, 1H), 7.47 (t, *J* = 7.9 Hz, 1H), 7.40 (dd, *J* = 6.8, 2.8 Hz, 2H), 7.28 – 7.19 (m, 8H), 7.09 (t, *J* = 7.6 Hz, 1H), 6.77 (d, *J* = 7.9 Hz, 1H), 5.30 (s, 2H), 4.35 (ddt, *J* = 11.9, 9.1, 4.3 Hz, 1H), 2.06 – 1.89 (m, 8H), 1.74 (d, *J* = 12.6 Hz, 1H), 1.44 – 1.29 (m, 3H); ^{13}C {^{1}H} NMR (101 MHz, $CDCl_3$) δ 157.23 (C), 153.83 (C), 150.38 (C), 142.45 (C), 141.59 (C), 135.33 (C), 133.16 (C), 130.82 (CH), 130.56 (CH), 129.36 (C), 129.10 (CH), 128.91 (CH), 128.36 (CH), 128.25 (CH), 127.65 (CH), 123.45 (CH), 123.21 (C), 123.15 (CH), 116.93 (C), 112.68 (CH), 58.21 (CH), 39.44 (CH_2), 32.70 (CH_2), 25.26 (CH_2), 24.87 (CH_2); HRMS (ESI/TOF) m/z: $[M + H]^+$ Calcd for $C_{31}H_{28}N_5O_2$ 502.2237, Found 502.2219.

*2-((1-Cyclohexyl-1H-tetrazol-5-yl)methyl)-3,4-diphenylbenzo[4,5]thieno[2,3-c]pyridin-1(2H)-one (**5h**)*

Compound **2h** (0.25 mmol, 85.4 mg), diphenylacetylene (**3a**, 0.375 mmol, 66.8 mg), $Cu(OAc)_2$ (0.5 mmol, 90.8 mg), CsOAc (0.125 mmol, 24.0 mg) and $[RhCp^*Cl_2]_2$ (0.0125 mmol, 7.7 mg) were reacted according to general procedure B. Purification by LCC afforded compound **5h** as a yellow solid (103 mg, 80%). R_f = 0.28 (heptane/AcOEt, 2:1); mp 246-248 °C; FT-IR (KBr) ν 1642 (C=O), 715 (C-H); ^{1}H NMR (600 MHz, $CDCl_3$) δ 7.92 (d, *J* = 8.1 Hz, 1H), 7.42 (t, *J* = 7.6 Hz, 1H), 7.35 (dd, *J* = 6.6, 2.7 Hz, 2H), 7.30 – 7.26 (m, 3H), 7.25 – 7.18 (m, 5H), 7.07 (t, *J* = 7.8 Hz, 1H), 6.60 (d, *J* = 8.4 Hz, 1H), 5.30 (s, 2H), 4.28 (tt, *J* = 11.7, 4.2 Hz, 1H), 2.04 – 1.99 (m, 2H), 1.97 – 1.89 (m, 4H), 1.75 (d, *J* = 13.1 Hz, 1H), 1.41 (qt, *J* = 13.4, 2.9 Hz, 2H), 1.34 – 1.27 (m, 1H); ^{13}C {^{1}H} NMR (151 MHz, $CDCl_3$) δ 158.47 (C), 150.46 (C), 142.86 (C), 142.76 (C), 140.63 (C), 136.31 (C), 135.58 (C), 133.34 (C), 131.05 (CH), 130.58 (CH), 129.03 (C), 128.91 (CH), 128.40 (CH), 128.24 (CH), 127.69 (CH), 127.56 (CH), 125.95 (CH), 124.39 (CH), 123.25 (CH), 119.38 (C), 58.14 (CH), 39.59 (CH_2), 32.63 (CH_2), 25.20 (CH_2), 24.85 (CH_2); HRMS (ESI/TOF) m/z: $[M + H]^+$ Calcd for $C_{31}H_{28}N_5OS$ 518.2009, Found 518.1994.

*2-((1-Cyclohexyl-1H-tetrazol-5-yl)methyl)-5-methyl-3,4-diphenyl-2,5-dihydro-1H-pyrido[4,3-b]indol-1-one (**5i**)*

Compound **2i** (0.25 mmol, 84.6 mg), diphenylacetylene (**3a**, 0.375 mmol, 66.8 mg), $Cu(OAc)_2$ (0.5 mmol, 90.8 mg), CsOAc (0.125 mmol, 24.0 mg) and $[RhCp^*Cl_2]_2$ (0.0125 mmol, 7.7 mg) were reacted according to general procedure B. Purification by LCC afforded compound **5i** as a

brown solid (20 mg, 16%). R_f = 0.18 (heptane/AcOEt, 2:1); mp 272-274 °C; FT-IR (KBr) ν 1645 (C=O), 708 (C-H); ^{1}H NMR (400 MHz, $CDCl_3$) δ 8.44 (d, *J* = 7.7 Hz, 1H), 7.45 – 7.26 (m, 5H), 7.19 (d, *J* = 9.4 Hz, 8H), 5.31 (s, 2H), 4.27 (t, *J* = 4.3 Hz, 1H), 3.15 (s, 3H), 2.00 – 1.84 (m, 6H), 1.45 – 1.22 (m, 4H); ^{13}C {^{1}H} NMR (151 MHz, $CDCl_3$) δ 159.12 (C), 151.13 (C), 144.23 (C), 143.24 (C), 140.02 (C), 135.36 (C), 133.99 (C), 131.84 (CH), 130.39 (CH), 128.68 (CH), 128.09 (CH), 128.07 (CH), 127.63 (CH), 124.52 (CH), 124.03 (C), 121.89 (CH), 121.81 (CH), 111.70 (C), 108.90 (CH), 106.86 (C), 57.99 (CH), 38.82 (CH_2), 32.61 (CH_2), 31.85 (CH_3), 25.19 (CH_2), 24.87 (CH_2); HRMS (ESI/TOF) m/z: $[M + H]^+$ Calcd for $C_{32}H_{31}N_6O$ 515.2554, Found 515.2540.

*2-((1-Cyclohexyl-1H-tetrazol-5-yl)methyl)-3,4-diphenyl-2,6-naphthyridin-1(2H)-one (**5j**)*

Compound **2j** (0.25 mmol, 71.6 mg), diphenylacetylene (**3a**, 0.375 mmol, 66.8 mg), $Cu(OAc)_2$ (0.5 mmol, 90.8 mg), CsOAc (0.125 mmol, 24.0 mg) and $[RhCp^*Cl_2]_2$ (0.0125 mmol, 7.7 mg) were reacted according to general procedure B. Purification by LCC afforded compound **5j** as a white solid (57 mg, 49%). R_f = 0.35 (heptane/AcOEt, 0:1); mp 210-212 °C; FT-IR (KBr) ν 1666 (C=O), 697 (C-H); ^{1}H NMR (600 MHz, $CDCl_3$) δ 8.95 – 8.45 (m, 2H), 8.21 (d, *J* = 5.1 Hz, 1H), 7.36 – 7.29 (m, 2H), 7.22 (dp, *J* = 8.6, 5.5, 3.6 Hz, 6H), 7.14 (d, *J* = 7.3 Hz, 2H), 5.22 (s, 2H), 4.17 (ddt, *J* = 11.0, 8.1, 4.0 Hz, 1H), 1.97 – 1.89 (m, 6H), 1.74 (d, *J* = 12.9 Hz, 1H), 1.40 – 1.26 (m, 3H); ^{13}C {^{1}H} NMR (151 MHz, $CDCl_3$) δ 161.25 (C), 150.33 (C), 149.50 (CH), 146.58 (CH), 142.09 (C), 134.25 (C), 133.20 (C), 131.23 (CH), 130.36 (CH), 129.23 (C), 129.09 (CH), 128.39 (CH), 128.25 (CH), 127.52 (CH), 119.63 (CH), 118.40 (C), 58.17 (CH), 39.73 (CH_2), 32.55 (CH_2), 25.17 (CH_2), 24.79 (CH_2); HRMS (ESI/TOF) m/z: $[M + H]^+$ Calcd for $C_{28}H_{27}N_6O$ 463.2241, Found 463.2236.

*5-((1-Cyclohexyl-1H-tetrazol-5-yl)methyl)-6,7-diphenylthiazolo[5,4-c]pyridin-4(5H)-one (**5k**)*

Compound **2k** (0.25 mmol, 73.1 mg), diphenylacetylene (**3a**, 0.375 mmol, 66.8 mg), $Cu(OAc)_2$ (0.5 mmol, 90.8 mg), CsOAc (0.125 mmol, 24.0 mg) and $[RhCp^*Cl_2]_2$ (0.0125 mmol, 7.7 mg) were reacted according to general procedure B during 18 h. The desired product could not be obtained.

*2-((1-Cyclohexyl-1H-tetrazol-5-yl)methyl)-3,4-diphenylbenzo[h]isoquinolin-1(2H)-one (**5l**)*

Compound **2l** (0.25 mmol, 78.9 mg), diphenylacetylene (**3a**, 0.375 mmol, 66.8 mg), $Cu(OAc)_2$ (0.5 mmol, 90.8 mg), CsOAc (0.125 mmol, 24.0 mg) and $[RhCp^*Cl_2]_2$ (0.0125 mmol, 7.7 mg)

were reacted according to general procedure B. Purification by LCC afforded compound **5l** as a light yellow solid (94 mg, 73%). R_f = 0.14 (heptane/AcOEt, 5:1); mp 203-205 °C; FT-IR (KBr) ν 1640 (C=O), 704 (C-H); ^{1}H NMR (600 MHz, $CDCl_3$) δ 10.16 (d, *J* = 8.8 Hz, 1H), 7.94 (d, *J* = 8.9 Hz, 1H), 7.90 (dd, *J* = 8.0, 1.4 Hz, 1H), 7.76 (ddd, *J* = 8.6, 6.9, 1.5 Hz, 1H), 7.63 (ddd, *J* = 8.0, 6.9, 1.2 Hz, 1H), 7.38 (dd, *J* = 6.6, 2.9 Hz, 2H), 7.28 – 7.20 (m, 7H), 7.19 – 7.15 (m, 2H), 5.35 (s, 2H), 4.38 (tt, *J* = 11.4, 3.9 Hz, 1H), 2.10 – 2.04 (m, 2H), 2.02 – 1.94 (m, 4H), 1.81 – 1.74 (m, 1H), 1.50 – 1.40 (m, 2H), 1.35 (tt, *J* = 12.7, 3.1 Hz, 1H); ^{13}C {^{1}H} NMR (151 MHz, $CDCl_3$) δ 162.29 (C), 150.93 (C), 142.25 (C), 139.04 (C), 136.75 (C), 134.24 (C), 134.04 (CH), 132.26 (C), 131.80 (C), 131.69 (CH), 130.33 (CH), 128.73 (CH), 128.61 (CH), 128.27 (CH), 128.23 (CH), 128.10 (CH), 127.44 (CH), 127.06 (CH), 126.57 (CH), 123.55 (CH), 120.32 (C), 118.33 (C), 58.10 (CH), 40.03 (CH_2), 32.70 (CH_2), 25.26 (CH_2), 24.91 (CH_2); HRMS (ESI/TOF) m/z: $[M + H]^+$ Calcd for $C_{33}H_{30}N_5O$ 512.2445, Found 512.2422.

IR and NMR spectra of *N*-acyl aminomethyl tetrazoles

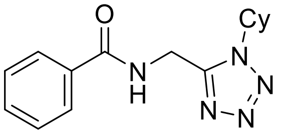

1a

N-((1-cyclohexyl-1*H*-tetrazol-5-yl)methyl)benzamide

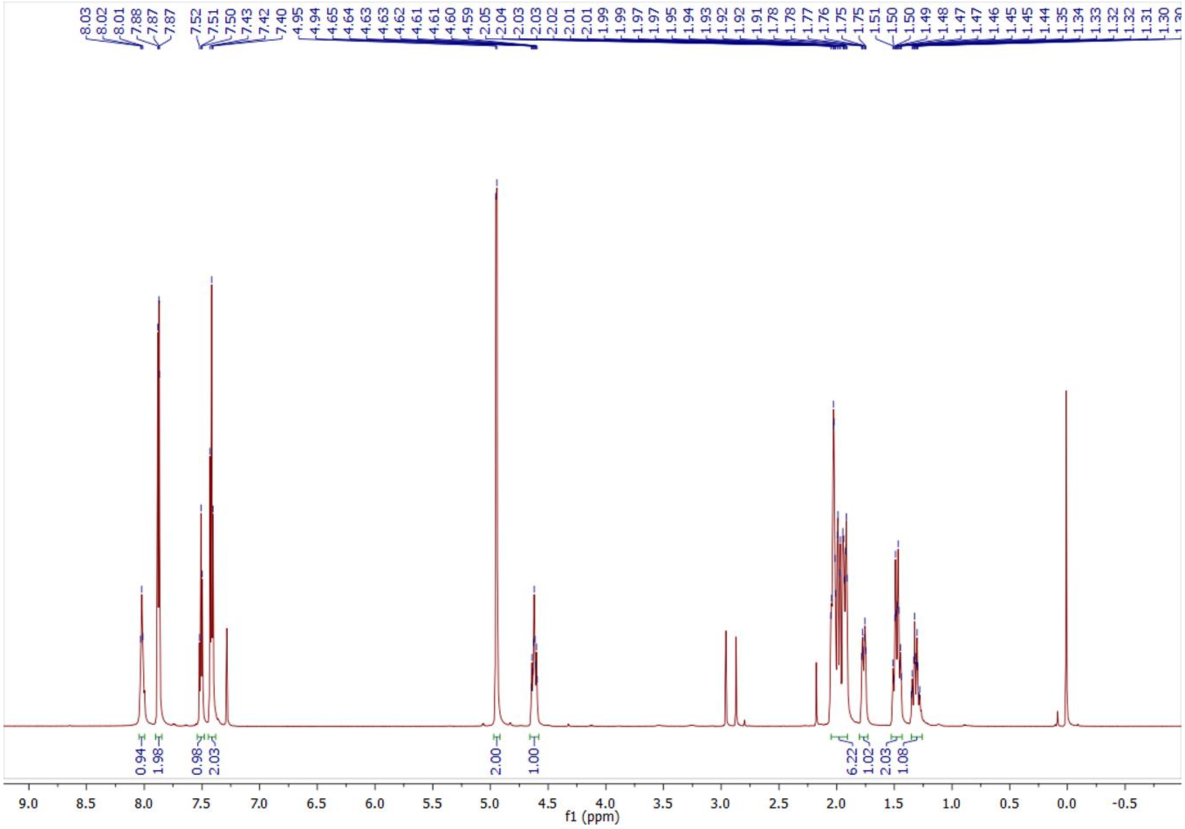

Figure S1. ^{1}H NMR (600 MHz, $CDCl_3$) spectrum of compound **1a**.

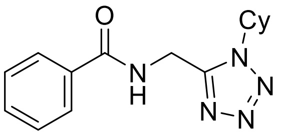

1a

N-((1-cyclohexyl-1*H*-tetrazol-5-yl)methyl)benzamide

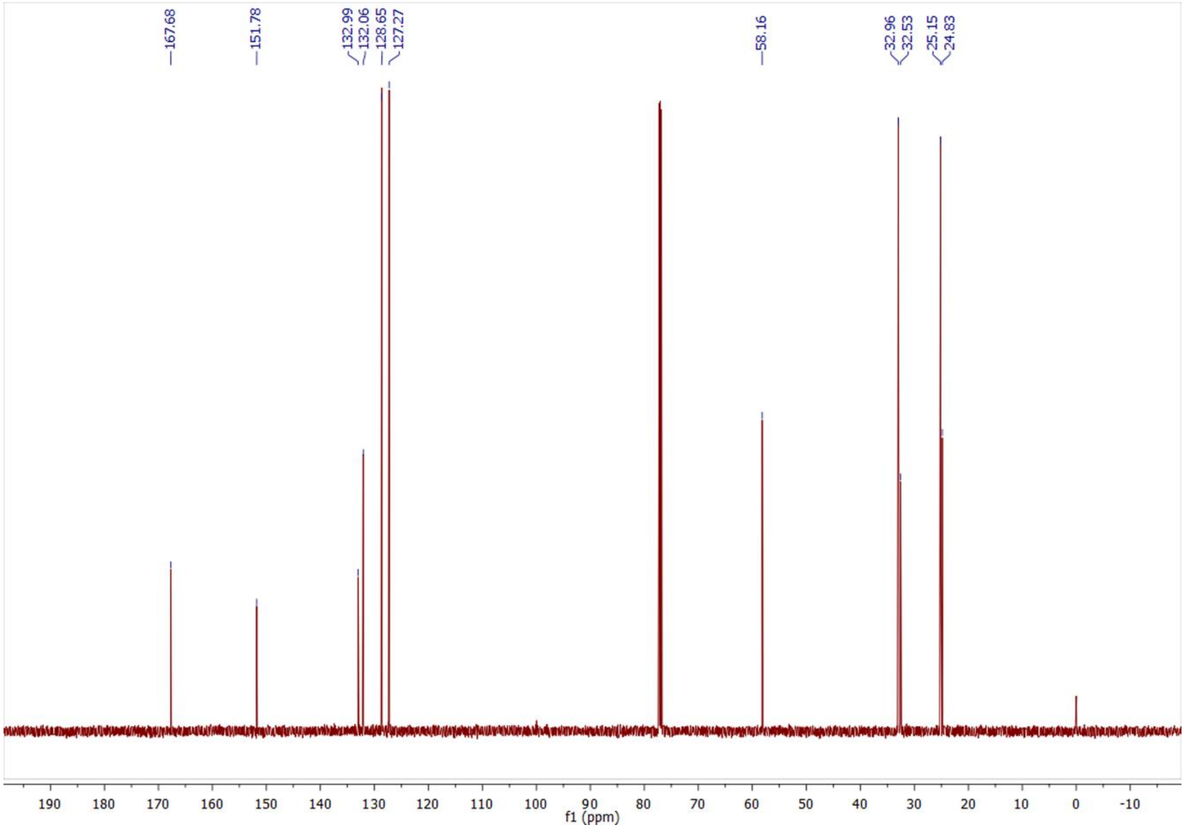

Figure S2. ^{13}C {^{1}H} NMR (151 MHz, $CDCl_3$) spectrum of compound **1a**.

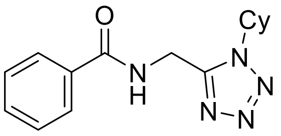

1a

N-((1-cyclohexyl-1*H*-tetrazol-5-yl)methyl)benzamide

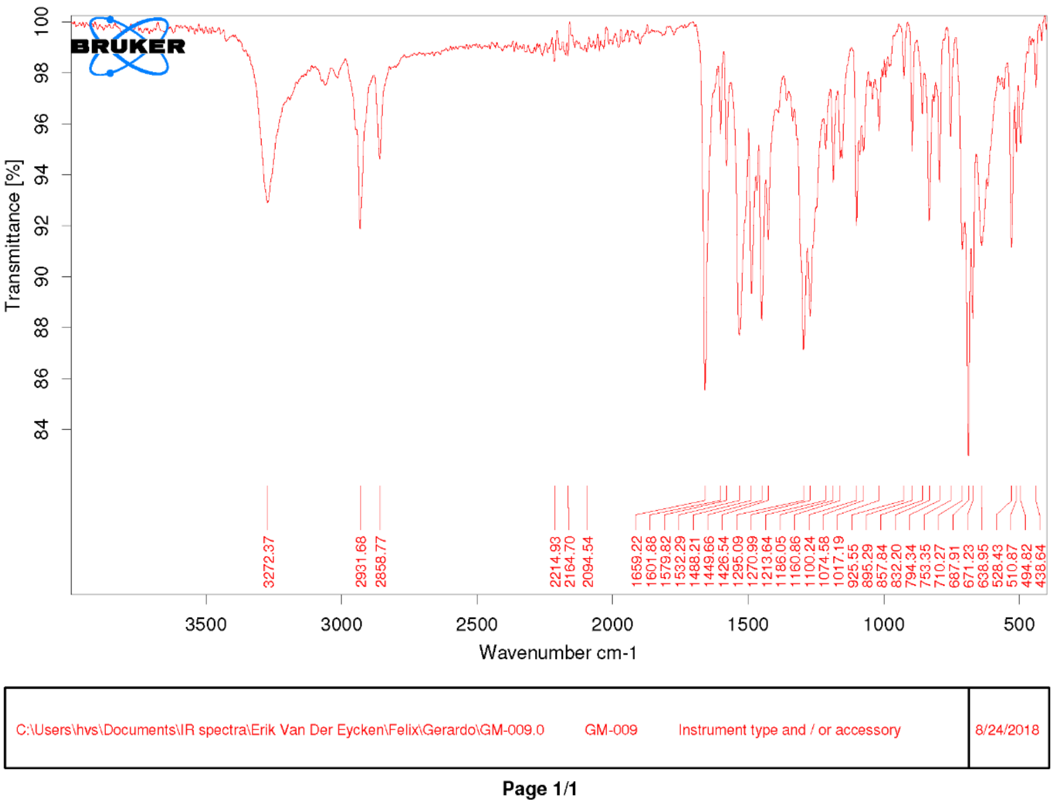

Figure S3. FT-IR (KBr) spectrum of compound **1a**.

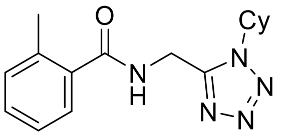

1b

N-((1-cyclohexyl-1*H*-tetrazol-5-yl)methyl)-2-methylbenzamide

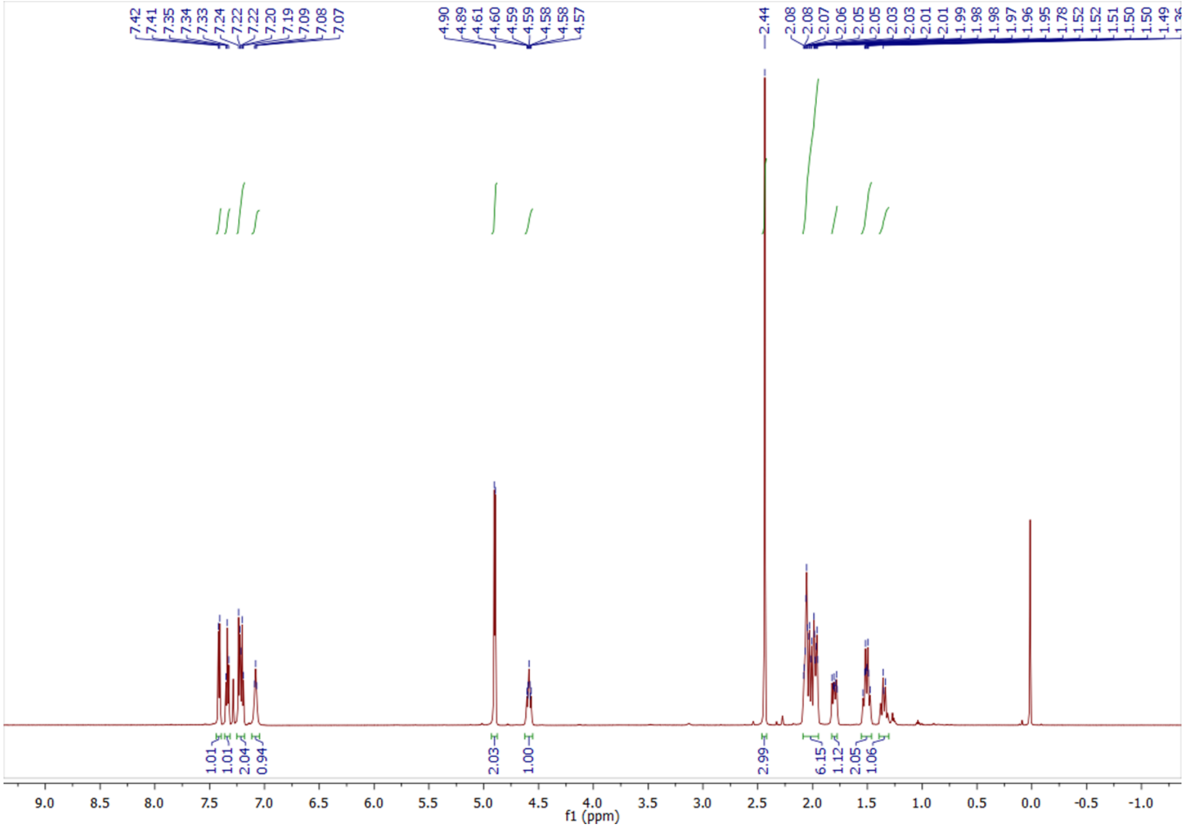

Figure S4. ^{1}H NMR (600 MHz, $CDCl_3$) spectrum of compound **1b**.

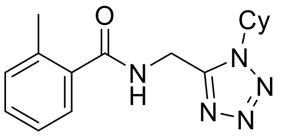

1b

N-((1-cyclohexyl-1*H*-tetrazol-5-yl)methyl)-2-methylbenzamide

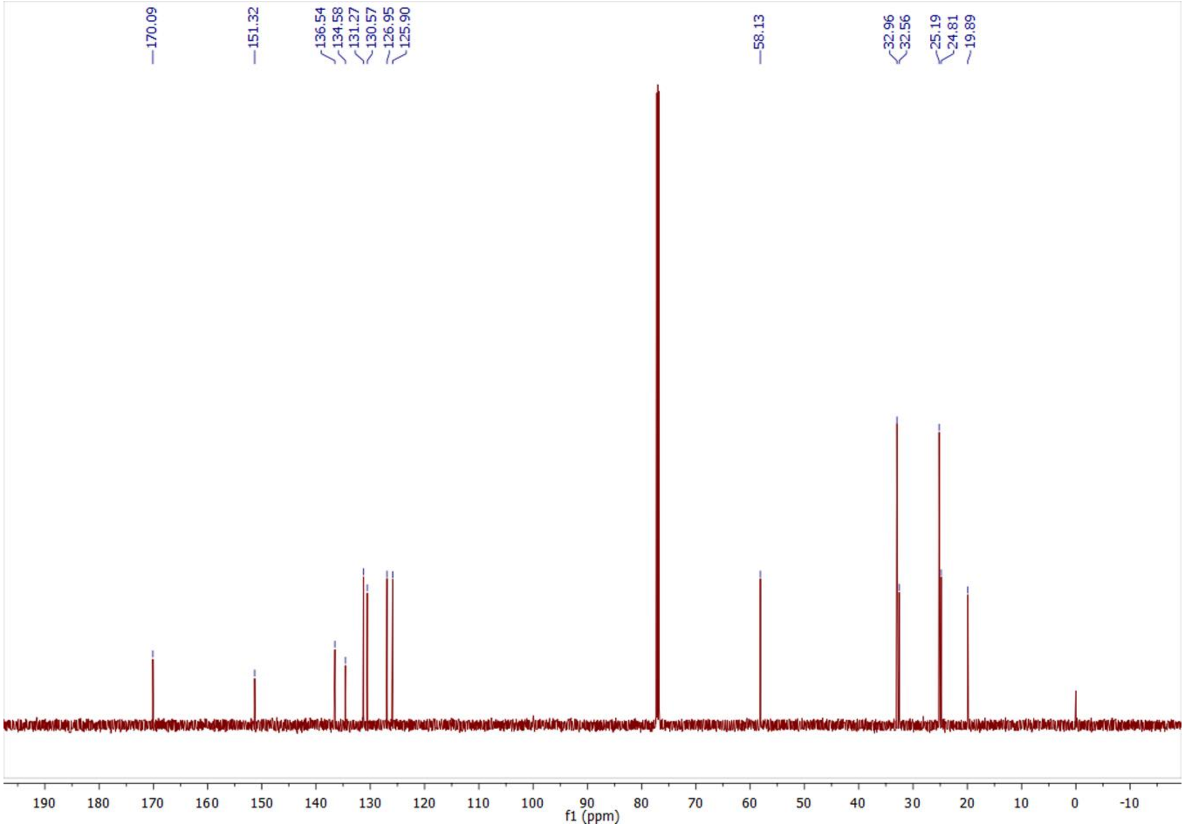

Figure S5. $^{13}C\ \{^{1}H\}$ NMR (151 MHz, $CDCl_3$) spectrum of compound **1b**.

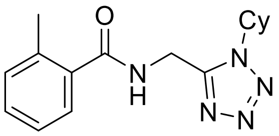

1b

N-((1-cyclohexyl-1*H*-tetrazol-5-yl)methyl)-2-methylbenzamide

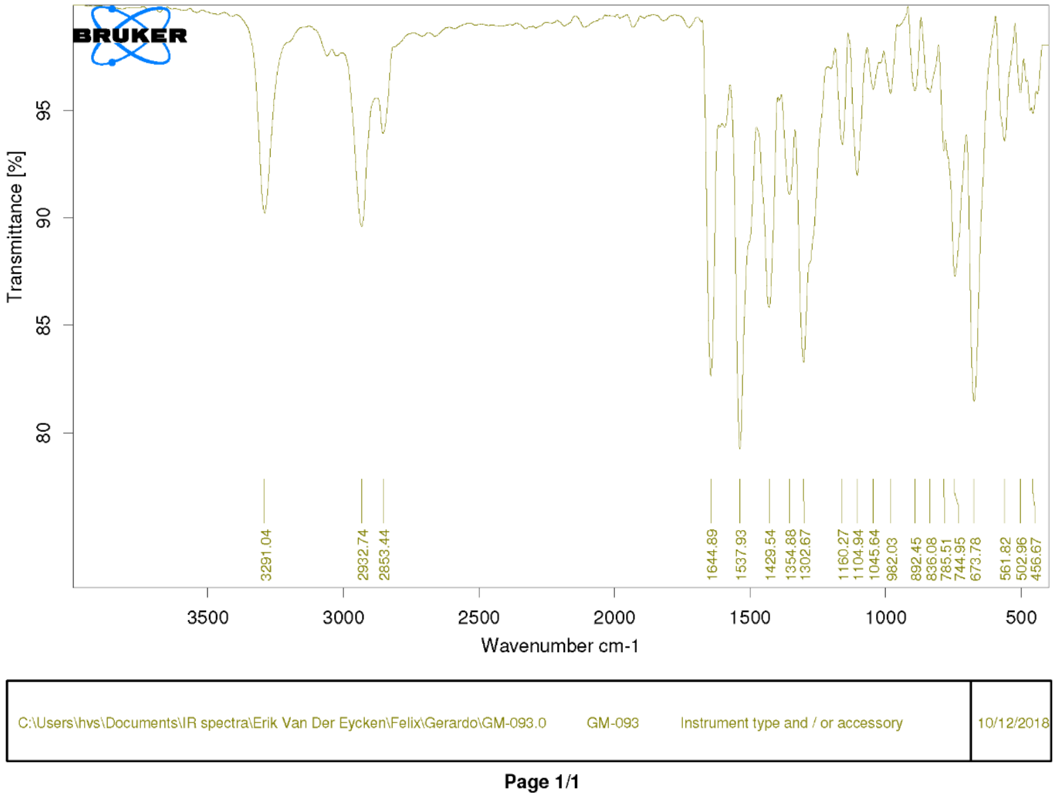

Figure S6. FT-IR (KBr) spectrum of compound **1b**.

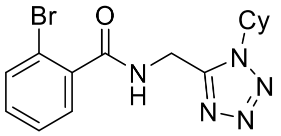

1c

2-bromo-*N*-((1-cyclohexyl-1*H*-tetrazol-5-yl)methyl)benzamide

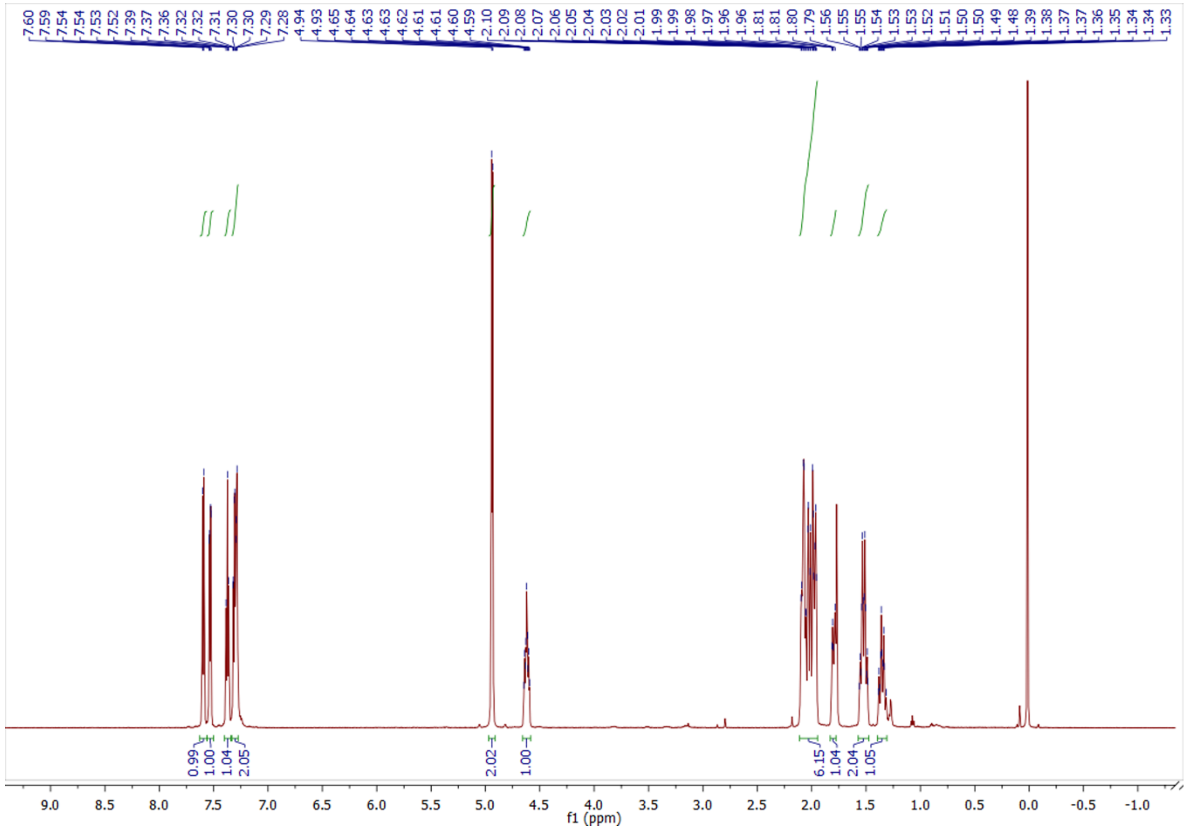

Figure S7. ^{1}H NMR (600 MHz, $CDCl_3$) spectrum of compound **1c**.

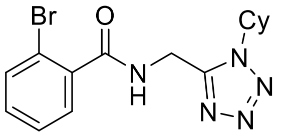

1c

2-bromo-*N*-((1-cyclohexyl-1*H*-tetrazol-5-yl)methyl)benzamide

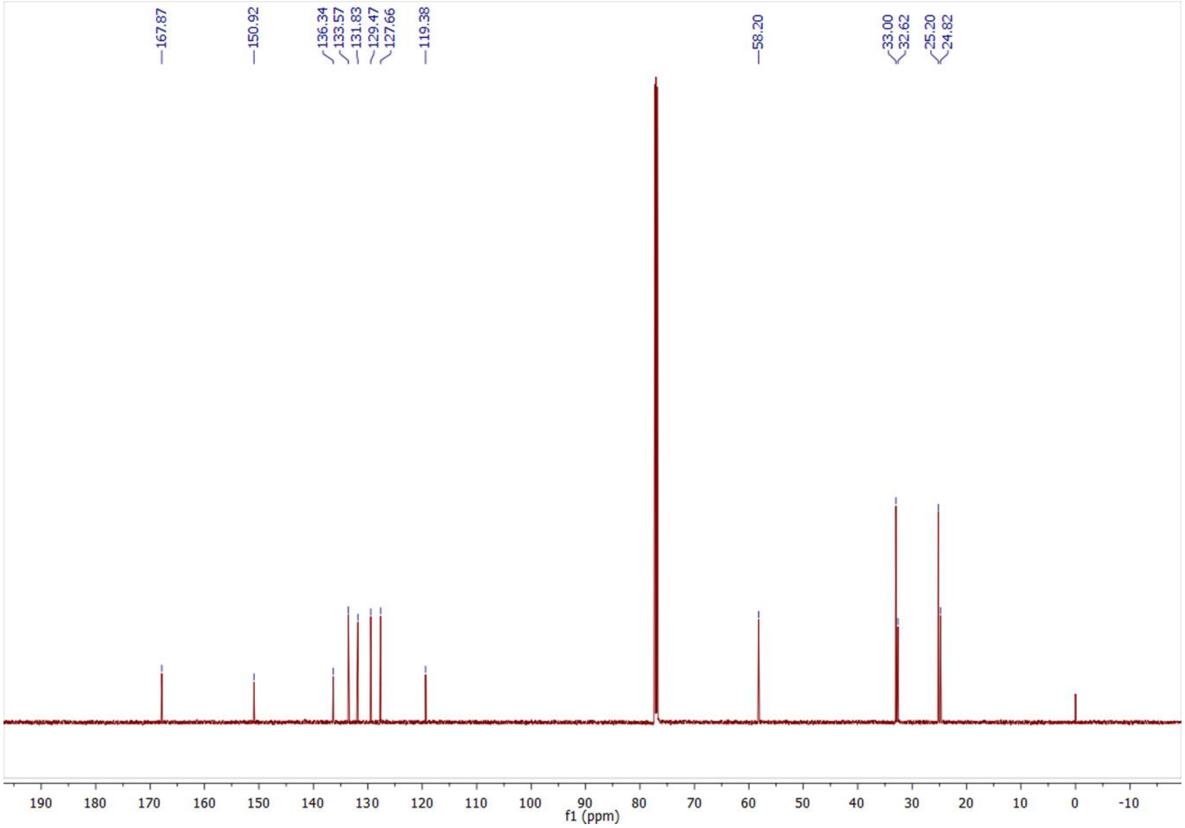

Figure S8. ^{13}C {^{1}H} NMR (151 MHz, $CDCl_3$) spectrum of compound **1c**.

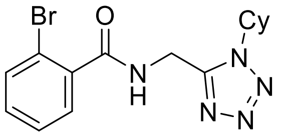

1c

2-bromo-*N*-((1-cyclohexyl-1*H*-tetrazol-5-yl)methyl)benzamide

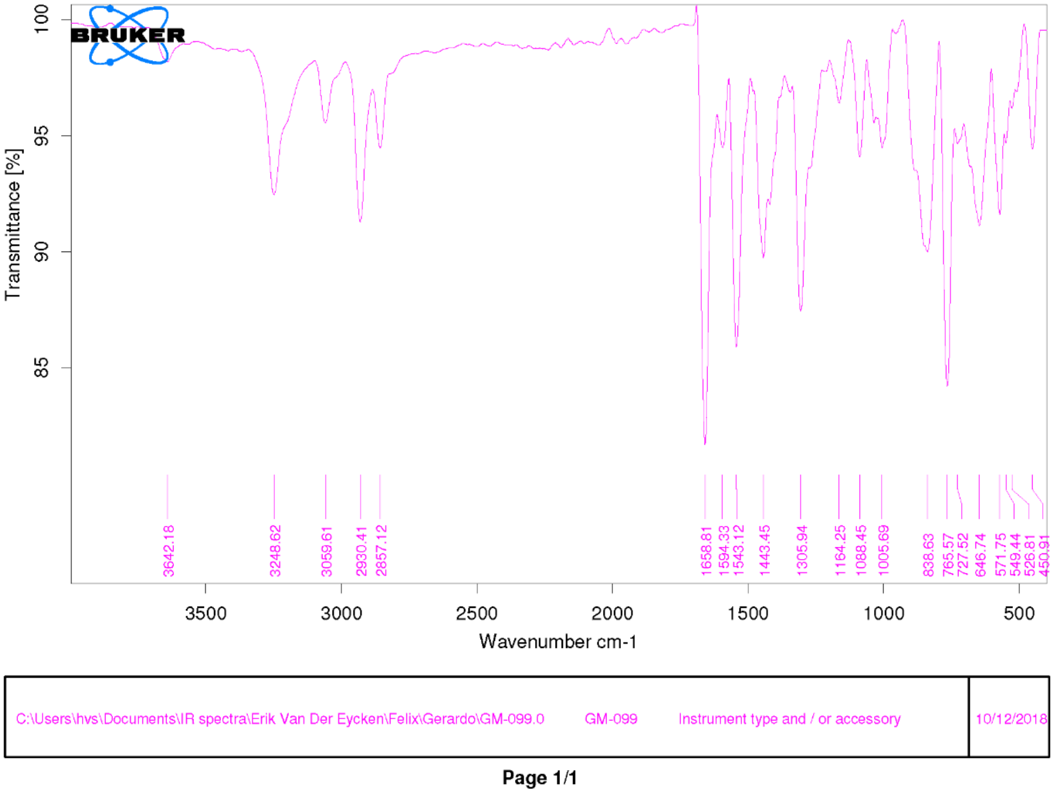

Figure S9. FT-IR (KBr) spectrum of compound **1c**.

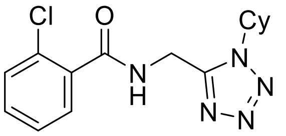

1d

2-chloro-*N*-((1-cyclohexyl-1*H*-tetrazol-5-yl)methyl)benzamide

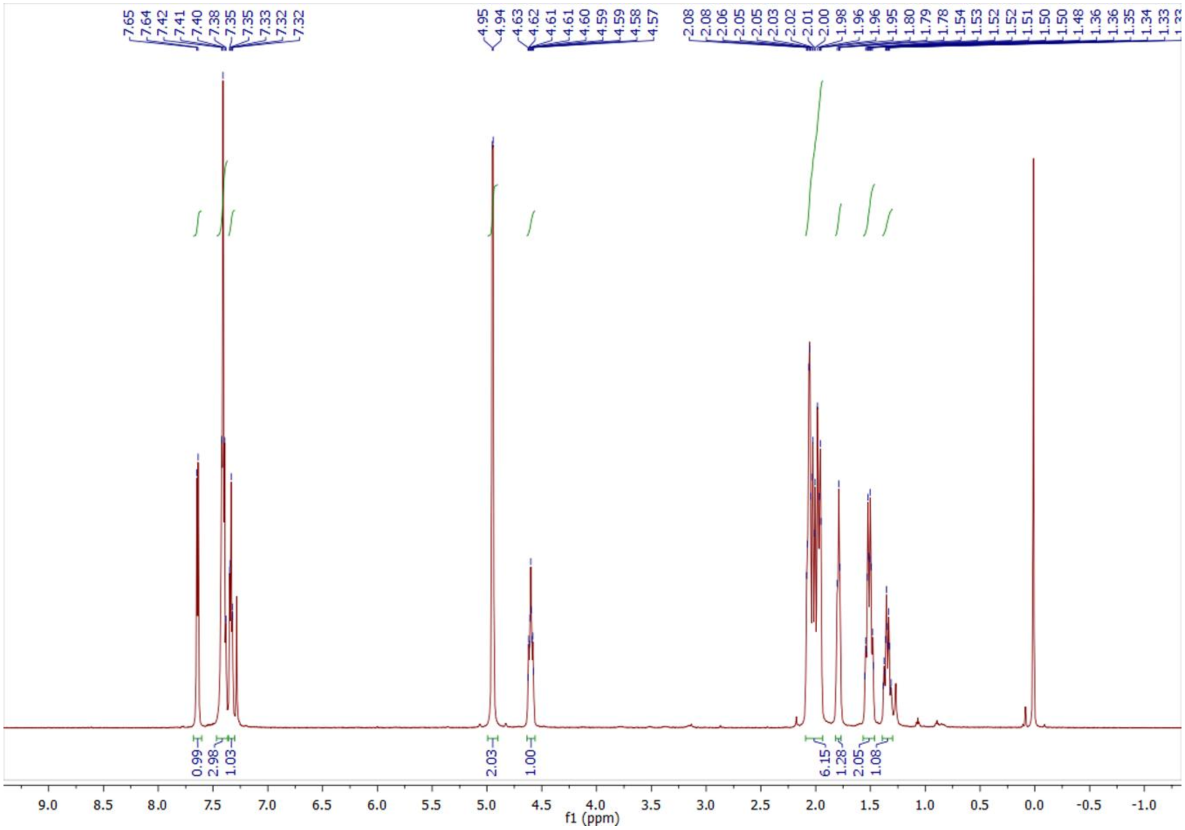

Figure S10. ^{1}H NMR (600 MHz, $CDCl_3$) spectrum of compound **1d**.

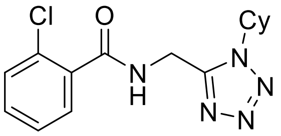

1d

2-chloro-*N*-((1-cyclohexyl-1*H*-tetrazol-5-yl)methyl)benzamide

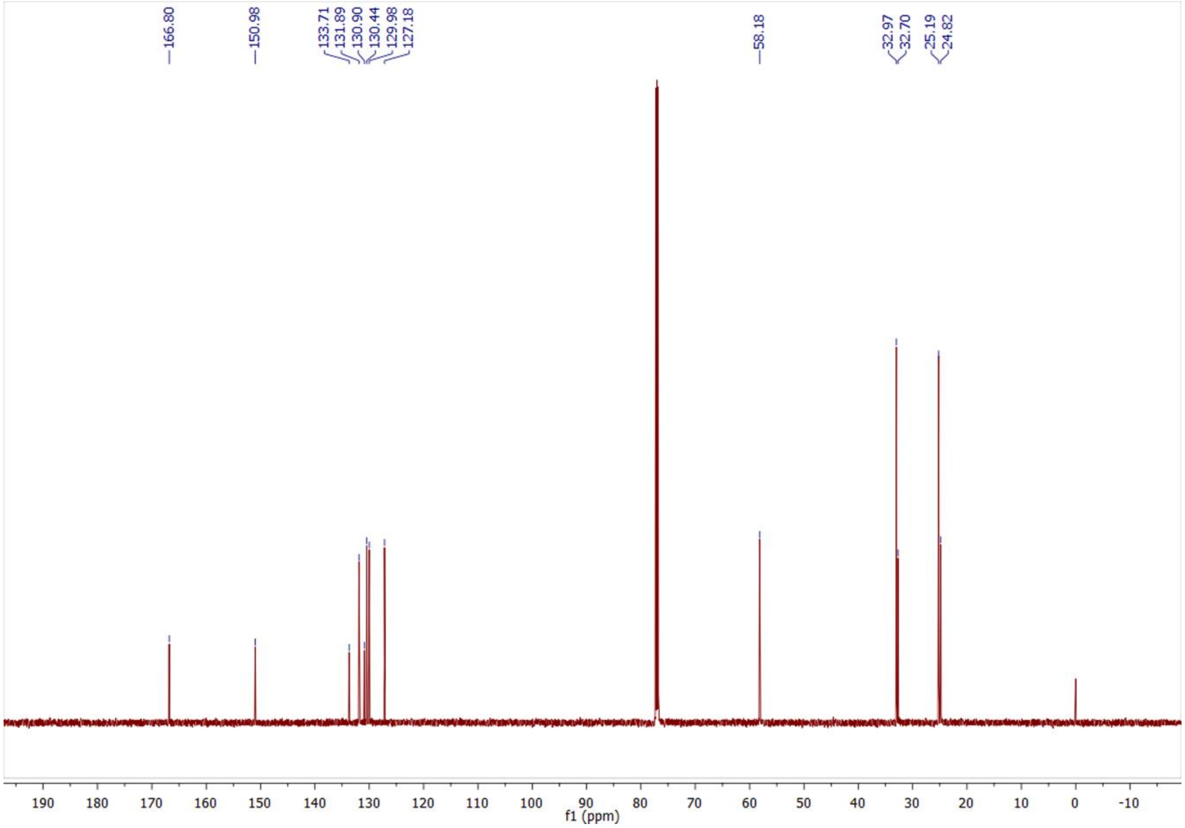

Figure S11. $^{13}C\ \{^1H\}$ NMR (151 MHz, $CDCl_3$) spectrum of compound **1d**.

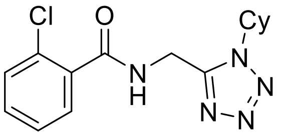

1d

2-chloro-*N*-((1-cyclohexyl-1*H*-tetrazol-5-yl)methyl)benzamide

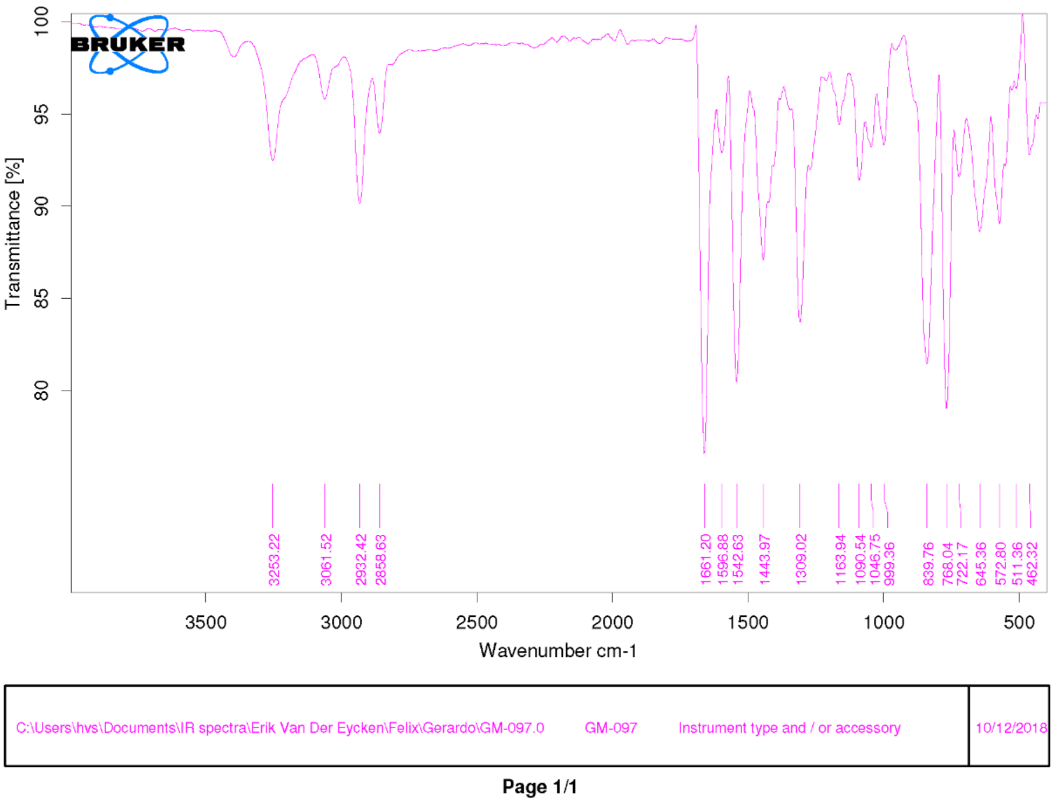

Figure S12. FT-IR (KBr) spectrum of compound **1d**.

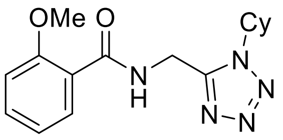

1e

N-((1-cyclohexyl-1*H*-tetrazol-5-yl)methyl)-2-methoxybenzamide

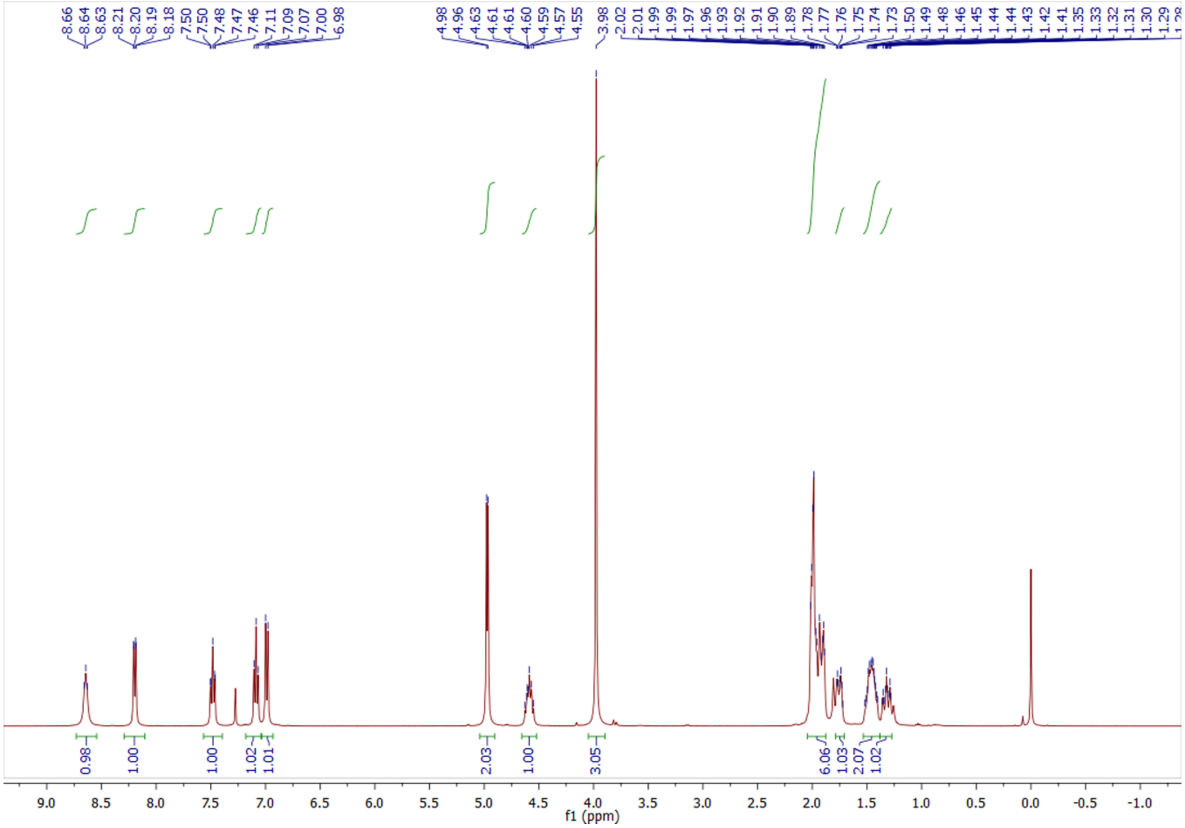

Figure S13. ^{1}H NMR (400 MHz, $CDCl_3$) spectrum of compound **1e**.

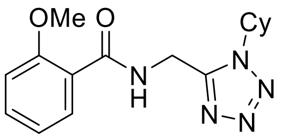

1e

N-((1-cyclohexyl-1*H*-tetrazol-5-yl)methyl)-2-methoxybenzamide

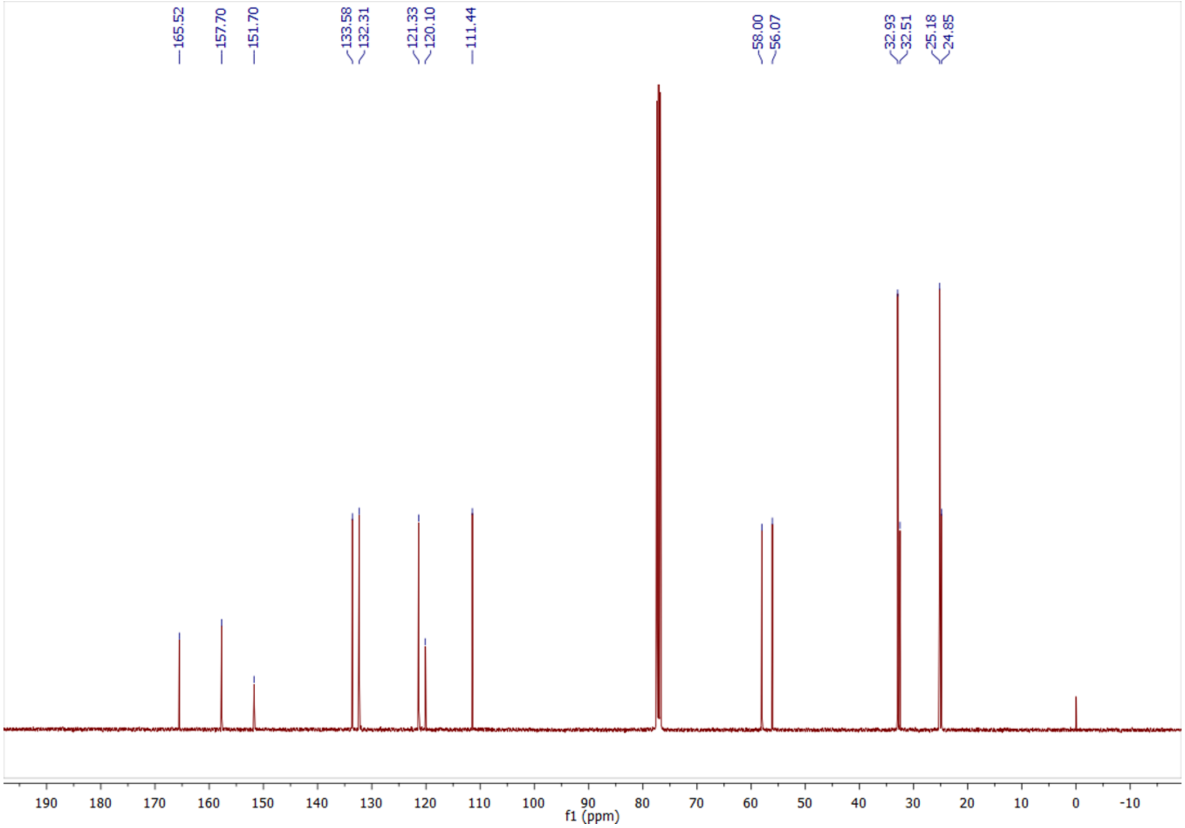

Figure S14. $^{13}C\ \{^{1}H\}$ NMR (101 MHz, $CDCl_3$) spectrum of compound **1e**.

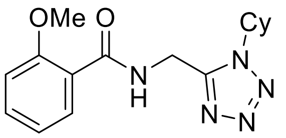

1e

N-((1-cyclohexyl-1*H*-tetrazol-5-yl)methyl)-2-methoxybenzamide

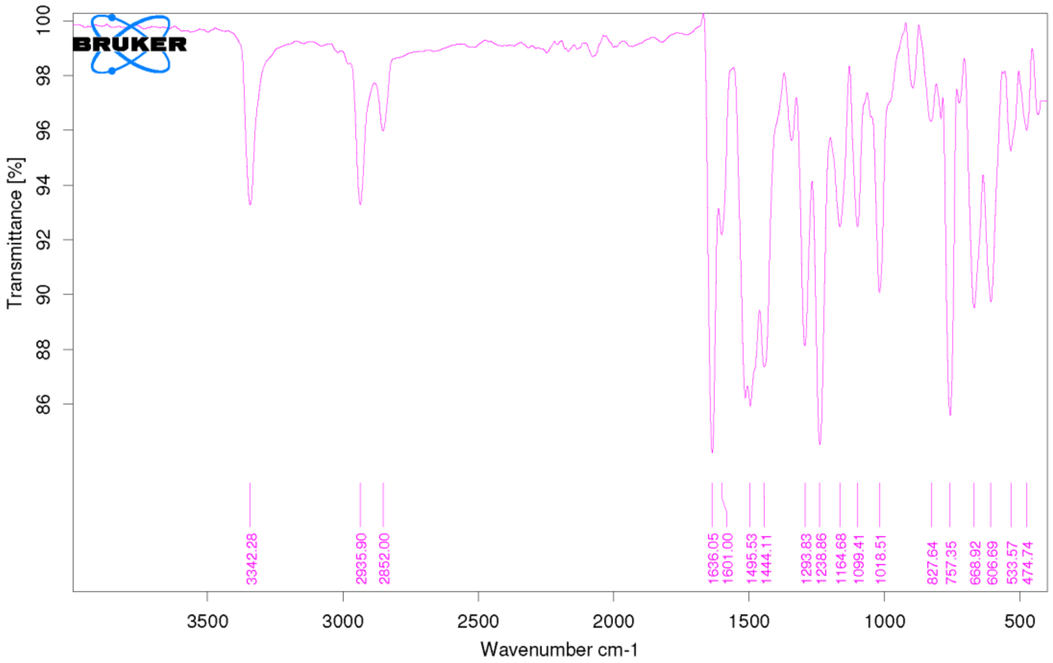

C:\Users\hvs\Documents\IR spectra\Erik Van Der Eycken\Felix\Gerardo\GM-095.0	GM-095	Instrument type and / or accessory	1/21/2019

Page 1/1

Figure S15. FT-IR (KBr) spectrum of compound **1e**.

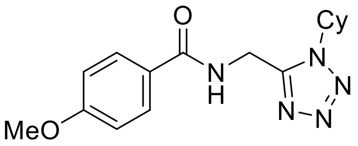

1f

N-((1-cyclohexyl-1*H*-tetrazol-5-yl)methyl)-4-methoxybenzamide

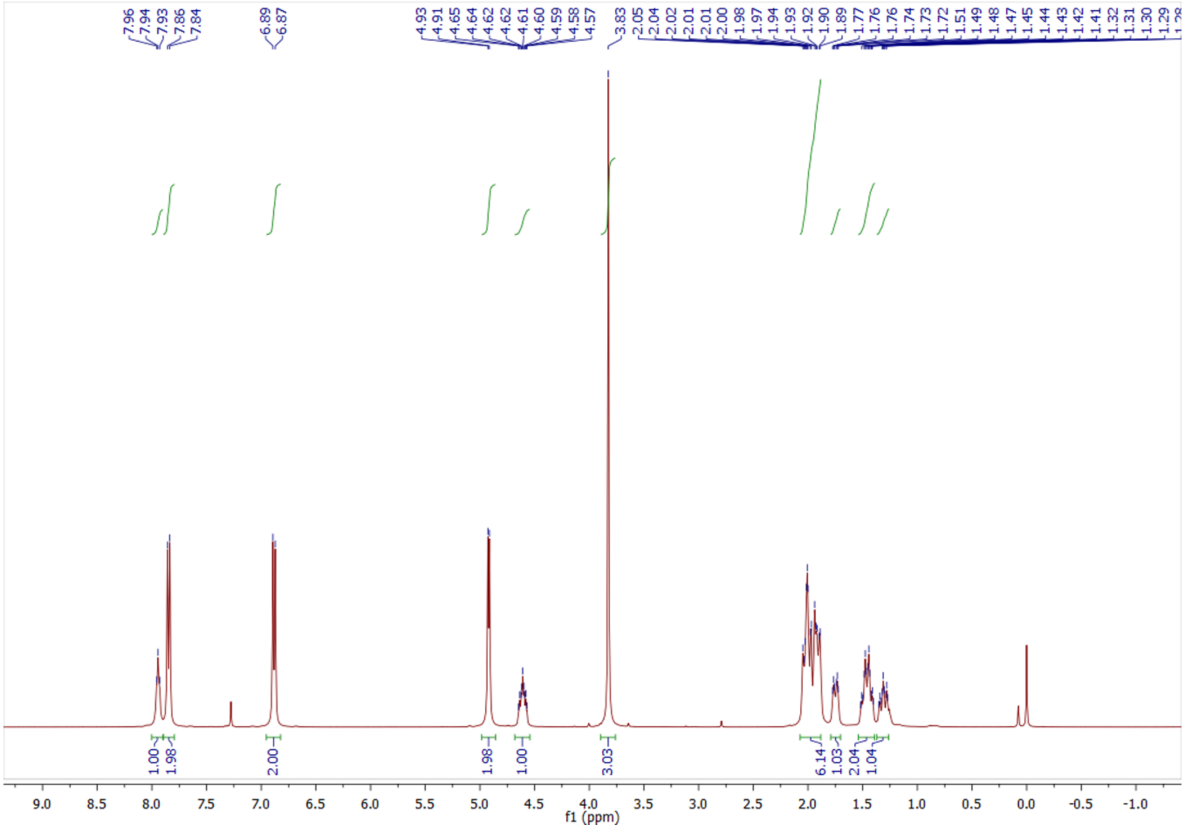

Figure S16. ^{1}H NMR (400 MHz, DMSO-*d*6) spectrum of compound **1f**.

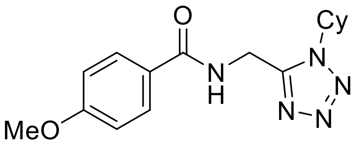

1f

N-((1-cyclohexyl-1*H*-tetrazol-5-yl)methyl)-4-methoxybenzamide

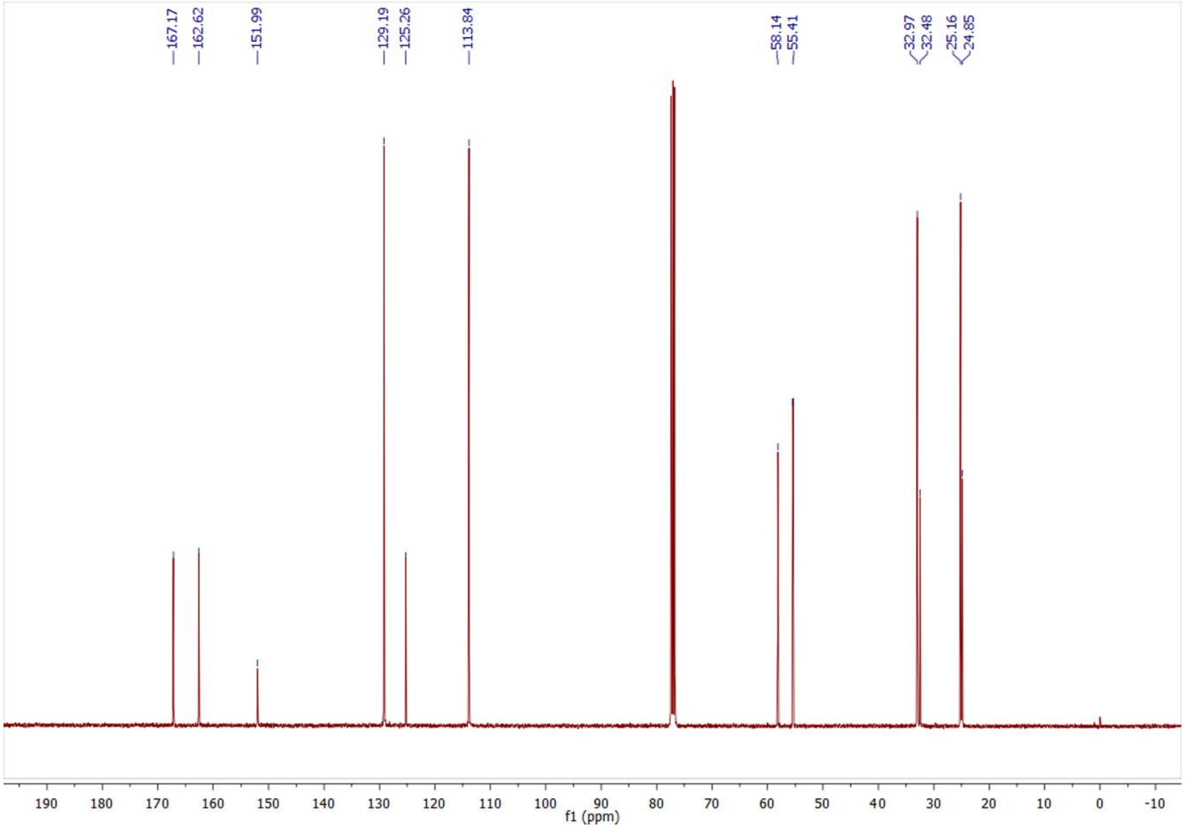

Figure S17. ^{13}C {^{1}H} NMR (101 MHz, DMSO-*d*6) spectrum of compound **1f**.

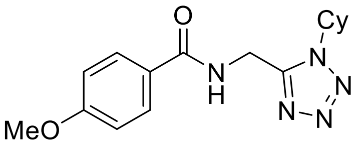

1f

N-((1-cyclohexyl-1*H*-tetrazol-5-yl)methyl)-4-methoxybenzamide

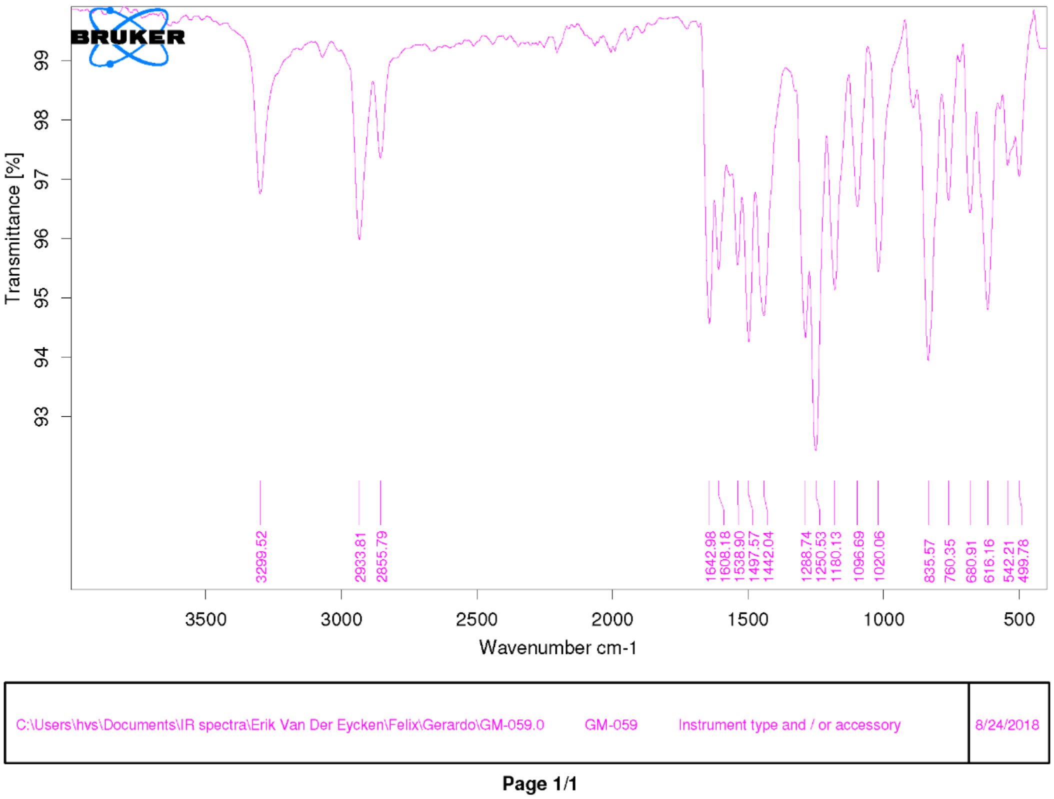

Figure S18. FT-IR (KBr) spectrum of compound **1f**.

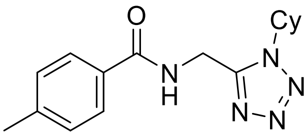

1g

N-((1-cyclohexyl-1*H*-tetrazol-5-yl)methyl)-4-methylbenzamide

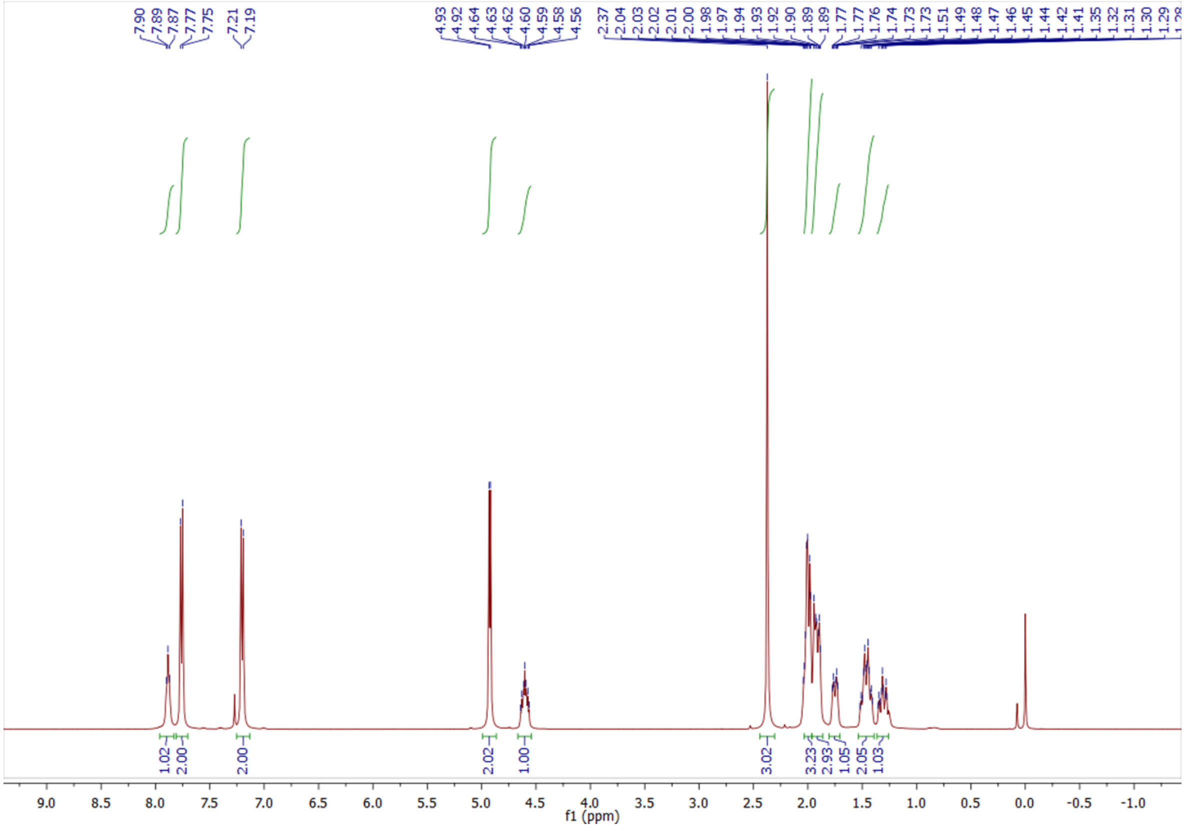

Figure S19. ^{1}H NMR (400 MHz, $CDCl_3$) spectrum of compound **1g**.

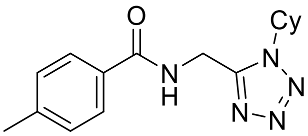

1g

N-((1-cyclohexyl-1*H*-tetrazol-5-yl)methyl)-4-methylbenzamide

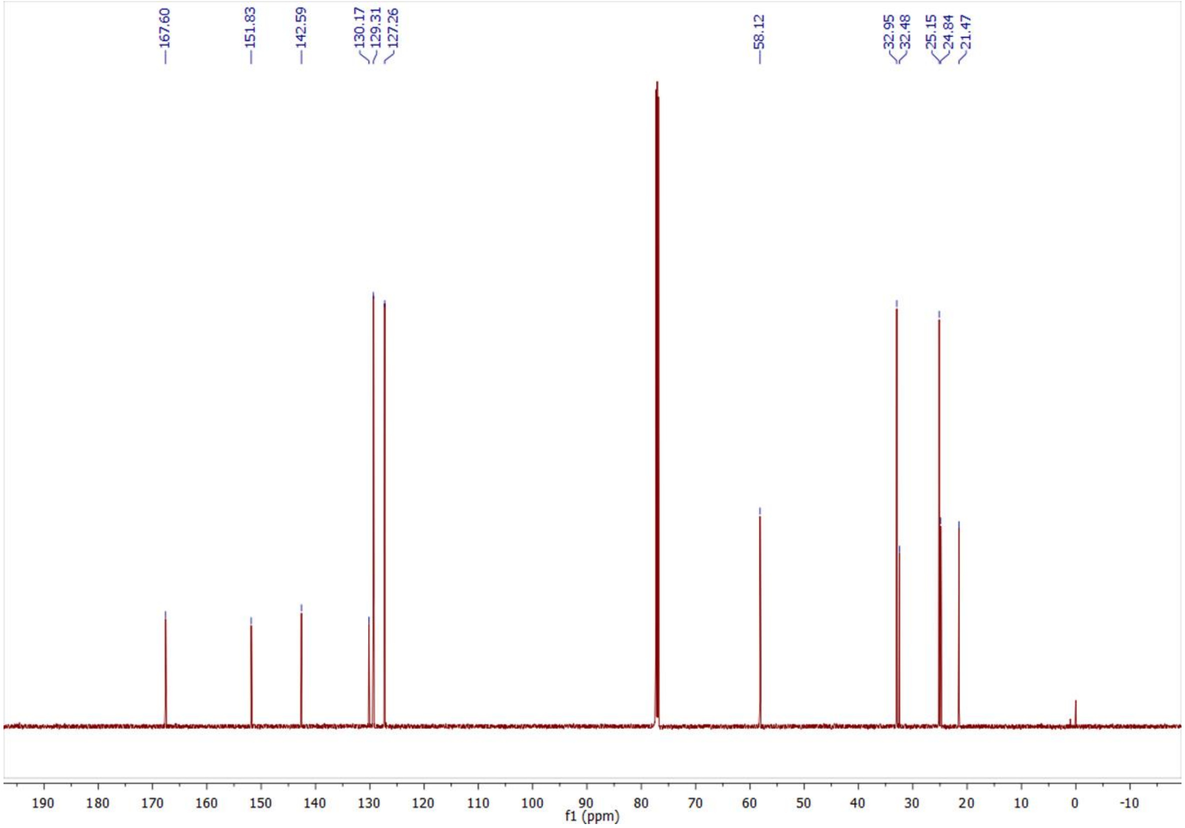

Figure S20. $^{13}C\{^{1}H\}$ NMR (151 MHz, $CDCl_3$) spectrum of compound **1g**.

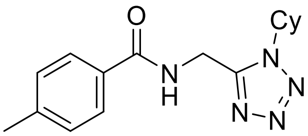

1g

N-((1-cyclohexyl-1*H*-tetrazol-5-yl)methyl)-4-methylbenzamide

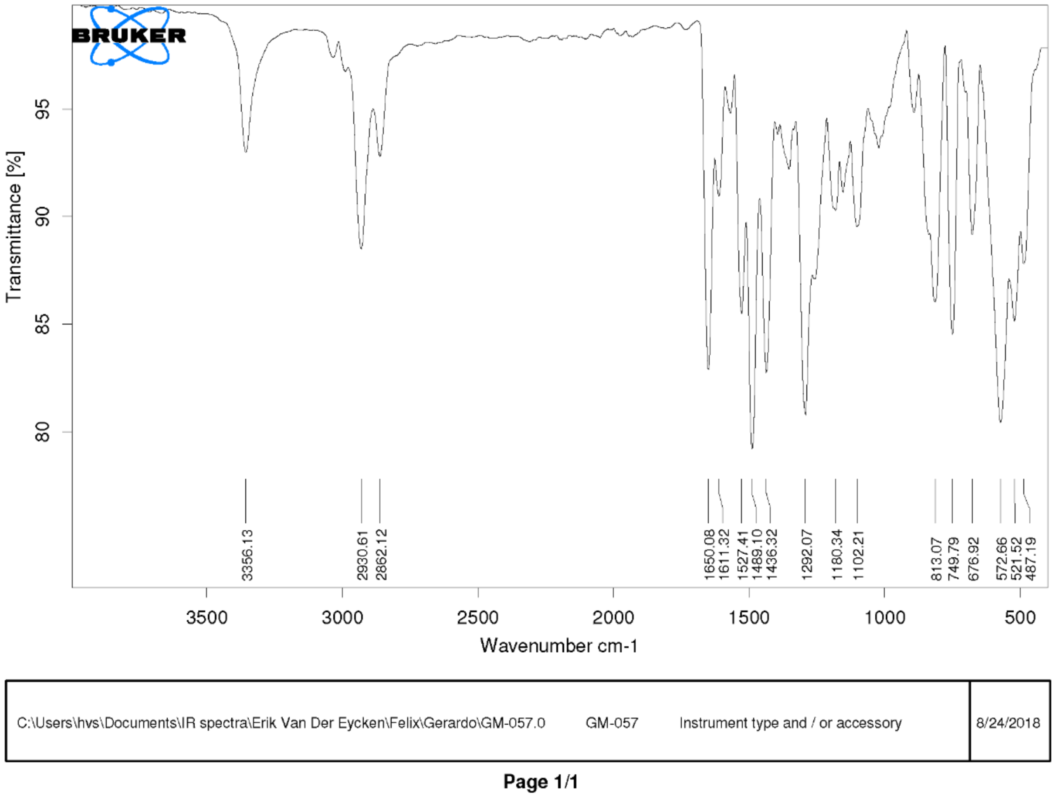

Figure S21. FT-IR (KBr) spectrum of compound **1g**.

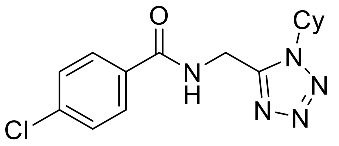

1h

4-chloro-*N*-((1-cyclohexyl-1*H*-tetrazol-5-yl)methyl)benzamide

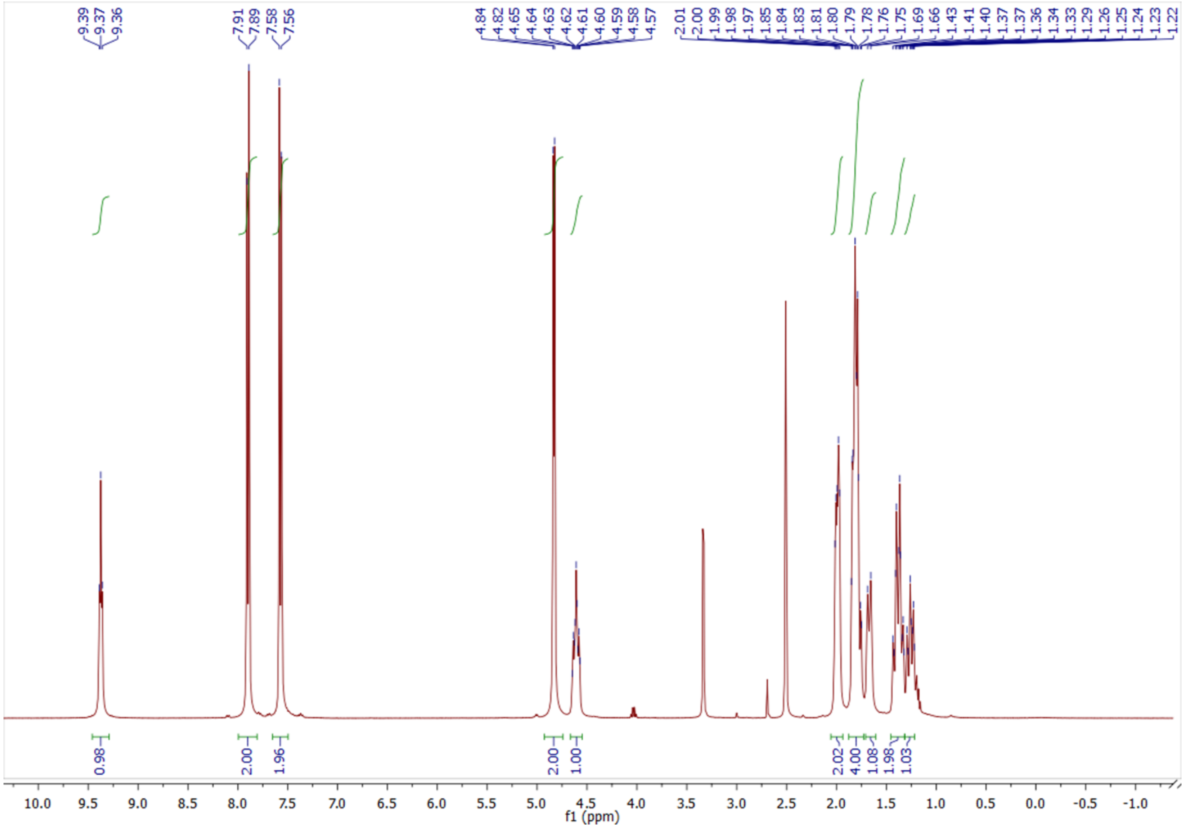

Figure S22. ^{1}H NMR (400 MHz, DMSO-*d*6) spectrum of compound **1h**.

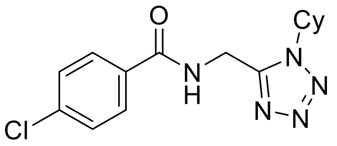

1h

4-chloro-*N*-((1-cyclohexyl-1*H*-tetrazol-5-yl)methyl)benzamide

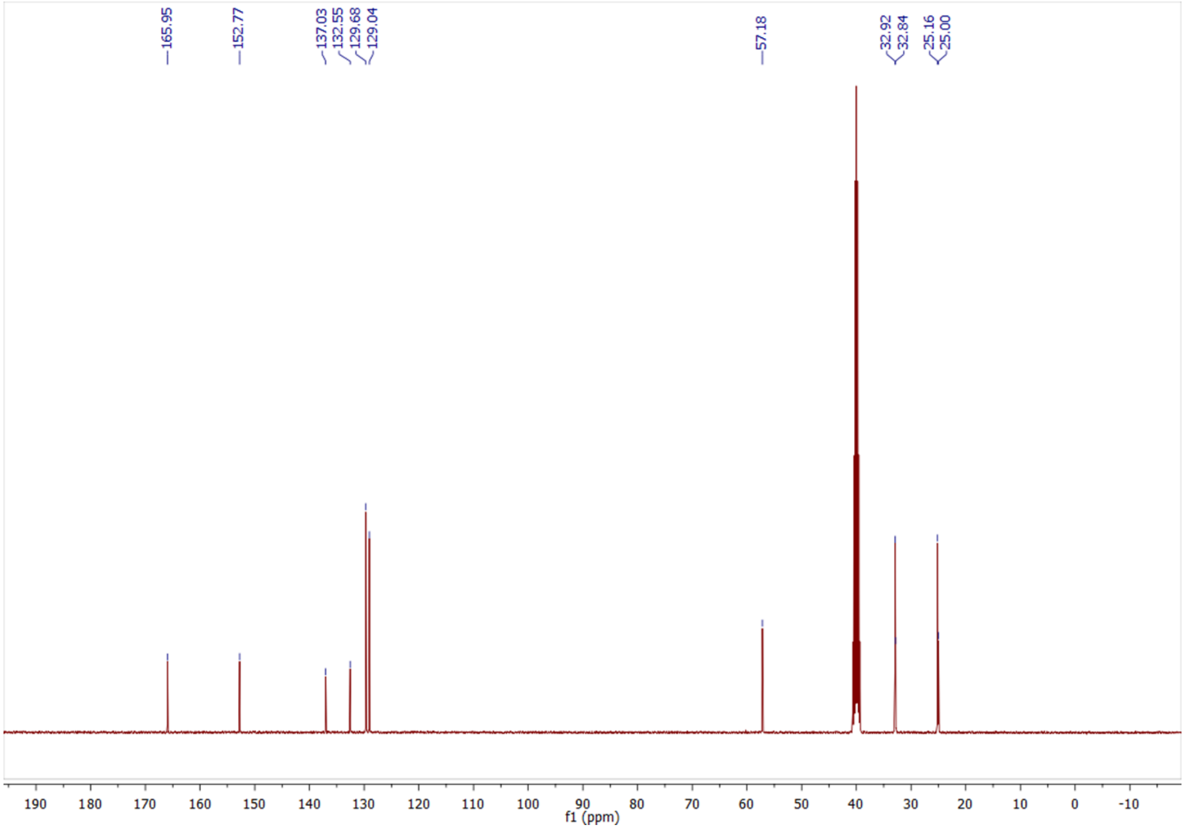

Figure S23. ^{13}C {^{1}H} NMR (101 MHz, DMSO-*d*6) spectrum of compound **1h**.

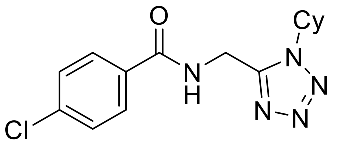

1h

4-chloro-*N*-((1-cyclohexyl-1*H*-tetrazol-5-yl)methyl)benzamide

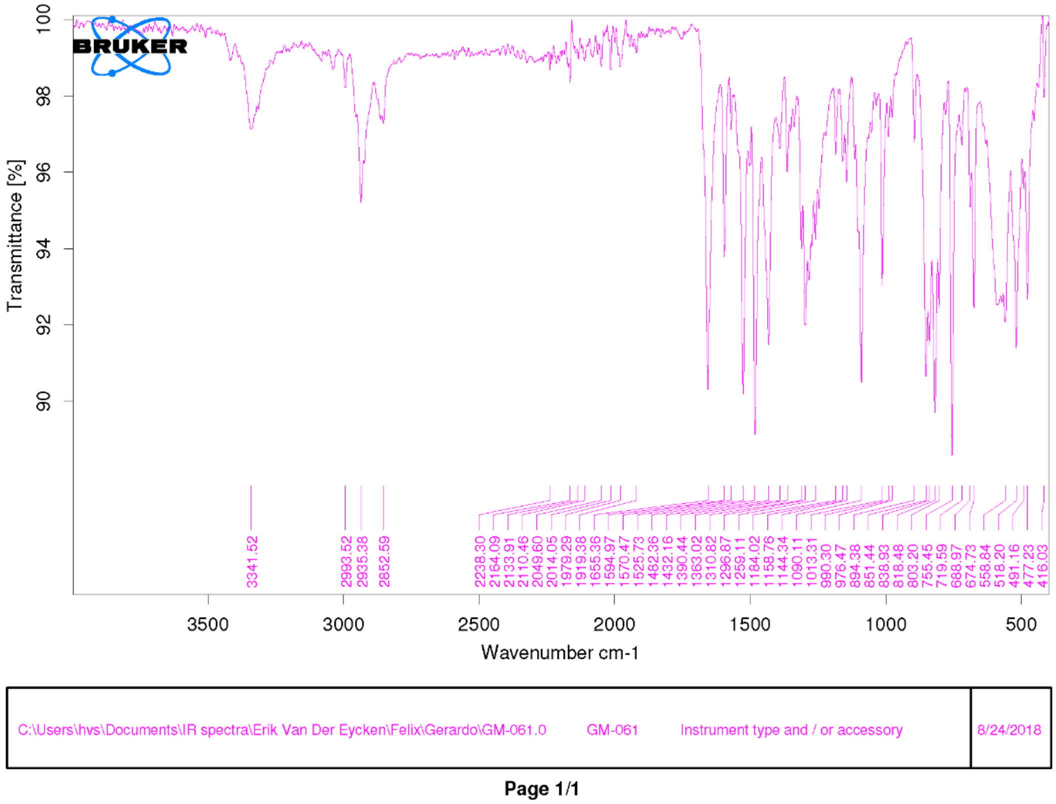

Figure S24. FT-IR (KBr) spectrum of compound **1h**.

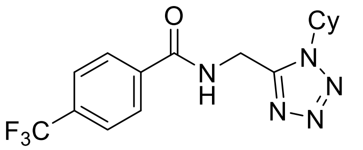

1i

N-((1-cyclohexyl-1*H*-tetrazol-5-yl)methyl)-4-(trifluoromethyl)benzamide

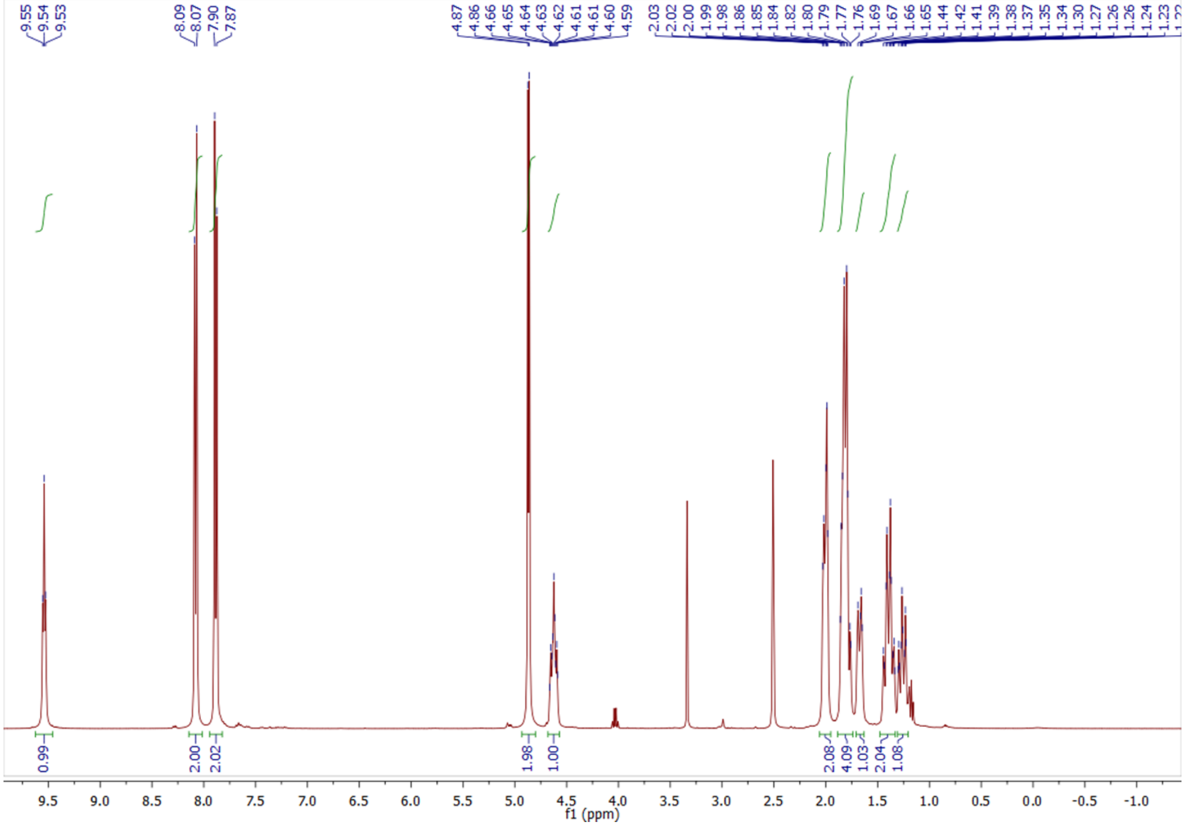

Figure S25. ^{1}H NMR (400 MHz, DMSO-*d*6) spectrum of compound **1i**.

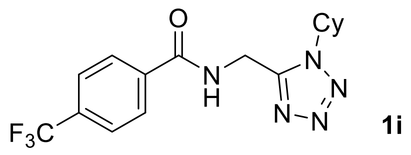

N-((1-cyclohexyl-1*H*-tetrazol-5-yl)methyl)-4-(trifluoromethyl)benzamide

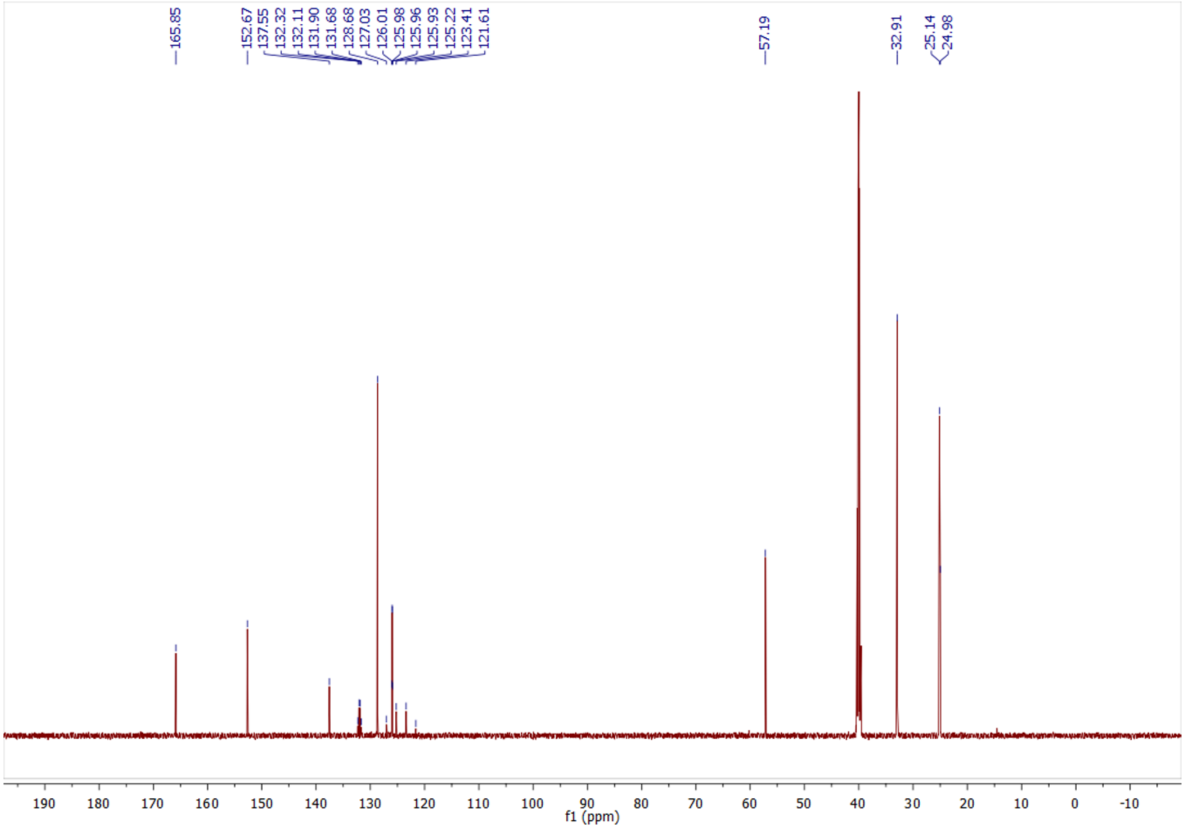

Figure S26. ^{13}C {^{1}H} NMR (151 MHz, DMSO-*d*6) spectrum of compound **1i**.

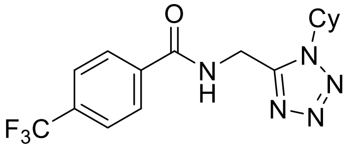

1i

N-((1-cyclohexyl-1*H*-tetrazol-5-yl)methyl)-4-(trifluoromethyl)benzamide

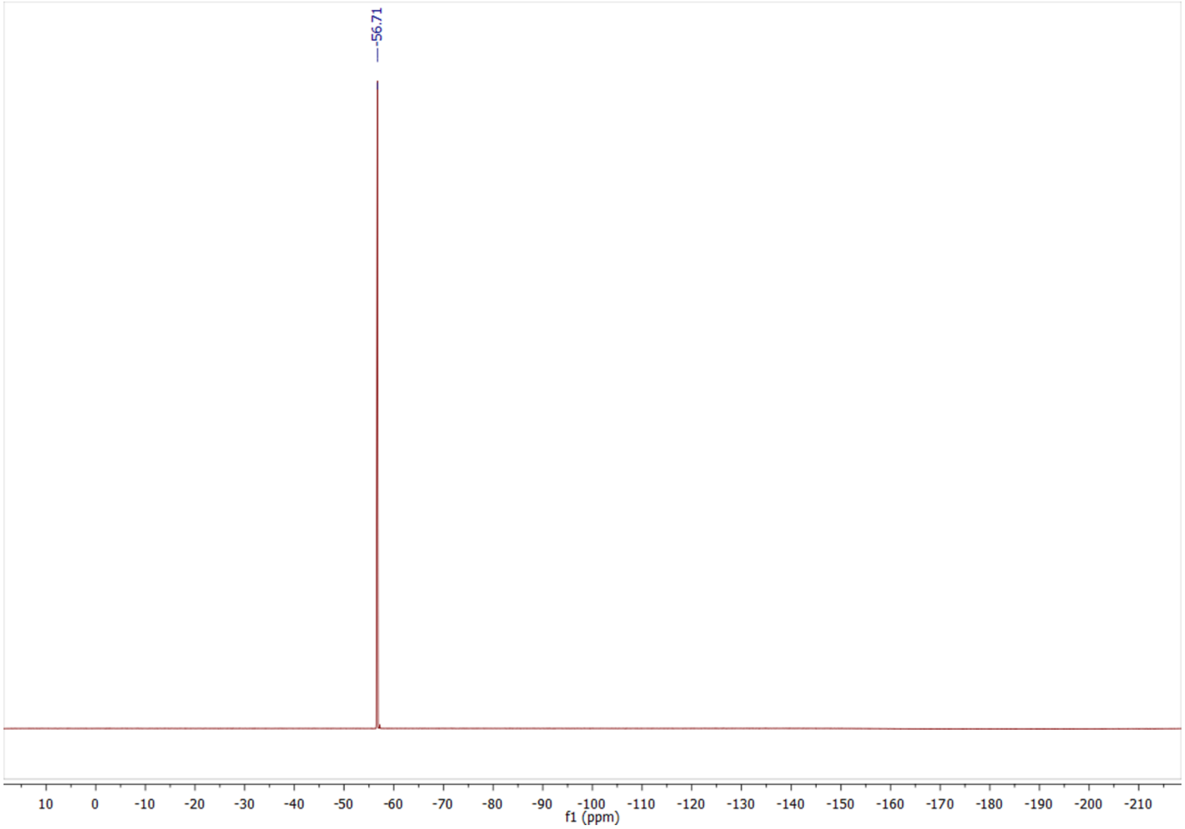

Figure S27. ^{19}F {^{13}C} NMR (376 MHz, $CDCl_3$) spectrum of compound **1i**.

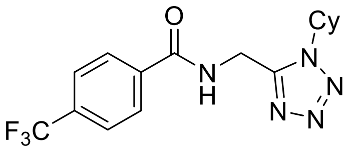

1i

N-((1-cyclohexyl-1*H*-tetrazol-5-yl)methyl)-4-(trifluoromethyl)benzamide

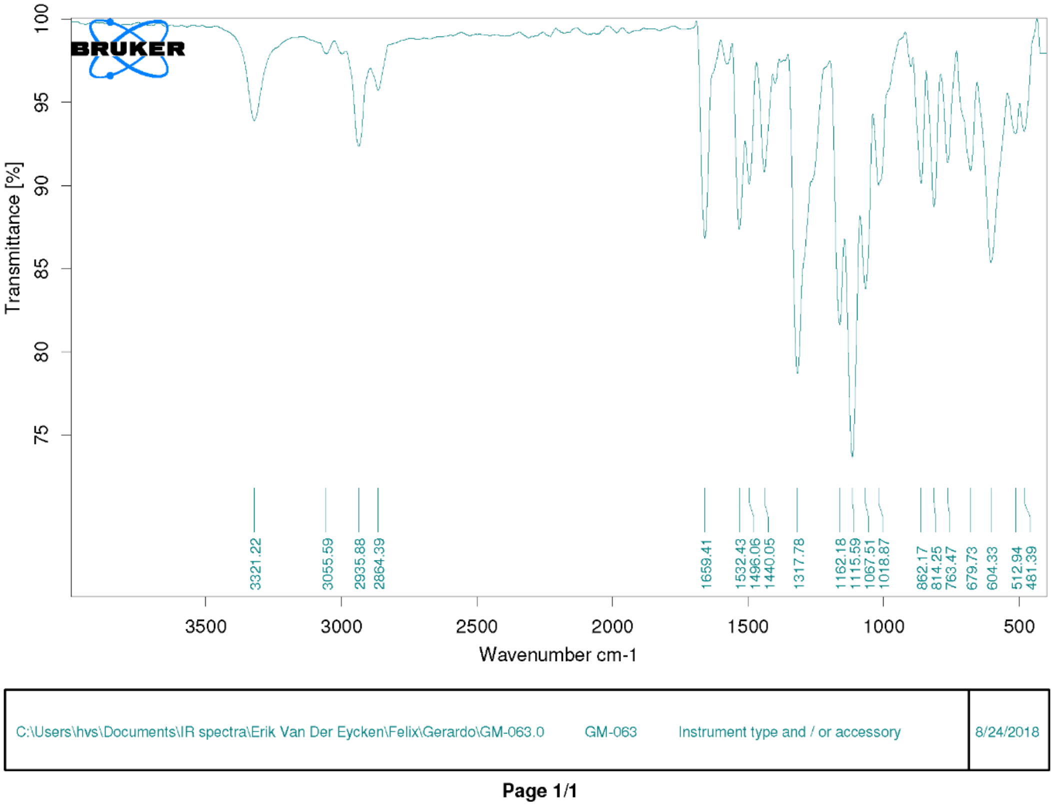

Figure S28. FT-IR (KBr) spectrum of compound **1i**.

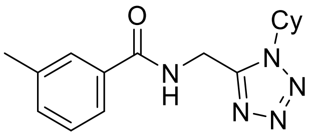

1j

N-((1-cyclohexyl-1*H*-tetrazol-5-yl)methyl)-3-methylbenzamide

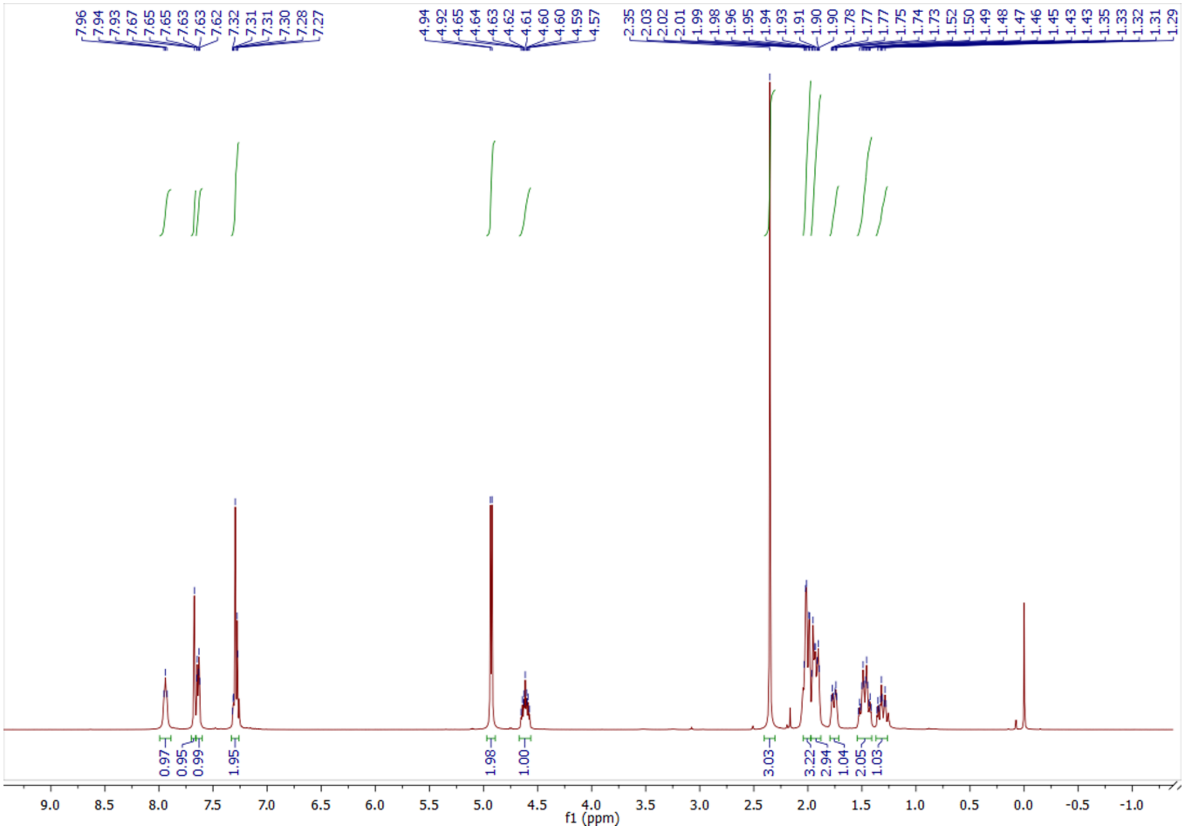

Figure S29. ^{1}H NMR (400 MHz, $CDCl_3$) spectrum of compound **1j**.

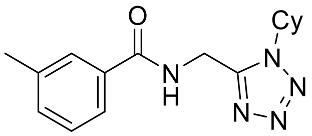

1j

N-((1-cyclohexyl-1*H*-tetrazol-5-yl)methyl)-3-methylbenzamide

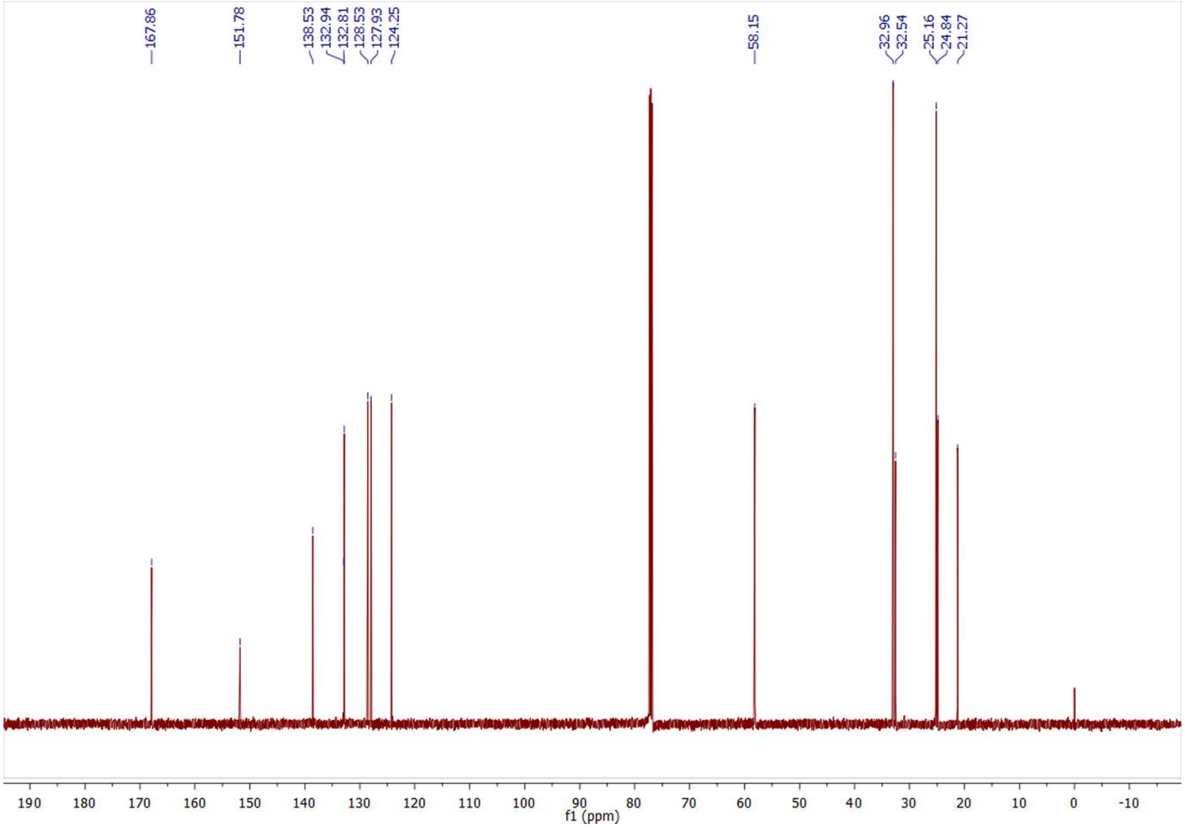

Figure S30. $^{13}C\ \{^{1}H\}$ NMR (151 MHz, $CDCl_3$) spectrum of compound **1j**.

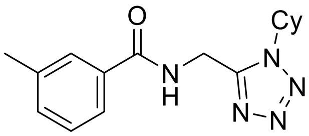

1j

N-((1-cyclohexyl-1*H*-tetrazol-5-yl)methyl)-3-methylbenzamide

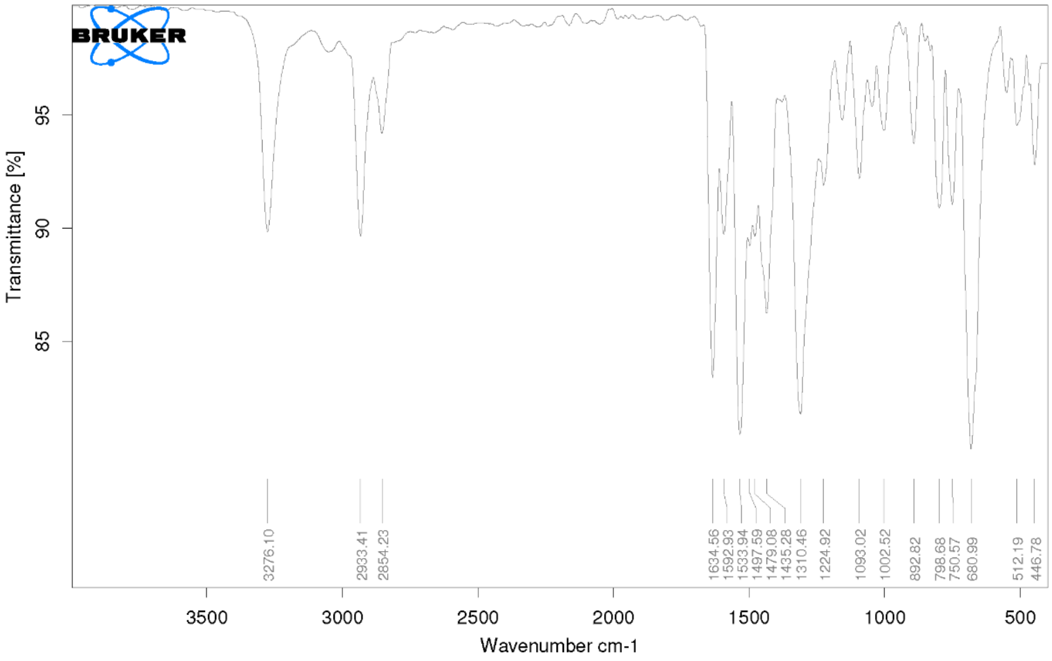

C:\Users\hvs\Documents\IR spectra\Erik Van Der Eycken\Felix\Gerardo\GM-087.0 GM-087 Instrument type and / or accessory	10/12/2018

Page 1/1

Figure S31. FT-IR (KBr) spectrum of compound **1j**.

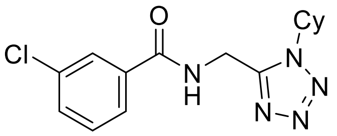

1k

3-chloro-*N*-((1-cyclohexyl-1*H*-tetrazol-5-yl)methyl)benzamide

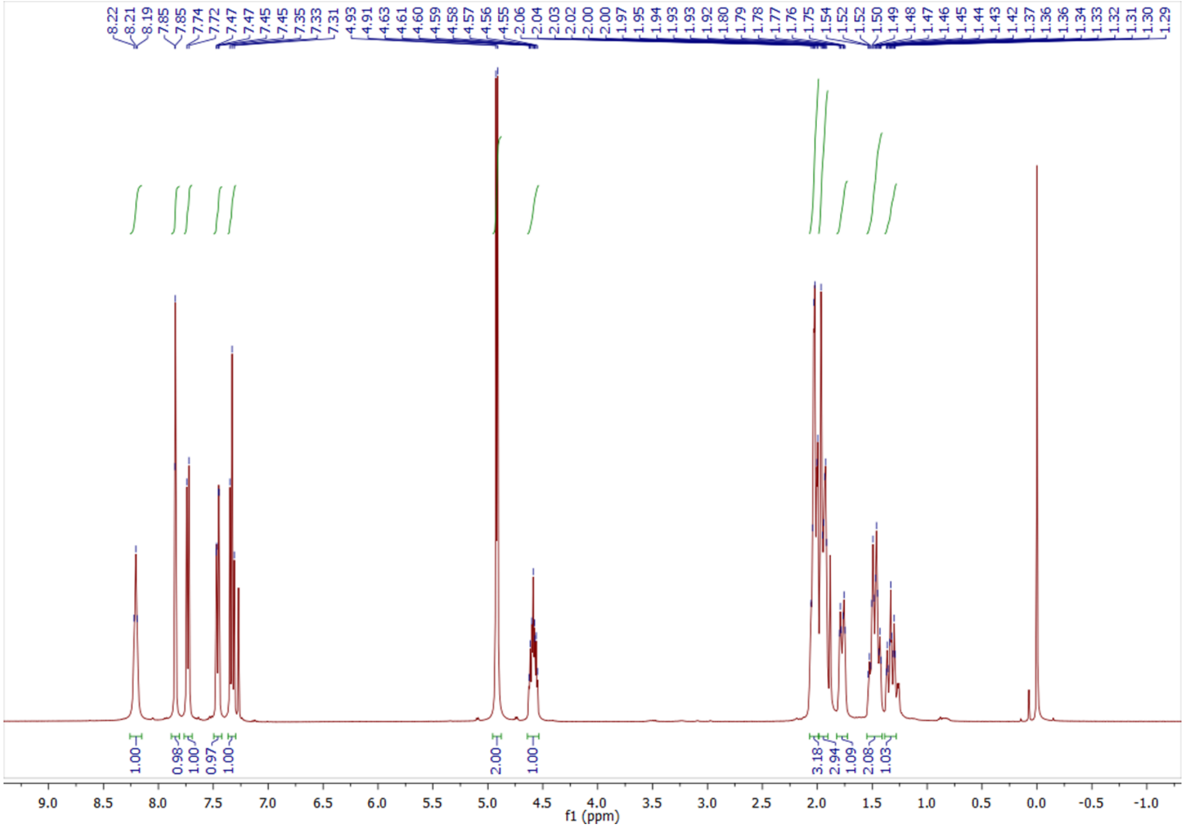

Figure S32. ^{1}H NMR (400 MHz, $CDCl_3$) spectrum of compound **1k**.

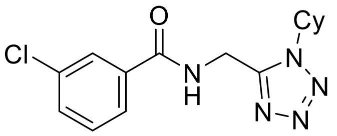

1k

3-chloro-*N*-((1-cyclohexyl-1*H*-tetrazol-5-yl)methyl)benzamide

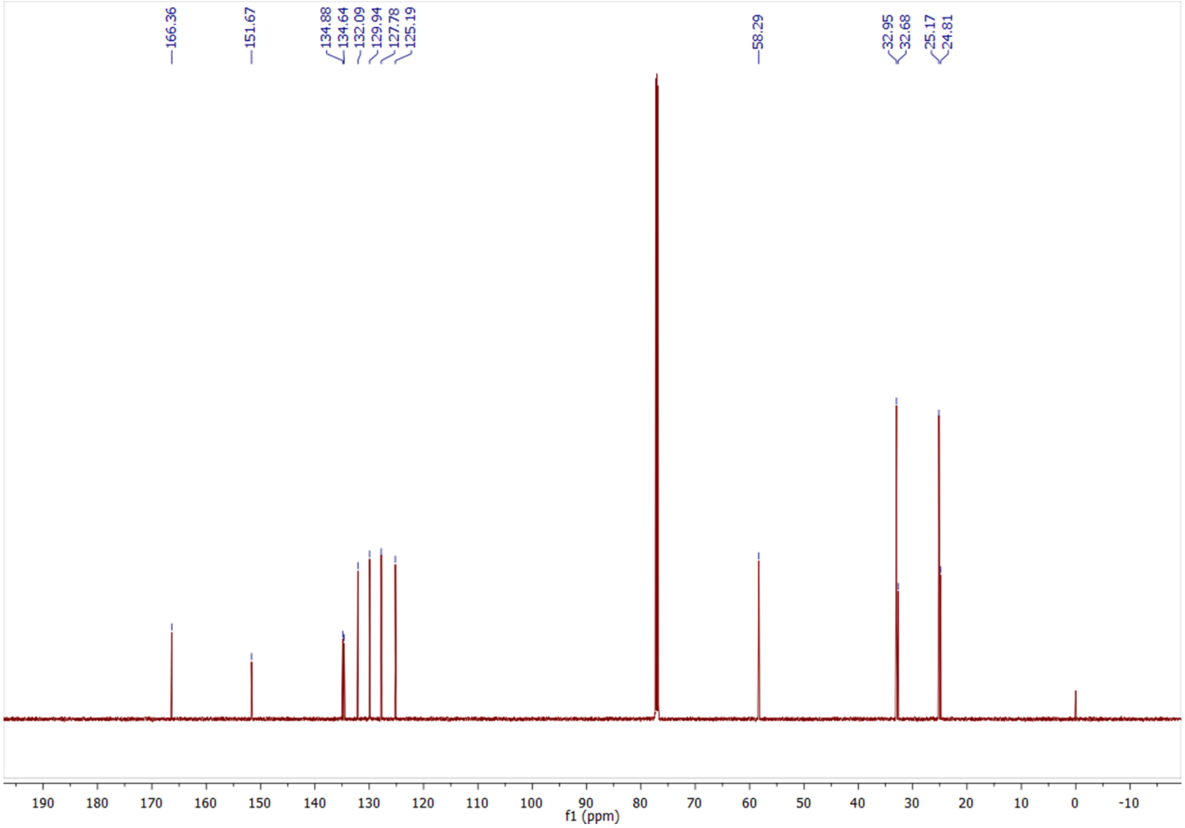

Figure S33. $^{13}C\ \{^1H\}$ NMR (151 MHz, $CDCl_3$) spectrum of compound **1k**.

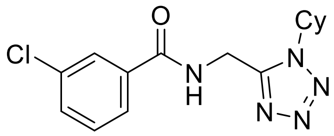

1k

3-chloro-*N*-((1-cyclohexyl-1*H*-tetrazol-5-yl)methyl)benzamide

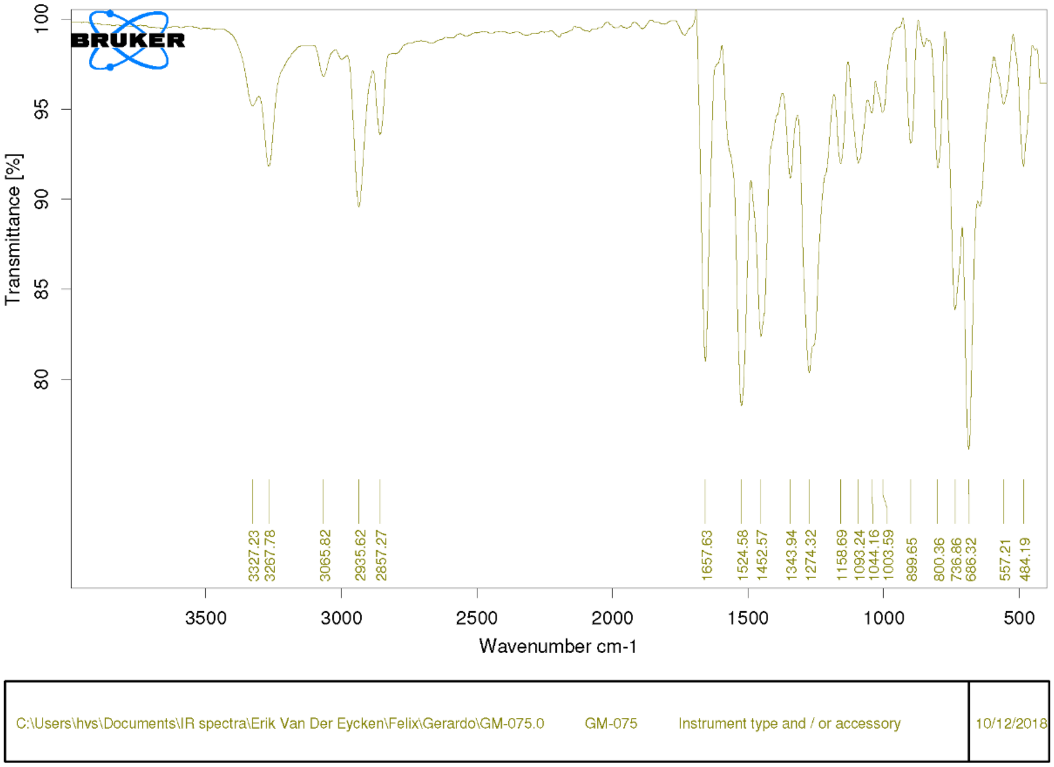

Page 1/1

Figure S34. FT-IR (KBr) spectrum of compound **1k**.

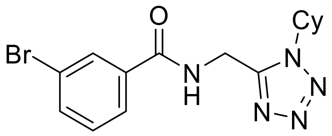

1l

3-bromo-*N*-((1-cyclohexyl-1*H*-tetrazol-5-yl)methyl)benzamide

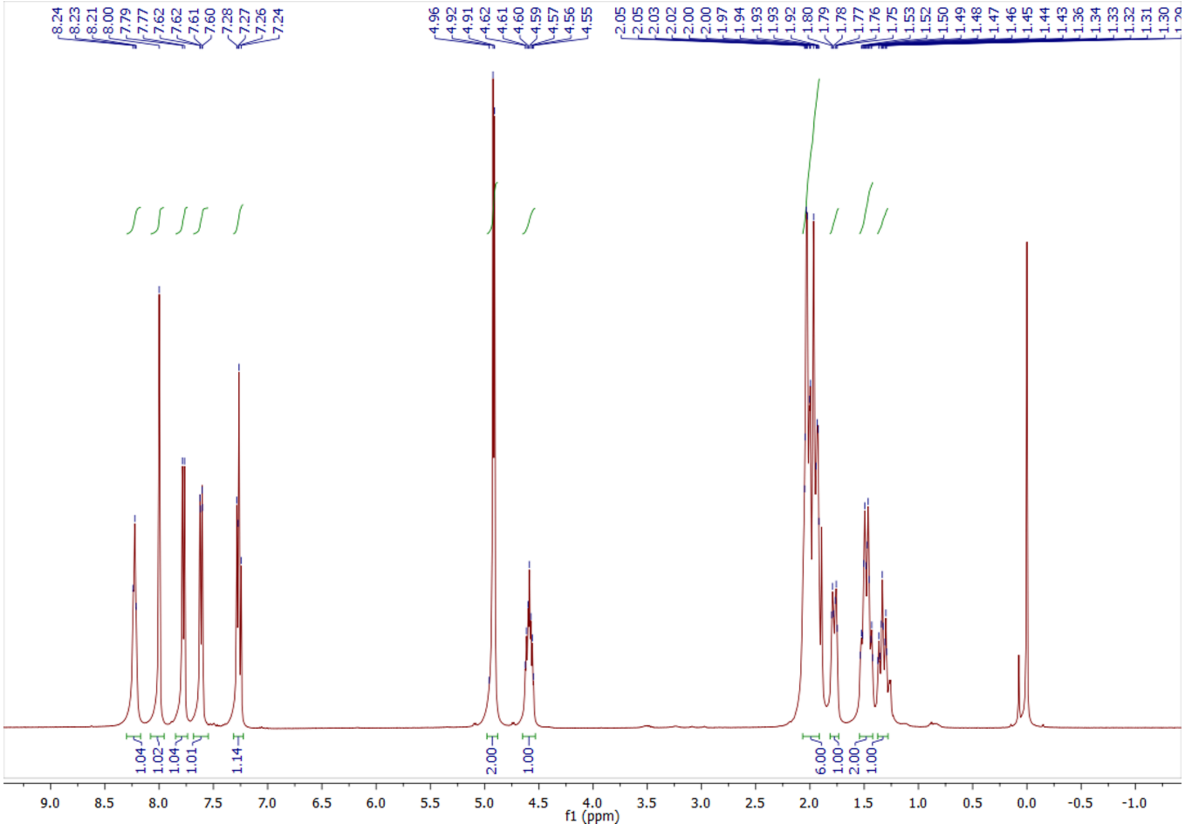

Figure S35. ^{1}H NMR (400 MHz, $CDCl_3$) spectrum of compound **1l**.

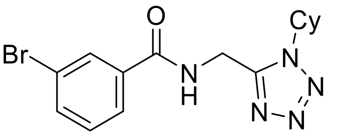

1l

3-bromo-*N*-((1-cyclohexyl-1*H*-tetrazol-5-yl)methyl)benzamide

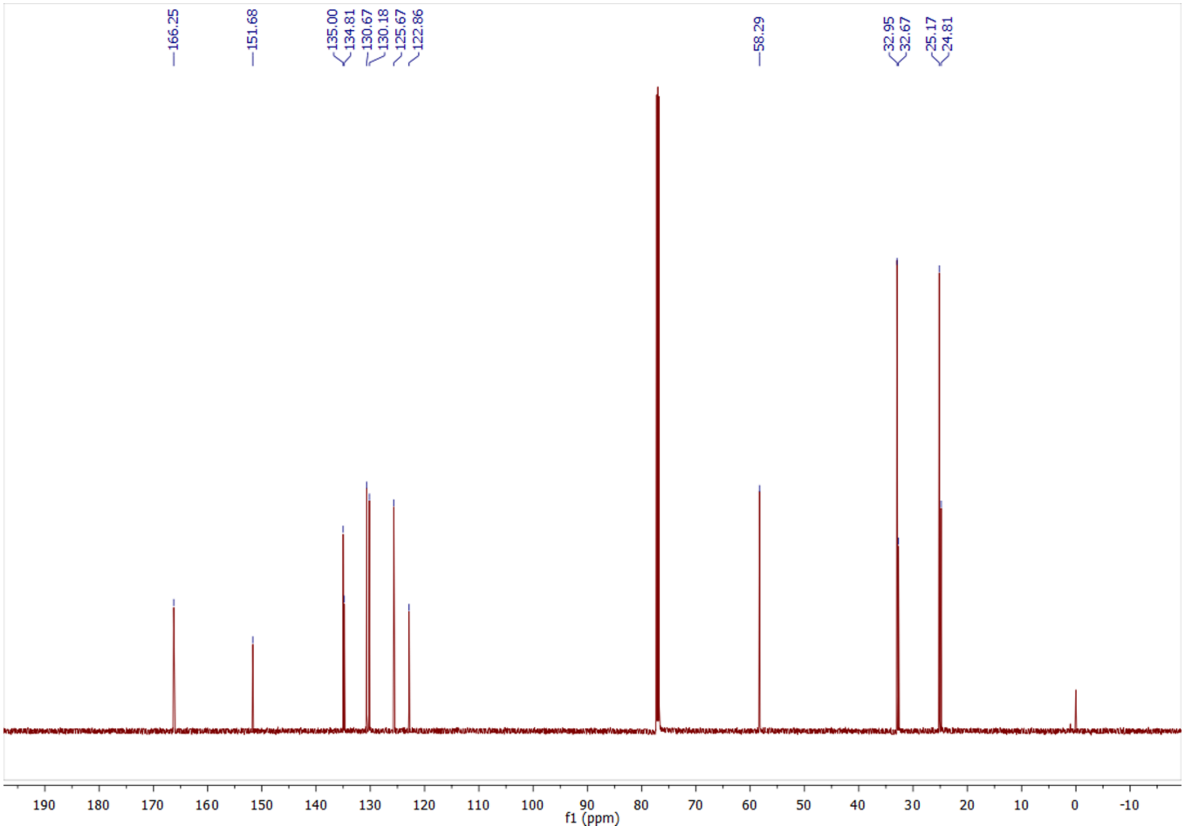

Figure S36. $^{13}C\ \{^1H\}$ NMR (151 MHz, $CDCl_3$) spectrum of compound **1l**.

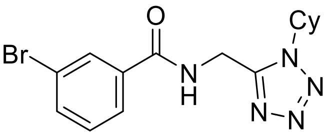

1l

3-bromo-*N*-((1-cyclohexyl-1*H*-tetrazol-5-yl)methyl)benzamide

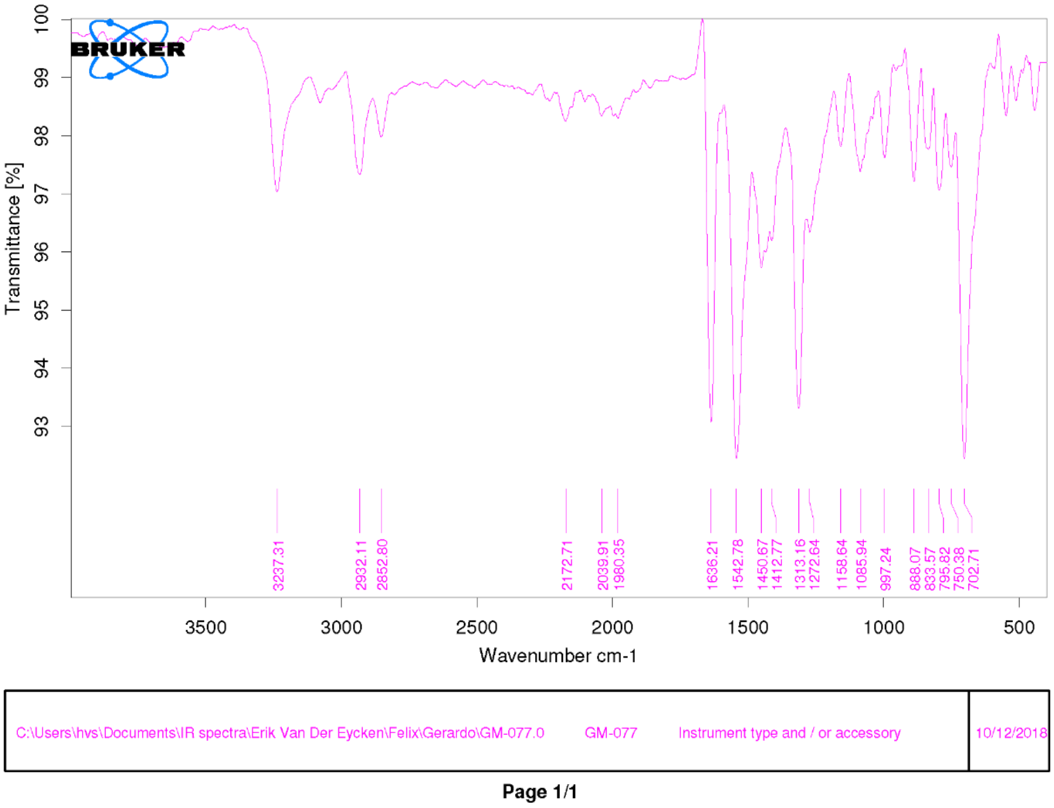

Figure S37. FT-IR (KBr) spectrum of compound **1l**.

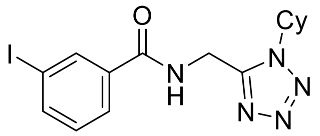

1m

N-((1-cyclohexyl-1*H*-tetrazol-5-yl)methyl)-3-iodobenzamide

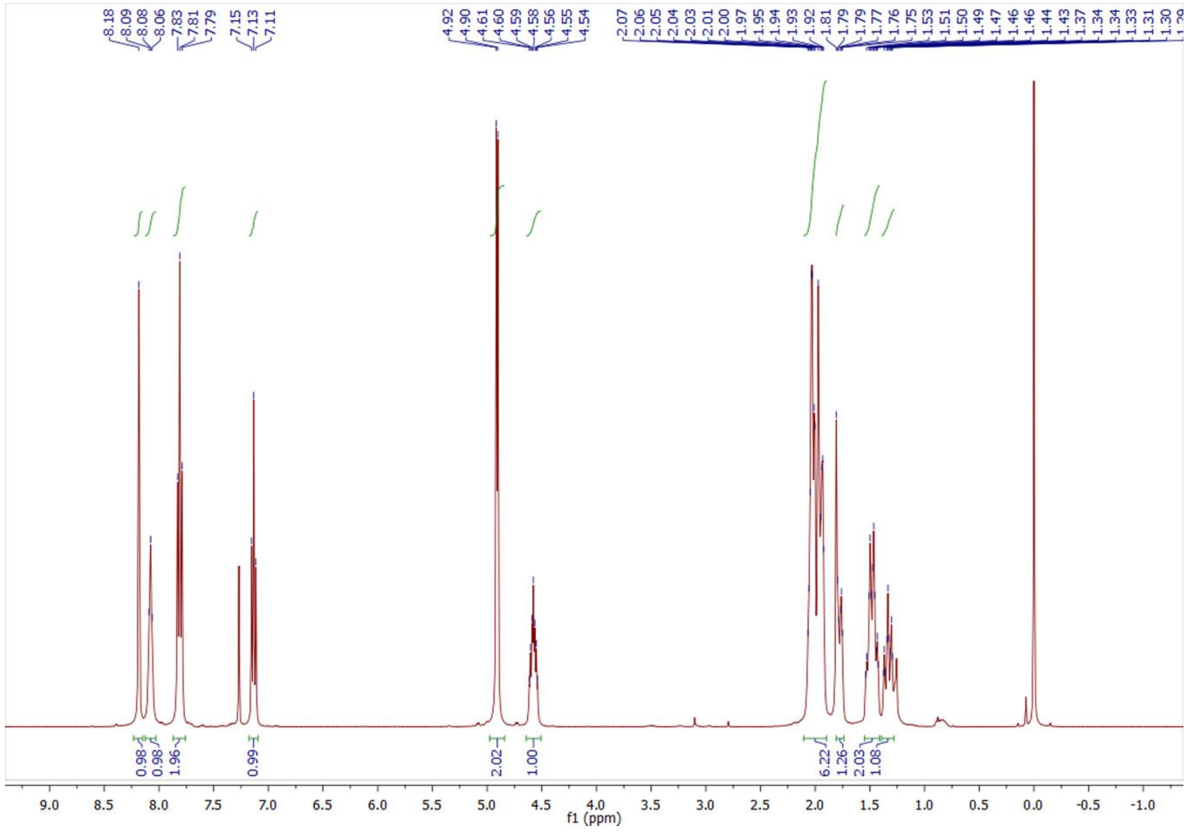

Figure S38. ^{1}H NMR (400 MHz, $CDCl_3$) spectrum of compound **1m**.

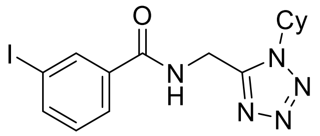

1m

N-((1-cyclohexyl-1*H*-tetrazol-5-yl)methyl)-3-iodobenzamide

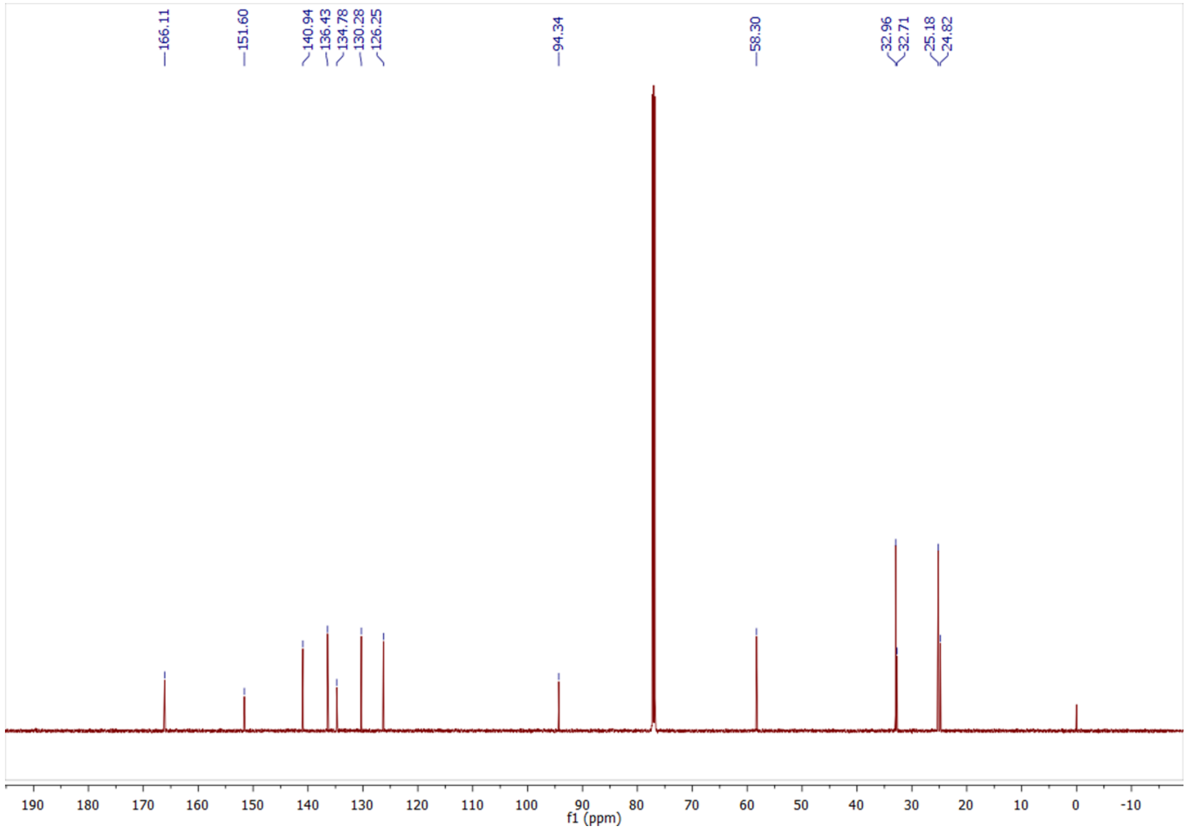

Figure S39. $^{13}C\ \{^{1}H\}$ NMR (151 MHz, $CDCl_3$) spectrum of compound **1m**.

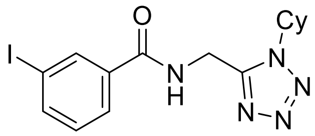

1m

N-((1-cyclohexyl-1*H*-tetrazol-5-yl)methyl)-3-iodobenzamide

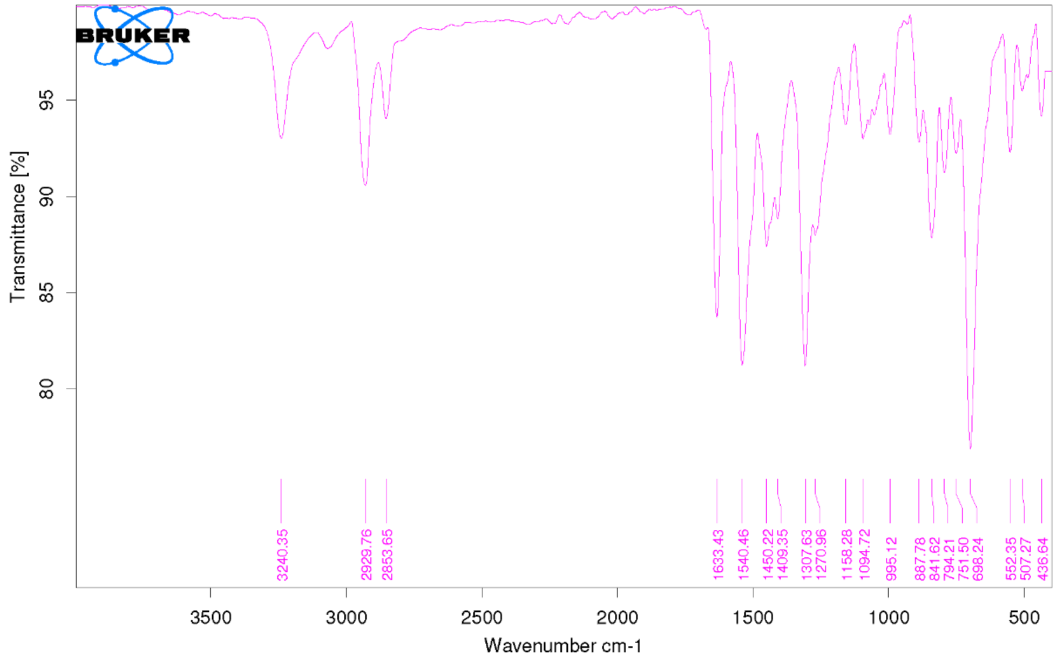

C:\Users\hvs\Documents\IR spectra\Erik Van Der Eycken\Felix\Gerardo\GM-079.0	GM-079	Instrument type and / or accessory	10/12/2018

Page 1/1

Figure S40. FT-IR (KBr) spectrum of compound **1m**.

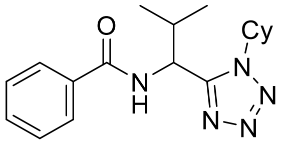

1n

N-(1-(1-cyclohexyl-1*H*-tetrazol-5-yl)-2-methylpropyl)benzamide

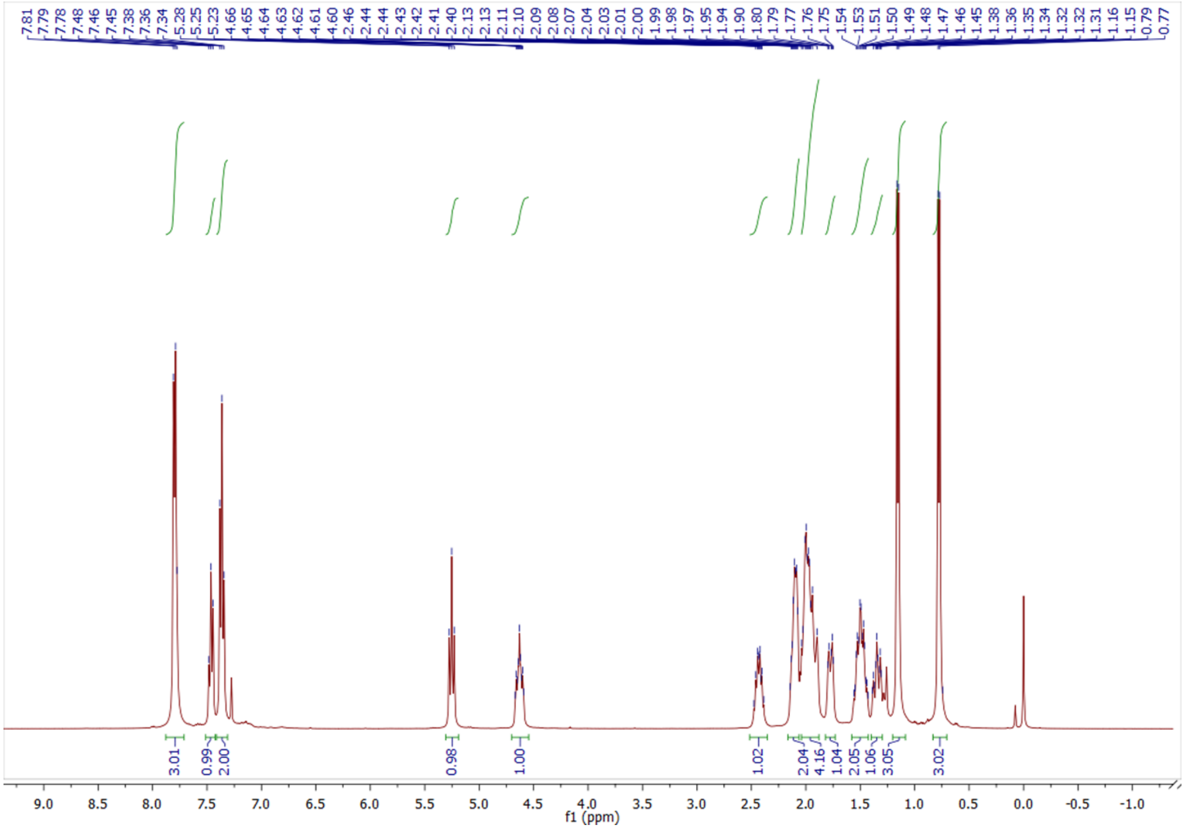

Figure S41. ^{1}H NMR (400 MHz, $CDCl_3$) spectrum of compound **1n**.

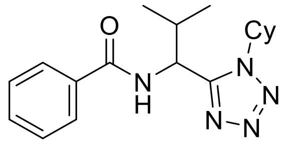

1n

N-(1-(1-cyclohexyl-1*H*-tetrazol-5-yl)-2-methylpropyl)benzamide

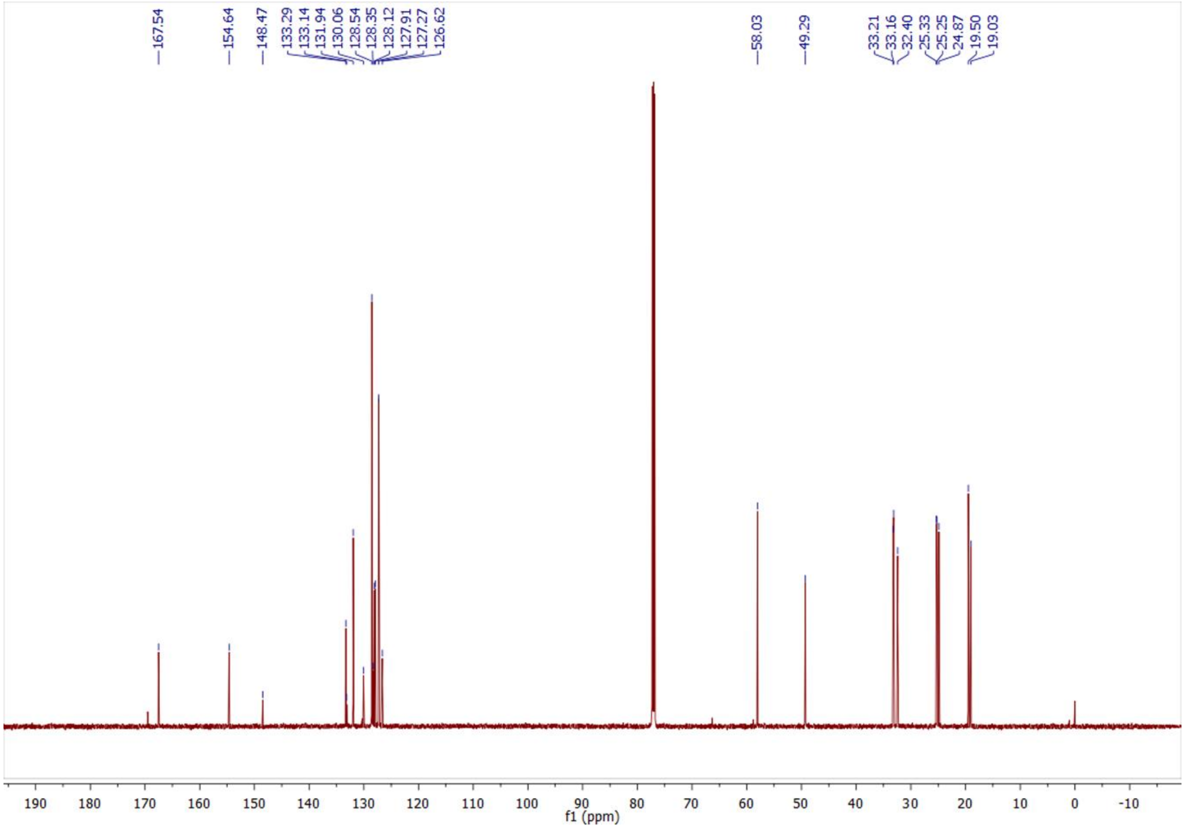

Figure S42. ^{13}C {^{1}H} NMR (151 MHz, $CDCl_3$) spectrum of compound **1n**.

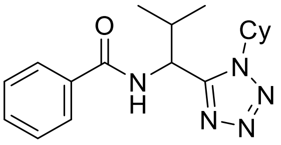

1n

N-(1-(1-cyclohexyl-1*H*-tetrazol-5-yl)-2-methylpropyl)benzamide

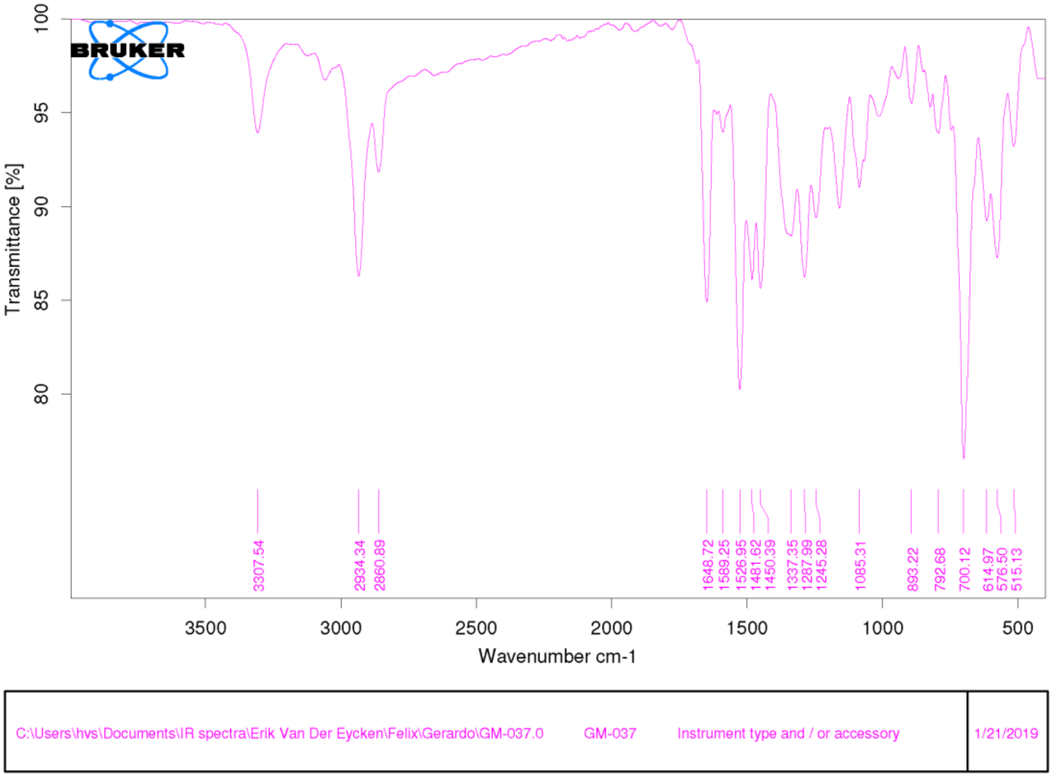

Page 1/1

Figure S43. FT-IR (KBr) spectrum of compound **1n**.

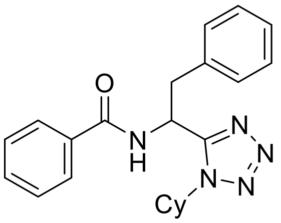

1o

N-(1-(1-cyclohexyl-1*H*-tetrazol-5-yl)-2-phenylethyl)benzamide

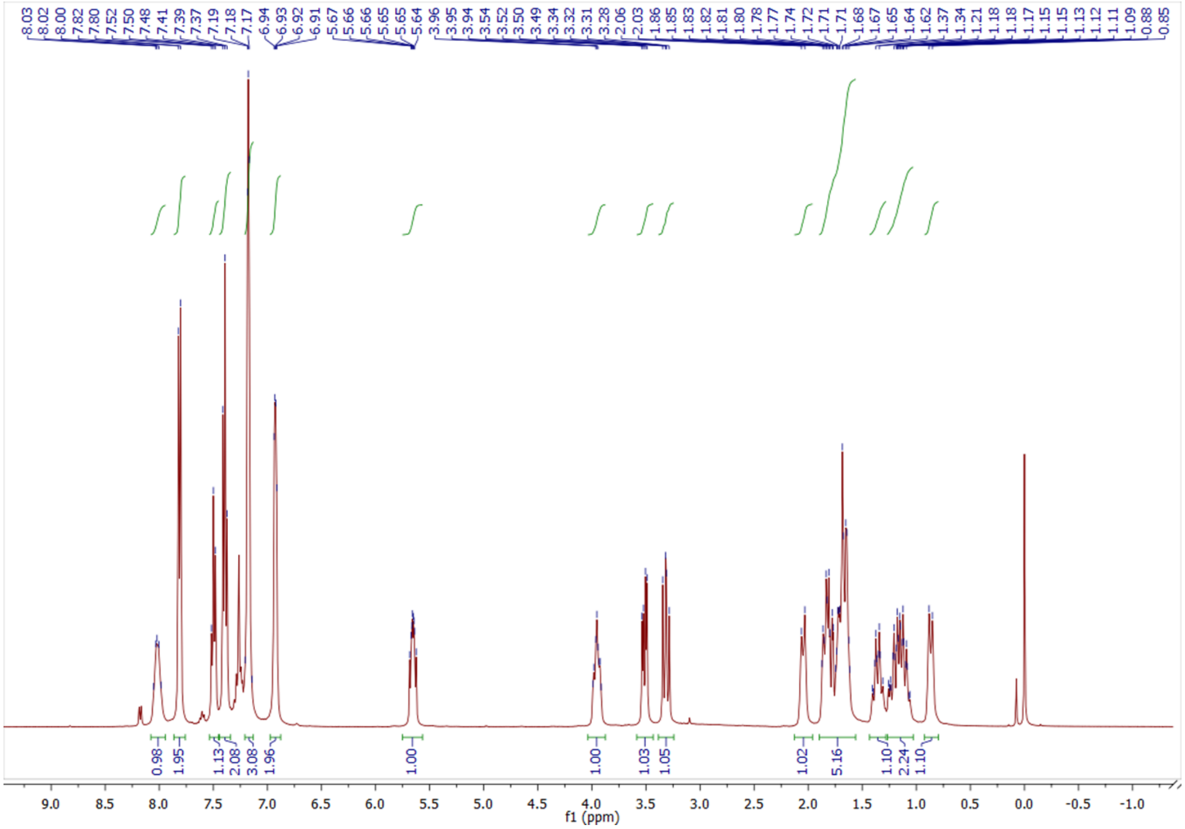

Figure S44. ^{1}H NMR (400 MHz, $CDCl_3$) spectrum of compound **1o**.

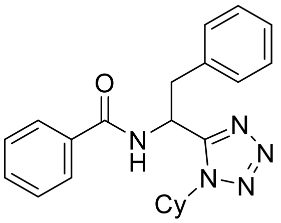

1o

N-(1-(1-cyclohexyl-1*H*-tetrazol-5-yl)-2-phenylethyl)benzamide

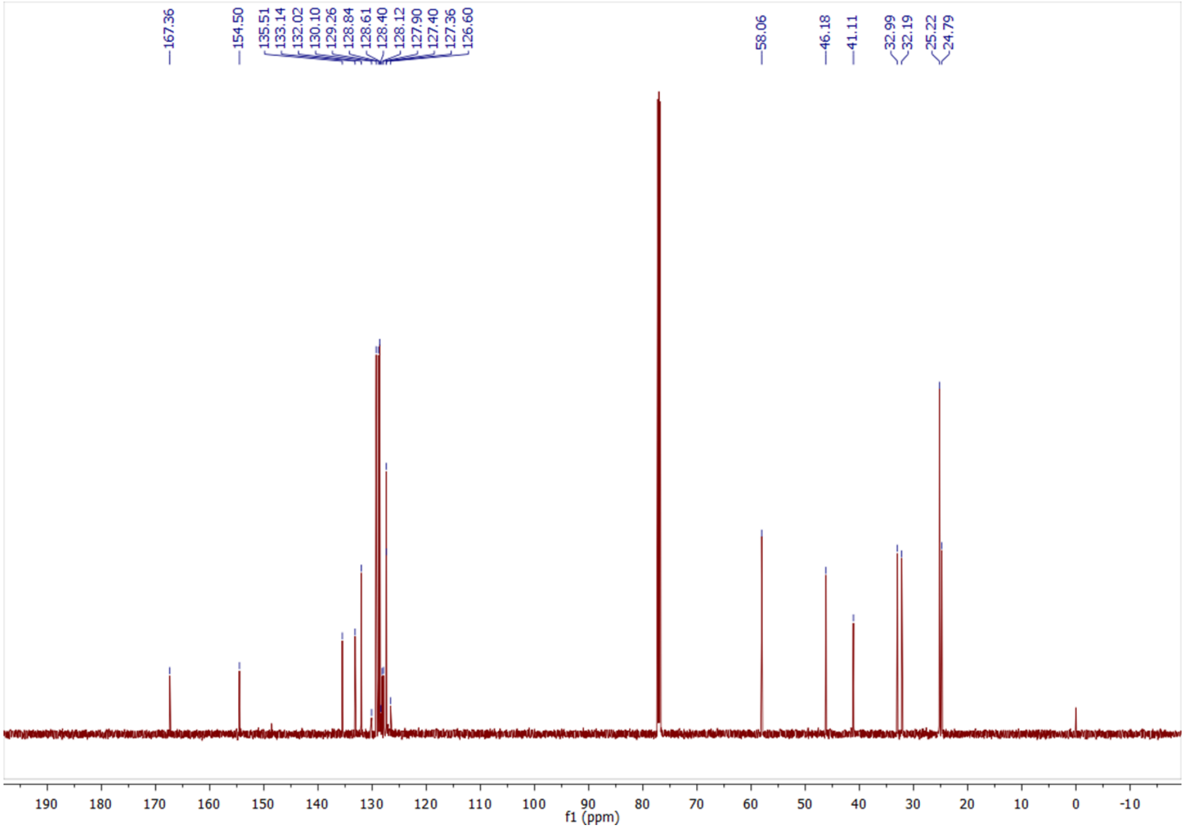

Figure S45. $^{13}C\ \{^1H\}$ NMR (151 MHz, $CDCl_3$) spectrum of compound **1o**.

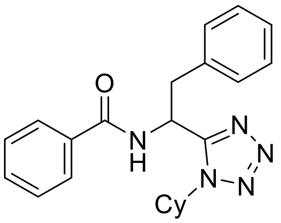

1o

N-(1-(1-cyclohexyl-1*H*-tetrazol-5-yl)-2-phenylethyl)benzamide

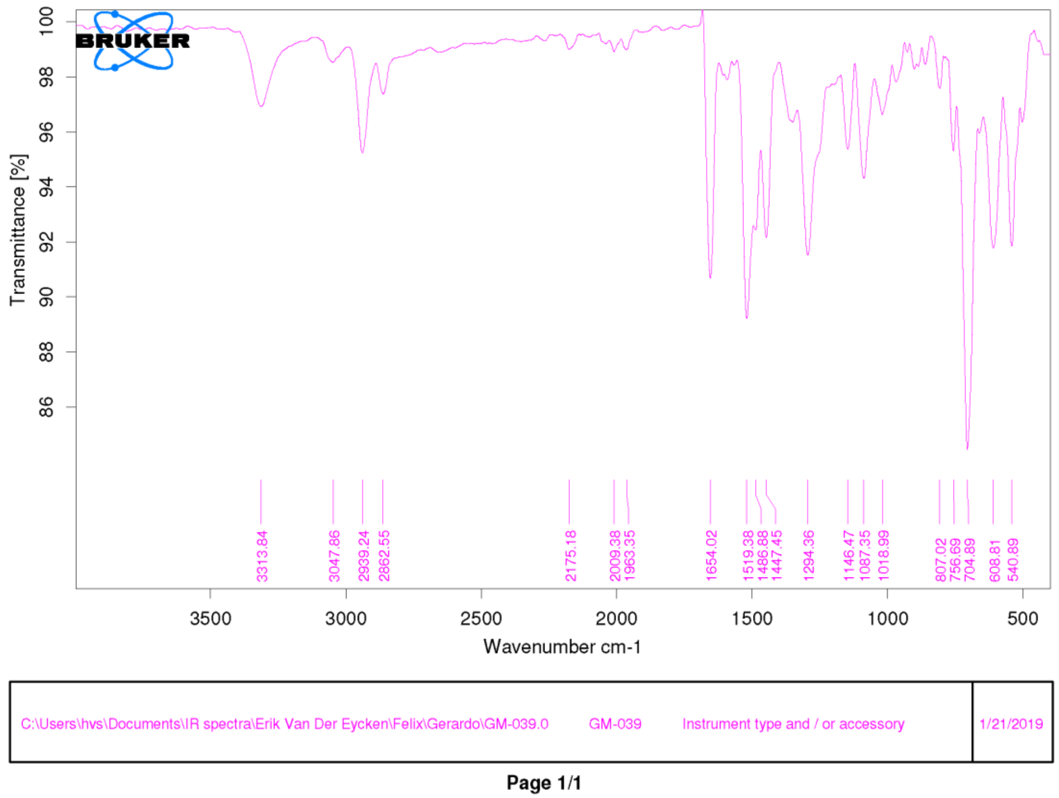

Figure S46. FT-IR (KBr) spectrum of compound **1o**.

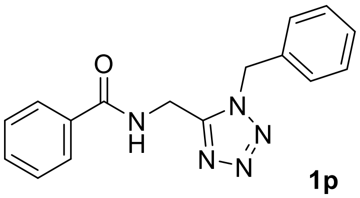

1p

N-((1-benzyl-1*H*-tetrazol-5-yl)methyl)benzamide

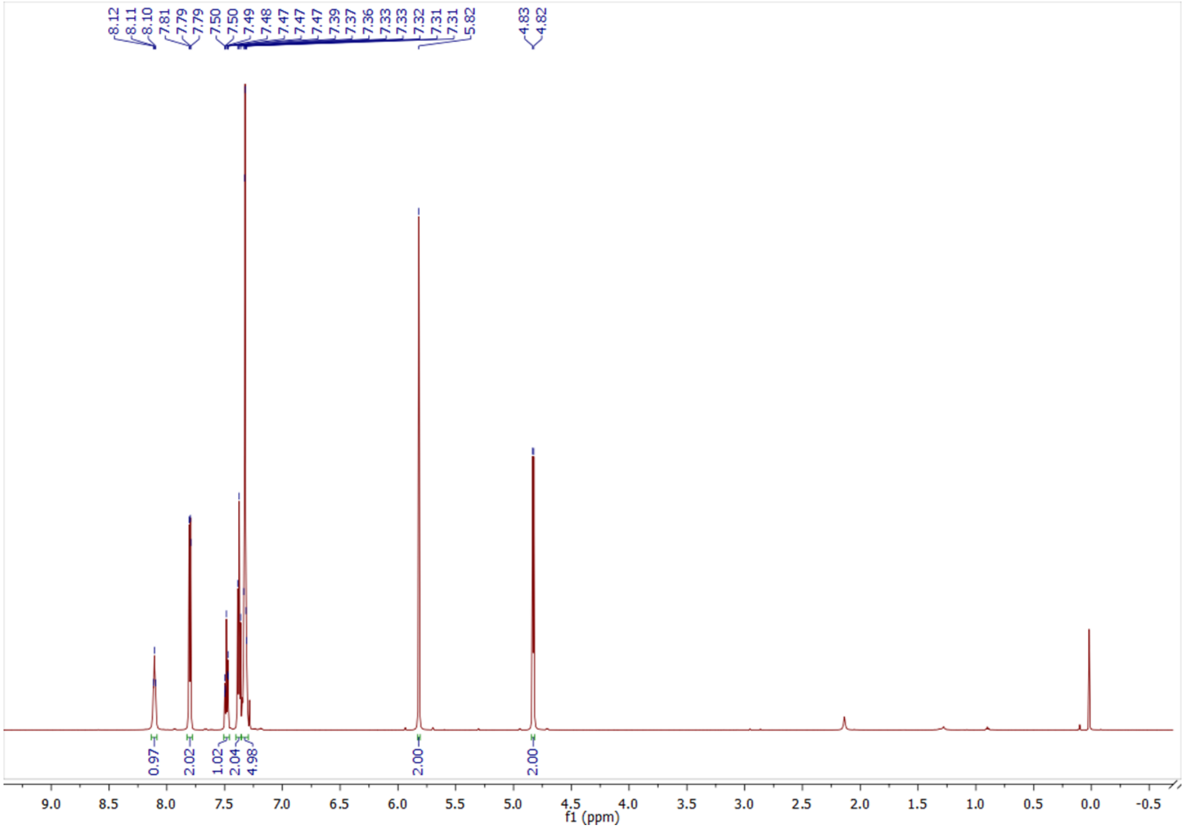

Figure S47. ^{1}H NMR (600 MHz, $CDCl_3$) spectrum of compound **1p**.

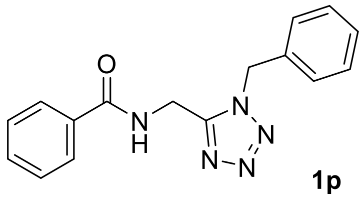

1p

N-((1-benzyl-1*H*-tetrazol-5-yl)methyl)benzamide

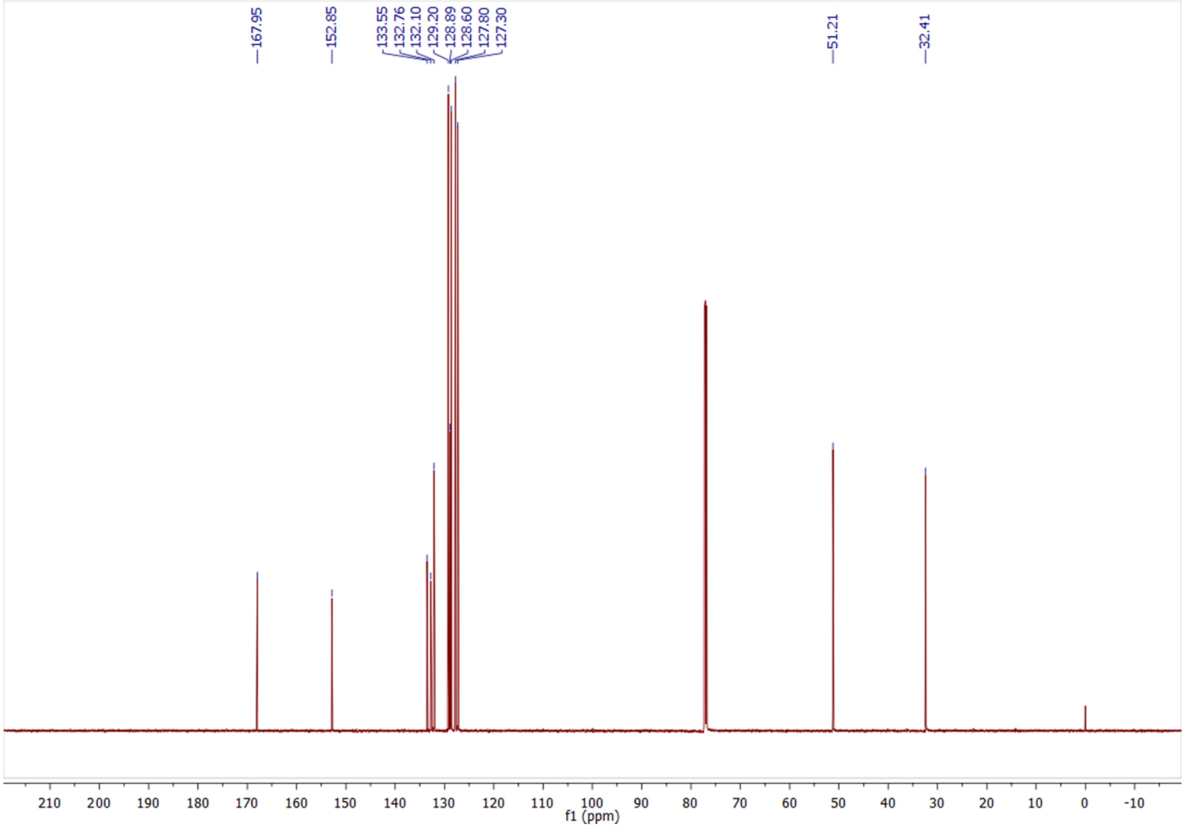

Figure S48. ^{13}C {^{1}H} NMR (151 MHz, $CDCl_3$) spectrum of compound **1p**.

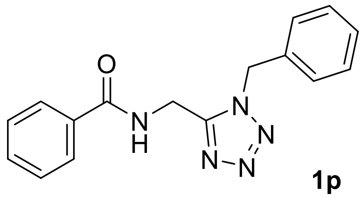

1p

N-((1-benzyl-1*H*-tetrazol-5-yl)methyl)benzamide

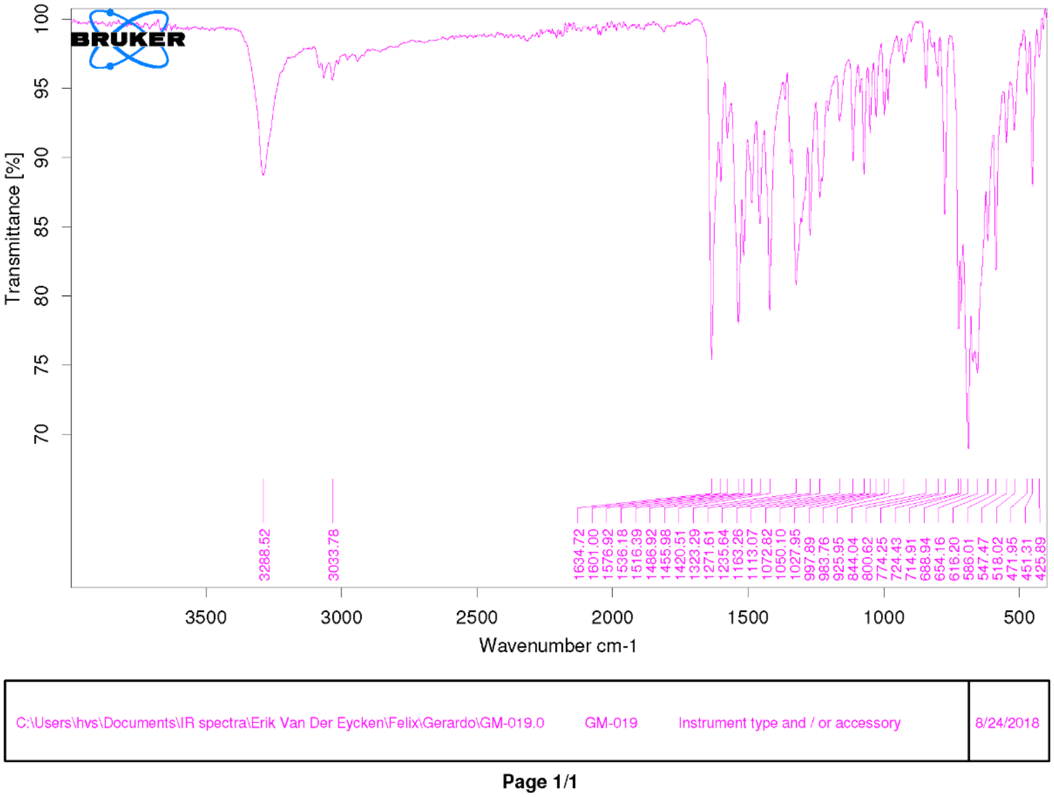

Figure S49. FT-IR (KBr) spectrum of compound **1p**.

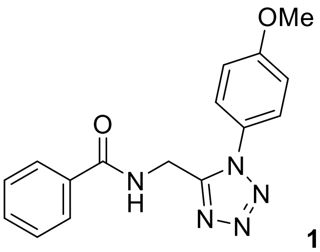

1q

N-((1-(4-methoxyphenyl)-1*H*-tetrazol-5-yl)methyl)benzamide

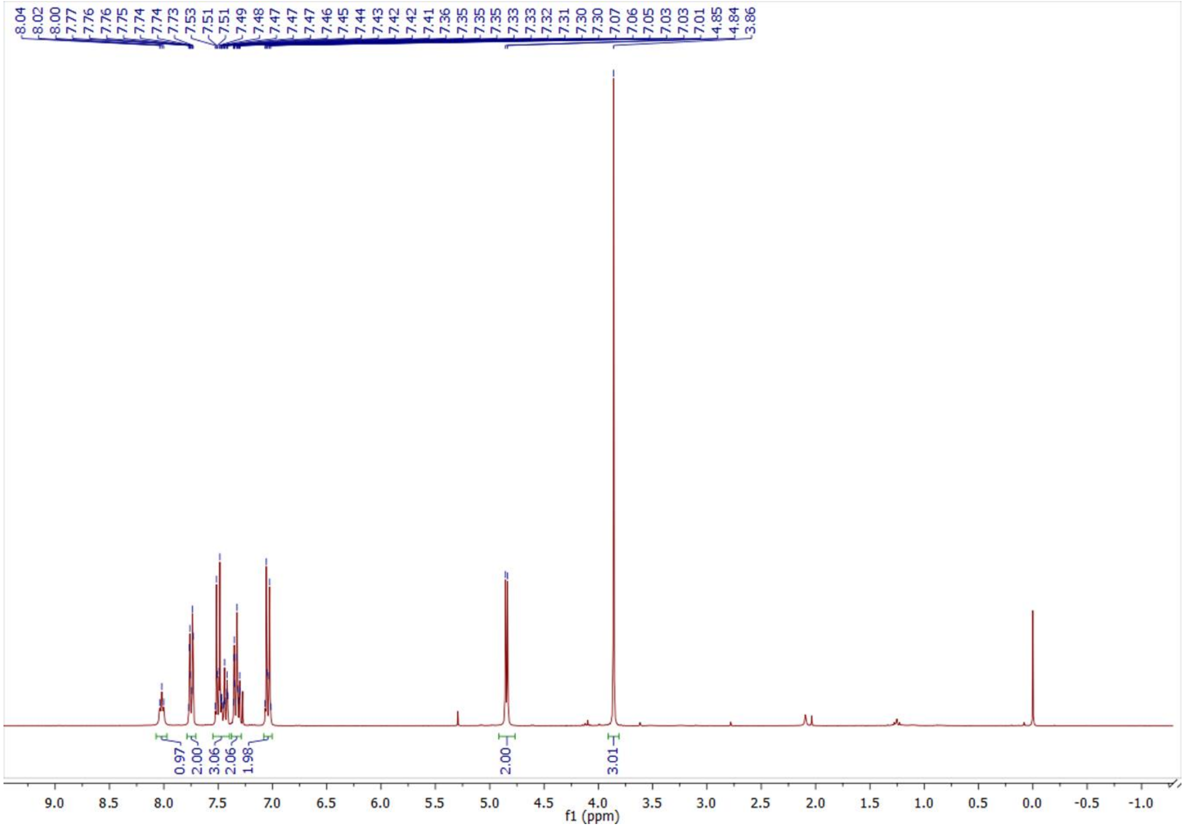

Figure S50. ^{1}H NMR (300 MHz, $CDCl_3$) spectrum of compound **1q**.

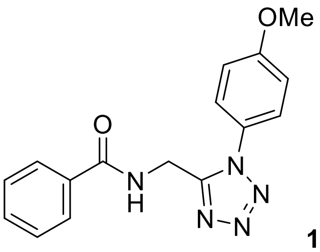

1q

N-((1-(4-methoxyphenyl)-1*H*-tetrazol-5-yl)methyl)benzamide

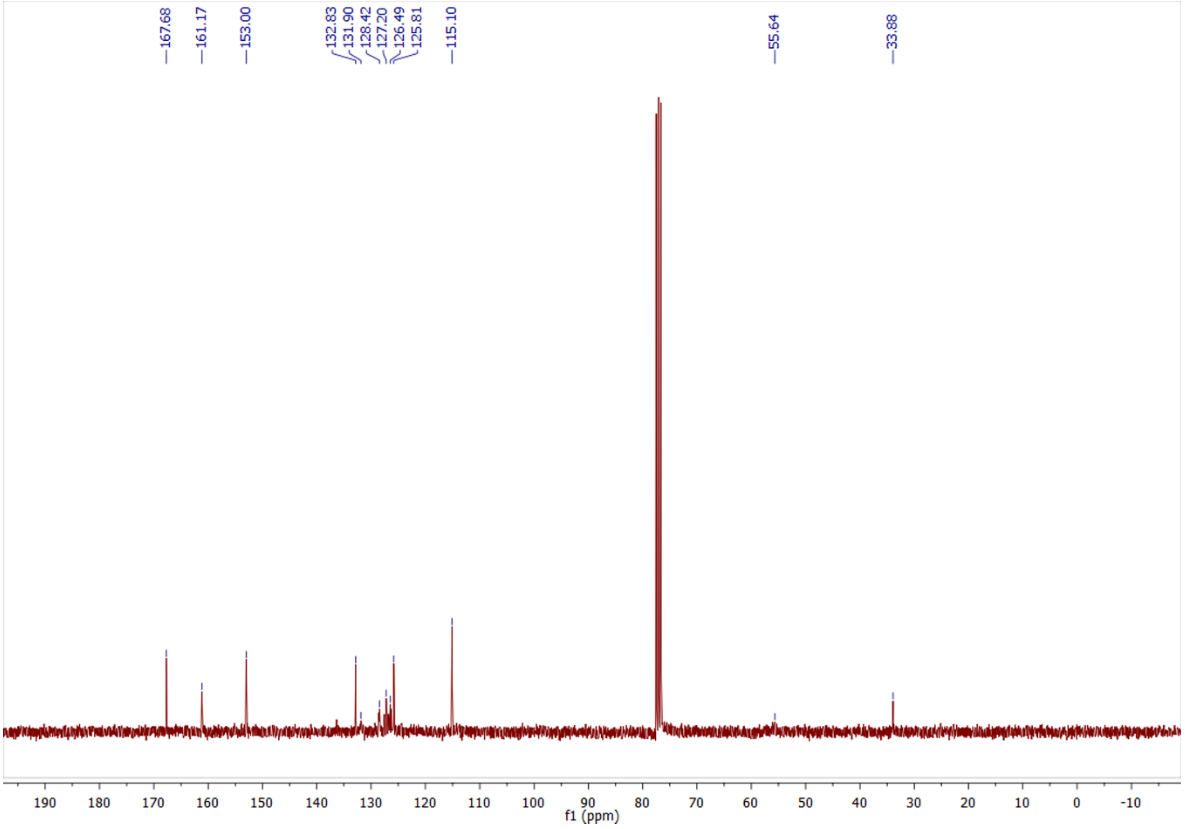

Figure S51. $^{13}C\ \{^{1}H\}$ NMR (75 MHz, $CDCl_3$) spectrum of compound **1q**.

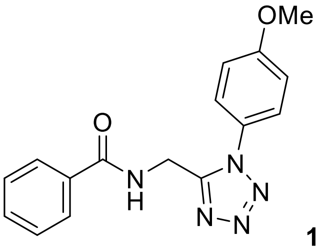

1q

N-((1-(4-methoxyphenyl)-1*H*-tetrazol-5-yl)methyl)benzamide

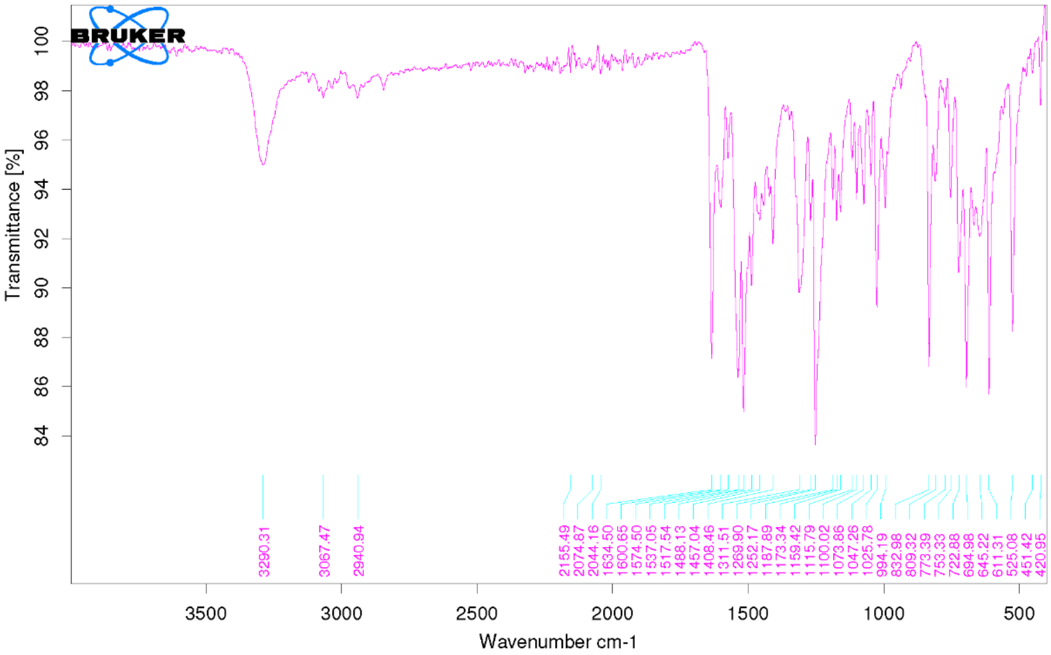

C:\Users\hvs\Documents\IR spectra\Erik Van Der Eycken\Felix\Gerardo\GM-21.0	GM-21	Instrument type and / or accessory	8/24/2018

Page 1/1

Figure S52. FT-IR (KBr) spectrum of compound **1q**.

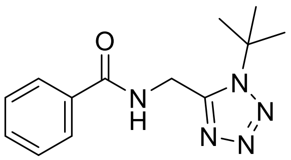

1r

N-((1-(*tert*-butyl)-1*H*-tetrazol-5-yl)methyl)benzamide

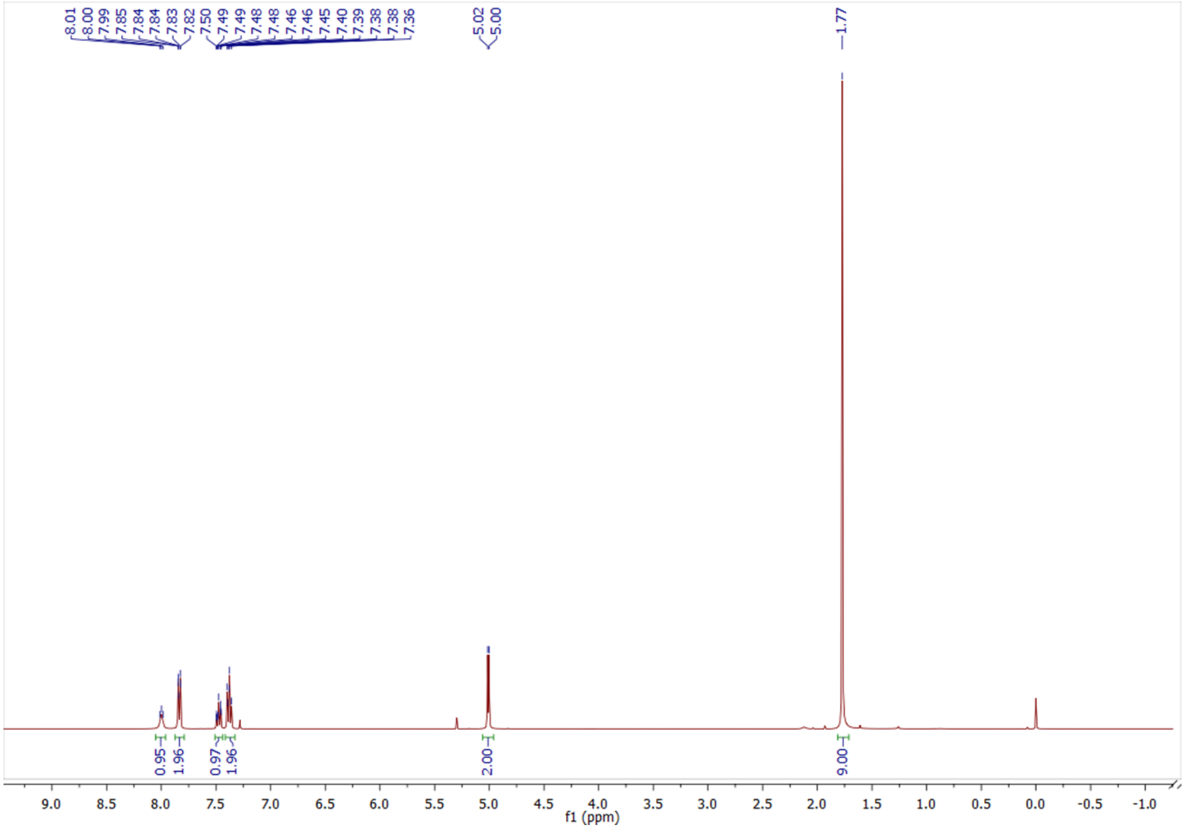

Figure S52. ^{1}H NMR (400 MHz, $CDCl_3$) spectrum of compound **1r**.

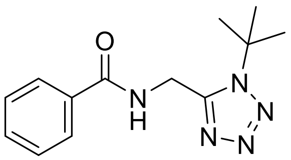

1r

N-((1-(*tert*-butyl)-1*H*-tetrazol-5-yl)methyl)benzamide

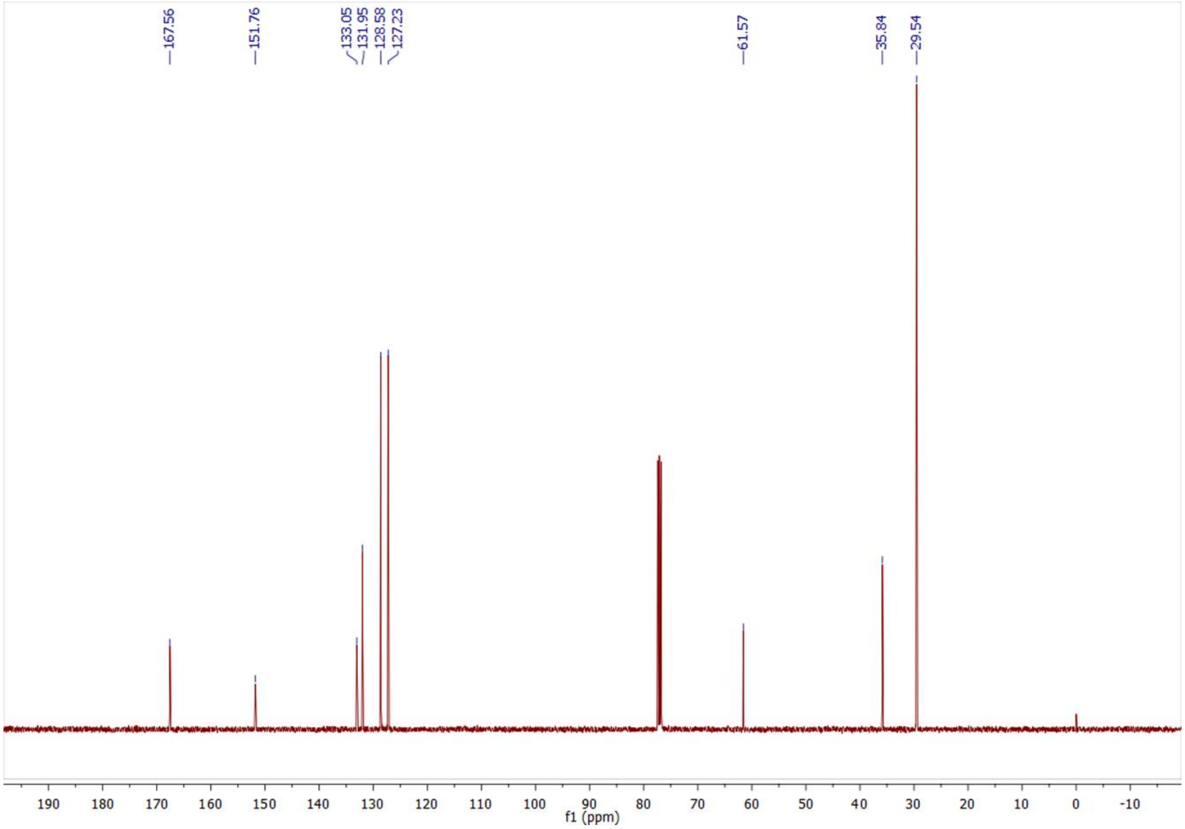

Figure S54. $^{13}C\ \{^{1}H\}$ NMR (101 MHz, $CDCl_3$) spectrum of compound **1r**.

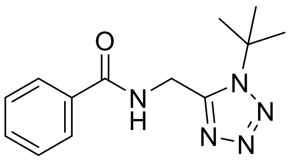

1r

N-((1-(*tert*-butyl)-1*H*-tetrazol-5-yl)methyl)benzamide

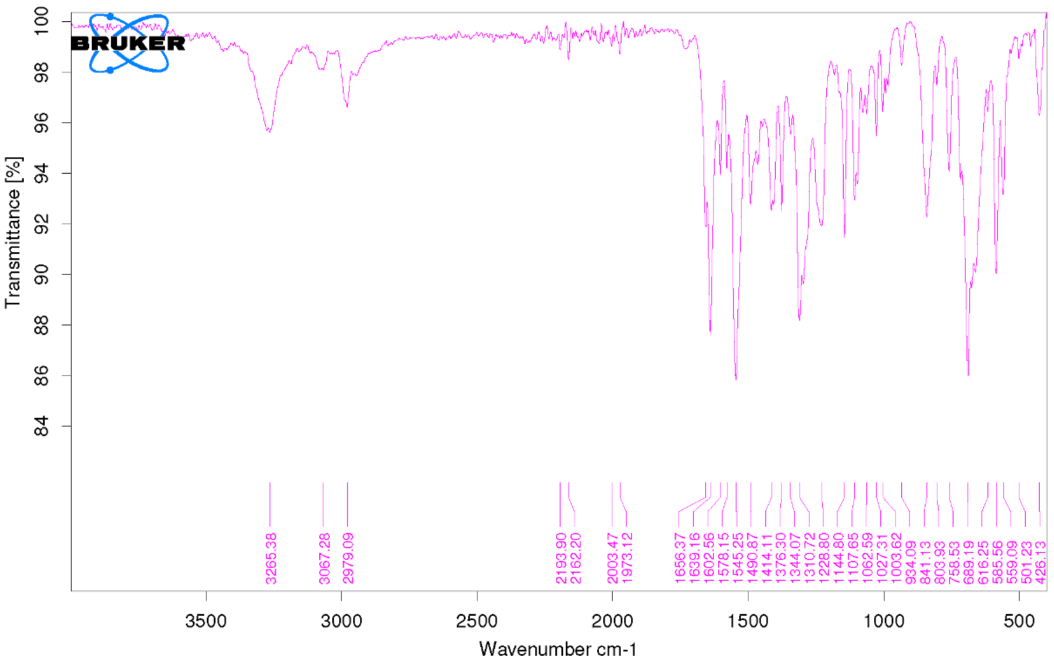

C:\Users\hvs\Documents\IR spectra\Erik Van Der Eycken\Felix\Gerardo\GM-017.0 GM-017 Instrument type and / or accessory 8/24/2018

Page 1/1

Figure S55. FT-IR (KBr) spectrum of compound **1r**.

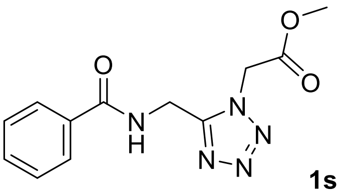

1s

methyl 2-(5-(benzamidomethyl)-1*H*-tetrazol-1-yl)acetate

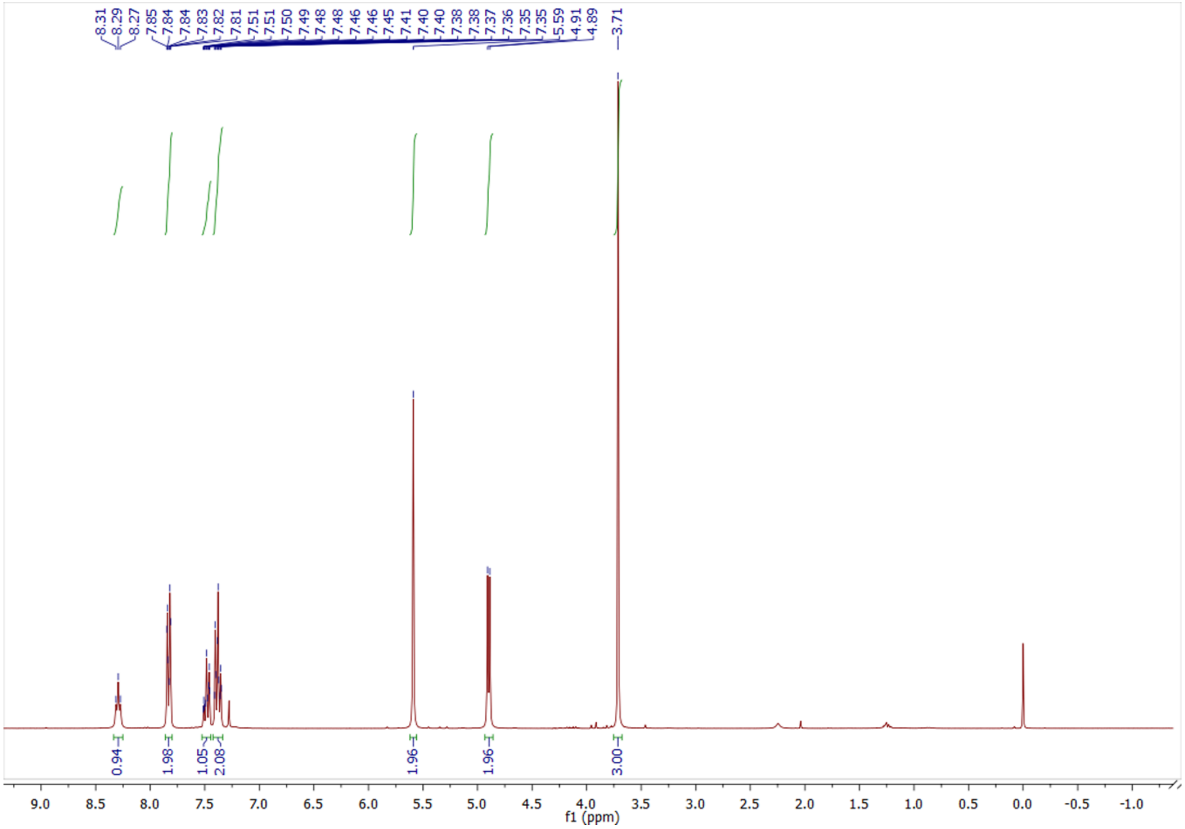

Figure S56. ^{1}H NMR (300 MHz, $CDCl_3$) spectrum of compound **1s**.

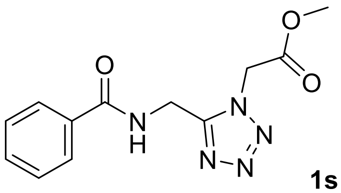

1s

methyl 2-(5-(benzamidomethyl)-1*H*-tetrazol-1-yl)acetate

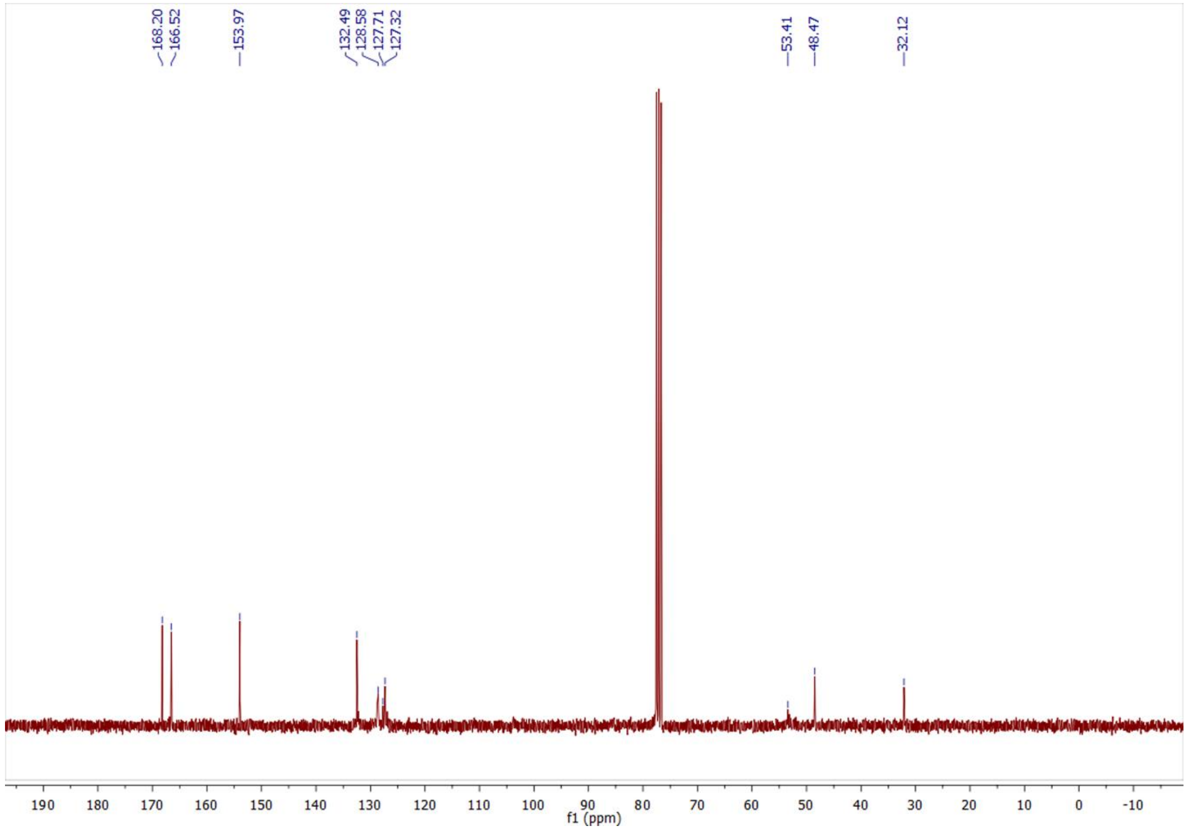

Figure S57. ^{13}C {^{1}H} NMR (75 MHz, $CDCl_3$) spectrum of compound **1s**.

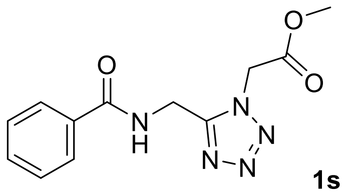

1s

methyl 2-(5-(benzamidomethyl)-1*H*-tetrazol-1-yl)acetate

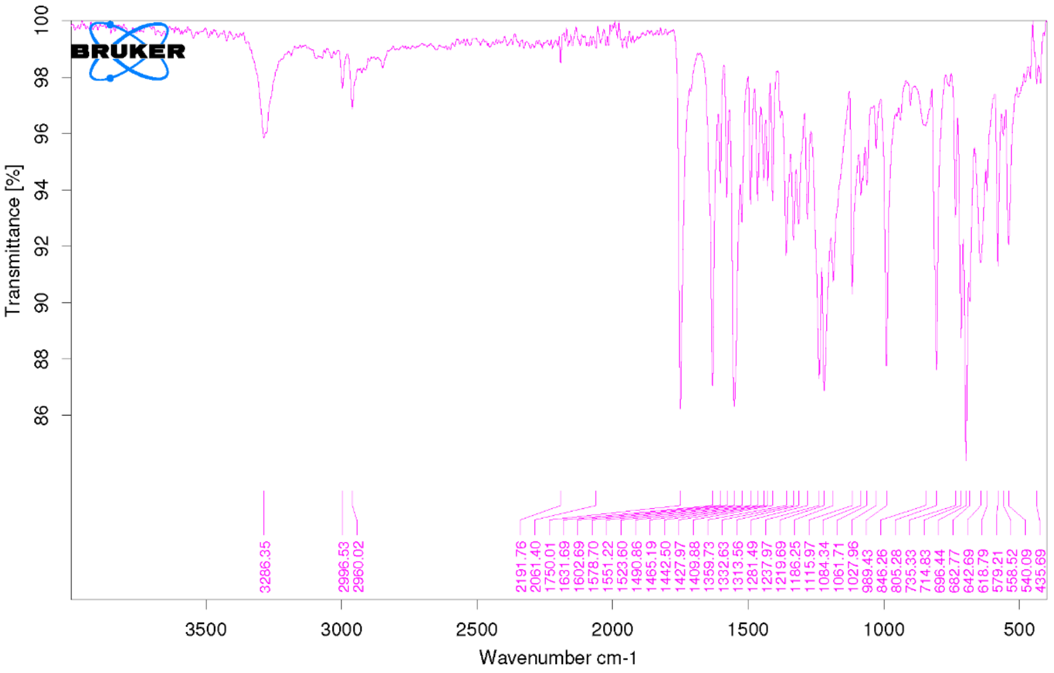

C:\Users\hvs\Documents\IR spectra\Erik Van Der Eycken\Felix\Gerardo\GM-23.0	GM-23	Instrument type and / or accessory		8/24/2018

Page 1/1

Figure S58. FT-IR (KBr) spectrum of compound **1s**.

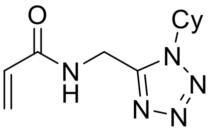

2a

N-((1-cyclohexyl-1*H*-tetrazol-5-yl)methyl)acrylamide

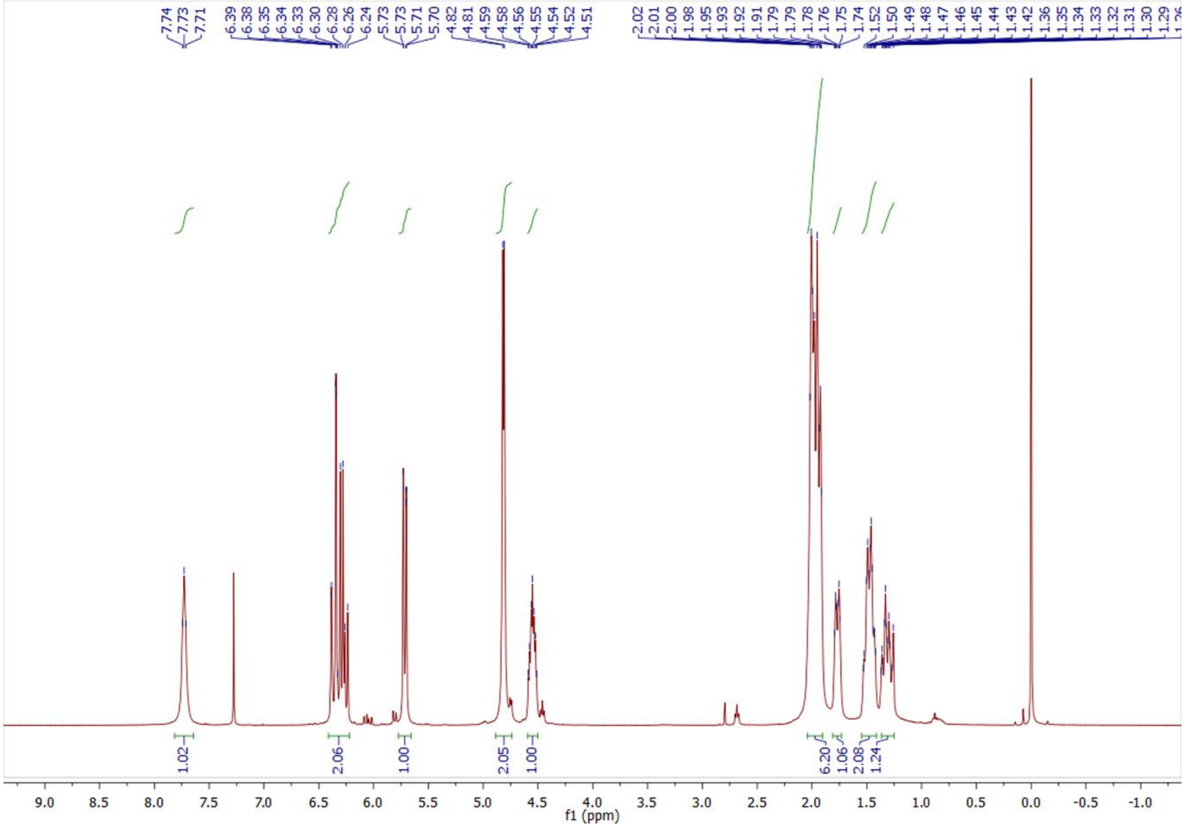

Figure S59. ^{1}H NMR (400 MHz, $CDCl_3$) spectrum of compound **2a**.

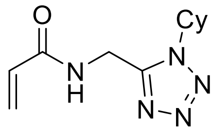

2a

N-((1-cyclohexyl-1*H*-tetrazol-5-yl)methyl)acrylamide

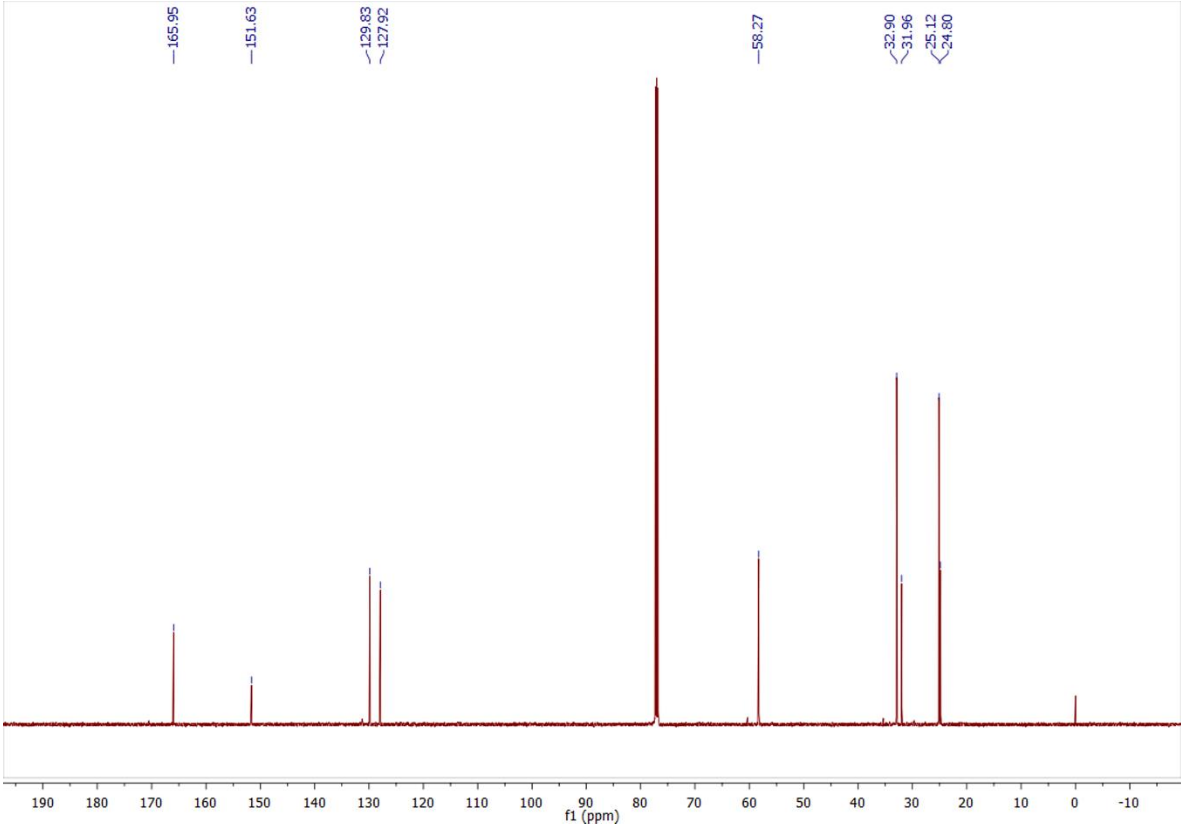

Figure S60. ^{13}C {^{1}H} NMR (151 MHz, $CDCl_3$) spectrum of compound **2a**.

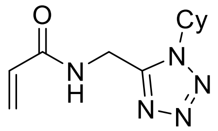

2a

N-((1-cyclohexyl-1*H*-tetrazol-5-yl)methyl)acrylamide

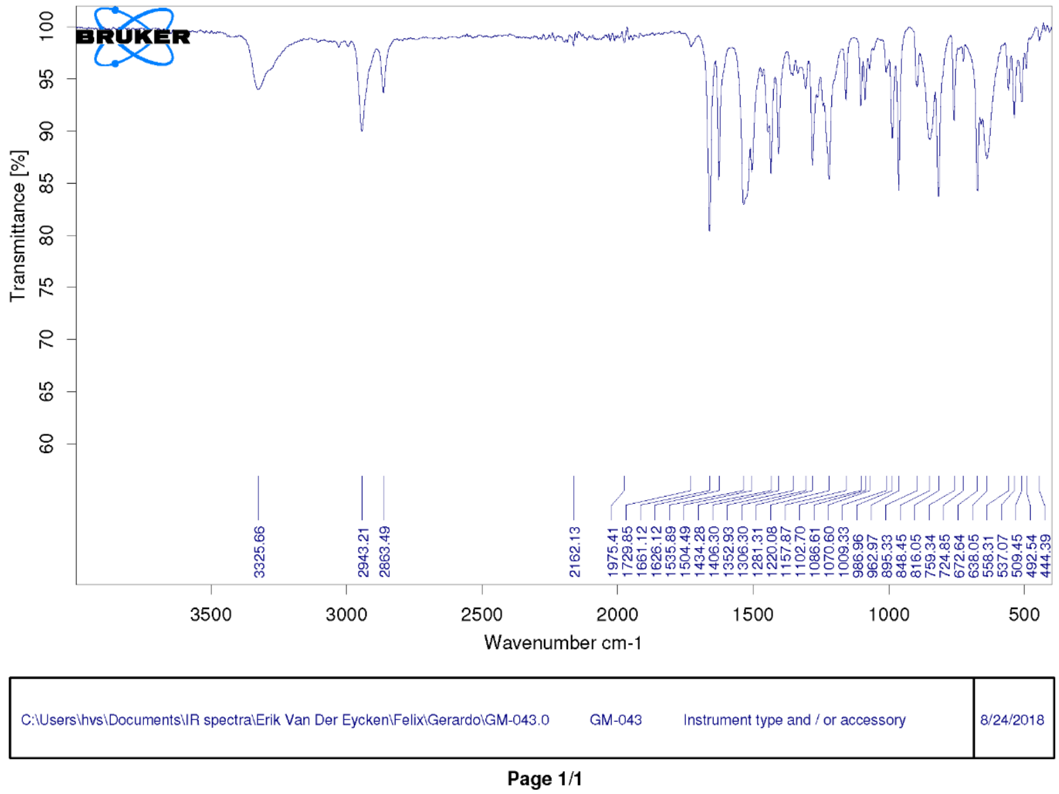

Figure S61. FT-IR (KBr) spectrum of compound **2a**.

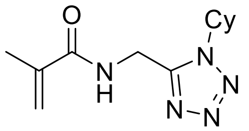

2b

N-((1-cyclohexyl-1*H*-tetrazol-5-yl)methyl)methacrylamide

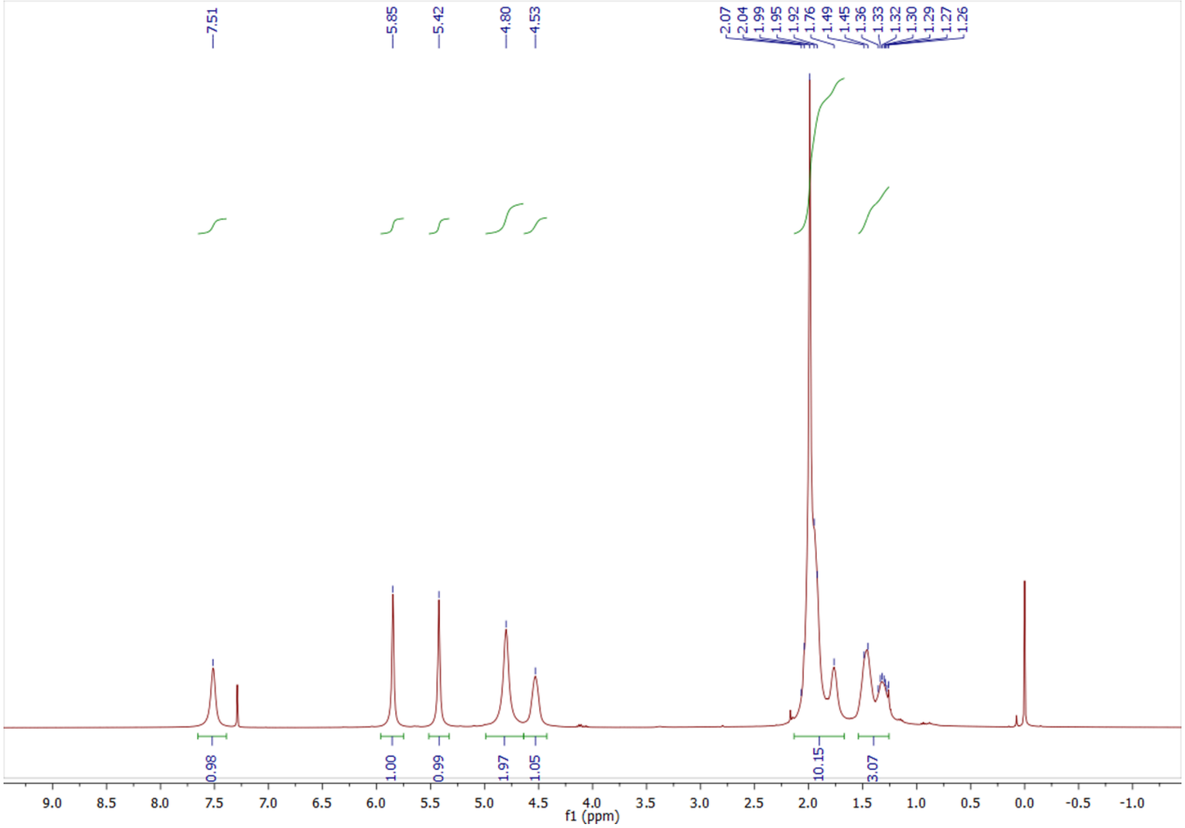

Figure S62. ^{1}H NMR (400 MHz, $CDCl_3$) spectrum of compound **2b**.

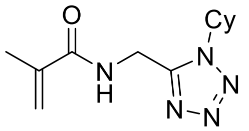

2b

N-((1-cyclohexyl-1*H*-tetrazol-5-yl)methyl)methacrylamide

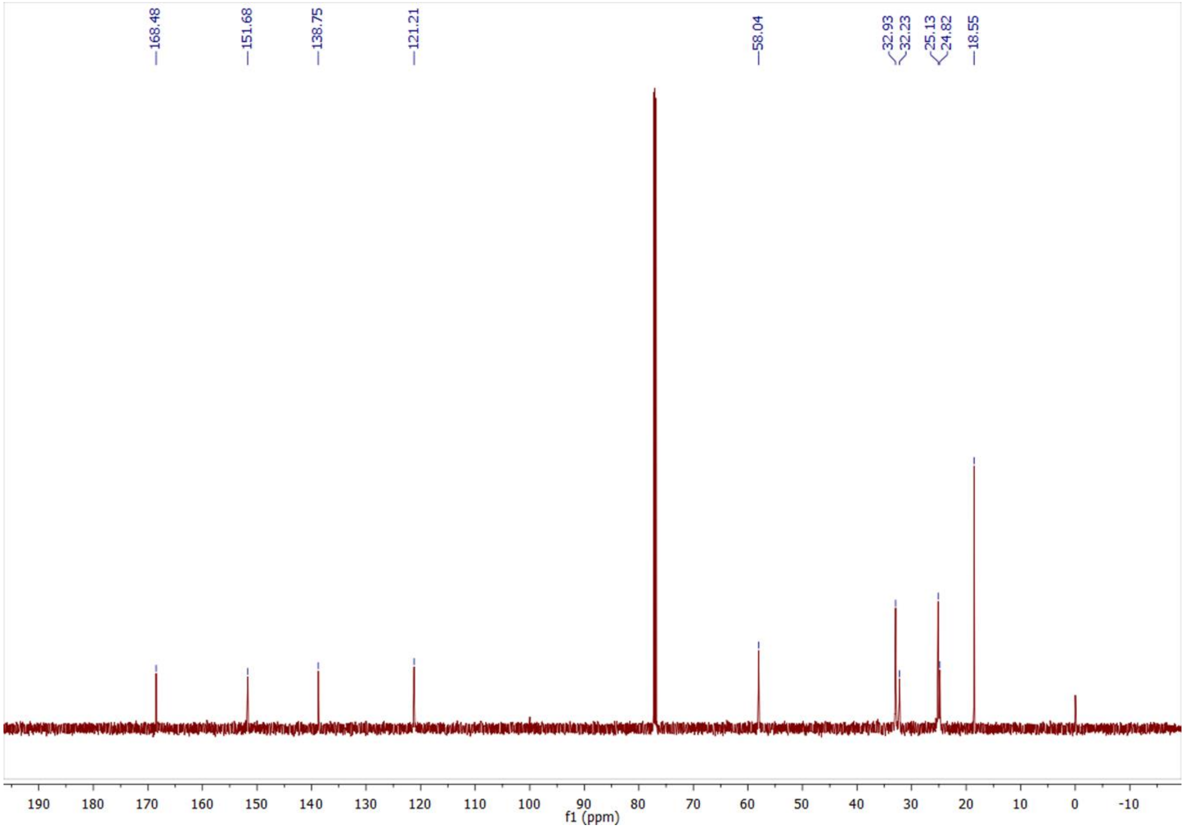

Figure S63. $^{13}C\ \{^{1}H\}$ NMR (151 MHz, $CDCl_3$) spectrum of compound **2b**.

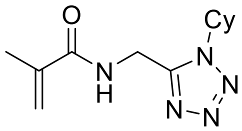

2b

N-((1-cyclohexyl-1*H*-tetrazol-5-yl)methyl)methacrylamide

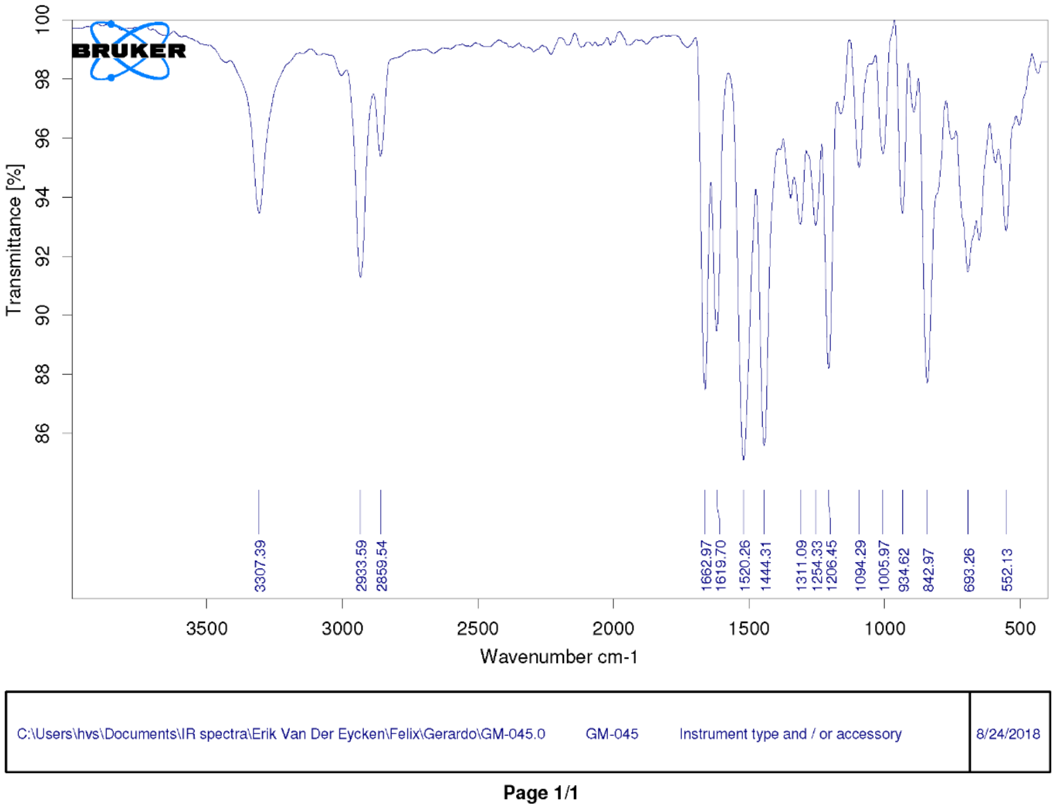

Figure S64. FT-IR (KBr) spectrum of compound **2b**.

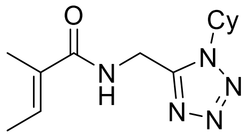

2c

(*E*)-*N*-((1-cyclohexyl-1*H*-tetrazol-5-yl)methyl)-2-methylbut-2-enamide

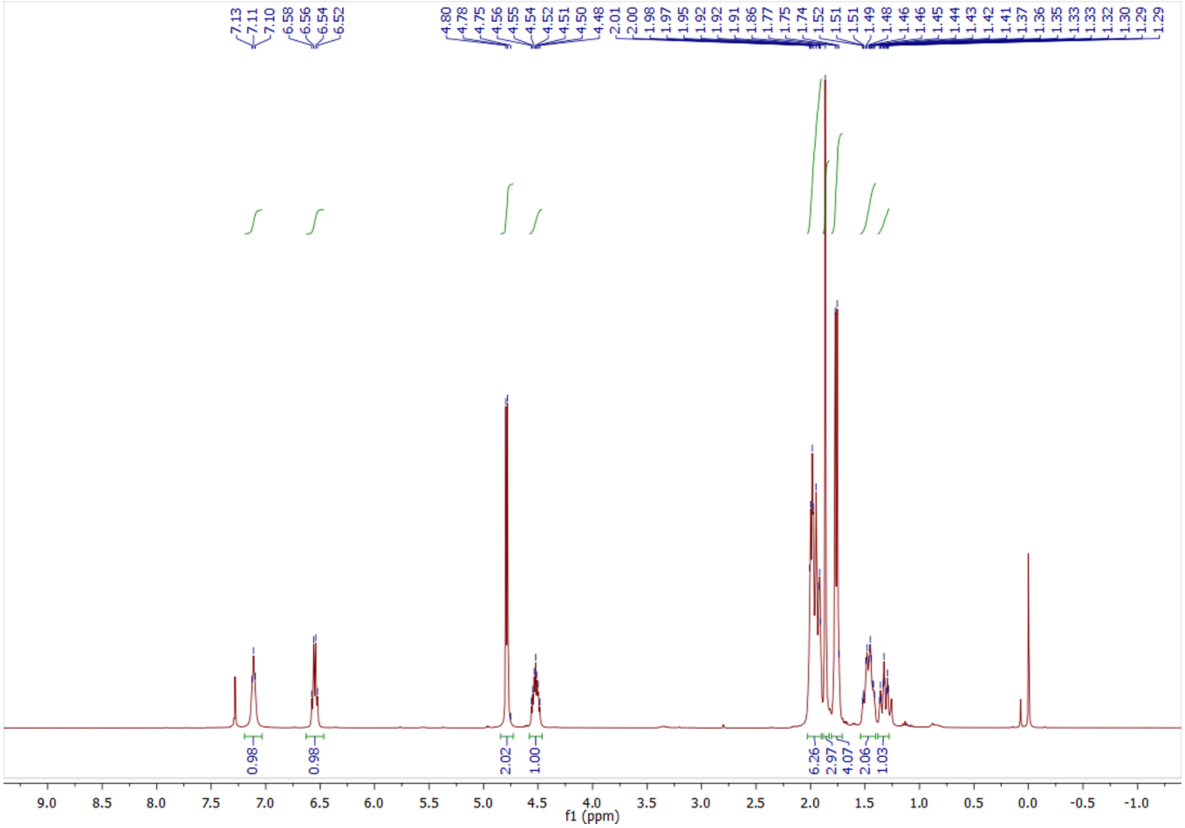

Figure S65. ^{1}H NMR (400 MHz, $CDCl_3$) spectrum of compound **2c**.

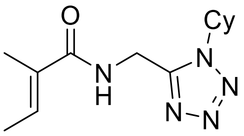

2c

(*E*)-*N*-((1-cyclohexyl-1*H*-tetrazol-5-yl)methyl)-2-methylbut-2-enamide

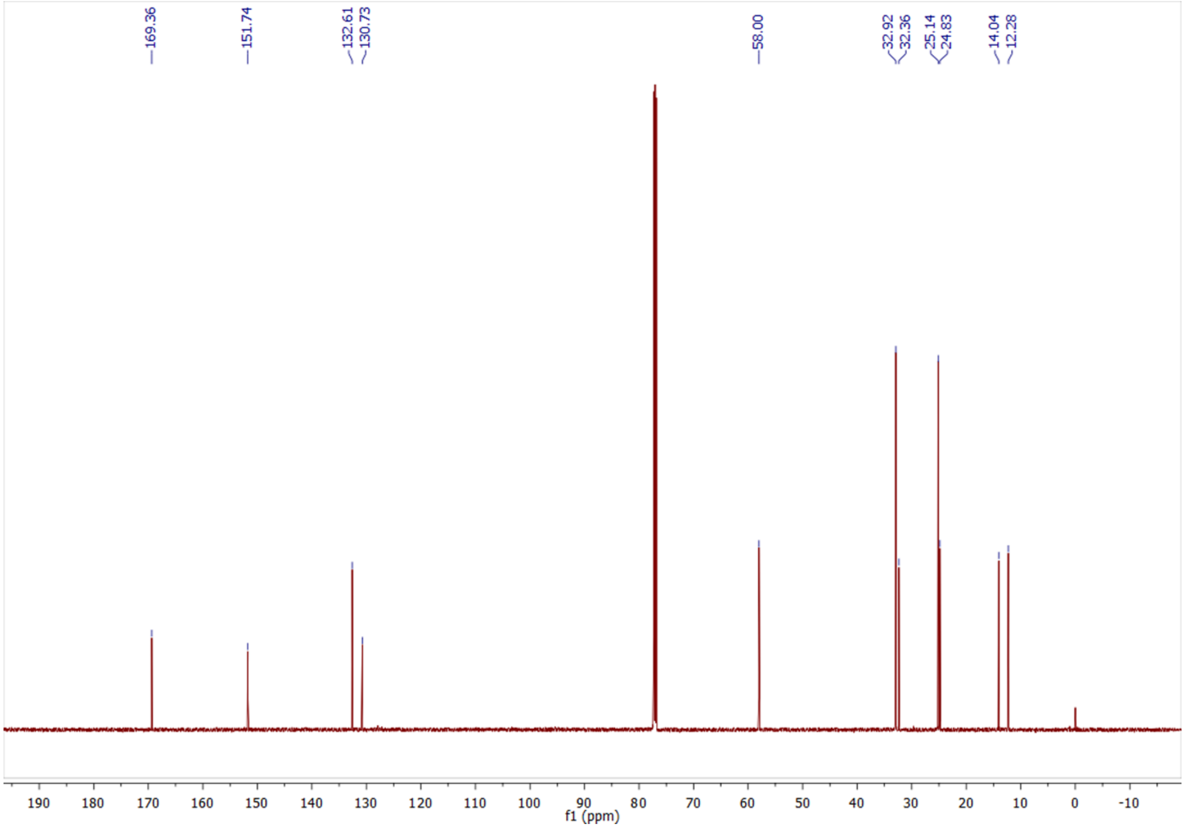

Figure S66. $^{13}C\ \{^{1}H\}$ NMR (151 MHz, $CDCl_3$) spectrum of compound **2c**.

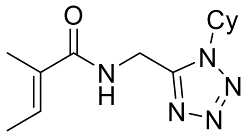

2c

(*E*)-*N*-((1-cyclohexyl-1*H*-tetrazol-5-yl)methyl)-2-methylbut-2-enamide

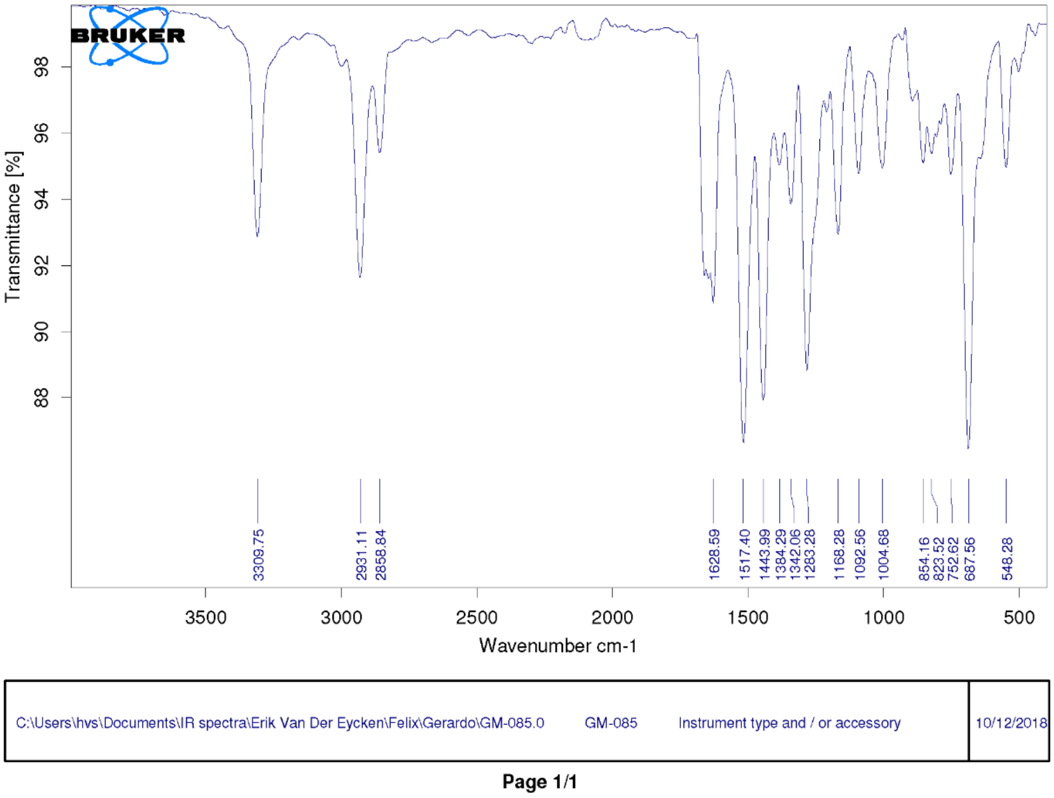

Figure S67. FT-IR (KBr) spectrum of compound **2c**.

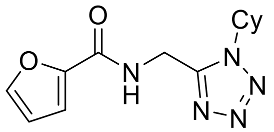

2d

N-((1-cyclohexyl-1*H*-tetrazol-5-yl)methyl)furan-2-carboxamide

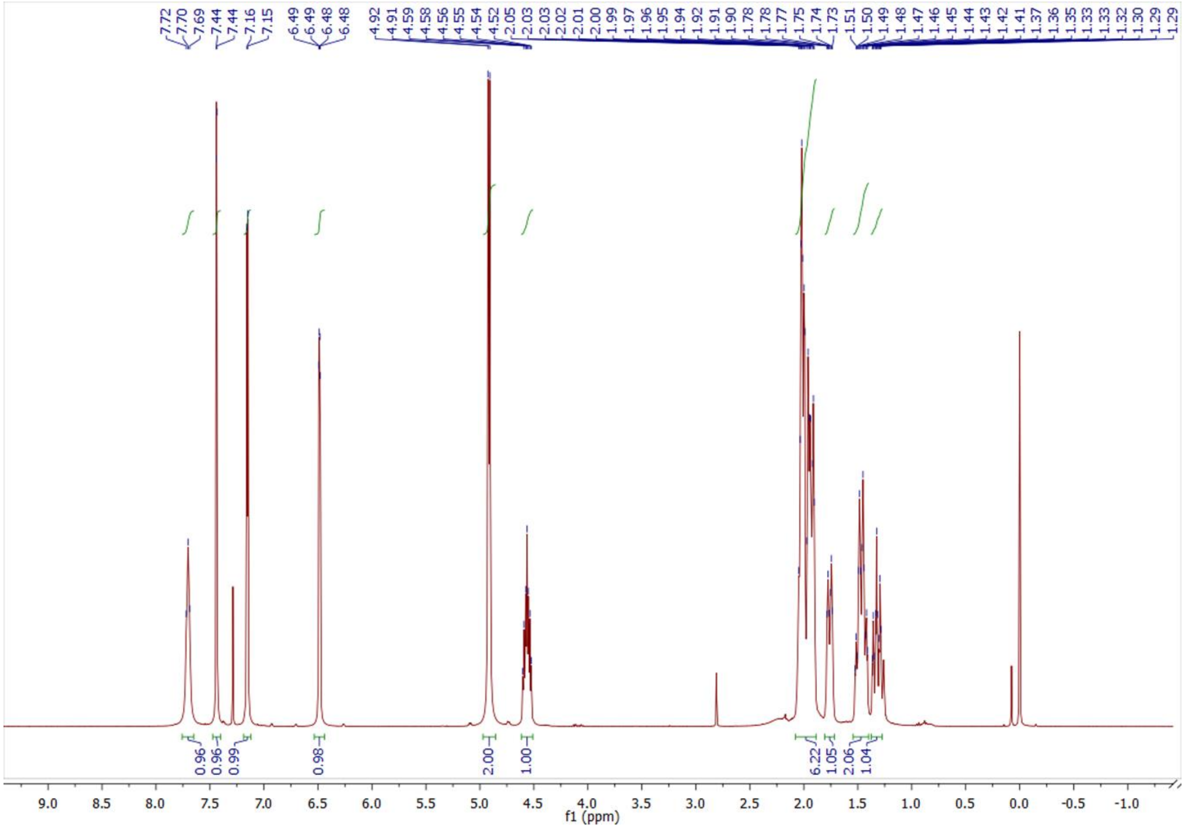

Figure S68. ^{1}H NMR (400 MHz, $CDCl_3$) spectrum of compound **2d**.

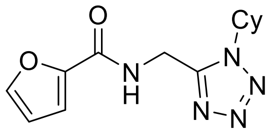

2d

N-((1-cyclohexyl-1*H*-tetrazol-5-yl)methyl)furan-2-carboxamide

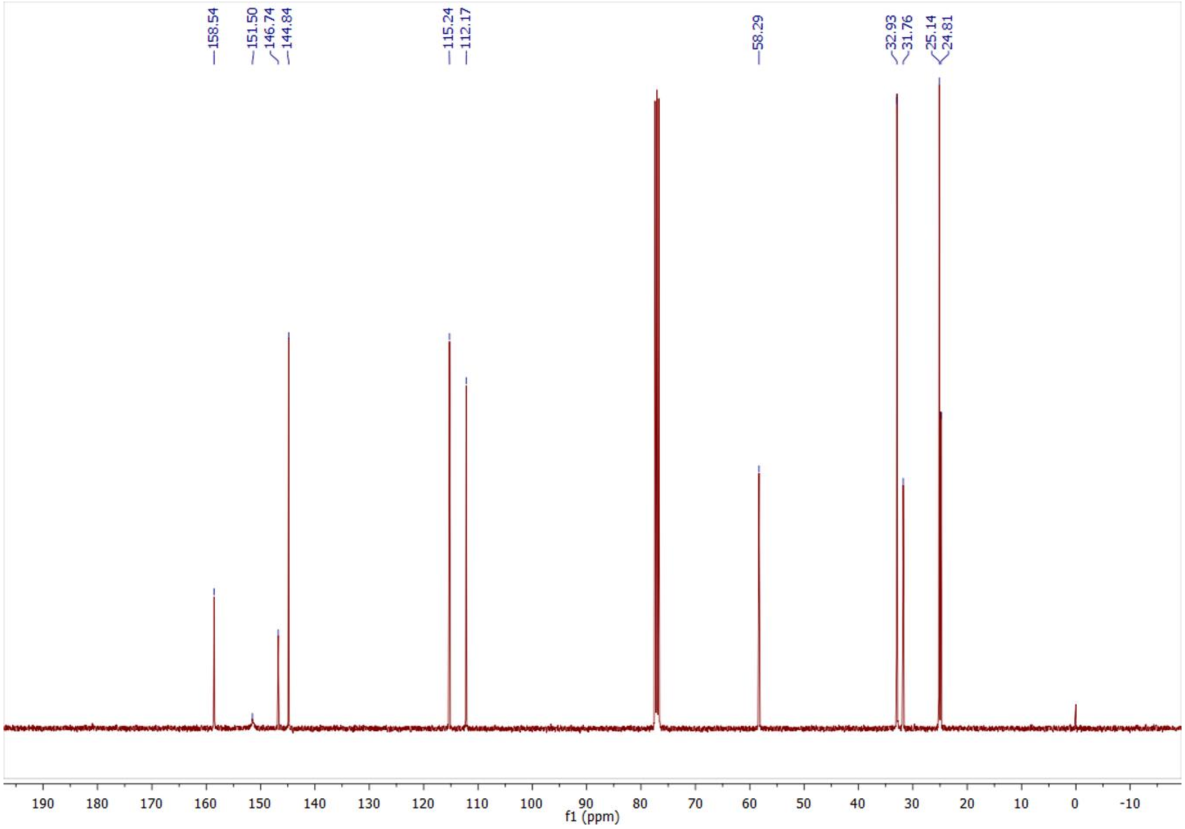

Figure S69. $^{13}C\ \{^{1}H\}$ NMR (101 MHz, $CDCl_3$) spectrum of compound **2d**.

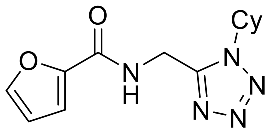

2d

N-((1-cyclohexyl-1*H*-tetrazol-5-yl)methyl)furan-2-carboxamide

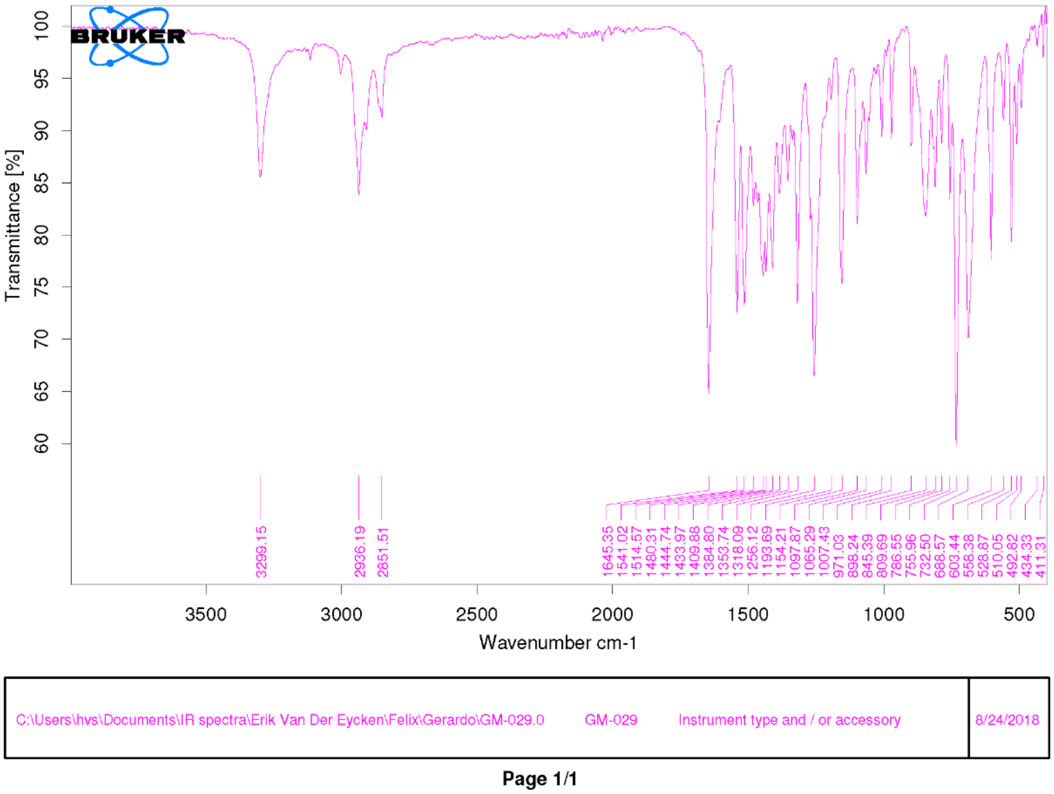

Figure S70. FT-IR (KBr) spectrum of compound **2d**.

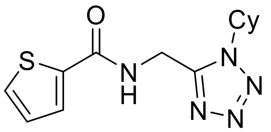

2e

N-((1-cyclohexyl-1*H*-tetrazol-5-yl)methyl)thiophene-2-carboxamide

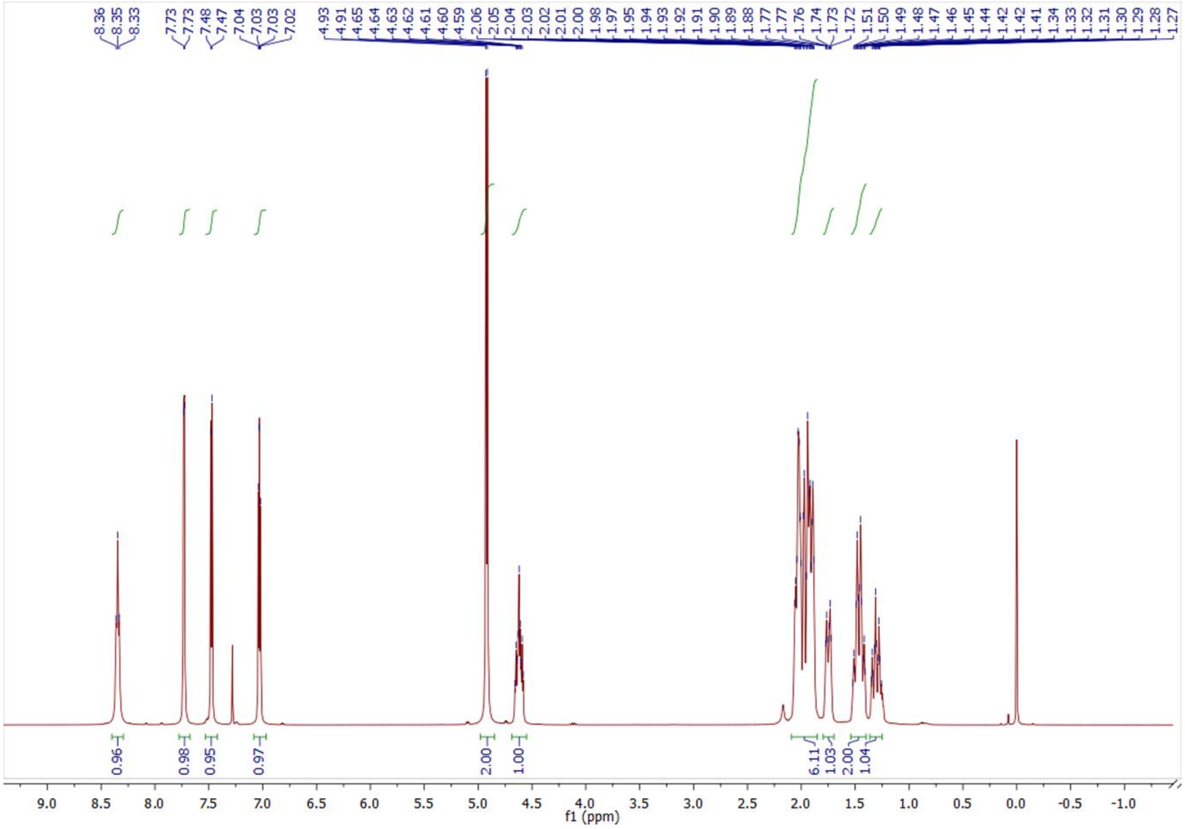

Figure S71. ^{1}H NMR (400 MHz, $CDCl_3$) spectrum of compound **2e**.

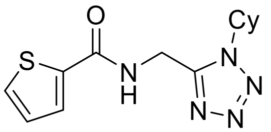

2e

N-((1-cyclohexyl-1*H*-tetrazol-5-yl)methyl)thiophene-2-carboxamide

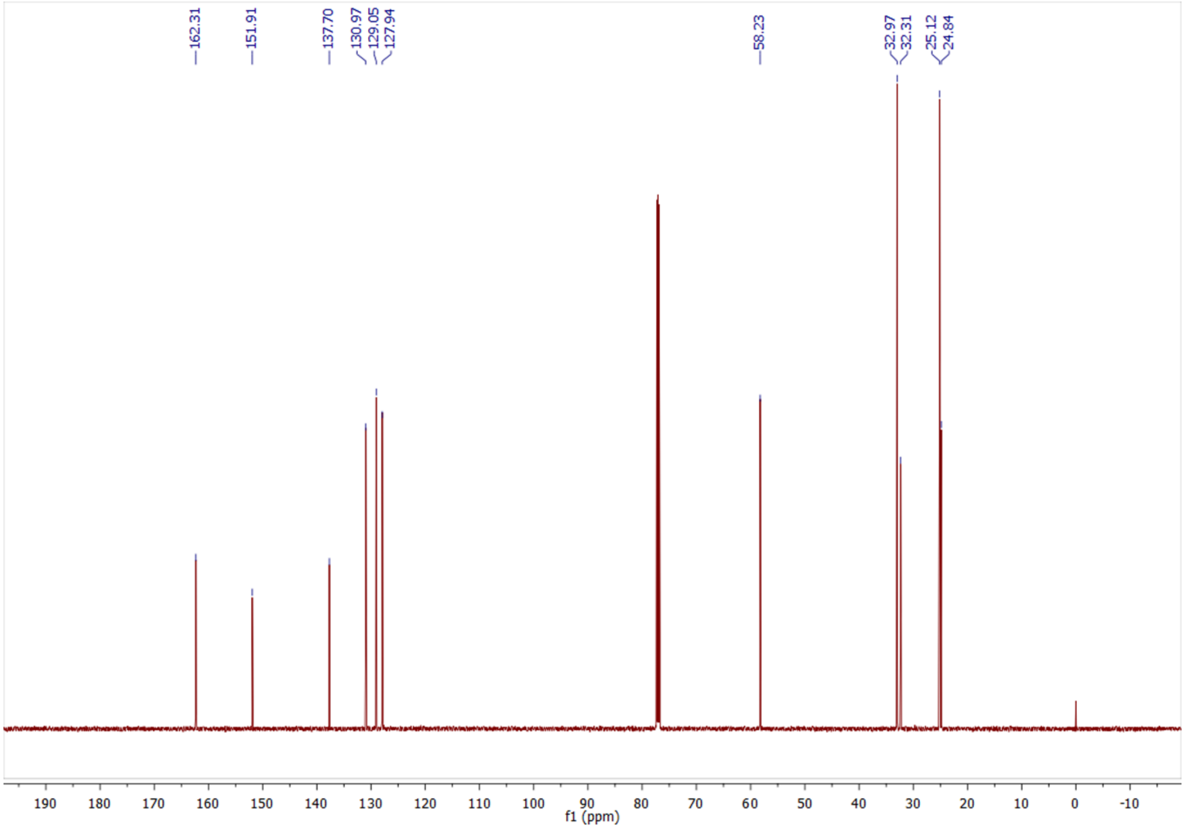

Figure S72. $^{13}C\{^1H\}$ NMR (151 MHz, $CDCl_3$) spectrum of compound **2e**.

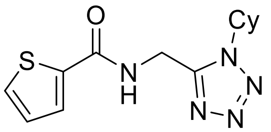

2e

N-((1-cyclohexyl-1*H*-tetrazol-5-yl)methyl)thiophene-2-carboxamide

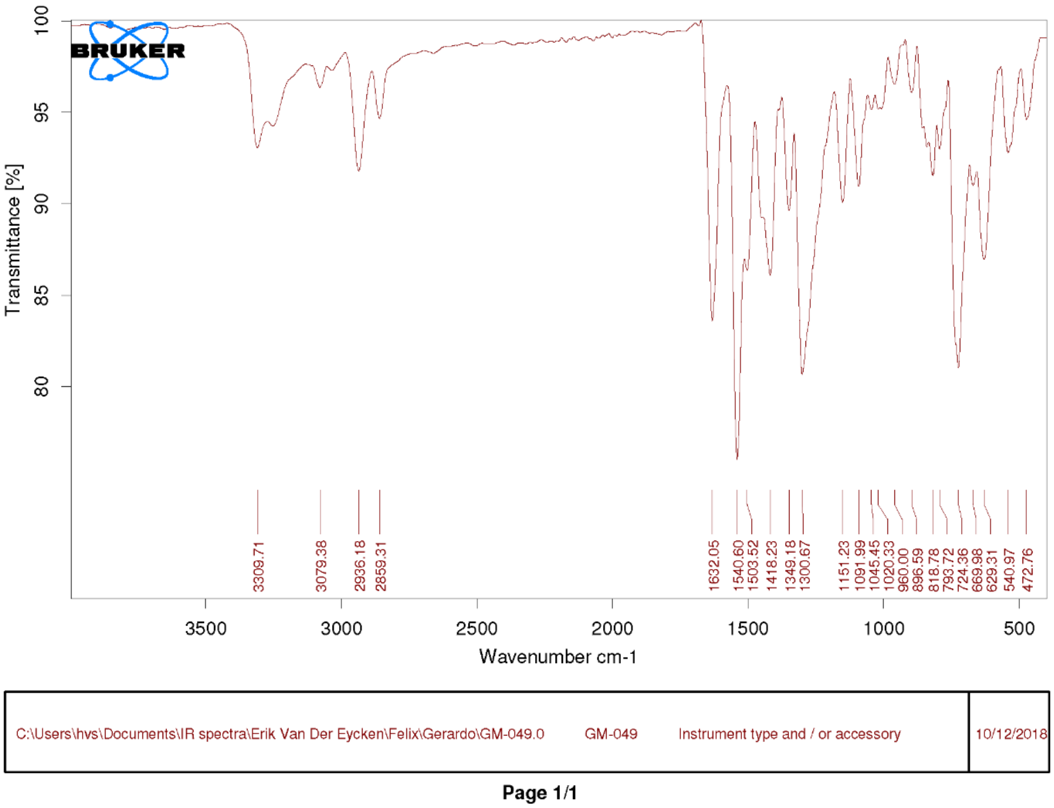

Figure S73. FT-IR (KBr) spectrum of compound **2e**.

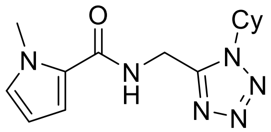

2f

N-((1-cyclohexyl-1*H*-tetrazol-5-yl)methyl)-1-methyl-1*H*-pyrrole-2-carboxamide

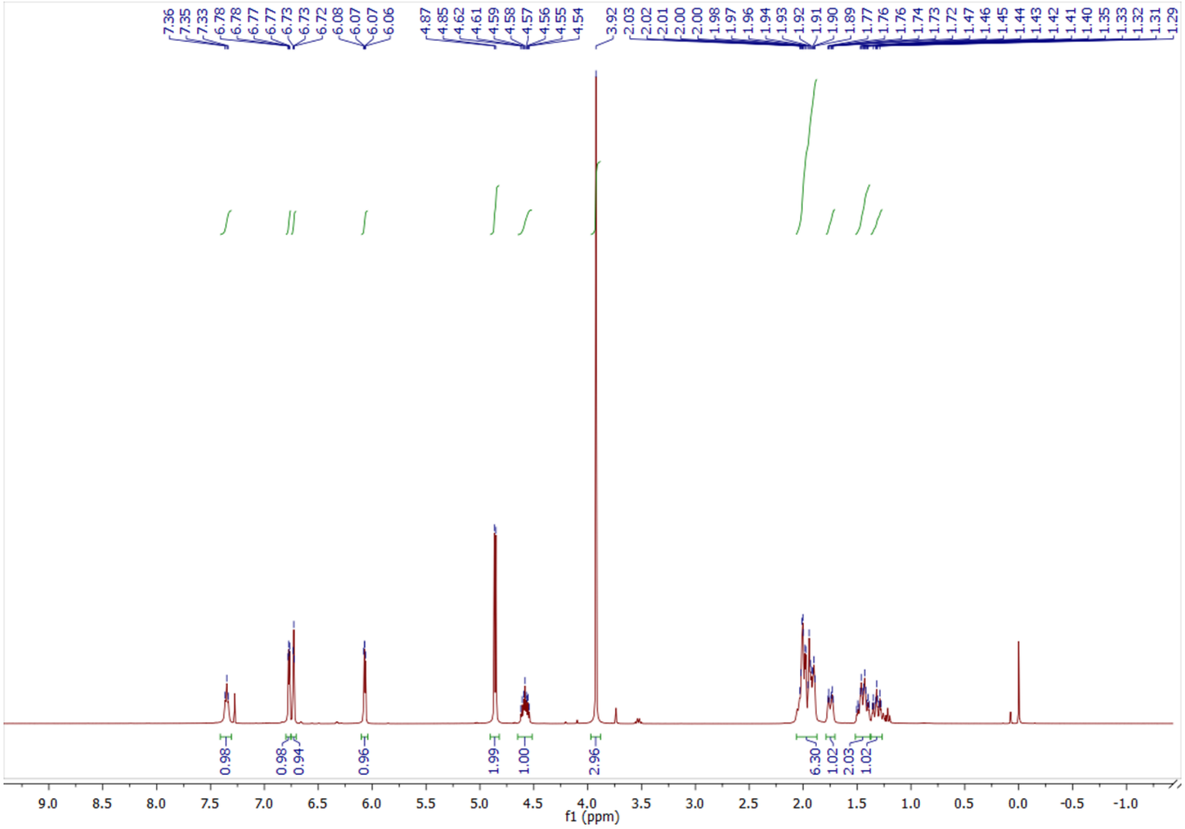

Figure S74. ^{1}H NMR (400 MHz, $CDCl_3$) spectrum of compound **2f**.

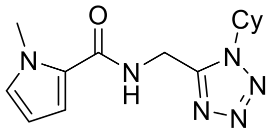

2f

N-((1-cyclohexyl-1*H*-tetrazol-5-yl)methyl)-1-methyl-
1*H*-pyrrole-2-carboxamide

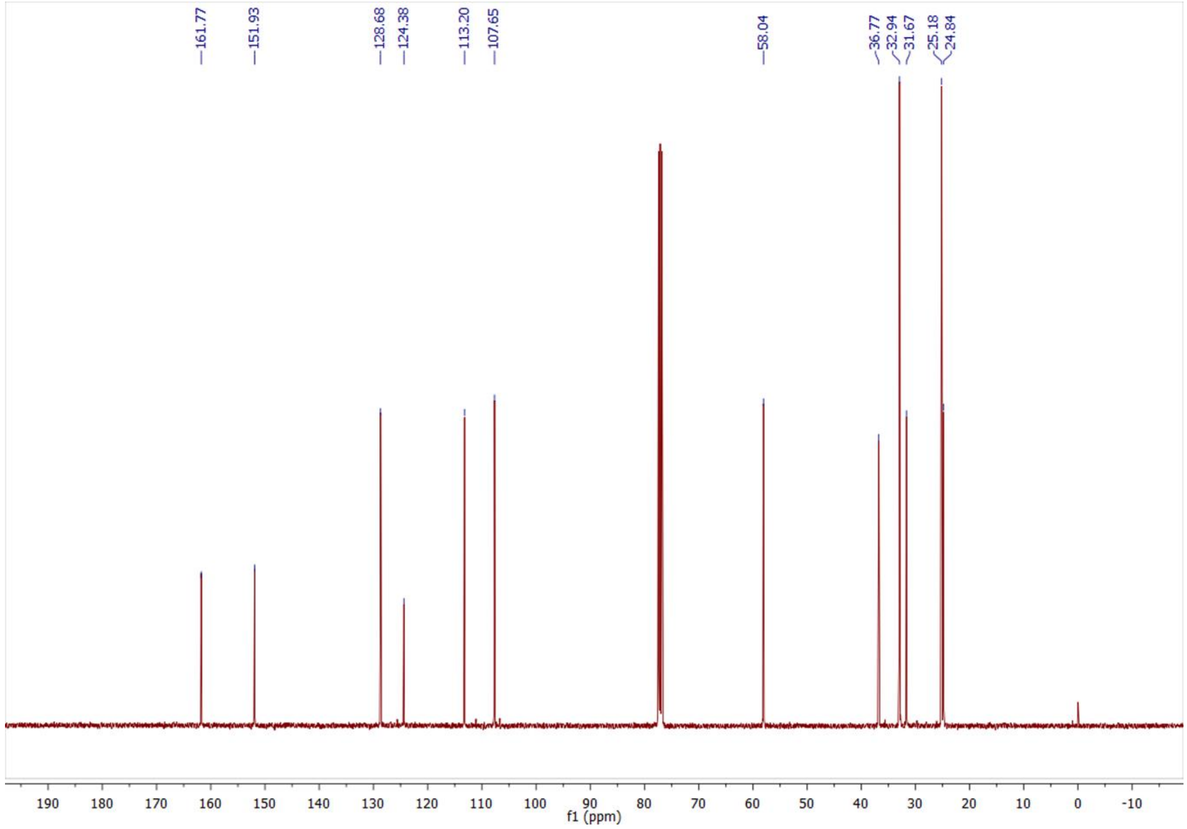

Figure S75. ^{13}C {^{1}H} NMR (101 MHz, $CDCl_3$) spectrum of compound **2f**.

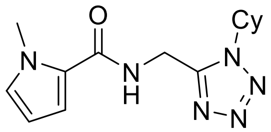

2f

N-((1-cyclohexyl-1*H*-tetrazol-5-yl)methyl)-1-methyl-
1*H*-pyrrole-2-carboxamide

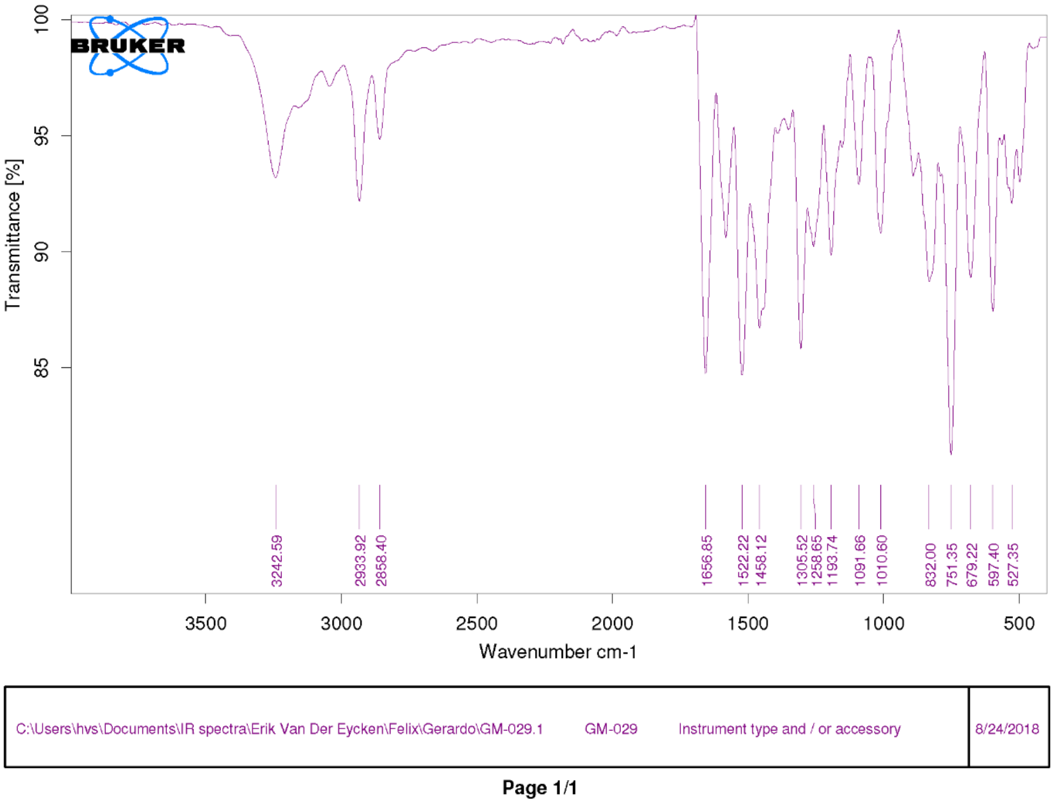

Figure S76. FT-IR (KBr) spectrum of compound **2f**.

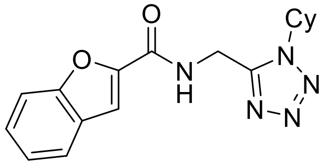

2g

N-((1-cyclohexyl-1*H*-tetrazol-5-yl)methyl)benzofuran-2-carboxamide

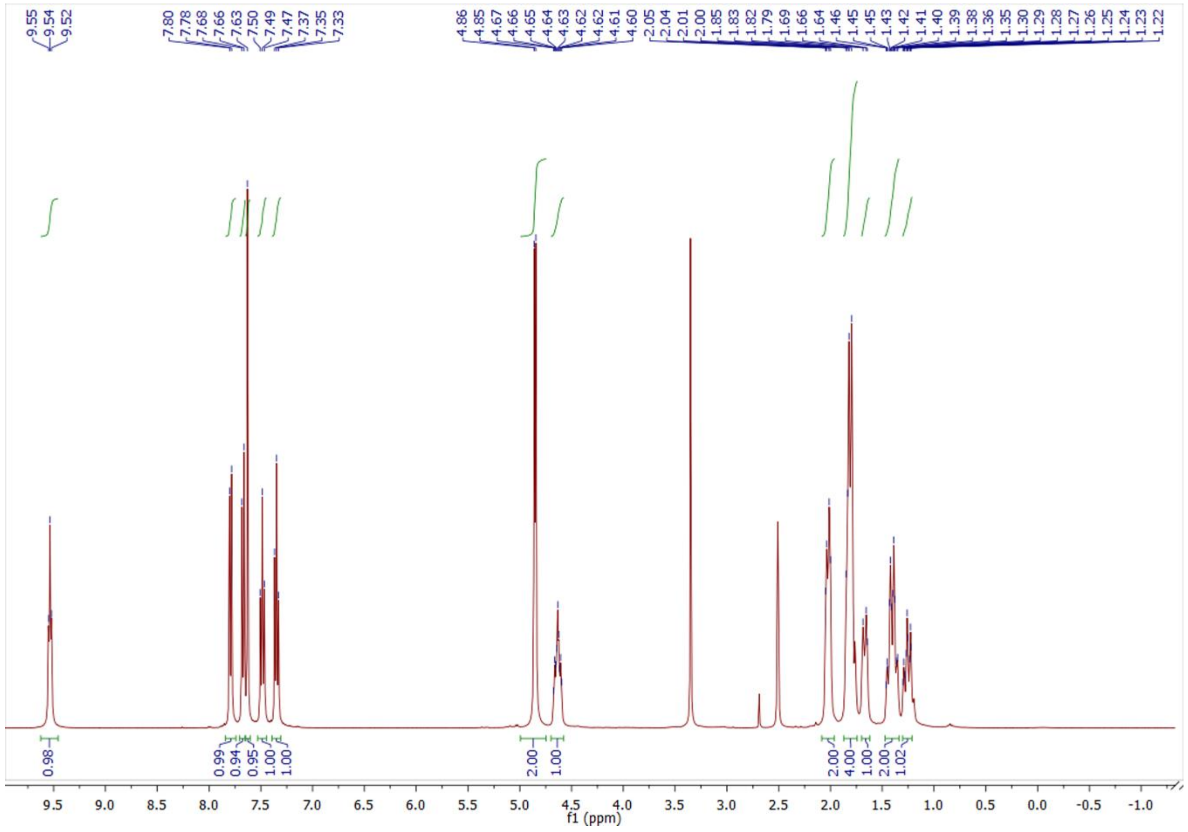

Figure S77. ^{1}H NMR (400 MHz, DMSO-*d*6) spectrum of compound **2g**.

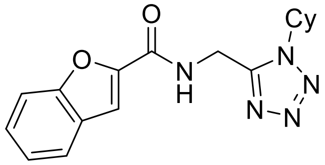

2g

N-((1-cyclohexyl-1*H*-tetrazol-5-yl)methyl)benzofuran-2-carboxamide

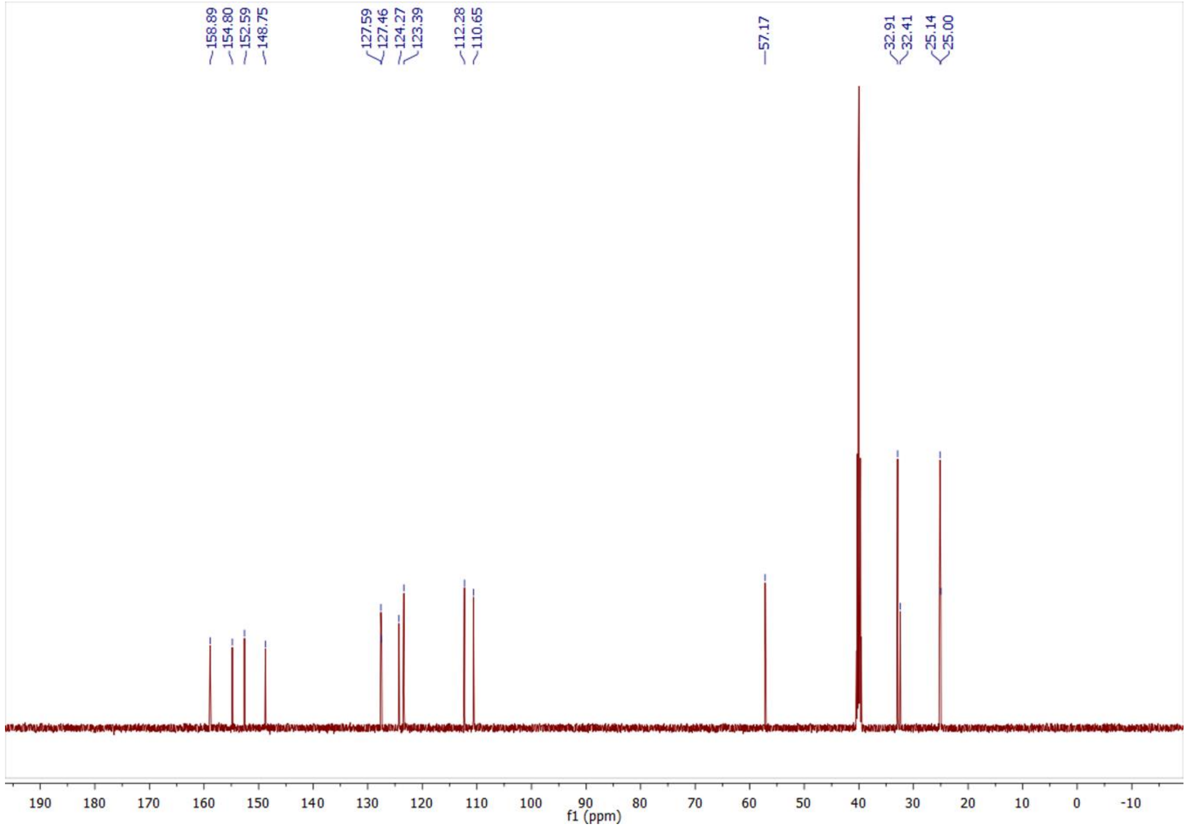

Figure S78. $^{13}C\ \{^1H\}$ NMR (151 MHz, DMSO-*d*6) spectrum of compound **2g**.

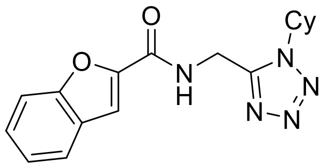

2g

N-((1-cyclohexyl-1*H*-tetrazol-5-yl)methyl)benzofuran-2-carboxamide

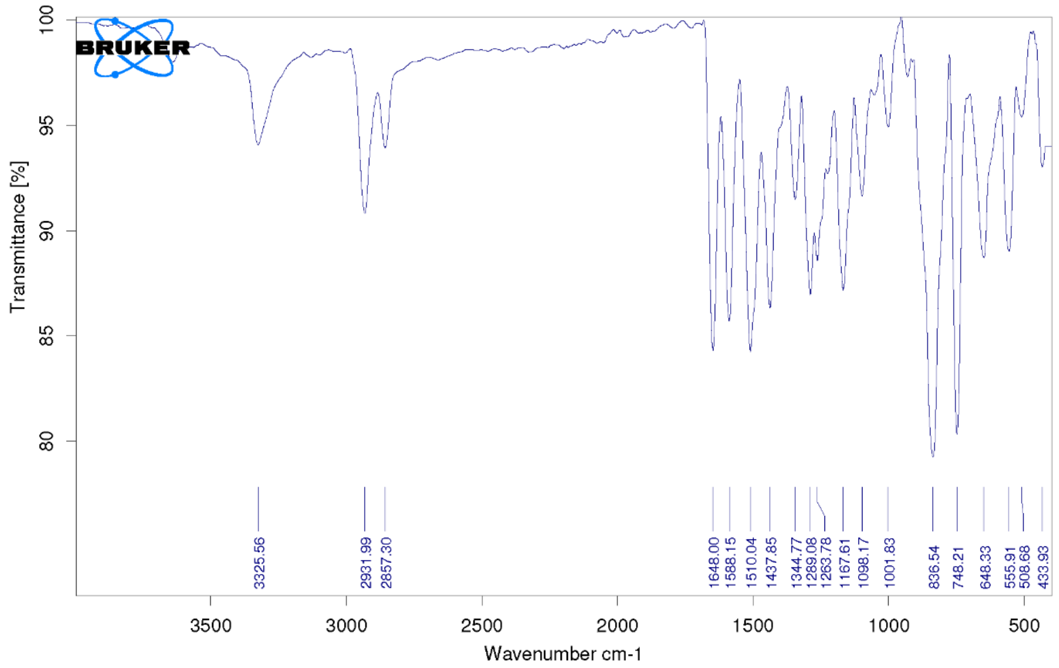

C:\Users\hvs\Documents\IR spectra\Erik Van Der Eycken\Felix\Gerardo\GM-51.0	GM-51	Instrument type and / or accessory	10/12/2018

Page 1/1

Figure S79. FT-IR (KBr) spectrum of compound **2g**.

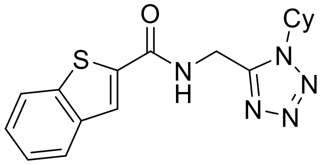

2h

N-((1-cyclohexyl-1*H*-tetrazol-5-yl)methyl)benzo[*b*]thiophene-2-carboxamide

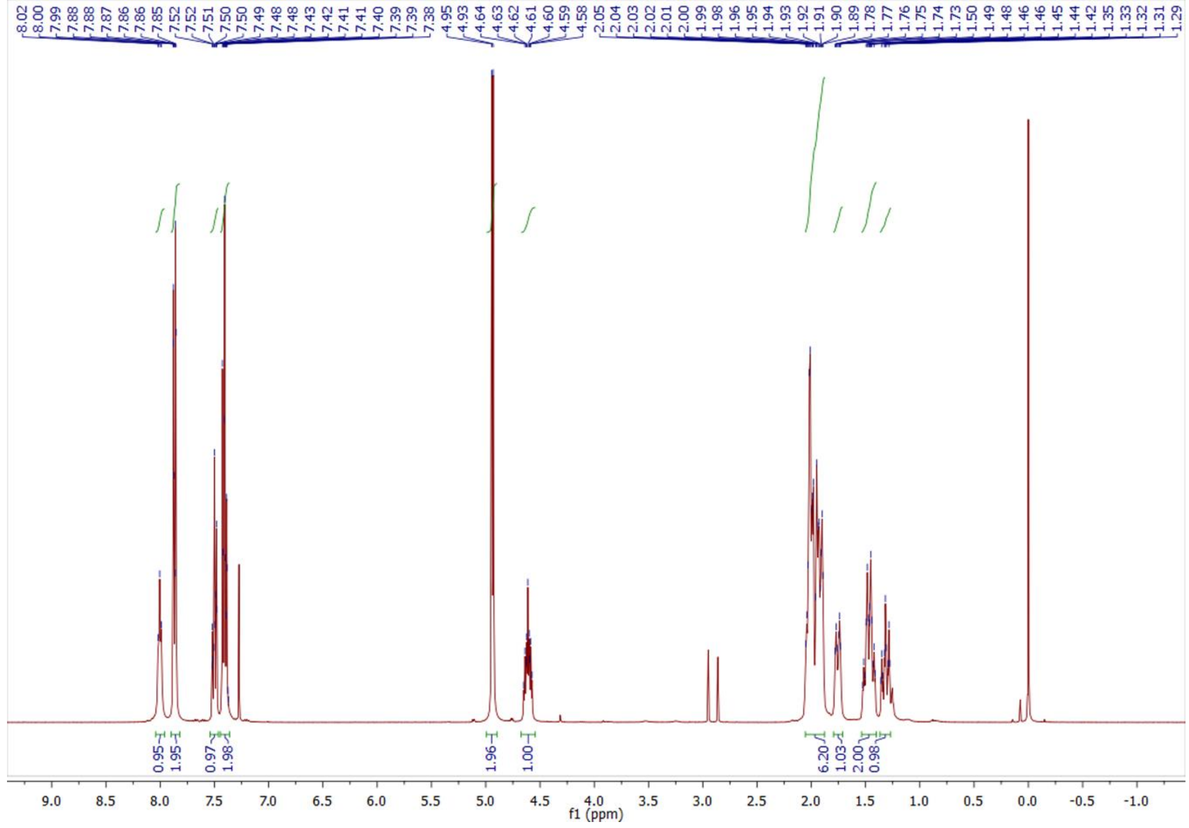

Figure S80. ^{1}H NMR (400 MHz, $CDCl_3$) spectrum of compound **2h**.

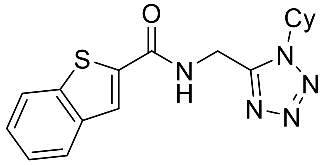

2h

N-((1-cyclohexyl-1*H*-tetrazol-5-yl)methyl)benzo[*b*]thiophene-2-carboxamide

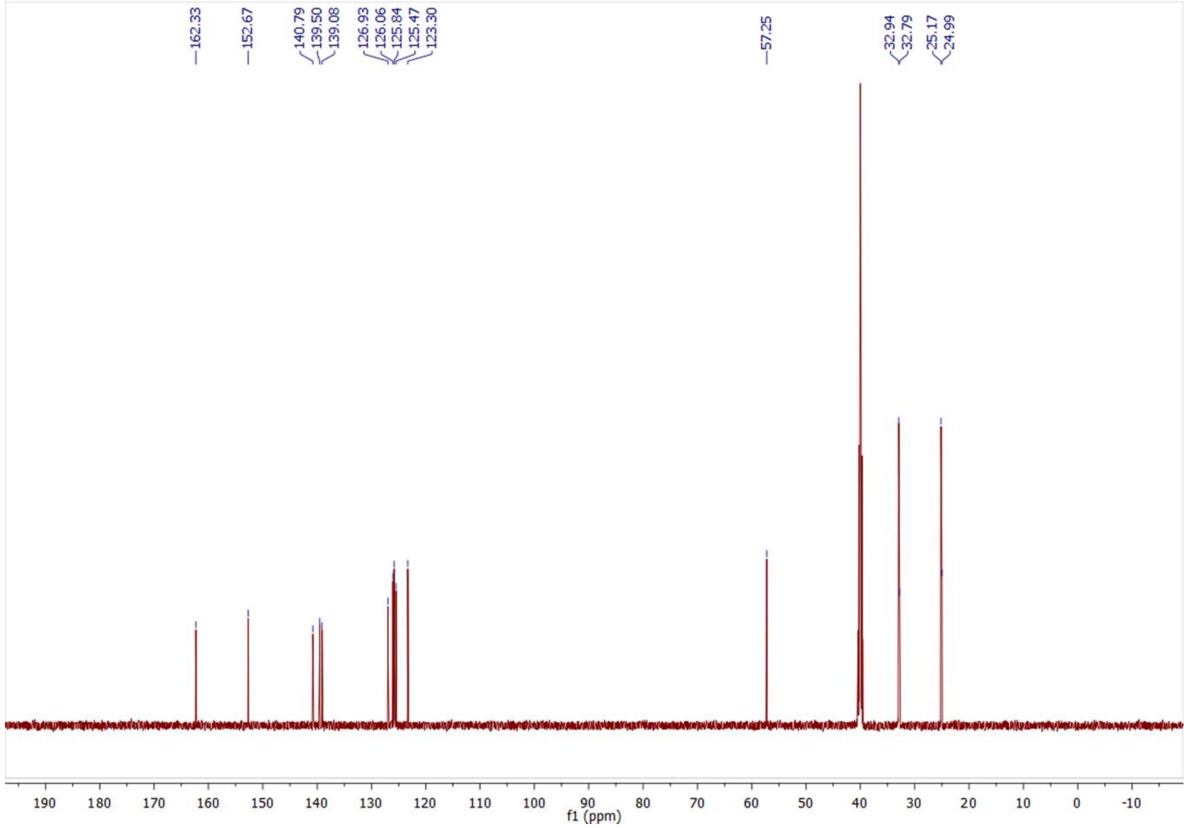

Figure S81. ^{13}C {^{1}H} NMR (151 MHz, DMSO-*d*6) spectrum of compound **2h**.

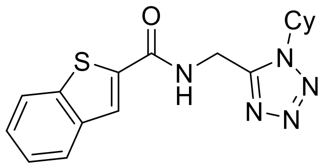

2h

N-((1-cyclohexyl-1*H*-tetrazol-5-yl)methyl)benzo[*b*]thiophene-2-carboxamide

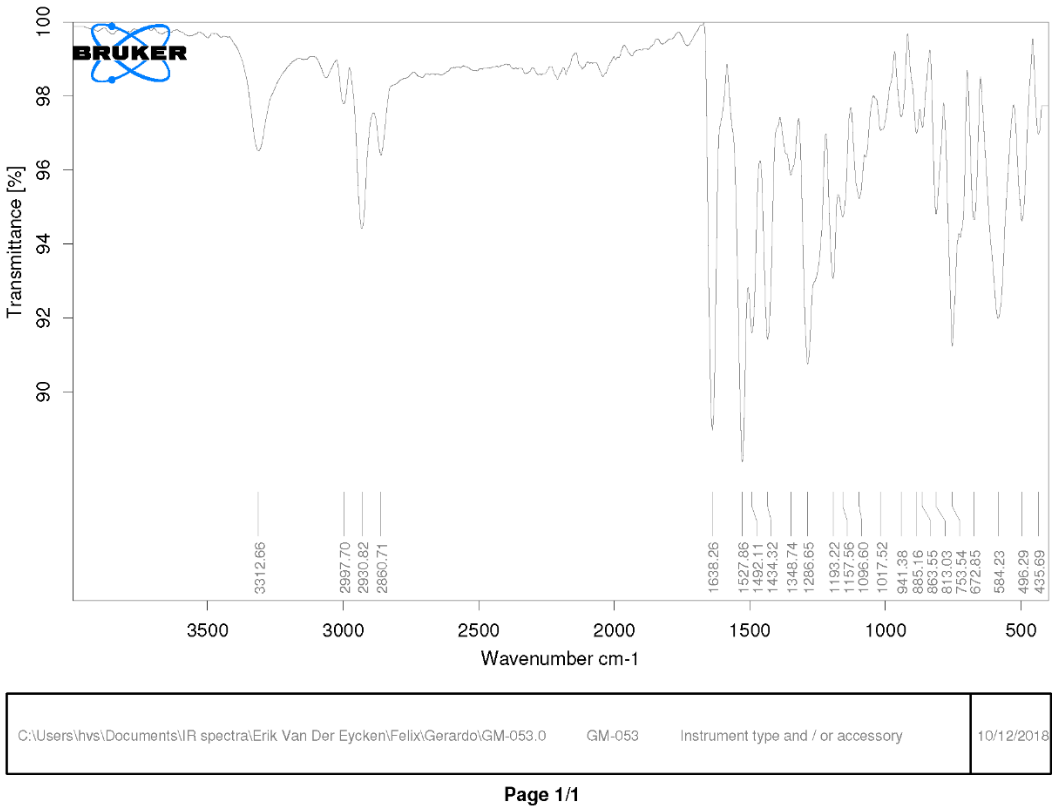

Figure S82. FT-IR (KBr) spectrum of compound **2h**.

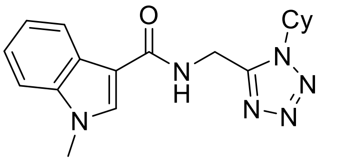

2i

N-((1-cyclohexyl-1*H*-tetrazol-5-yl)methyl)-1-methyl-1*H*-indole-3-carboxamide

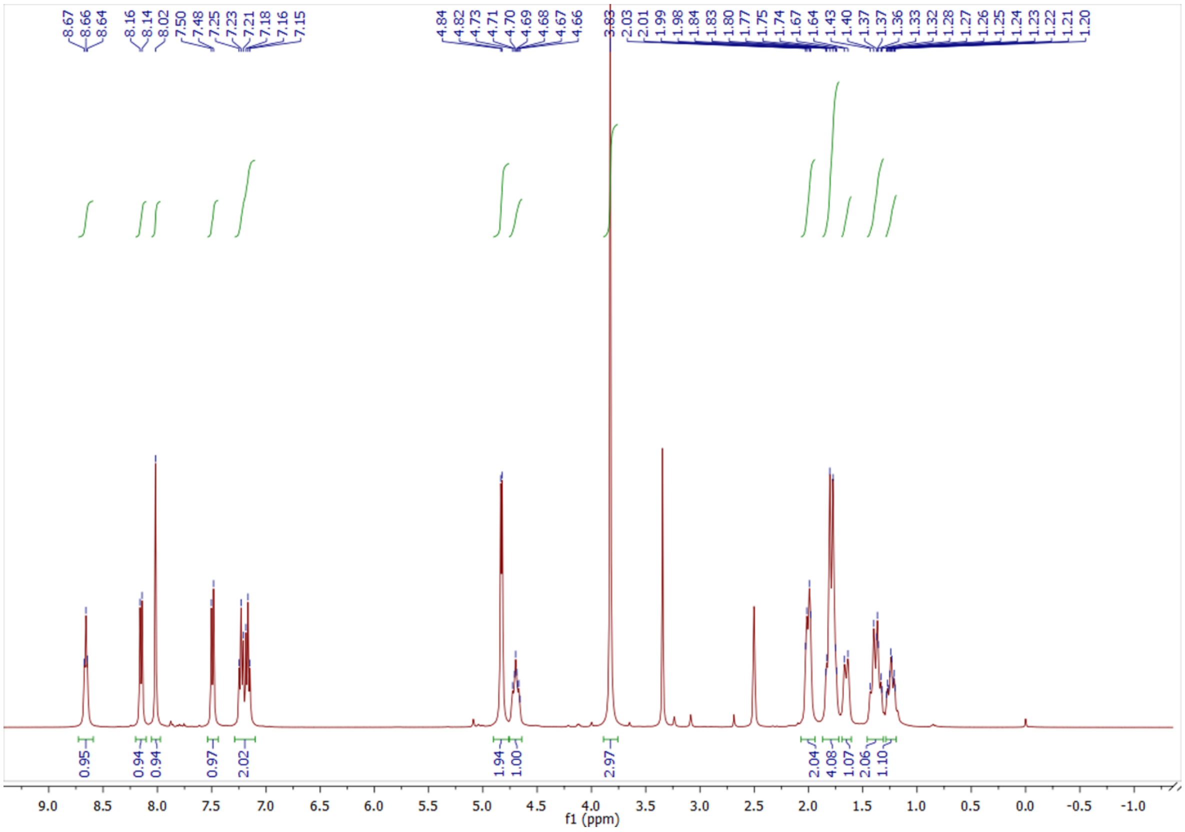

Figure S83. ^{1}H NMR (400 MHz, $CDCl_3$) spectrum of compound **2i**.

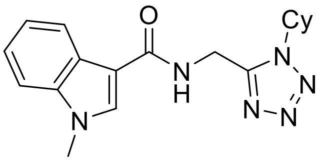

2i

N-((1-cyclohexyl-1*H*-tetrazol-5-yl)methyl)-1-methyl-1*H*-indole-3-carboxamide

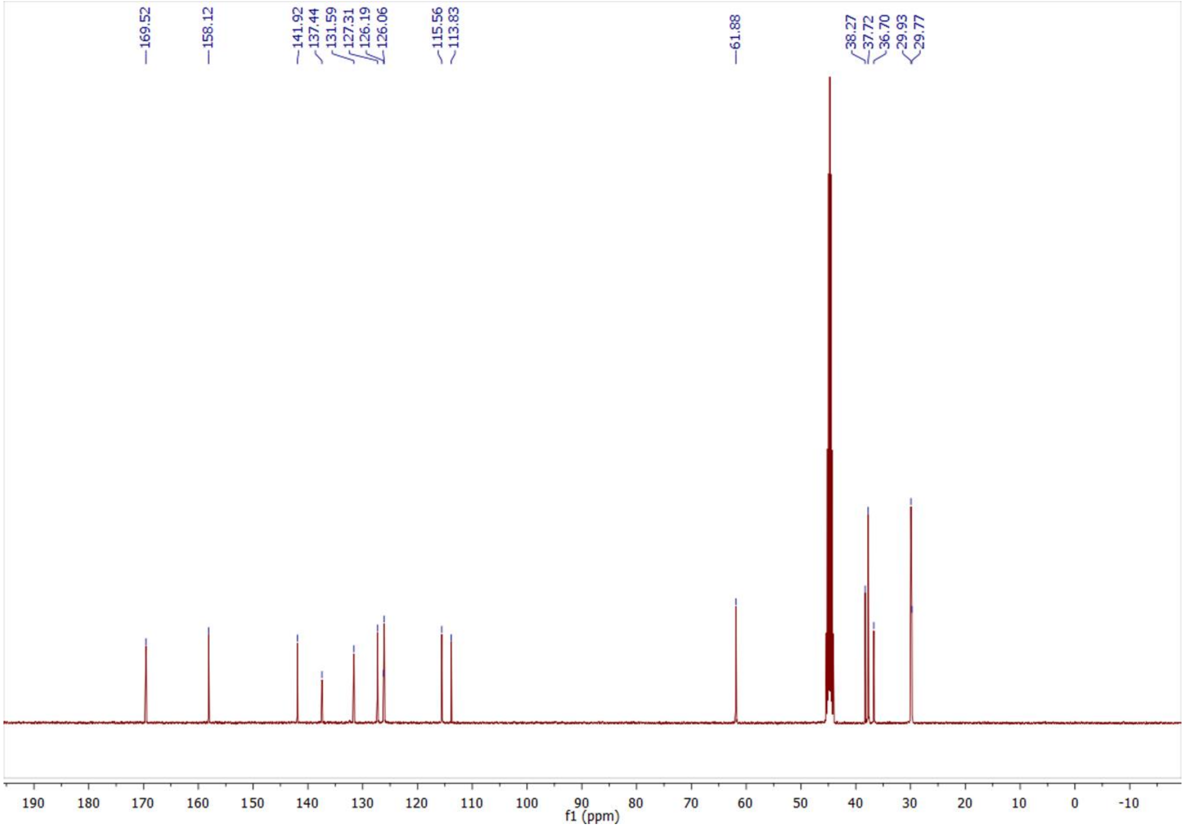

Figure S84. ^{13}C {^{1}H} NMR (101 MHz, $CDCl_3$) spectrum of compound **2i**.

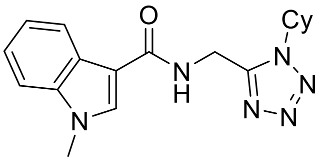

2i

N-((1-cyclohexyl-1*H*-tetrazol-5-yl)methyl)-1-methyl-1*H*-indole-3-carboxamide

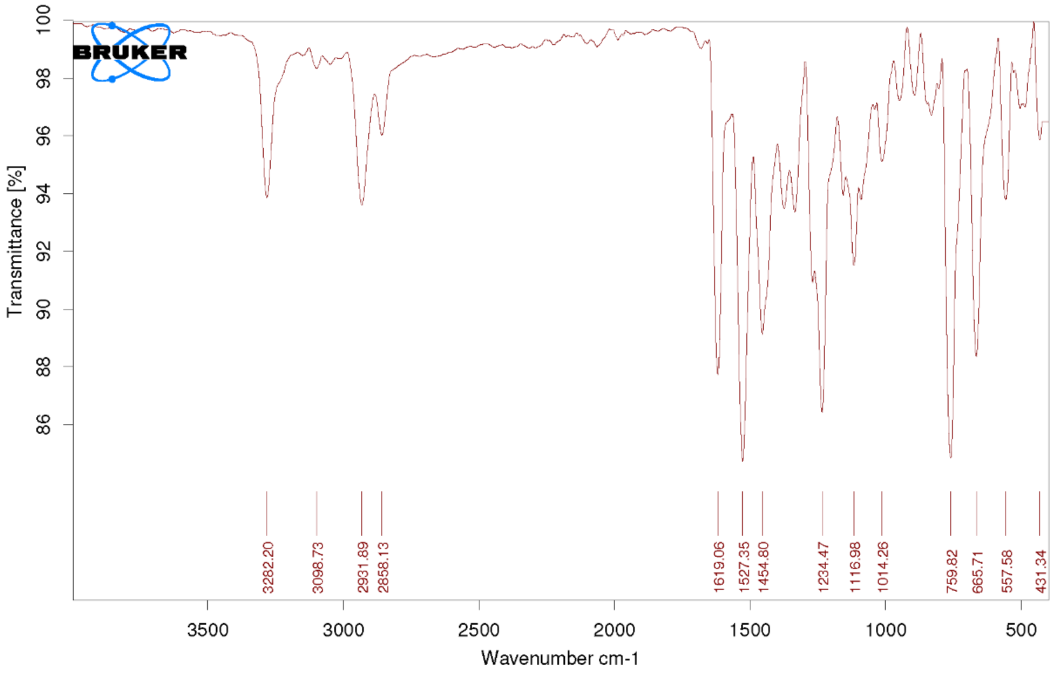

C:\Users\hvs\Documents\IR spectra\Erik Van Der Eycken\Felix\Gerardo\GM-027.0 GM-027 Instrument type and / or accessory			8/24/2018

Page 1/1

Figure S85. FT-IR (KBr) spectrum of compound **2i**.

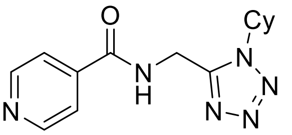

2j

N-((1-cyclohexyl-1*H*-tetrazol-5-yl)methyl)isonicotinamide

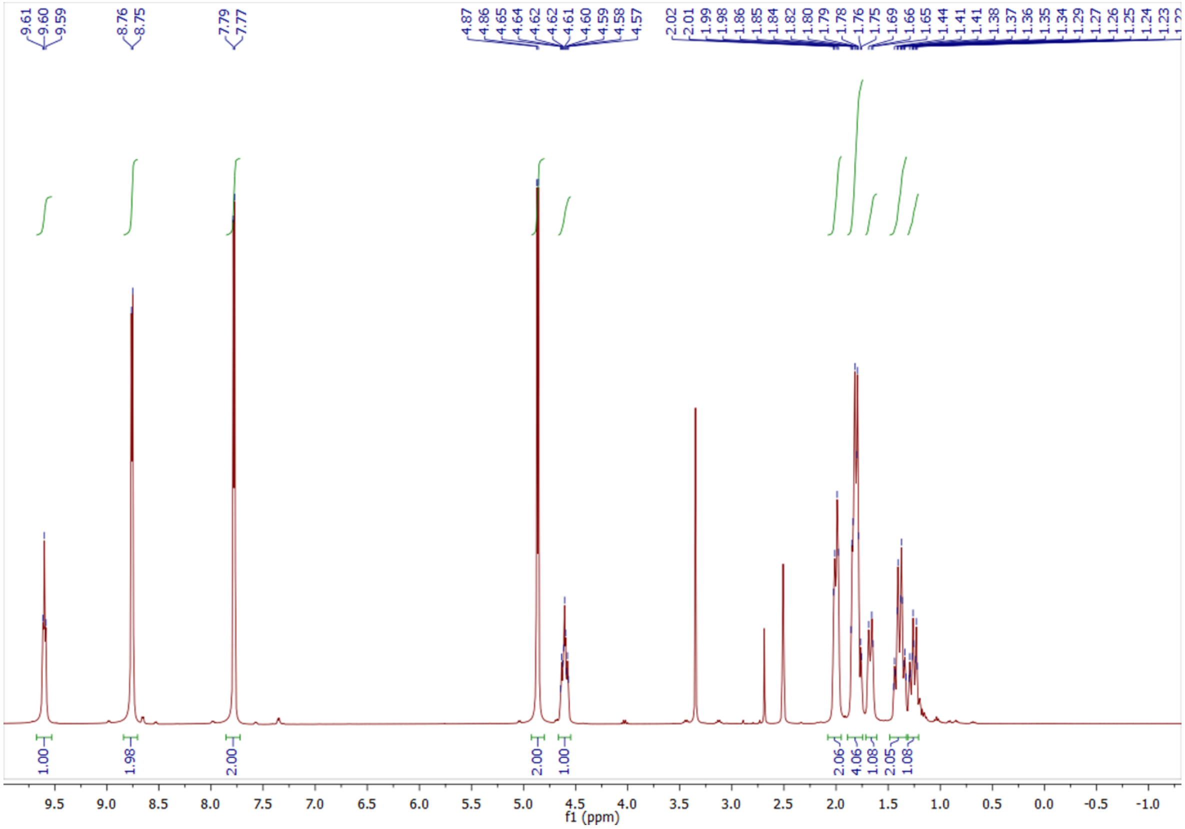

Figure S86. ^{1}H NMR (400 MHz, DMSO-*d*6) spectrum of compound **2j**.

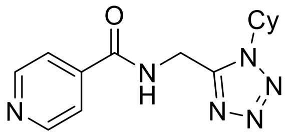

2j

N-((1-cyclohexyl-1*H*-tetrazol-5-yl)methyl)isonicotinamide

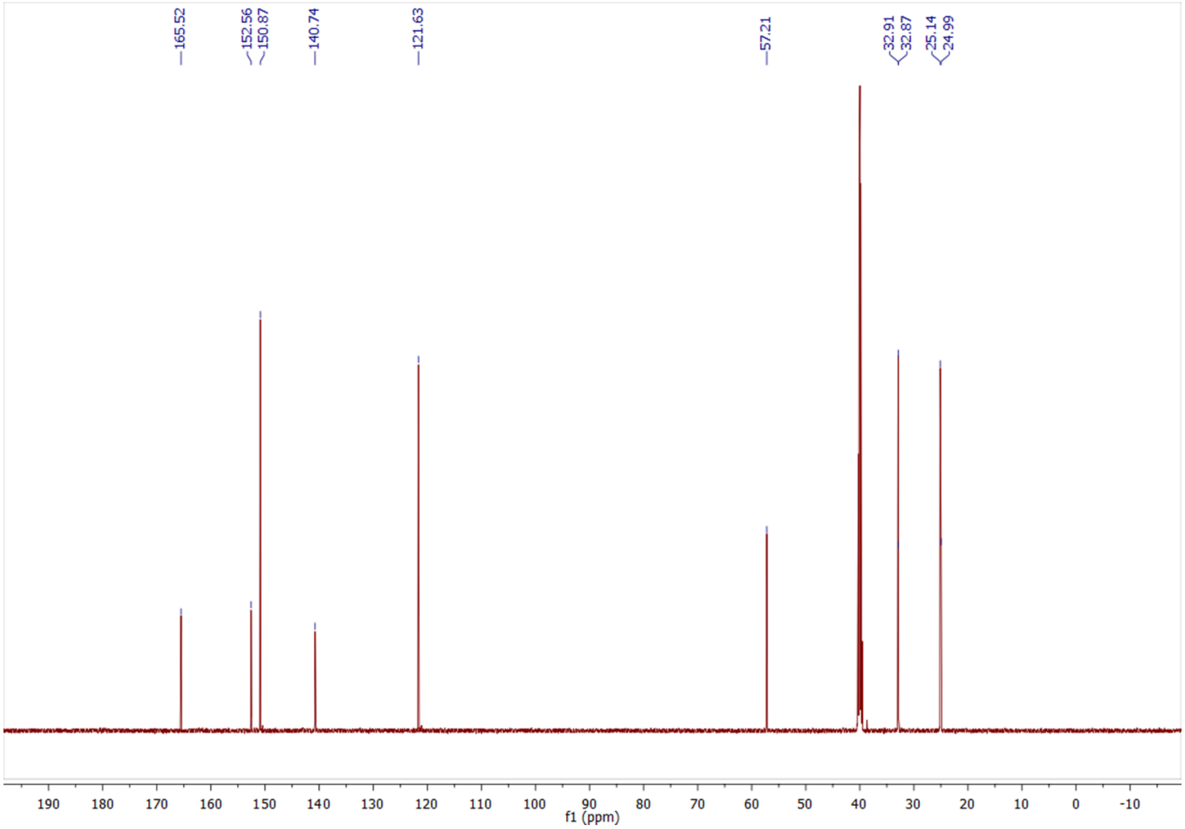

Figure S87. $^{13}C\ \{^{1}H\}$ NMR (151 MHz, DMSO-*d*6) spectrum of compound **2j**.

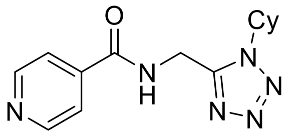

2j

N-((1-cyclohexyl-1*H*-tetrazol-5-yl)methyl)isonicotinamide

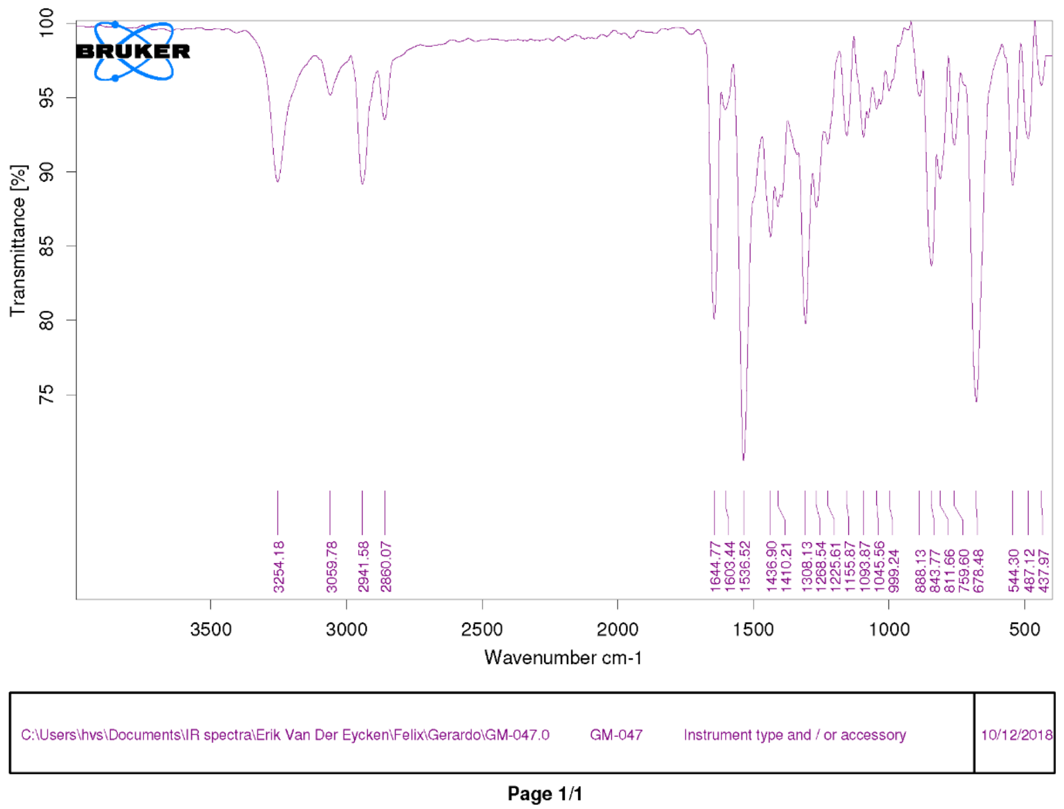

Figure S88. FT-IR (KBr) spectrum of compound **2j**.

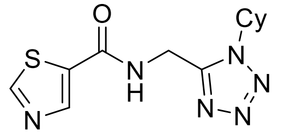

2k

N-((1-cyclohexyl-1*H*-tetrazol-5-yl)methyl)thiazole-5-carboxamide

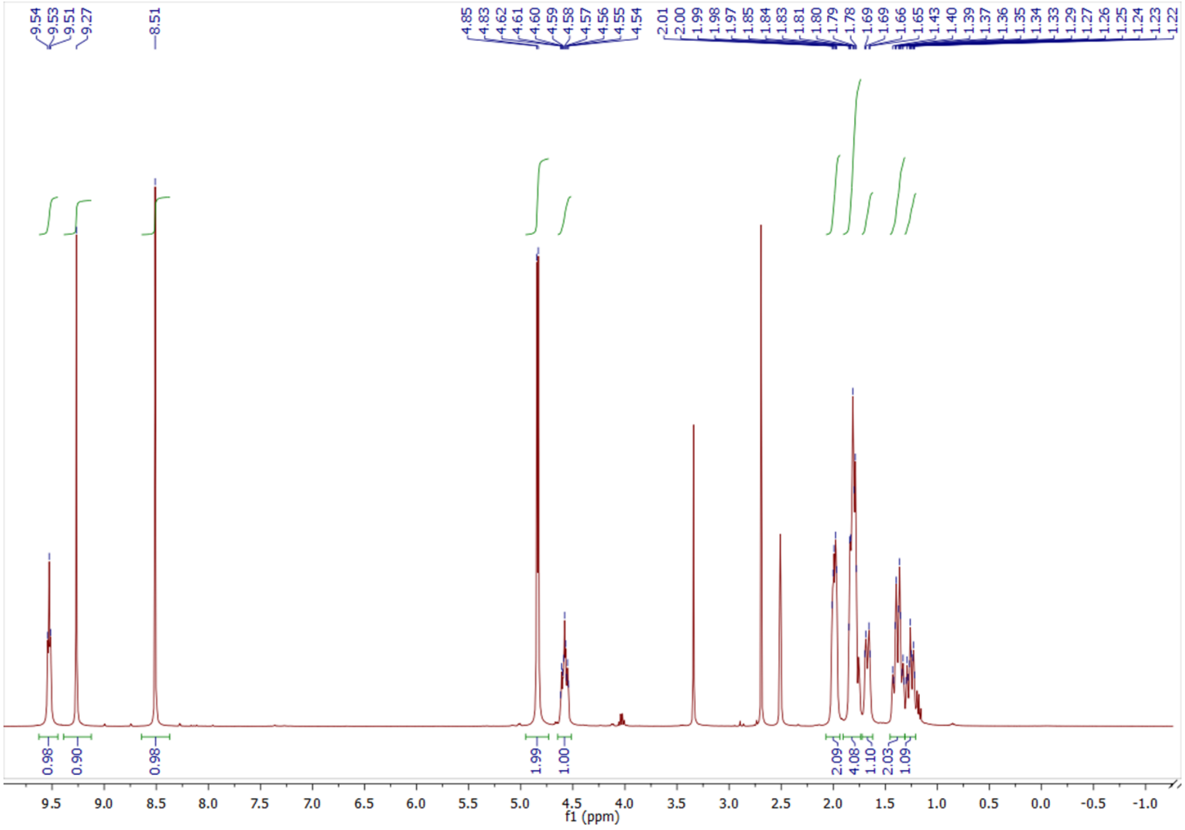

Figure S89. ^{1}H NMR (400 MHz, DMSO-*d*6) spectrum of compound **2k**.

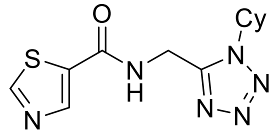

2k

N-((1-cyclohexyl-1*H*-tetrazol-5-yl)methyl)thiazole-5-carboxamide

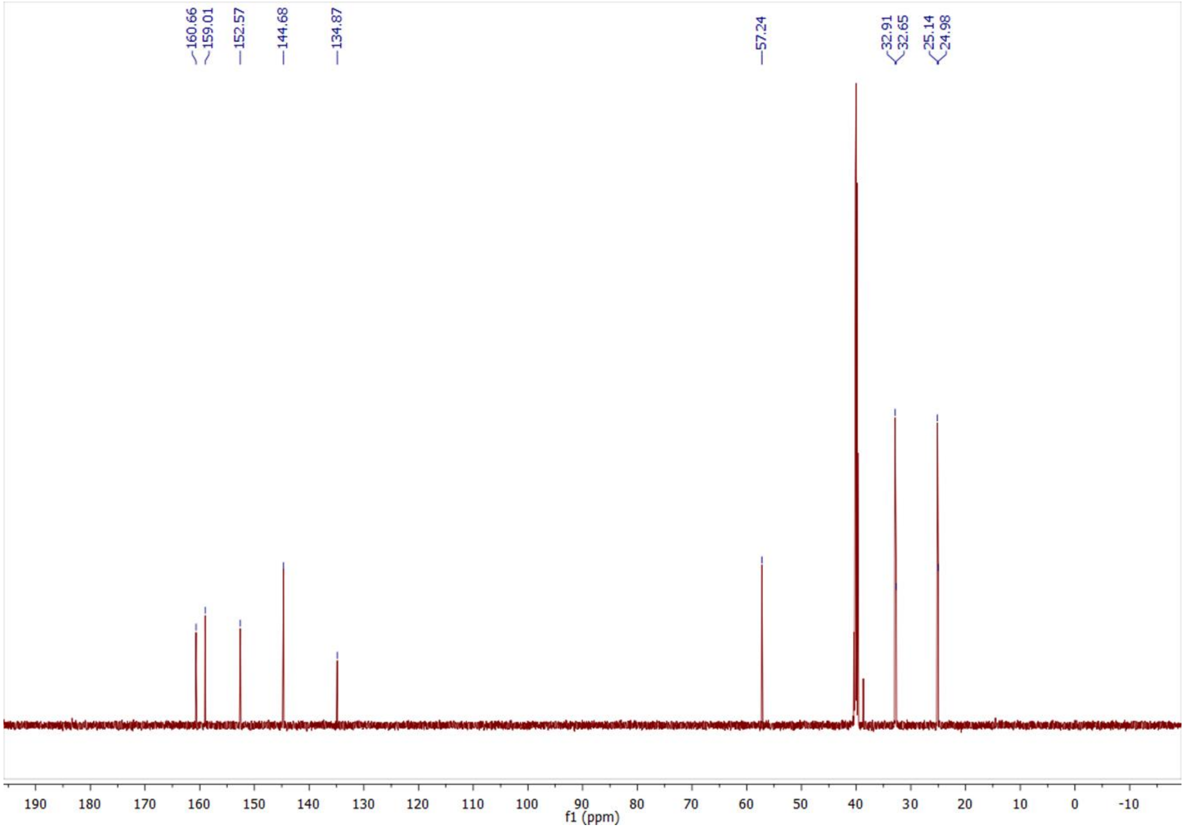

Figure S90. $^{13}C\ \{^{1}H\}$ NMR (151 MHz, DMSO-*d*6) spectrum of compound **2k**.

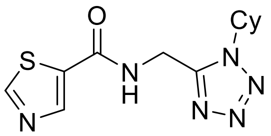

2k

N-((1-cyclohexyl-1*H*-tetrazol-5-yl)methyl)thiazole-5-carboxamide

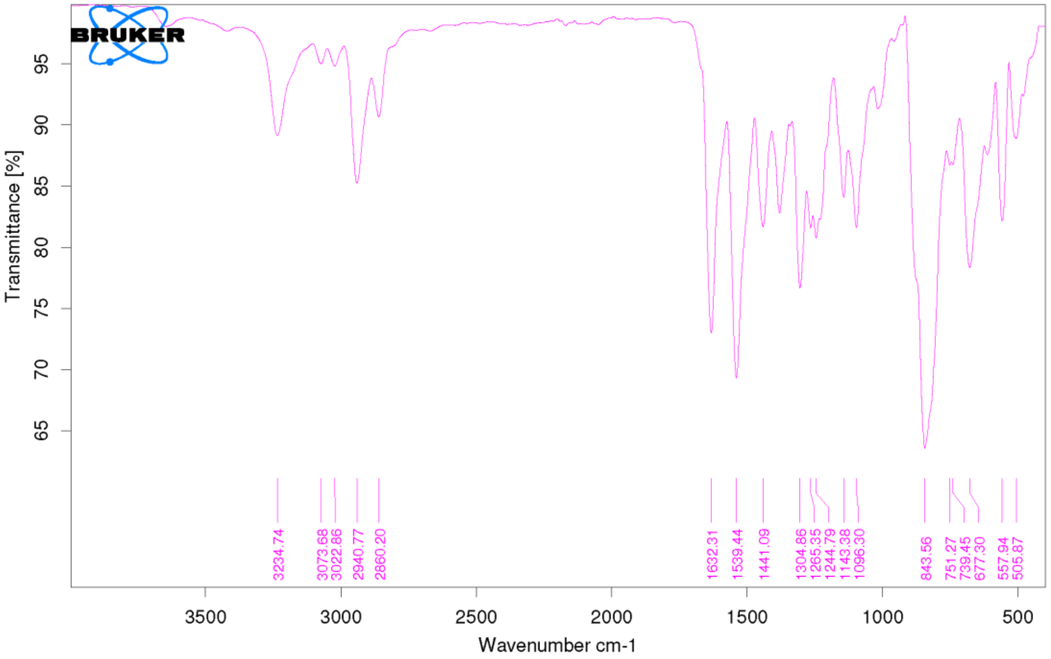

C:\Users\hvs\Documents\IR spectra\Erik Van Der Eycken\Felix\Gerardo\GM-073.0 | GM-073 | Instrument type and / or accessory | 1/21/2019

Page 1/1

Figure S91. FT-IR (KBr) spectrum of compound **2k**.

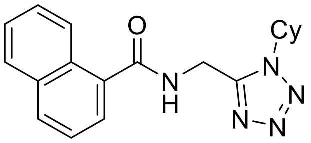

2l

N-((1-cyclohexyl-1*H*-tetrazol-5-yl)methyl)-1-naphthamide

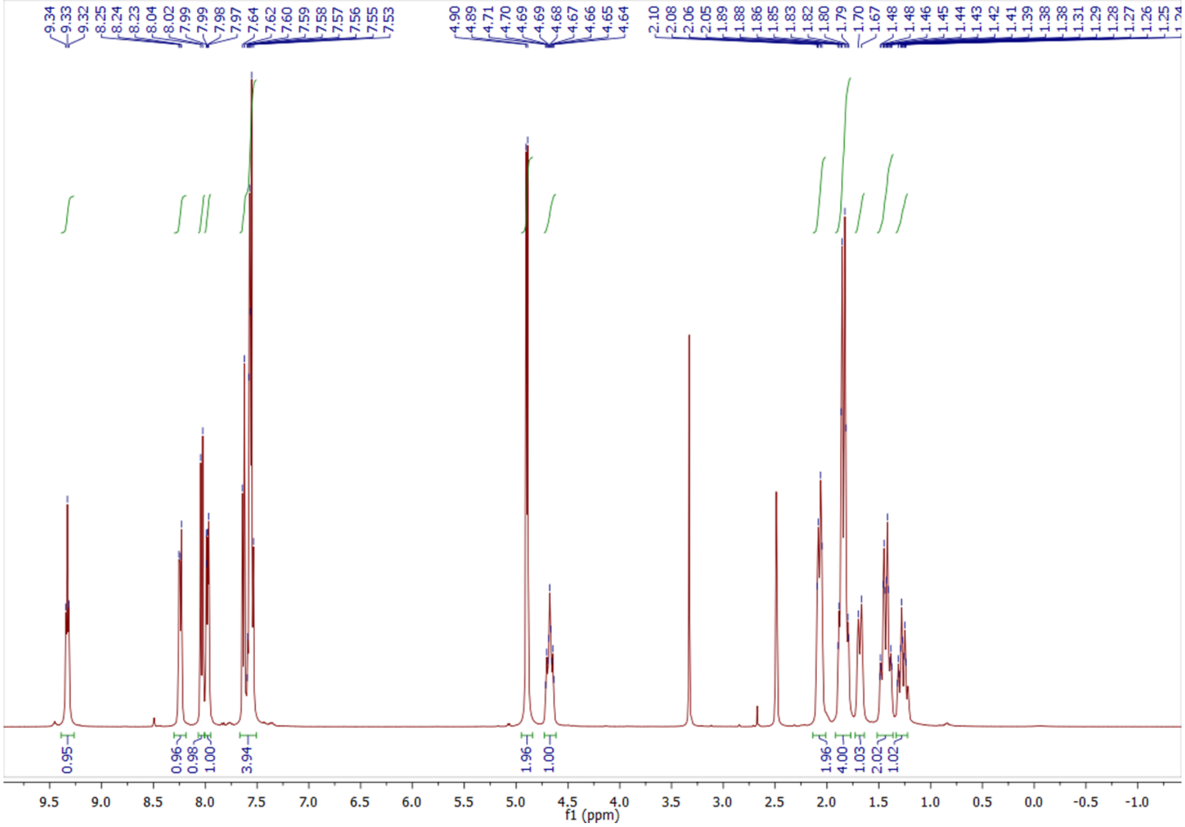

Figure S92. ^{1}H NMR (400 MHz, $CDCl_3$) spectrum of compound **2l**.

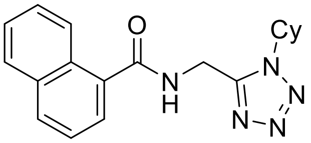

2l

N-((1-cyclohexyl-1*H*-tetrazol-5-yl)methyl)-1-naphthamide

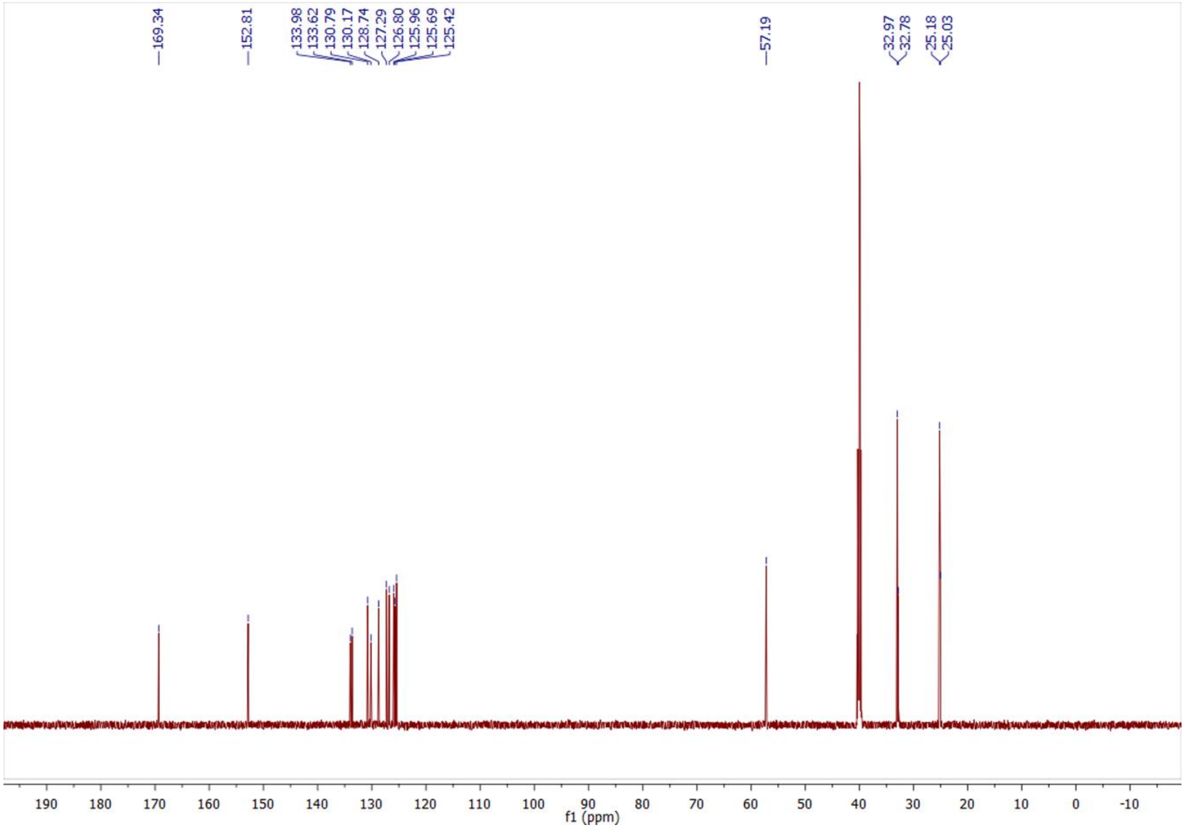

Figure S93. ^{13}C {^{1}H} NMR (151 MHz, DMSO-*d*6) spectrum of compound **2l**.

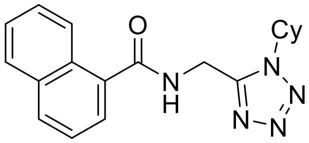

2l

N-((1-cyclohexyl-1*H*-tetrazol-5-yl)methyl)-1-naphthamide

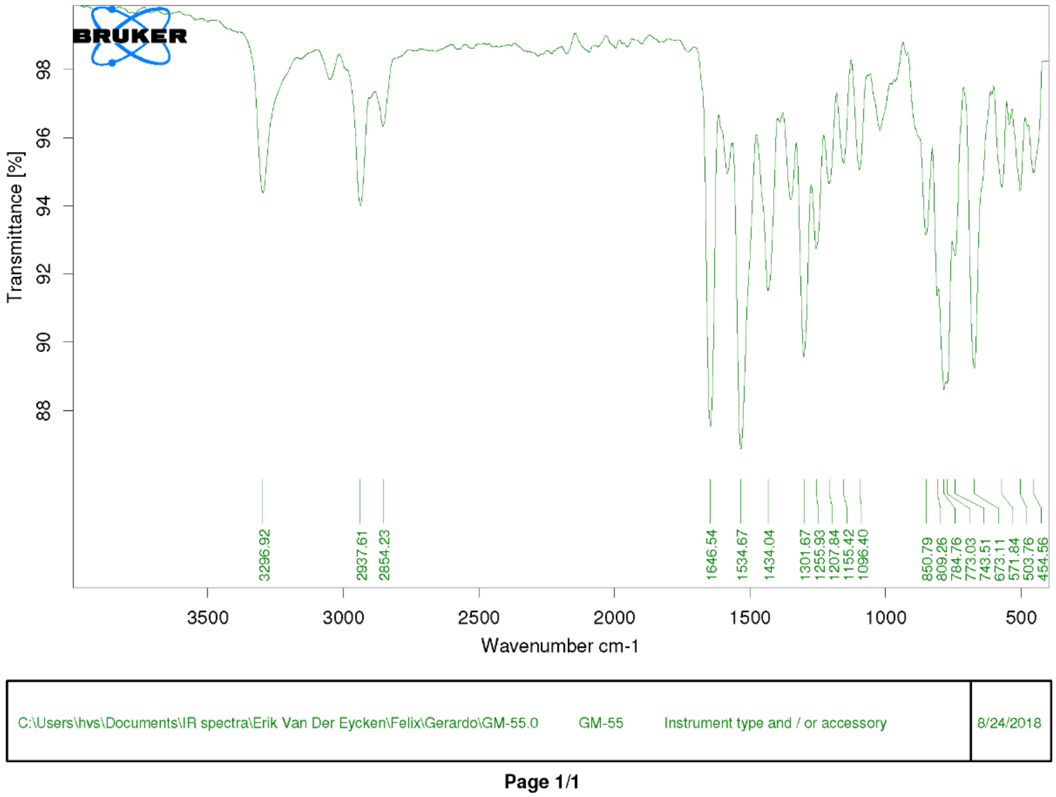

Figure S94. FT-IR (KBr) spectrum of compound **2l**.

IR and NMR spectra of tetrazole-isoquinolone/pyridone hybrids

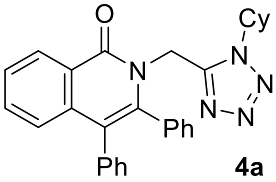

2-((1-cyclohexyl-1*H*-tetrazol-5-yl)methyl)-3,4-diphenylisoquinolin-1(2*H*)-one

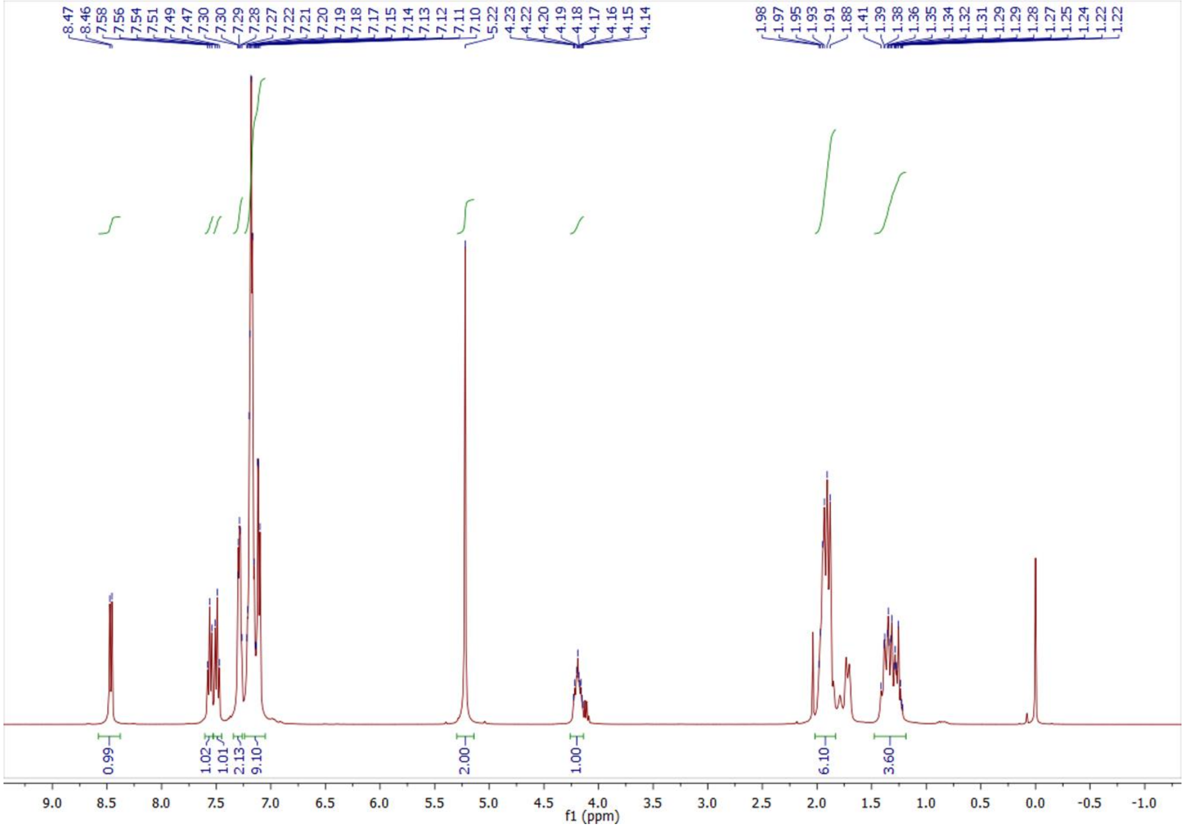

Figure S95. ^{1}H NMR (400 MHz, $CDCl_3$) spectrum of compound **4a**.

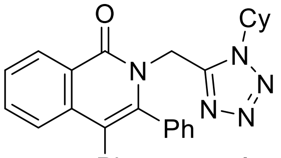

4a

2-((1-cyclohexyl-1*H*-tetrazol-5-yl)methyl)-3,4-diphenylisoquinolin-1(2*H*)-one

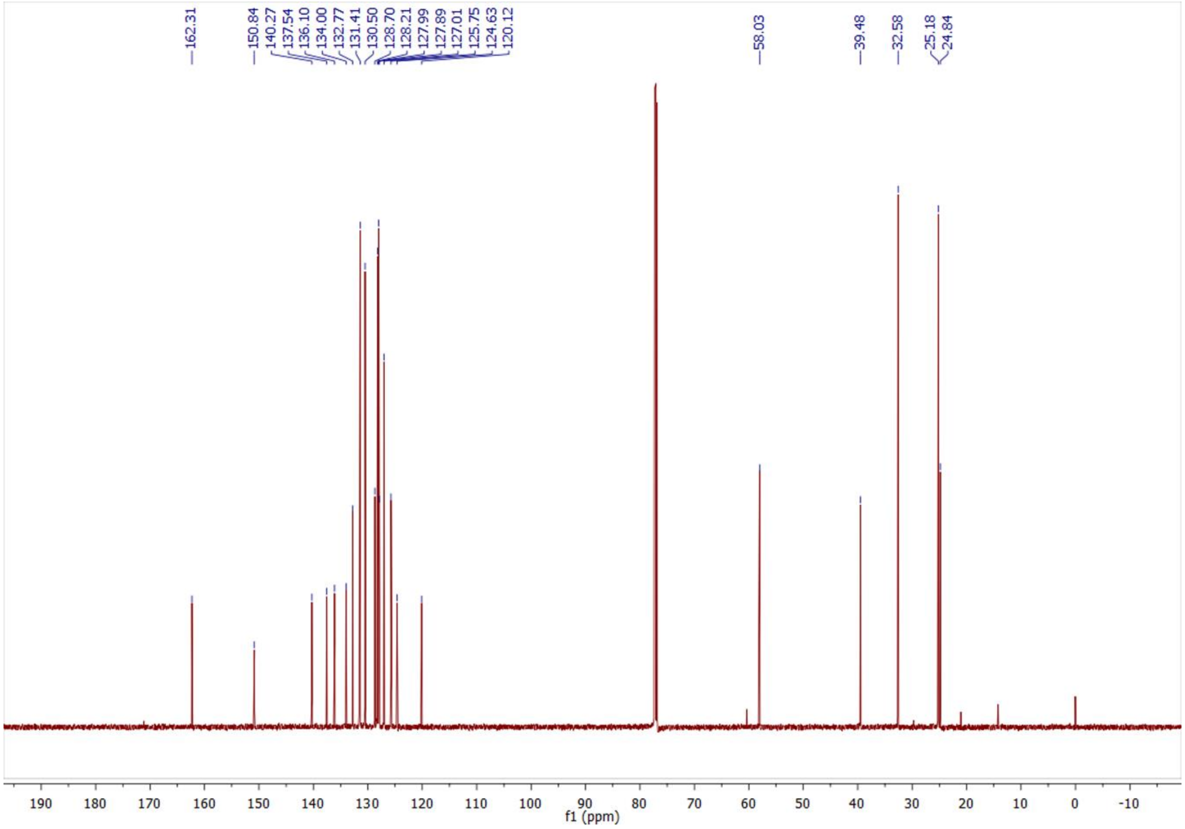

Figure S96. ^{13}C {^{1}H} NMR (151 MHz, $CDCl_3$) spectrum of compound **4a**.

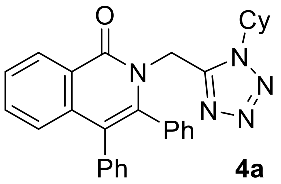

4a

2-((1-cyclohexyl-1*H*-tetrazol-5-yl)methyl)-3,4-diphenylisoquinolin-1(2*H*)-one

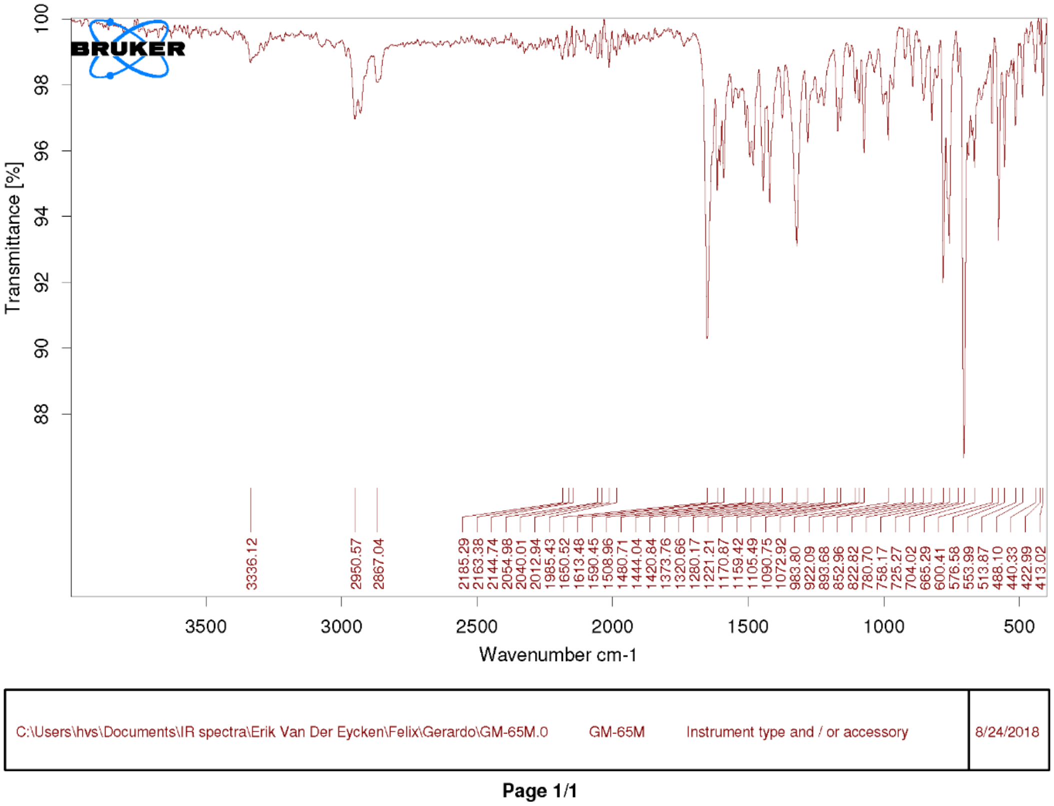

Figure S97. FT-IR (KBr) spectrum of compound **4a**.

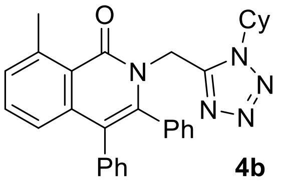

4b

2-((1-cyclohexyl-1*H*-tetrazol-5-yl)methyl)-8-methyl-3,4-diphenylisoquinolin-1(2*H*)-one

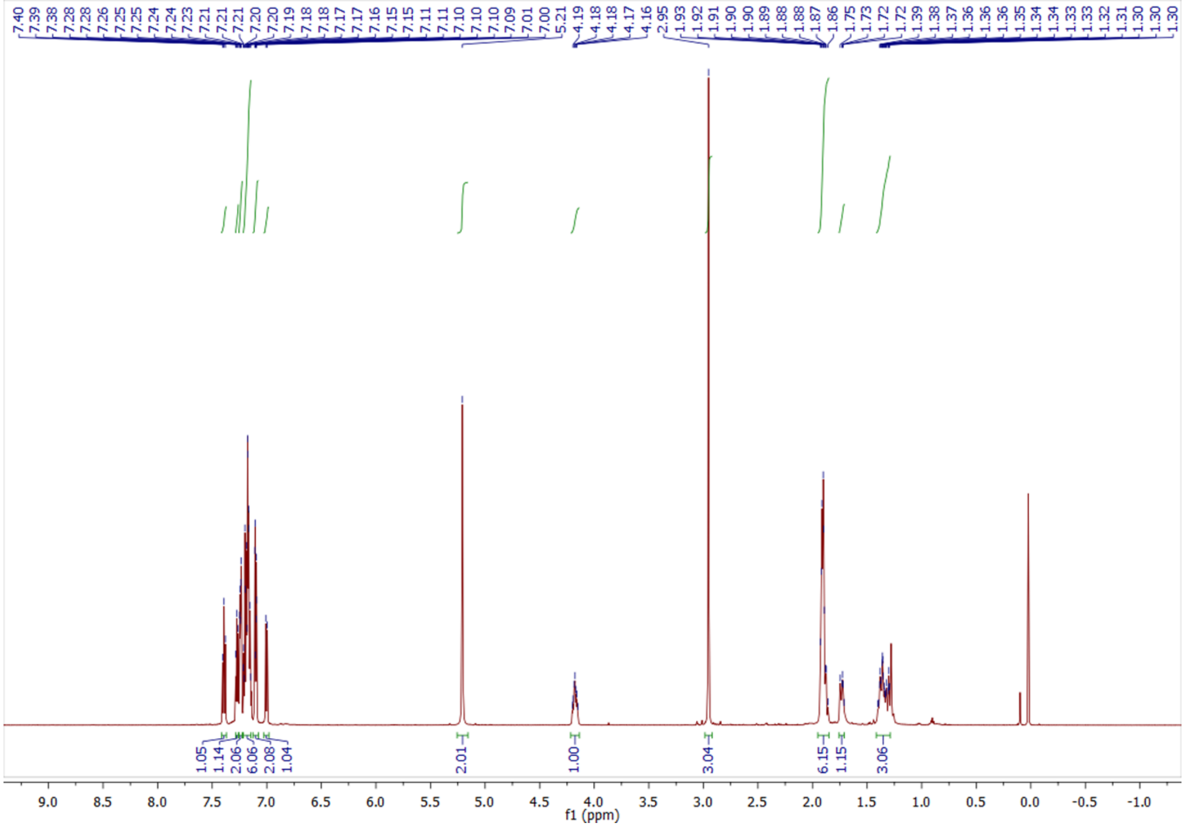

Figure S98. ^{1}H NMR (600 MHz, $CDCl_3$) spectrum of compound **4b**.

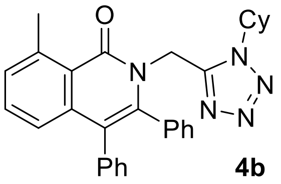

4b

2-((1-cyclohexyl-1*H*-tetrazol-5-yl)methyl)-8-methyl-3,4-diphenylisoquinolin-1(2*H*)-one

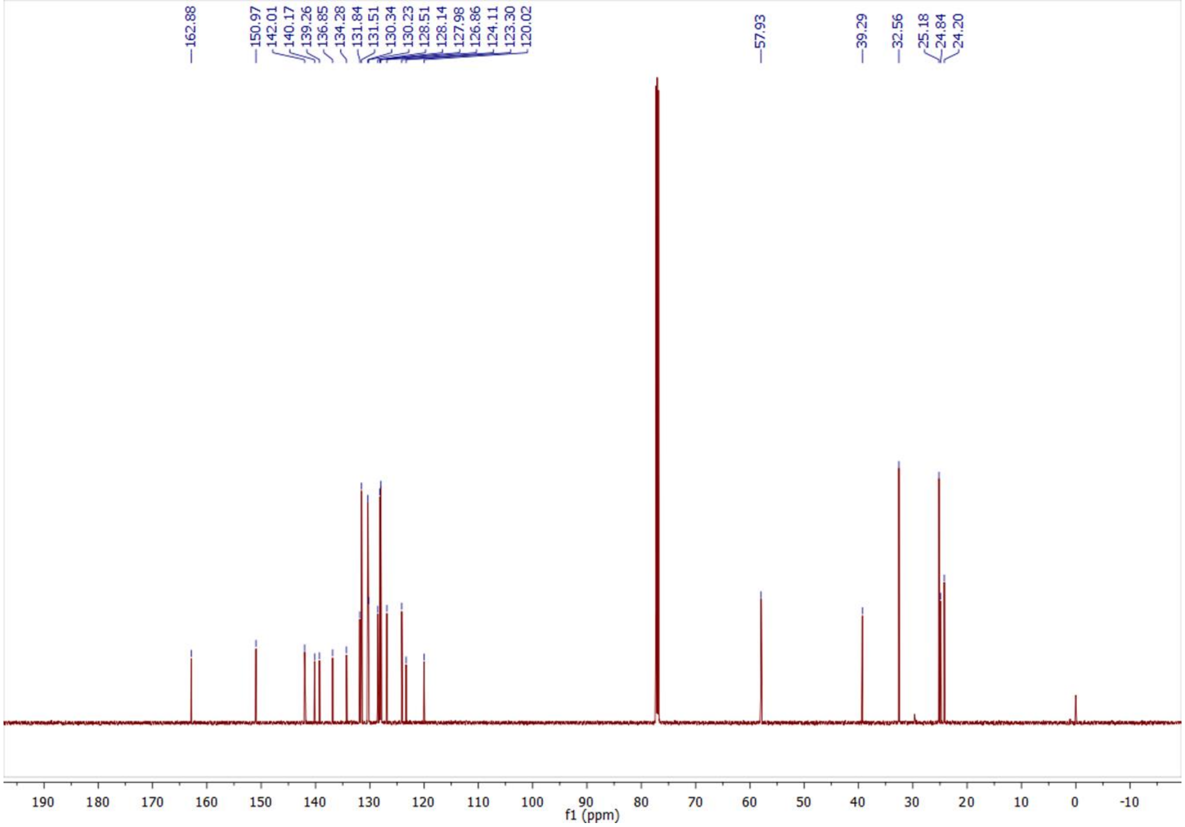

Figure S99. ^{13}C {^{1}H} NMR (151 MHz, $CDCl_3$) spectrum of compound **4b**.

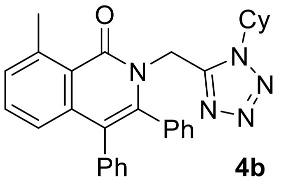

4b

2-((1-cyclohexyl-1*H*-tetrazol-5-yl)methyl)-8-methyl-3,4-diphenylisoquinolin-1(2*H*)-one

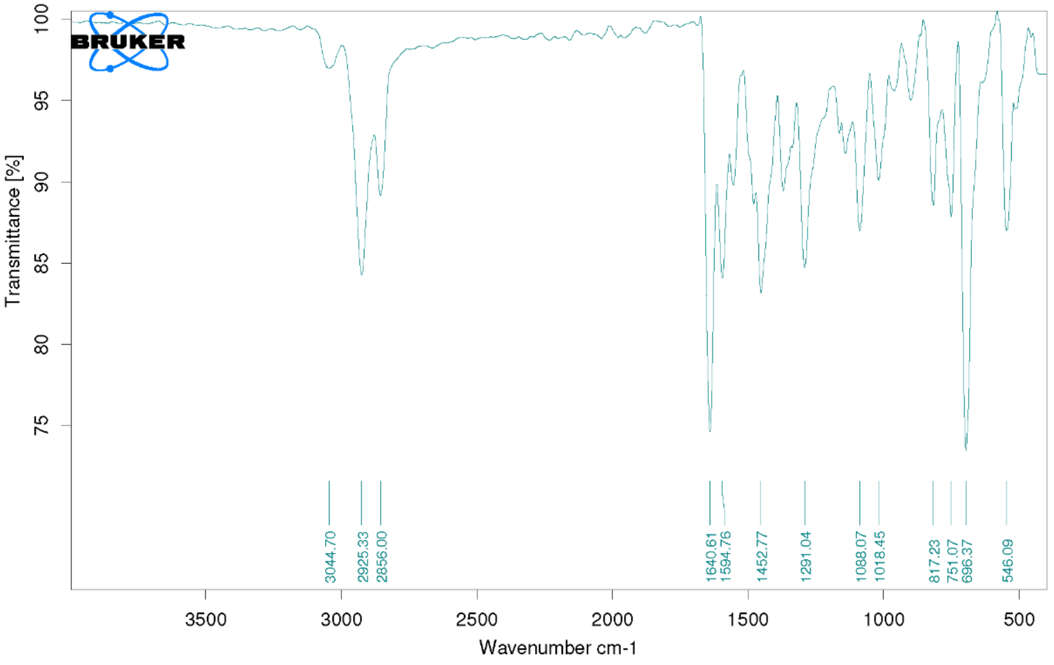

C:\Users\hvs\Documents\IR spectra\Erik Van Der Eycken\Felix\Gerardo\GM-094-A.0	GM-094-A	Instrument type and / or accessory	8/24/2018

Page 1/1

Figure S100. FT-IR (KBr) spectrum of compound **4b**.

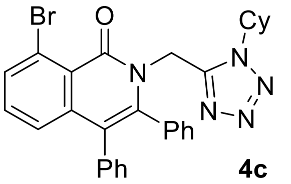

4c

8-bromo-2-((1-cyclohexyl-1*H*-tetrazol-5-yl)methyl)-3,4-diphenylisoquinolin-1(2*H*)-one

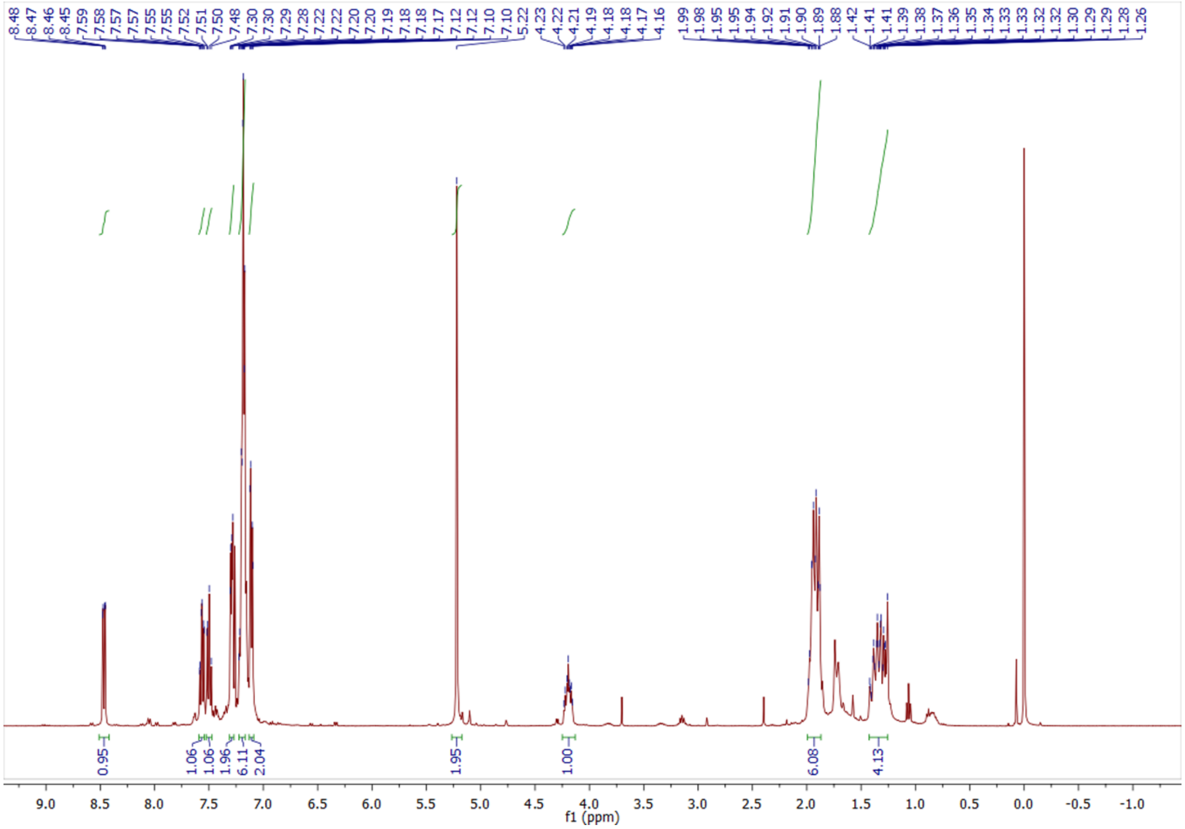

Figure S101. ^{1}H NMR (400 MHz, $CDCl_3$) spectrum of compound **4c**.

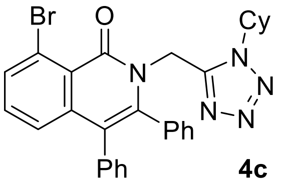

4c

8-bromo-2-((1-cyclohexyl-1*H*-tetrazol-5-yl)methyl)-3,4-diphenylisoquinolin-1(2*H*)-one

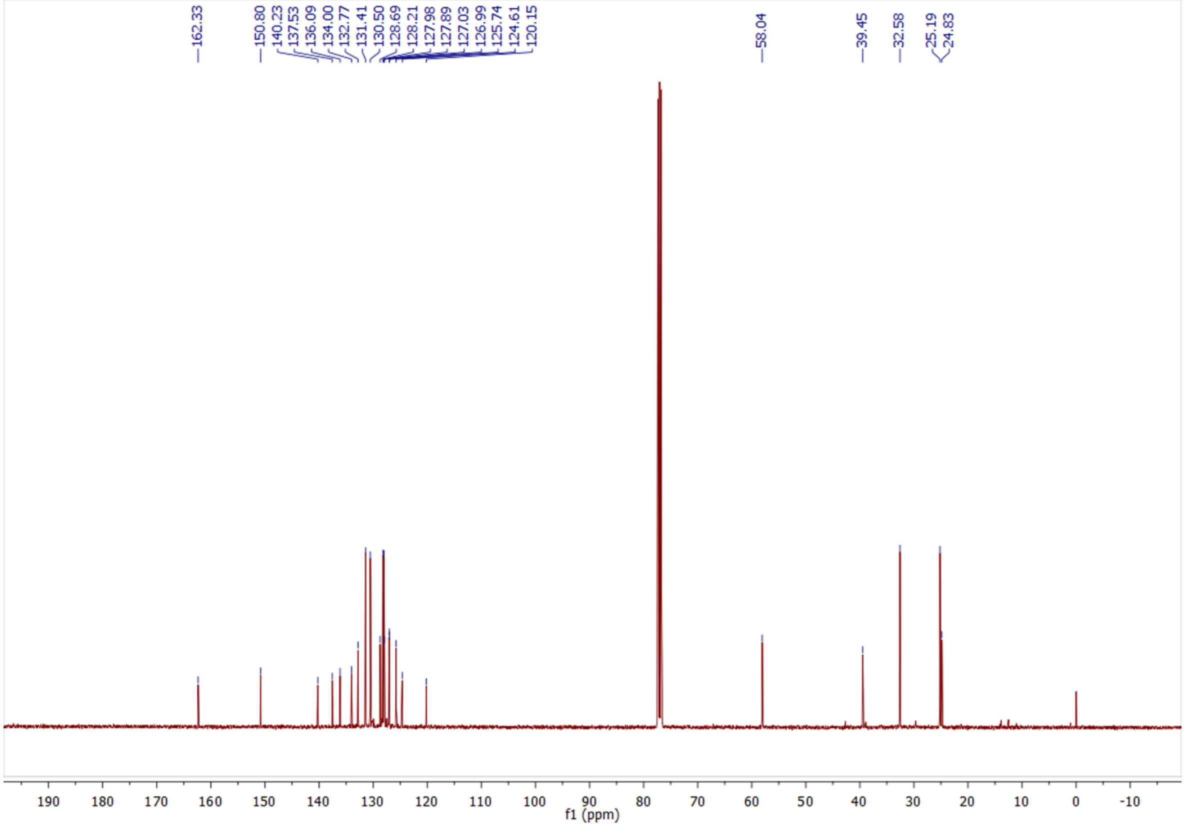

Figure S102. ^{13}C {^{1}H} NMR (101 MHz, $CDCl_3$) spectrum of compound **4c**.

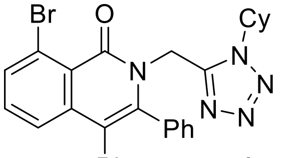

4c

8-bromo-2-((1-cyclohexyl-1*H*-tetrazol-5-yl)methyl)-3,4-diphenylisoquinolin-1(2*H*)-one

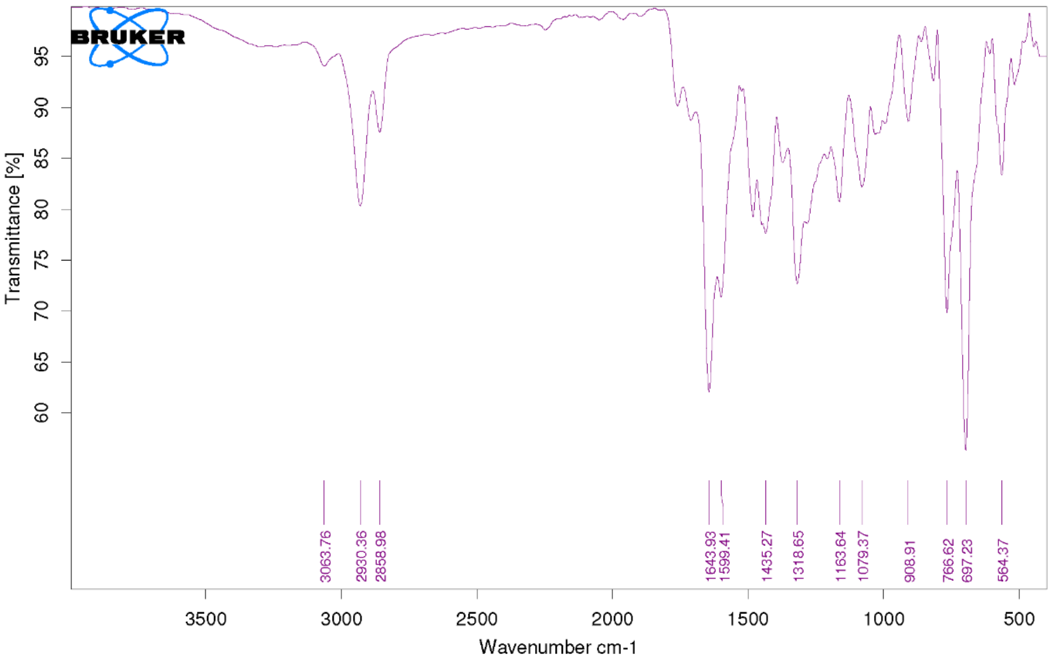

C:\Users\hvs\Documents\IR spectra\Erik Van Der Eycken\Felix\Gerardo\GM-100.0 GM-100 Instrument type and / or accessory	8/24/2018

Page 1/1

Figure S103. FT-IR (KBr) spectrum of compound **4c**.

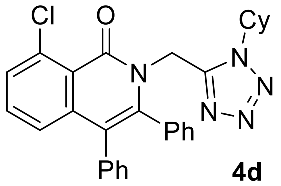

4d

8-chloro-2-((1-cyclohexyl-1*H*-tetrazol-5-yl)methyl)-3,4-diphenylisoquinolin-1(2*H*)-one

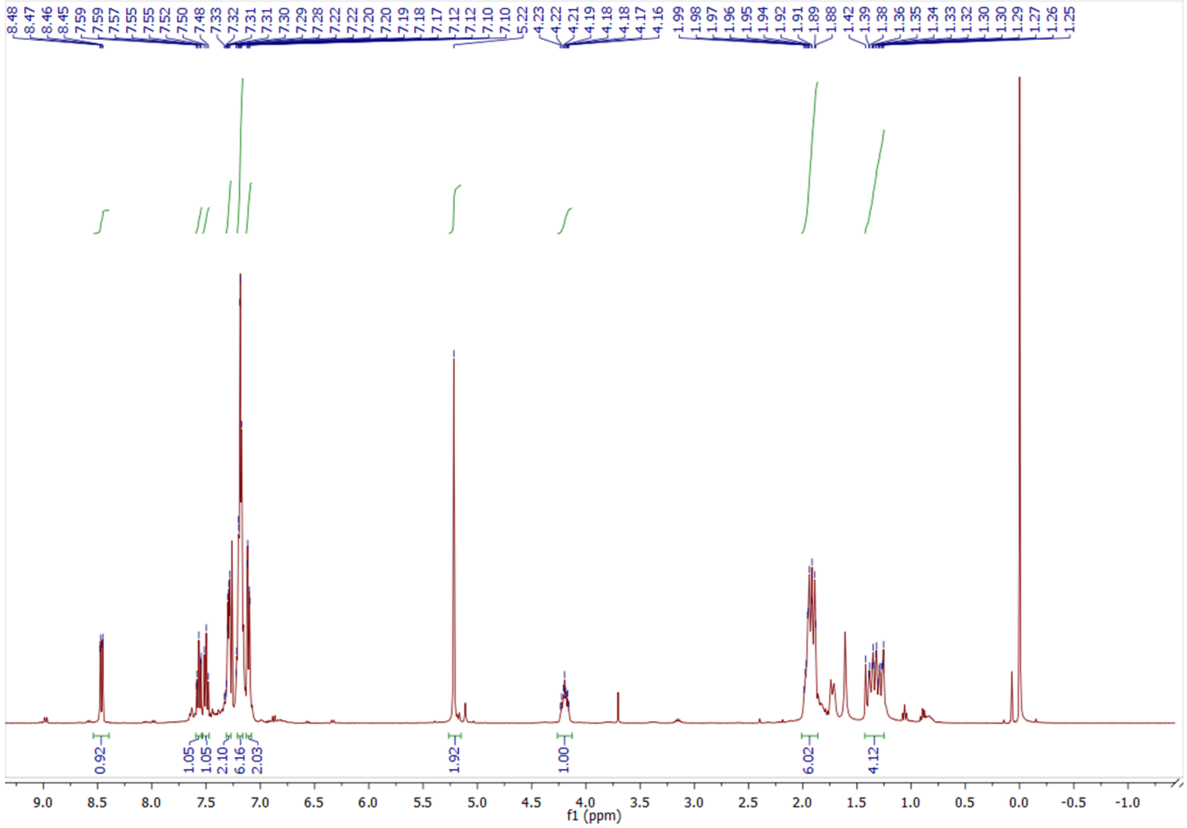

Figure S104. ^{1}H NMR (400 MHz, $CDCl_3$) spectrum of compound **4d**.

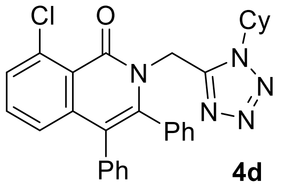

4d

8-chloro-2-((1-cyclohexyl-1*H*-tetrazol-5-yl)methyl)-3,4-diphenylisoquinolin-1(2*H*)-one

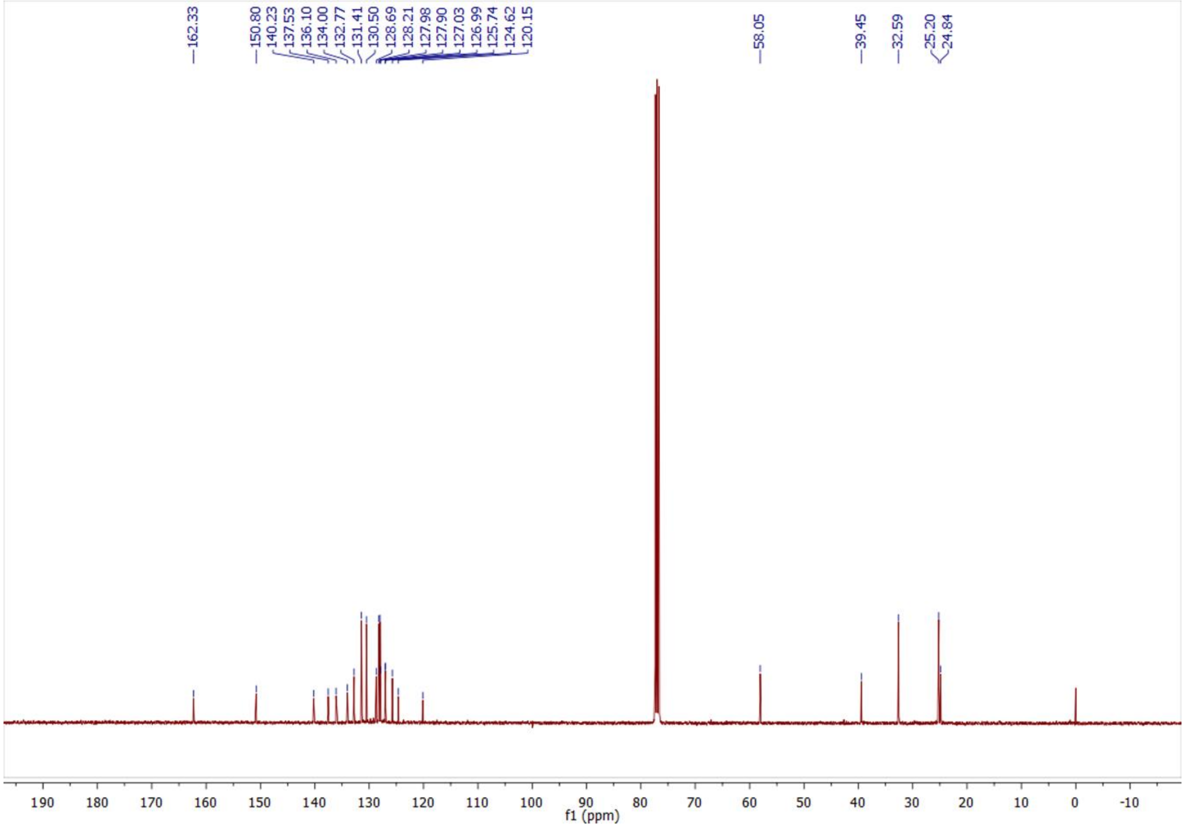

Figure S105. $^{13}C\ \{^1H\}$ NMR (101 MHz, $CDCl_3$) spectrum of compound **4d**.

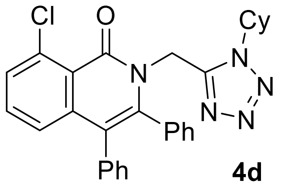

4d

8-chloro-2-((1-cyclohexyl-1*H*-tetrazol-5-yl)methyl)-3,4-diphenylisoquinolin-1(2*H*)-one

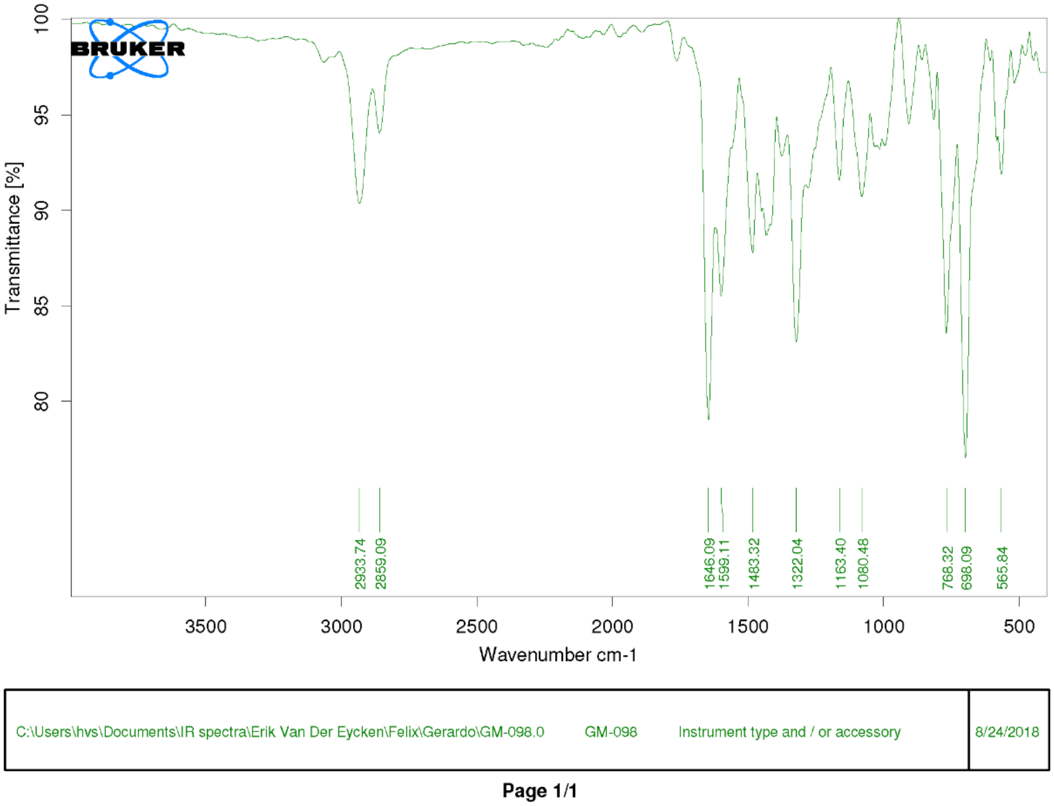

Figure S106. FT-IR (KBr) spectrum of compound **4d**.

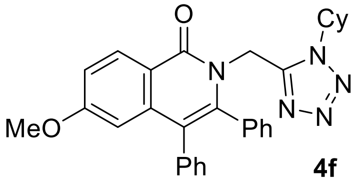

4f

2-((1-cyclohexyl-1*H*-tetrazol-5-yl)methyl)-6-methoxy-3,4-diphenylisoquinolin-1(2*H*)-one

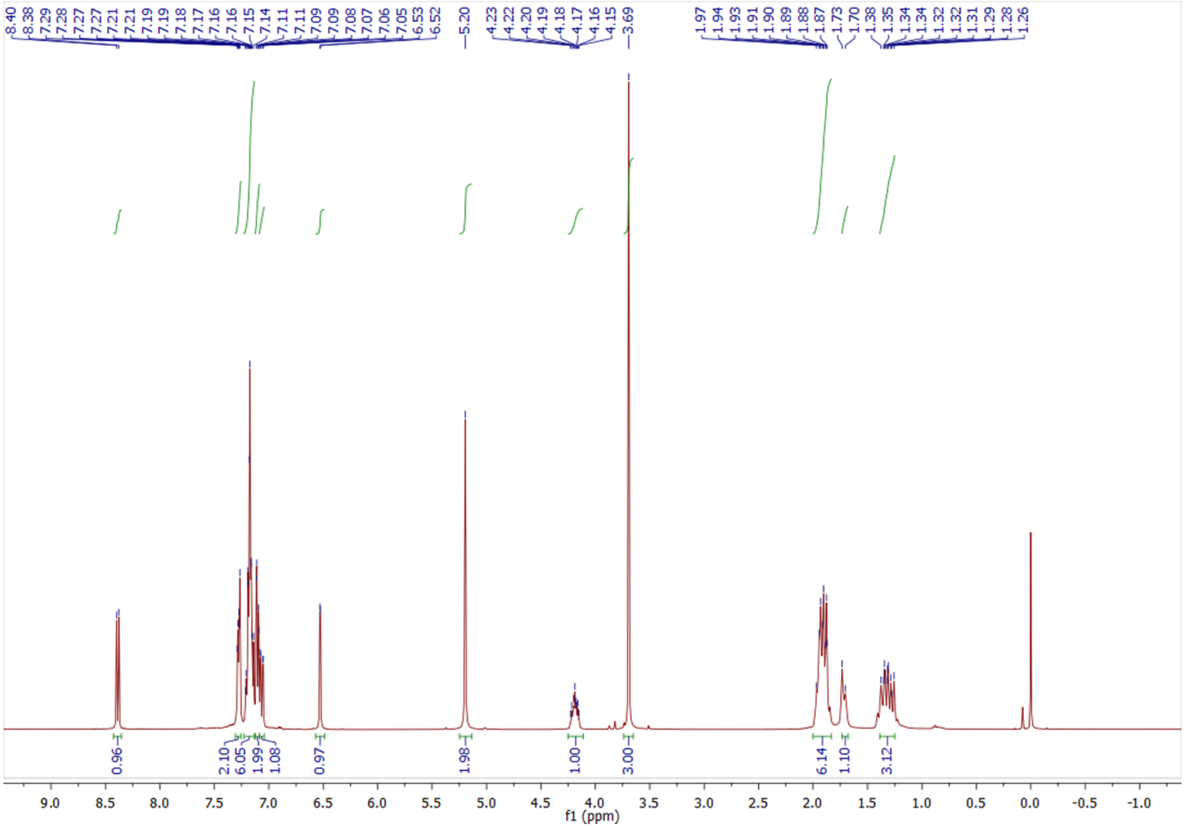

Figure S107. ^{1}H NMR (400 MHz, $CDCl_3$) spectrum of compound **4f**.

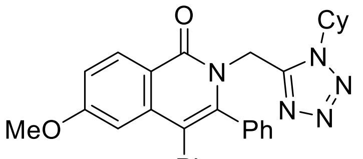

4f

2-((1-cyclohexyl-1*H*-tetrazol-5-yl)methyl)-6-methoxy-3,4-diphenylisoquinolin-1(2*H*)-one

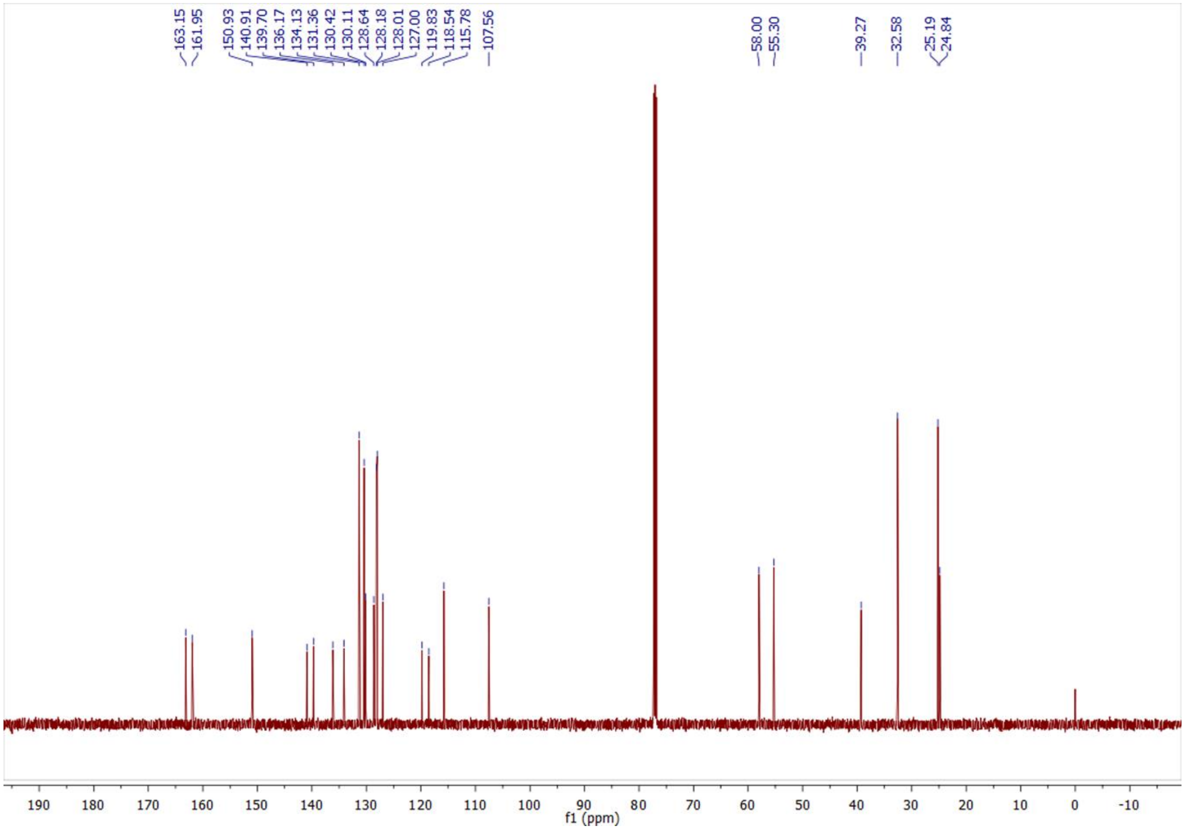

Figure S108. $^{13}C\ \{^{1}H\}$ NMR (151 MHz, $CDCl_3$) spectrum of compound **4f**.

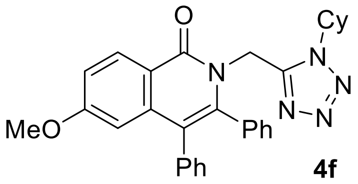

4f

2-((1-cyclohexyl-1*H*-tetrazol-5-yl)methyl)-6-methoxy-3,4-diphenylisoquinolin-1(2*H*)-one

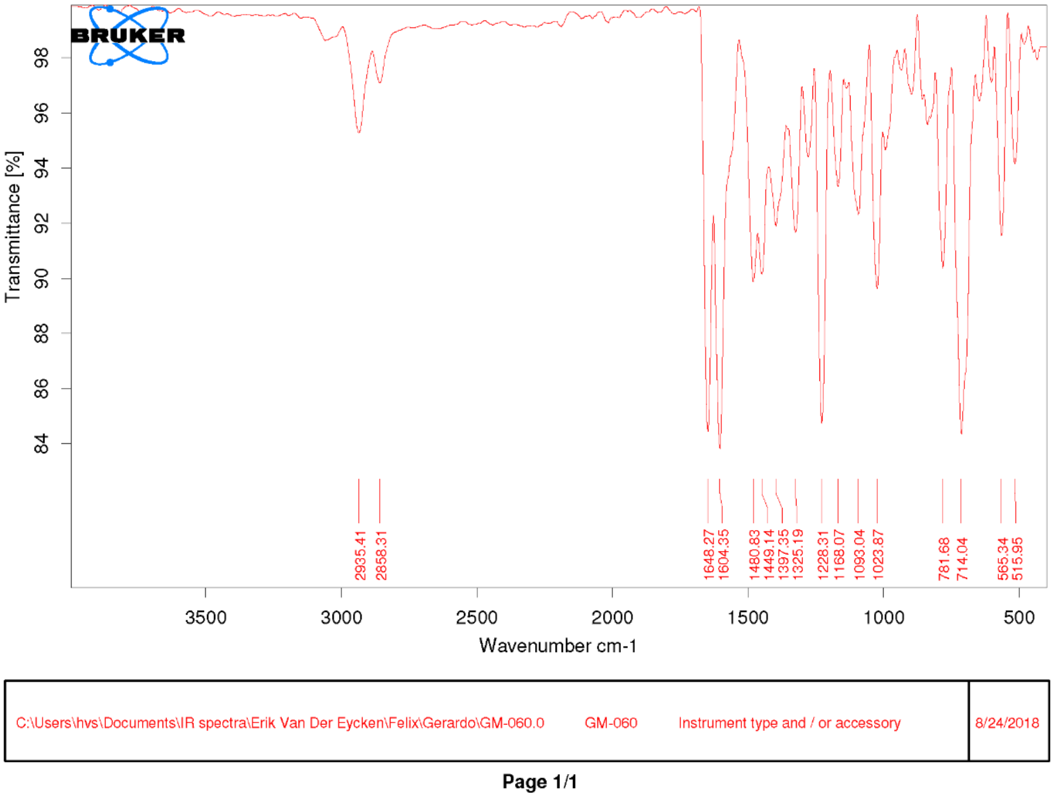

Figure S109. FT-IR (KBr) spectrum of compound **4f**.

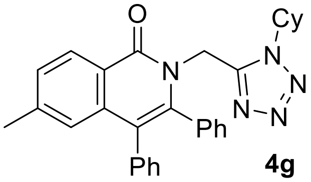

4g

2-((1-cyclohexyl-1*H*-tetrazol-5-yl)methyl)-6-methyl-3,4-diphenylisoquinolin-1(2*H*)-one

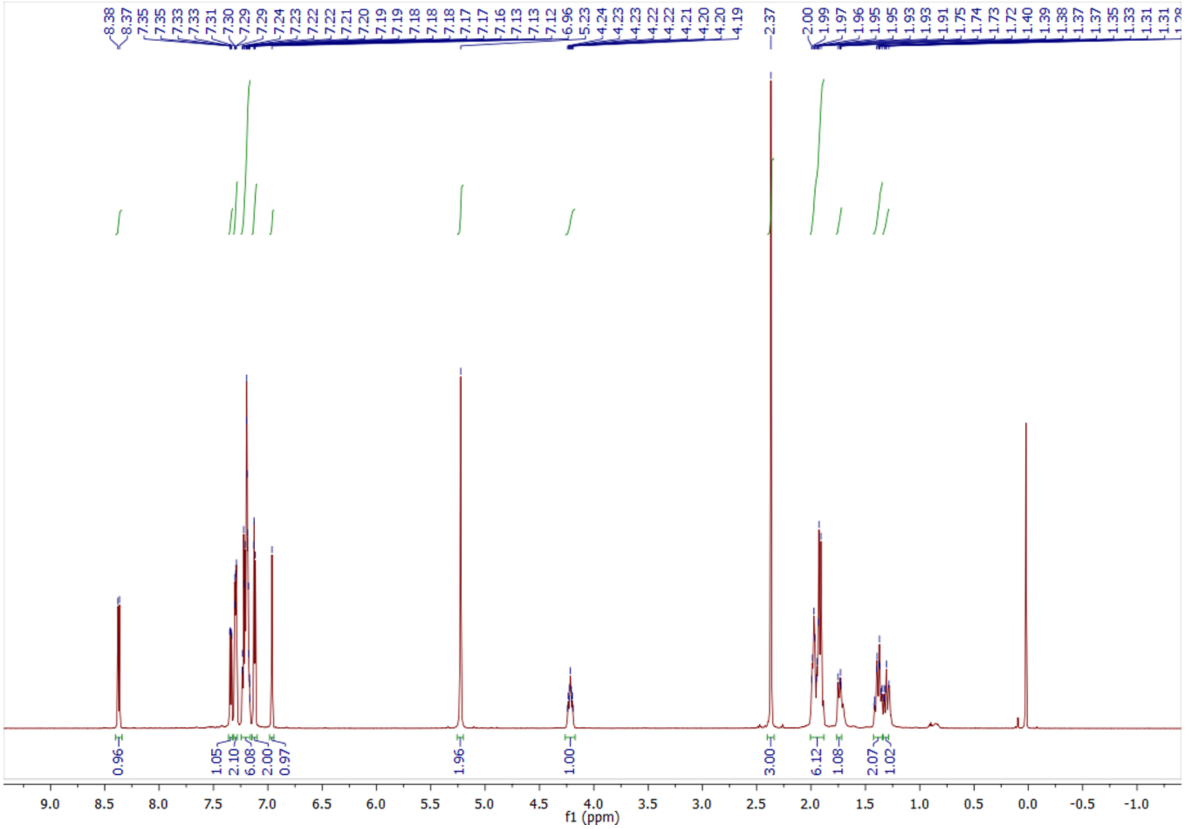

Figure S110. ^{1}H NMR (600 MHz, $CDCl_3$) spectrum of compound **4g**.

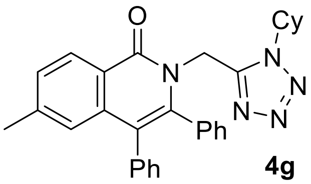

4g

2-((1-cyclohexyl-1*H*-tetrazol-5-yl)methyl)-6-methyl-3,4-diphenylisoquinolin-1(2*H*)-one

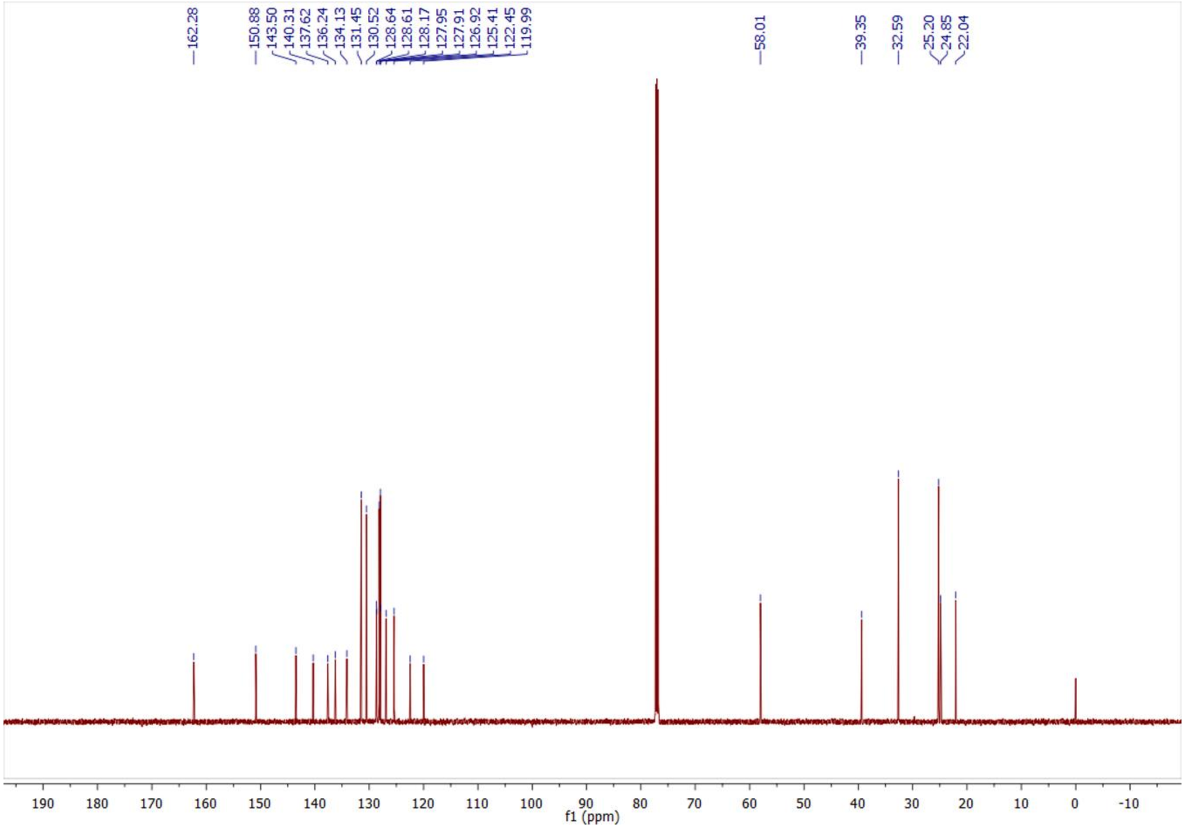

Figure S111. $^{13}C\ \{^{1}H\}$ NMR (151 MHz, $CDCl_3$) spectrum of compound **4g**.

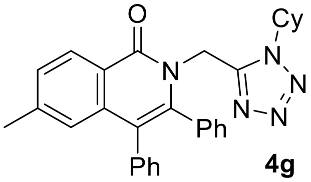

4g

2-((1-cyclohexyl-1*H*-tetrazol-5-yl)methyl)-6-methyl-
3,4-diphenylisoquinolin-1(2*H*)-one

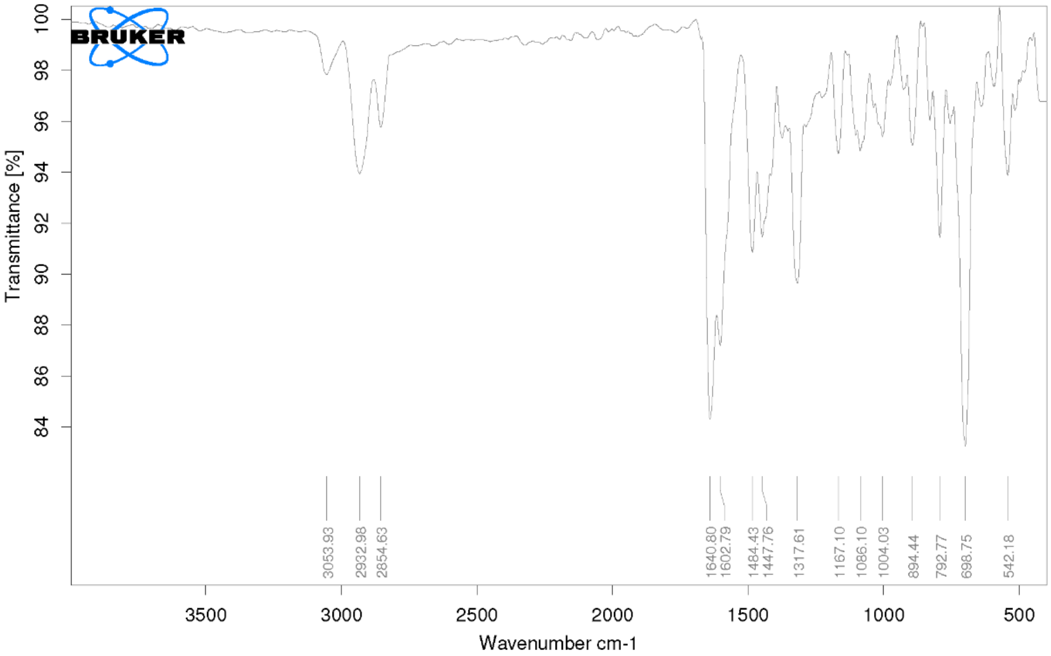

C:\Users\hvs\Documents\IR spectra\Erik Van Der Eycken\Felix\Gerardo\GM-058.0	GM-058	Instrument type and / or accessory	8/24/2018

Page 1/1

Figure S112. FT-IR (KBr) spectrum of compound **4g**.

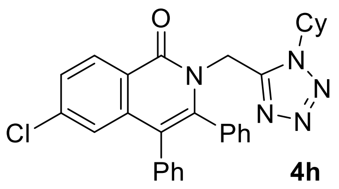

4h

6-chloro-2-((1-cyclohexyl-1*H*-tetrazol-5-yl)methyl)-
3,4-diphenylisoquinolin-1(2*H*)-one

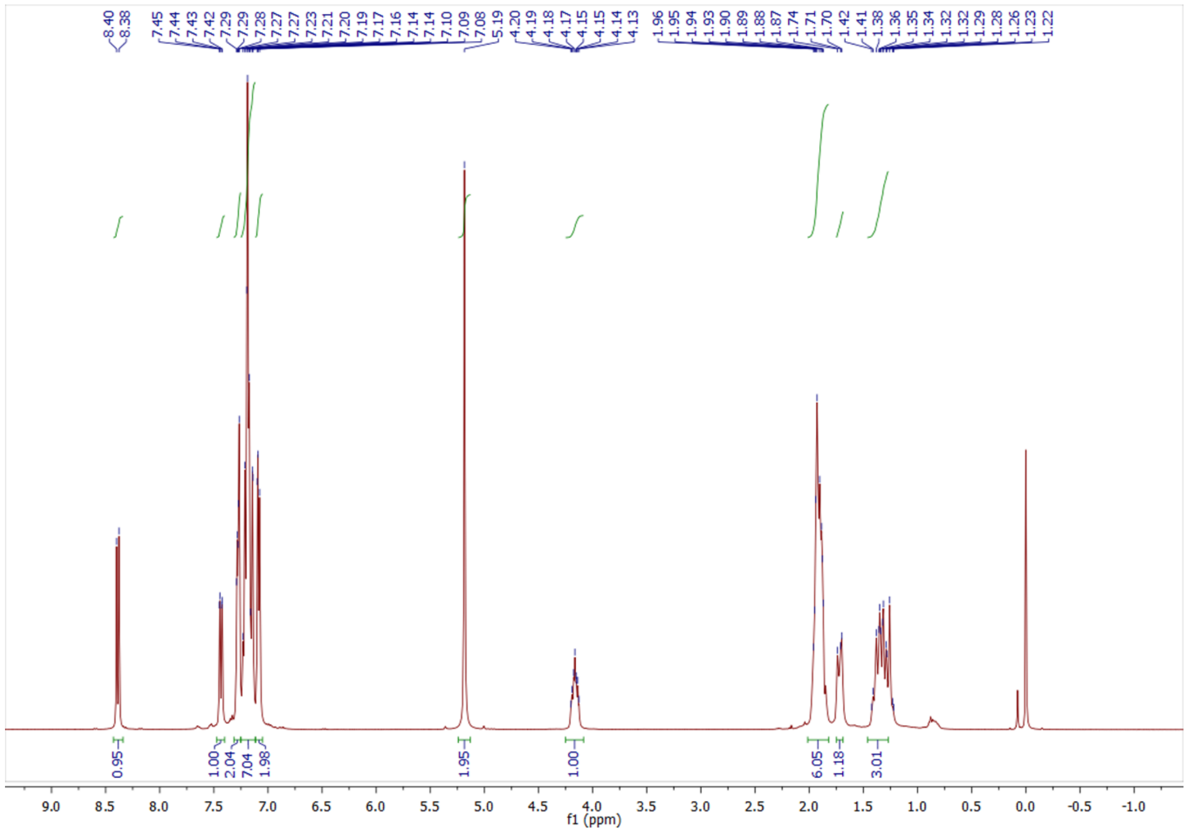

Figure S113. ^{1}H NMR (400 MHz, $CDCl_3$) spectrum of compound **4h**.

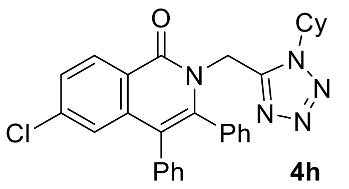

4h

6-chloro-2-((1-cyclohexyl-1*H*-tetrazol-5-yl)methyl)-
3,4-diphenylisoquinolin-1(2*H*)-one

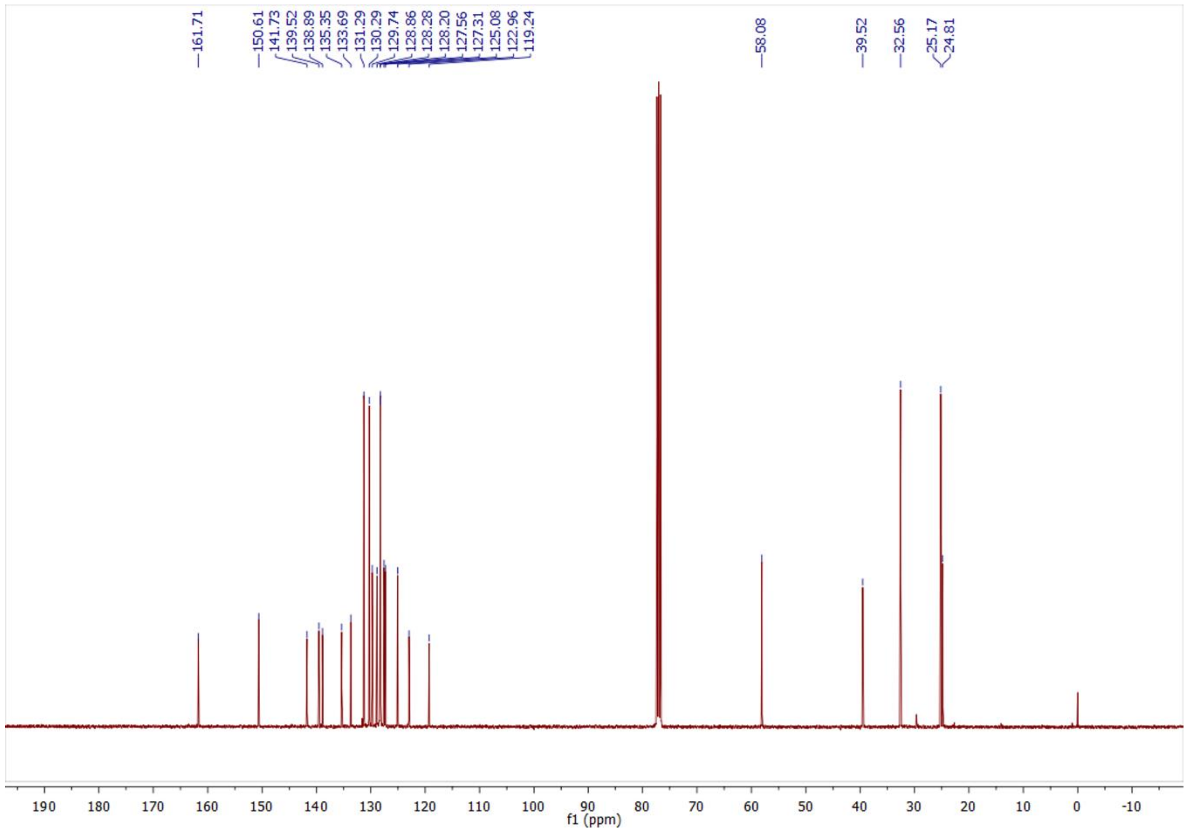

Figure S114. $^{13}C\ \{^1H\}$ NMR (101 MHz, $CDCl_3$) spectrum of compound **4h**.

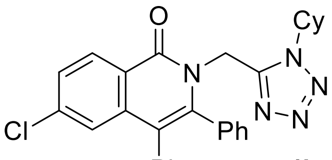

4h

6-chloro-2-((1-cyclohexyl-1*H*-tetrazol-5-yl)methyl)-
3,4-diphenylisoquinolin-1(2*H*)-one

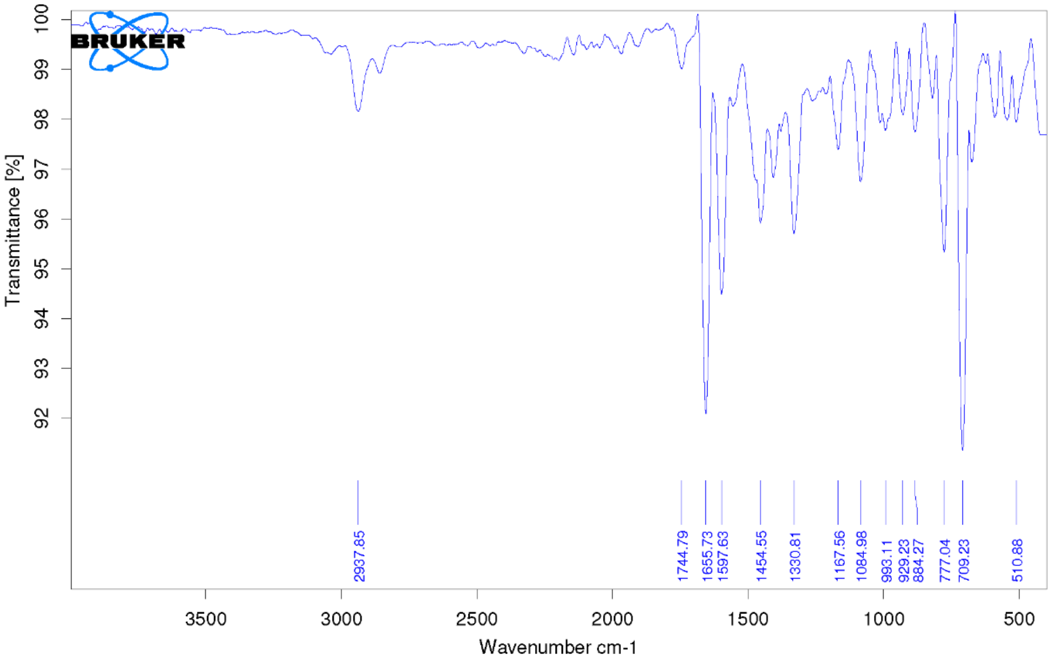

C:\Users\hvs\Documents\IR spectra\Erik Van Der Eycken\Felix\Gerardo\GM-62.0 GM-62 Instrument type and / or accessory	8/24/2018

Page 1/1

Figure S115. FT-IR (KBr) spectrum of compound **4h**.

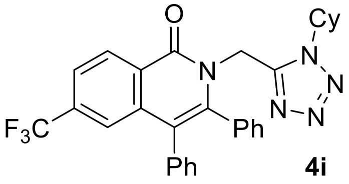

4i

2-((1-cyclohexyl-1*H*-tetrazol-5-yl)methyl)-3,4-diphenyl-6-(trifluoromethyl)isoquinolin-1(2*H*)-one

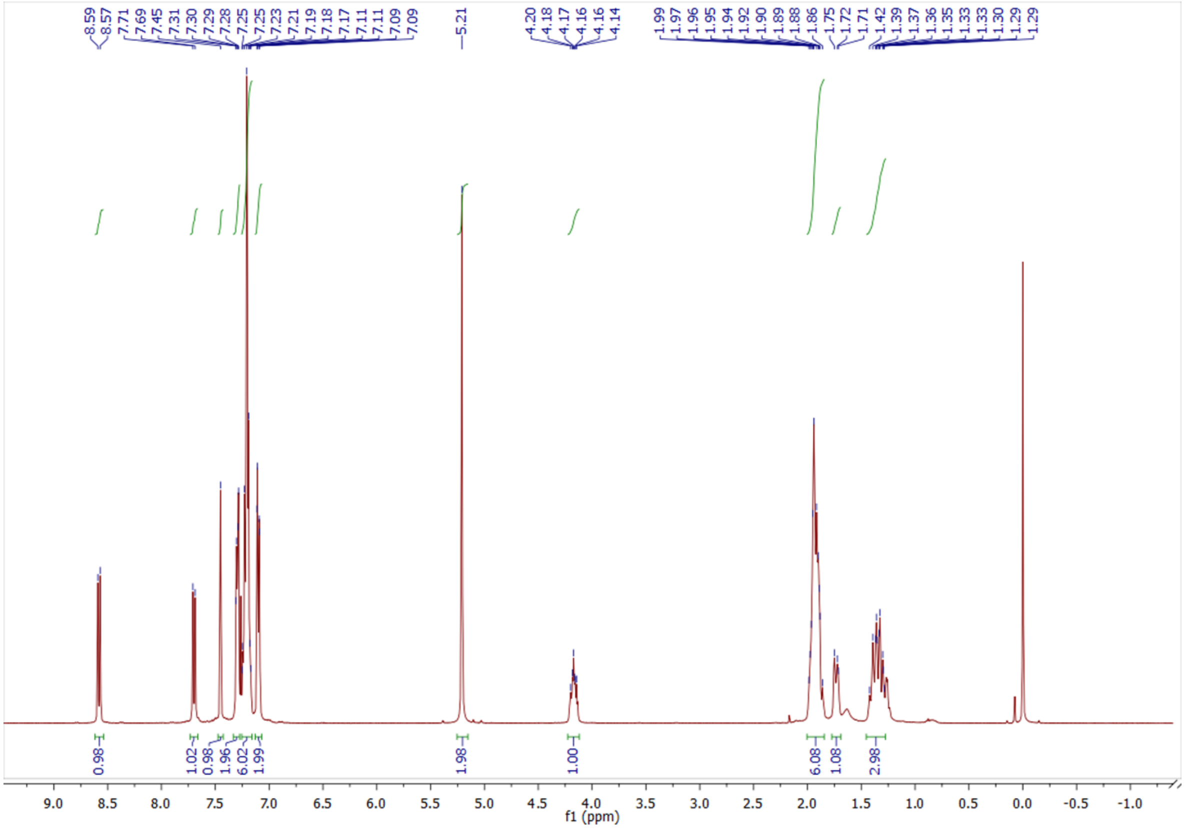

Figure S116. ^{1}H NMR (400 MHz, $CDCl_3$) spectrum of compound **4i**.

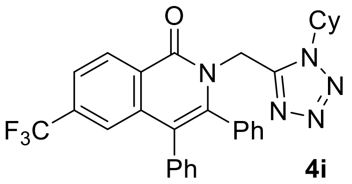

4i

2-((1-cyclohexyl-1*H*-tetrazol-5-yl)methyl)-3,4-diphenyl-6-(trifluoromethyl)isoquinolin-1(2*H*)-one

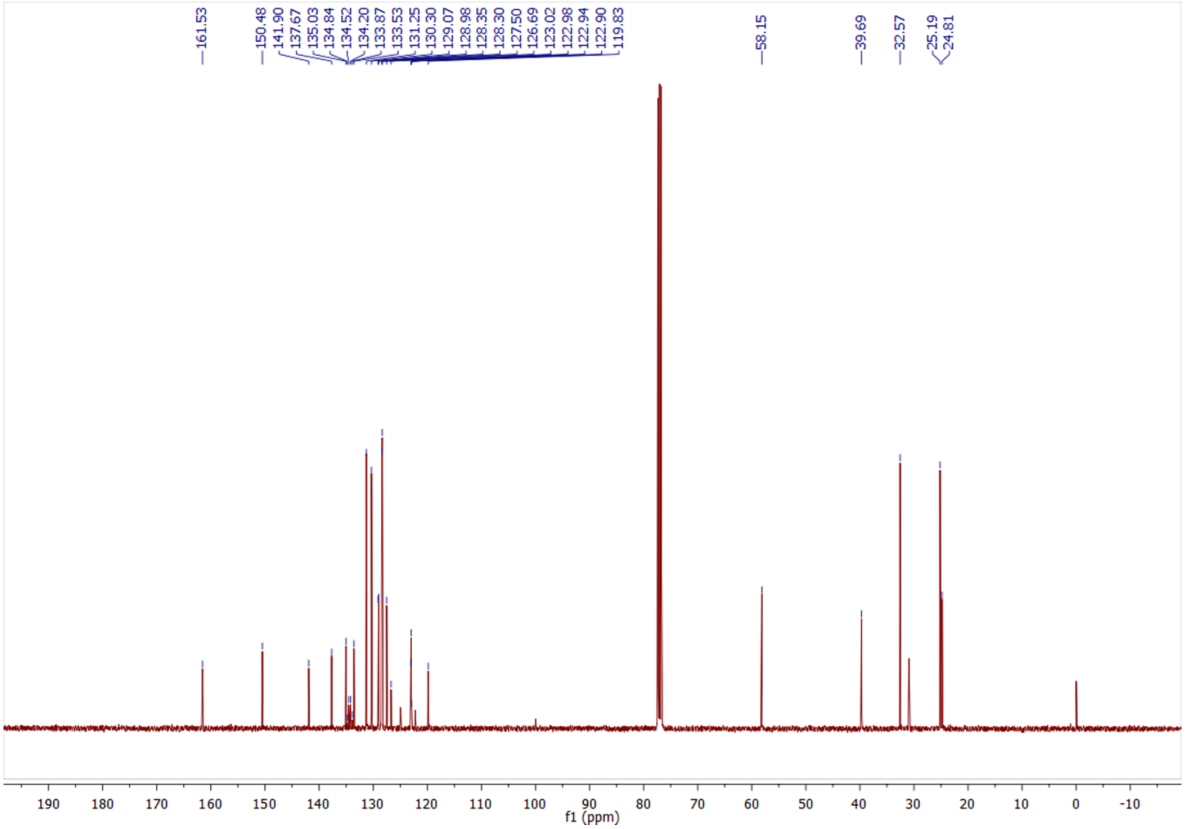

Figure S117. $^{13}C\ \{^{1}H\}$ NMR (101 MHz, $CDCl_3$) spectrum of compound **4i**.

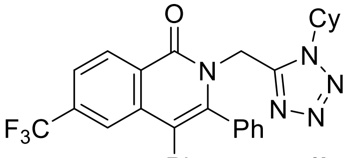

4i

2-((1-cyclohexyl-1*H*-tetrazol-5-yl)methyl)-3,4-diphenyl-6-(trifluoromethyl)isoquinolin-1(2*H*)-one

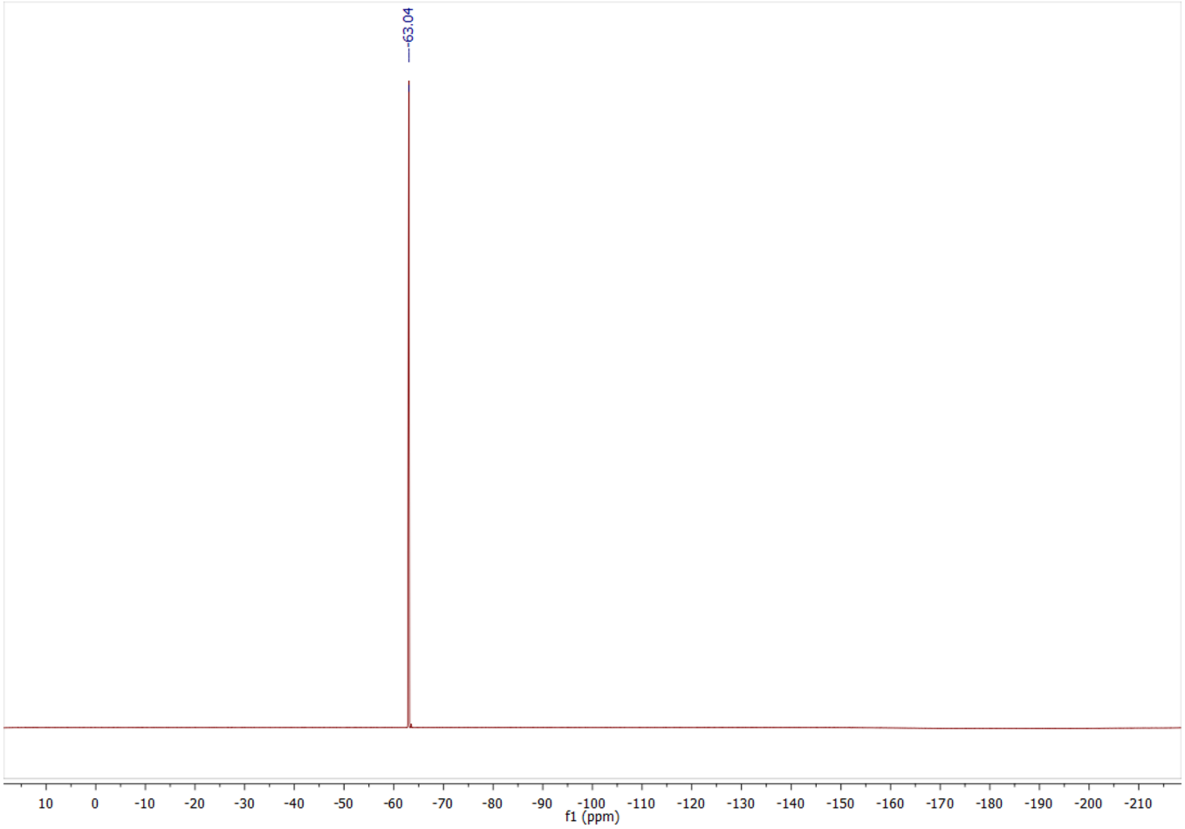

Figure S118. ^{19}F {^{13}C} NMR (376 MHz, $CDCl_3$) spectrum of compound **4i**.

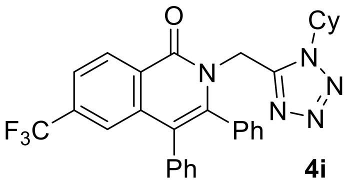

4i

2-((1-cyclohexyl-1*H*-tetrazol-5-yl)methyl)-3,4-diphenyl-6-(trifluoromethyl)isoquinolin-1(2*H*)-one

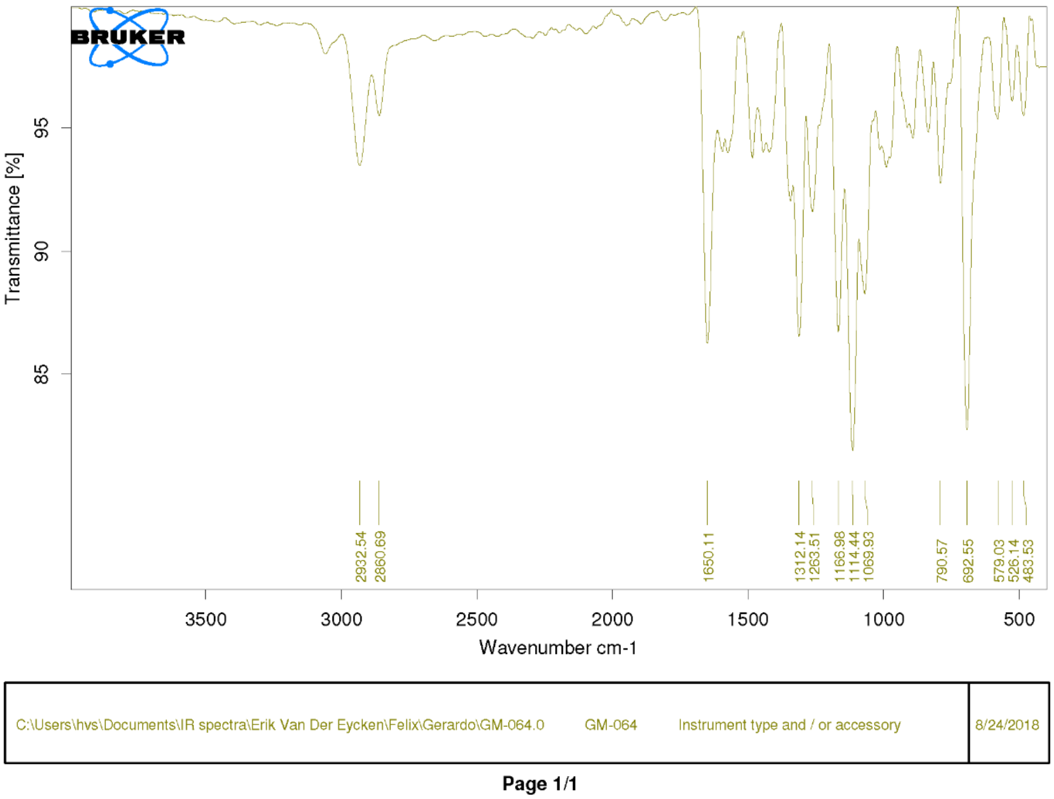

Figure S119. FT-IR (KBr) spectrum of compound **4i**.

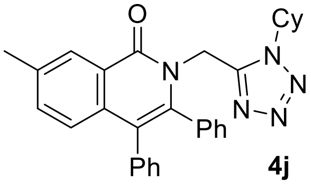

4j

2-((1-cyclohexyl-1*H*-tetrazol-5-yl)methyl)-7-methyl-3,4-diphenylisoquinolin-1(2*H*)-one

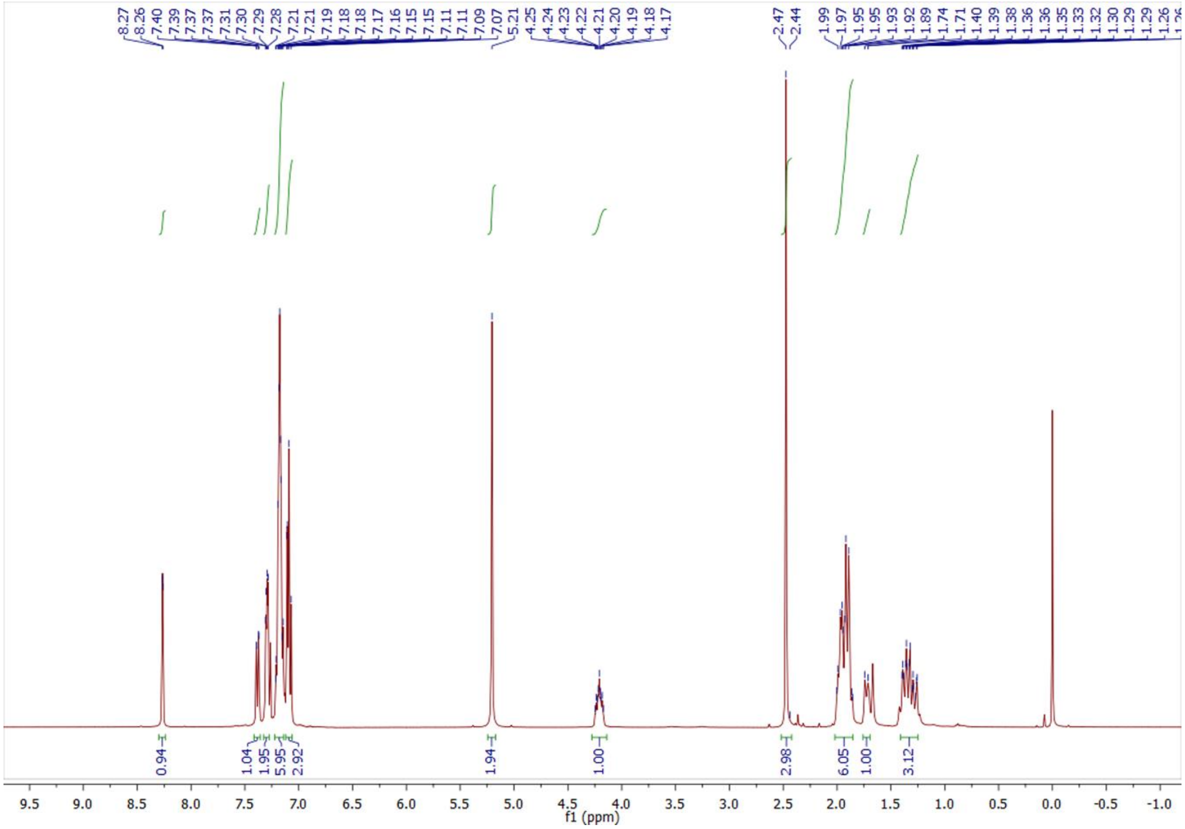

Figure S120. ^{1}H NMR (400 MHz, $CDCl_3$) spectrum of compound **4j**.

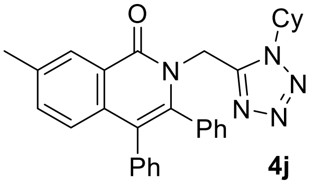

2-((1-cyclohexyl-1*H*-tetrazol-5-yl)methyl)-7-methyl-3,4-diphenylisoquinolin-1(2*H*)-one

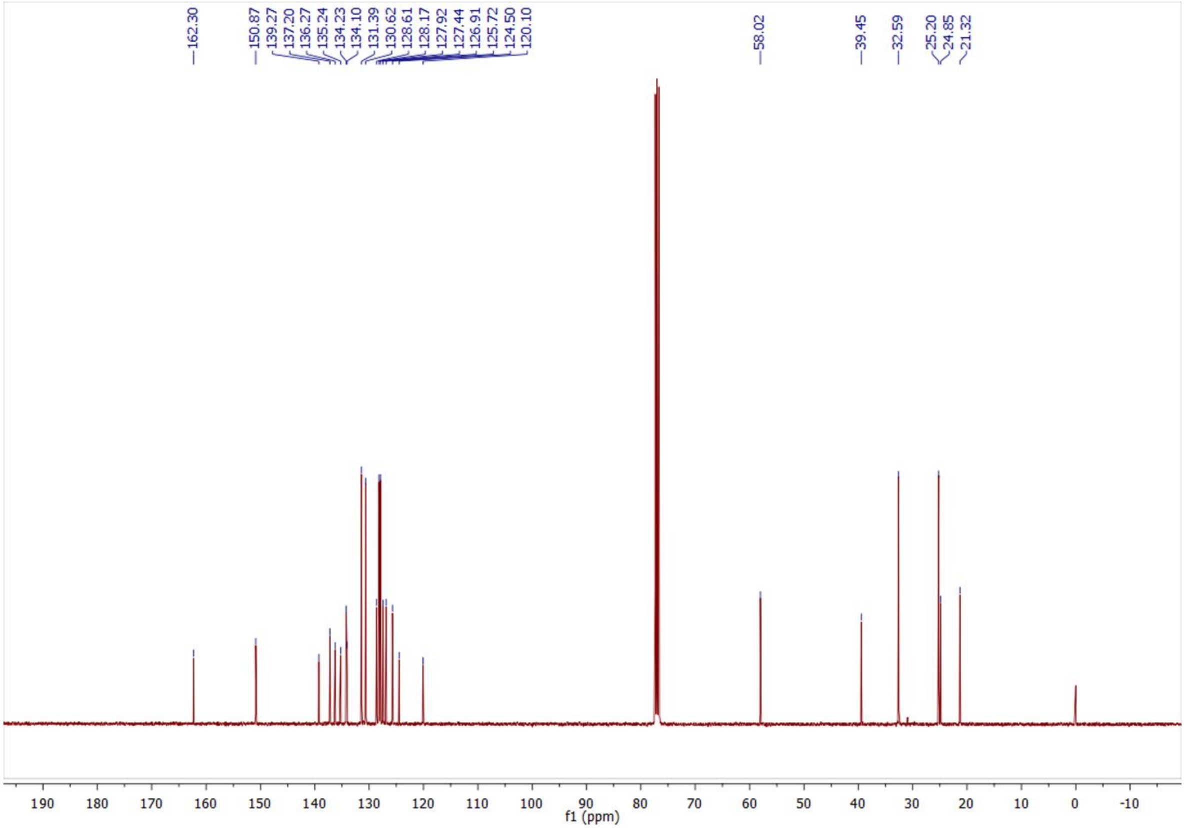

Figure S121. ^{13}C {^{1}H} NMR (101 MHz, $CDCl_3$) spectrum of compound **4j**.

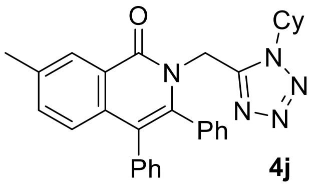

4j

2-((1-cyclohexyl-1*H*-tetrazol-5-yl)methyl)-7-methyl-3,4-diphenylisoquinolin-1(2*H*)-one

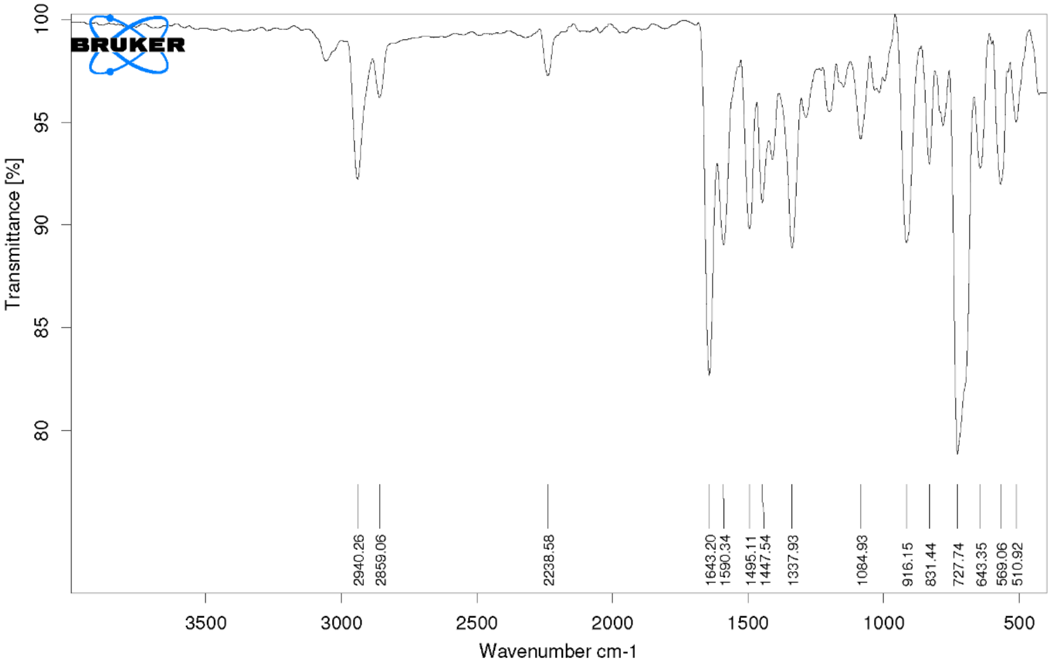

C:\Users\hvs\Documents\IR spectra\Erik Van Der Eycken\Felix\Gerardo\GM-088.0	GM-088	Instrument type and / or accessory	8/24/2018

Page 1/1

Figure S122. FT-IR (KBr) spectrum of compound **4j**.

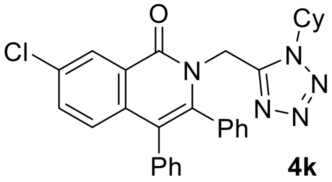

4k

7-chloro-2-((1-cyclohexyl-1*H*-tetrazol-5-yl)methyl)-3,4-diphenylisoquinolin-1(2*H*)-one

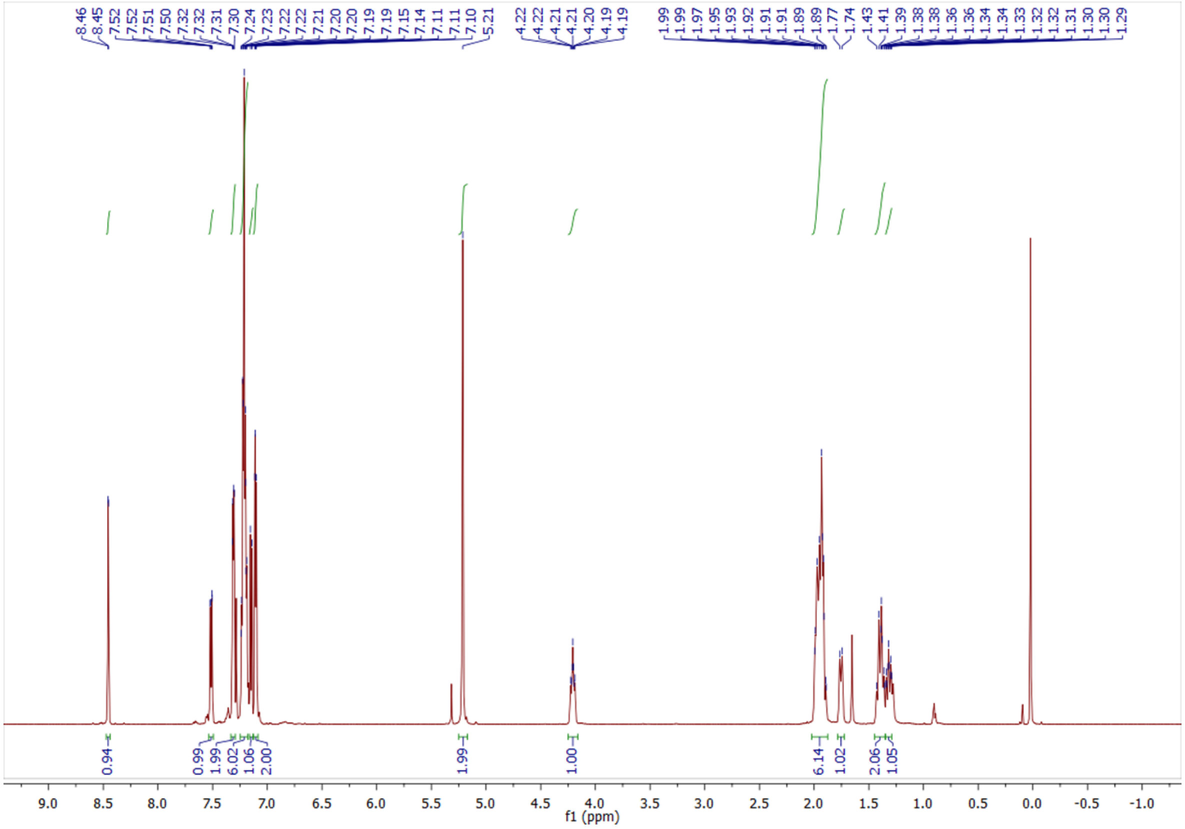

Figure S123. ^{1}H NMR (600 MHz, $CDCl_3$) spectrum of compound **4k**.

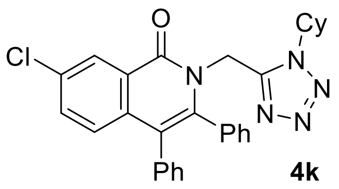

4k

7-chloro-2-((1-cyclohexyl-1*H*-tetrazol-5-yl)methyl)-
3,4-diphenylisoquinolin-1(2*H*)-one

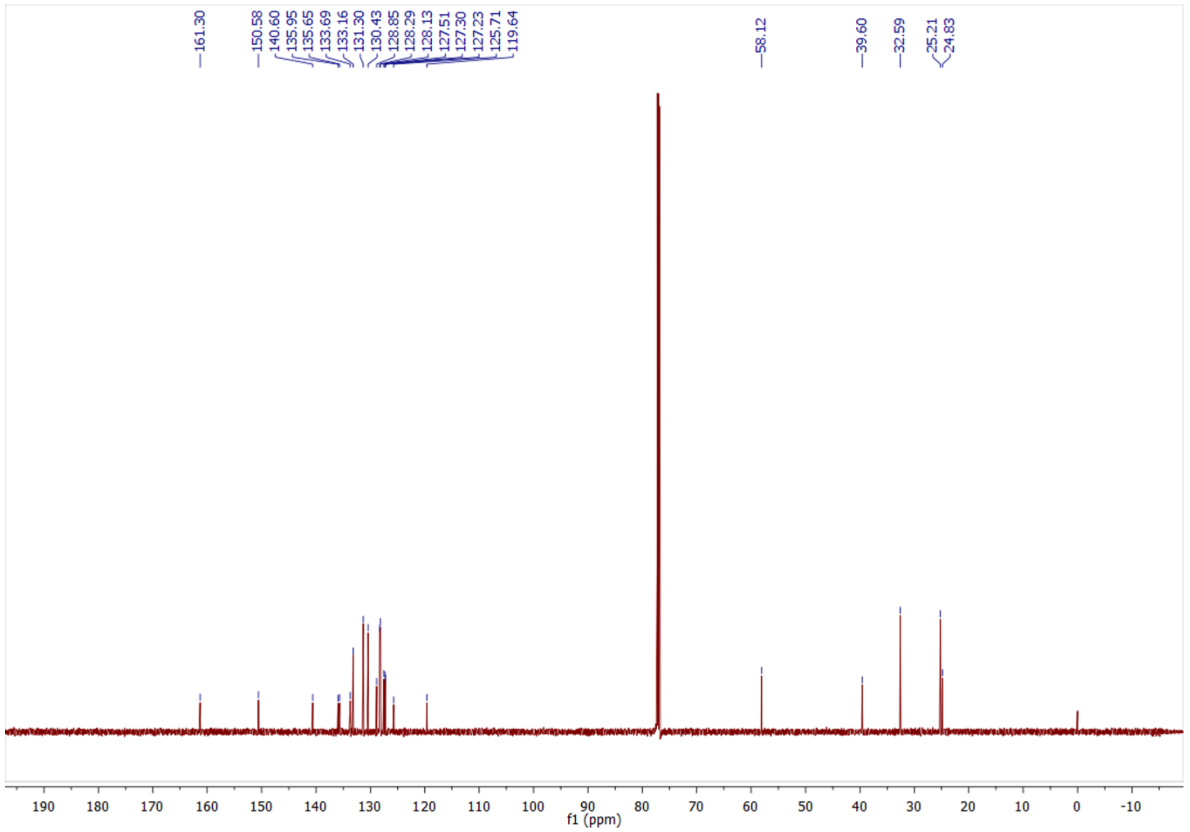

Figure S124. ^{13}C {^{1}H} NMR (151 MHz, $CDCl_3$) spectrum of compound **4k**.

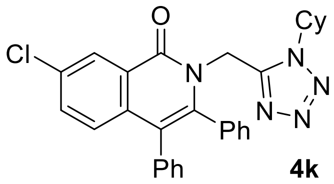

7-chloro-2-((1-cyclohexyl-1*H*-tetrazol-5-yl)methyl)-
3,4-diphenylisoquinolin-1(2*H*)-one

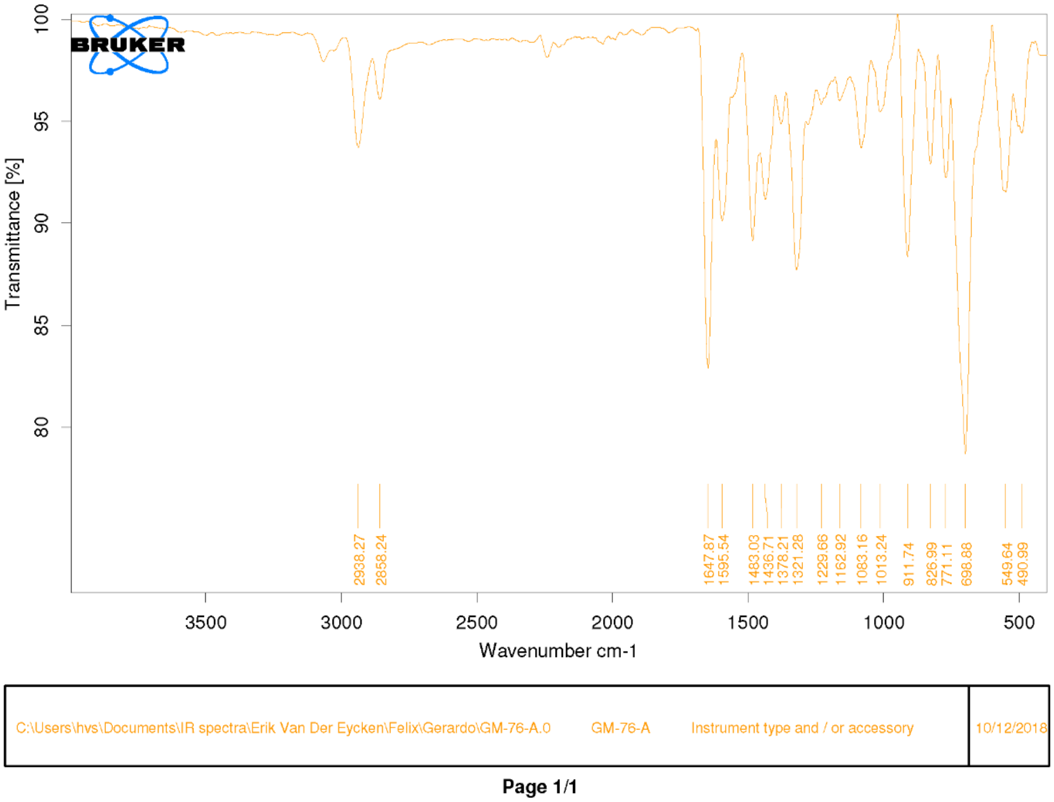

Figure S125. FT-IR (KBr) spectrum of compound **4k**.

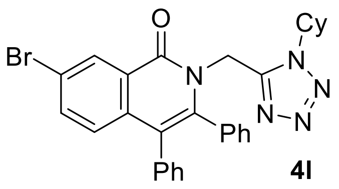

4l

7-bromo-2-((1-cyclohexyl-1*H*-tetrazol-5-yl)methyl)-
3,4-diphenylisoquinolin-1(2*H*)-one

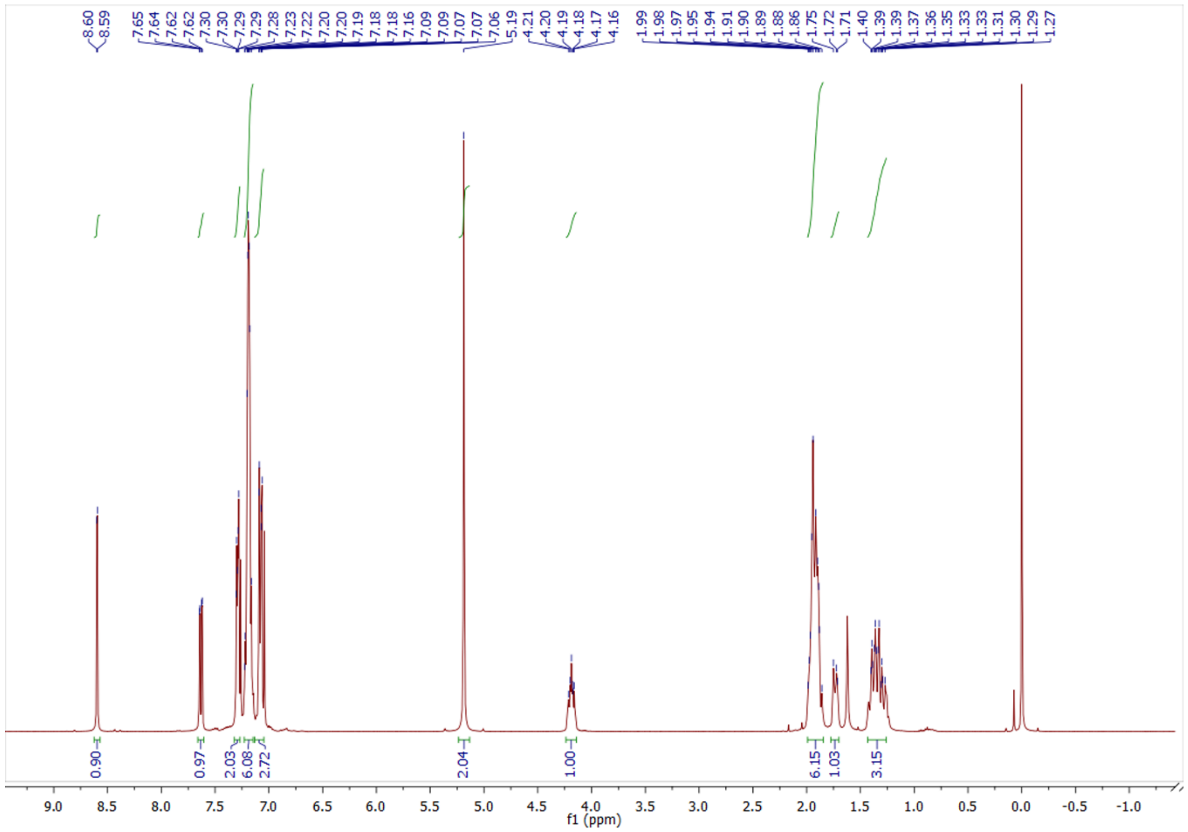

Figure S126. ^{1}H NMR (400 MHz, $CDCl_3$) spectrum of compound **4l**.

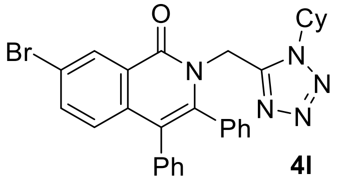

4l

7-bromo-2-((1-cyclohexyl-1*H*-tetrazol-5-yl)methyl)-
3,4-diphenylisoquinolin-1(2*H*)-one

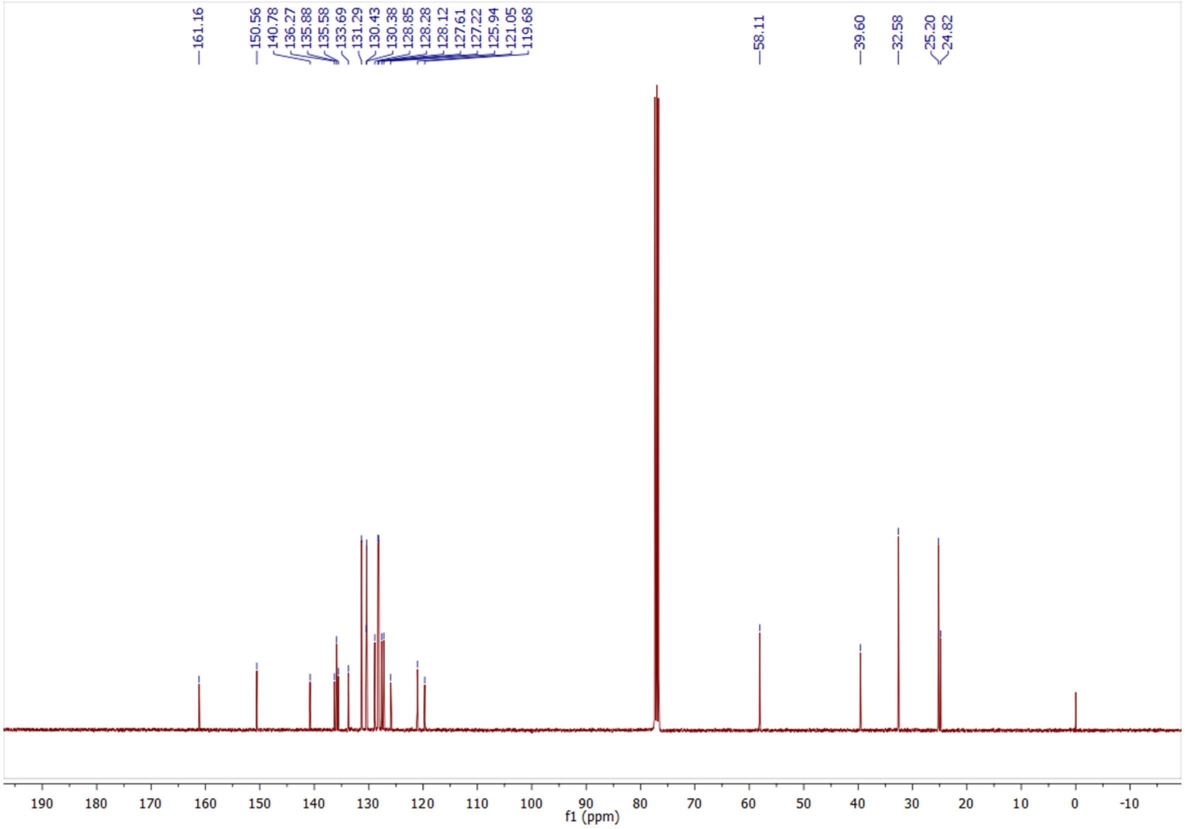

Figure S127. $^{13}C\ \{^{1}H\}$ NMR (101 MHz, $CDCl_3$) spectrum of compound **4l**.

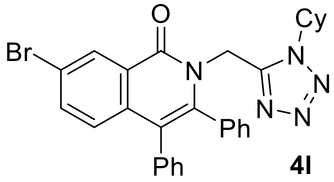

4l

7-bromo-2-((1-cyclohexyl-1*H*-tetrazol-5-yl)methyl)-
3,4-diphenylisoquinolin-1(2*H*)-one

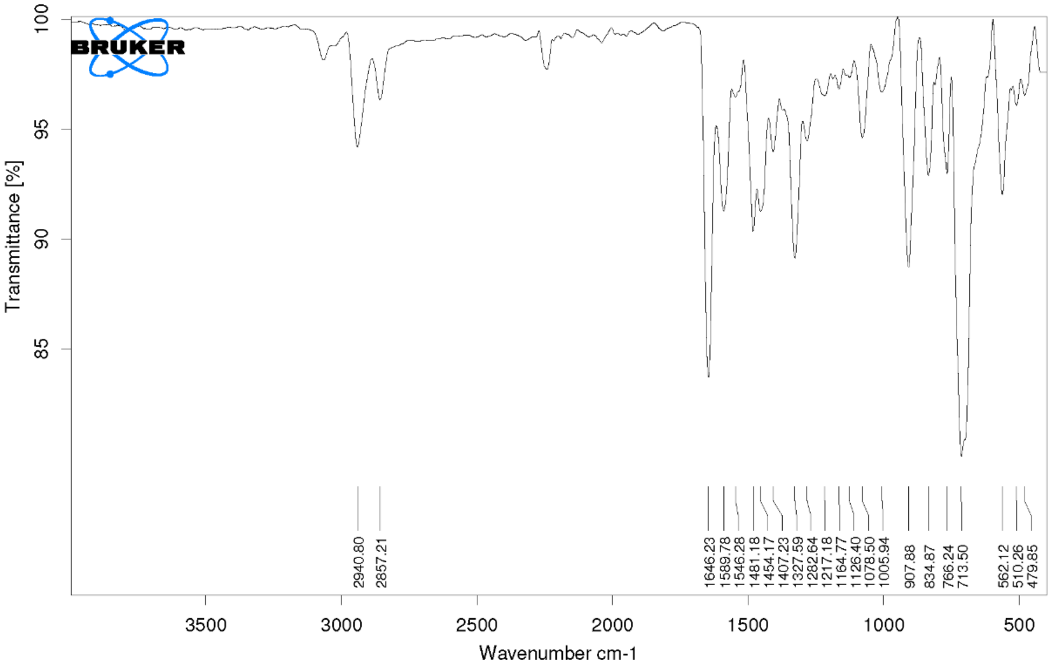

C:\Users\hvs\Documents\IR spectra\Erik Van Der Eycken\Felix\Gerardo\GM-078-A.0	GM-078-A	Instrument type and / or accessory	10/12/2018

Page 1/1

Figure S128. FT-IR (KBr) spectrum of compound **4l**.

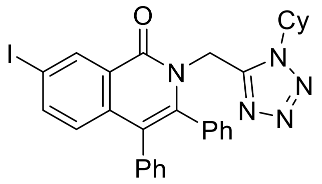

4m

2-((1-cyclohexyl-1*H*-tetrazol-5-yl)methyl)-7-iodo-3,4-diphenylisoquinolin-1(2*H*)-one

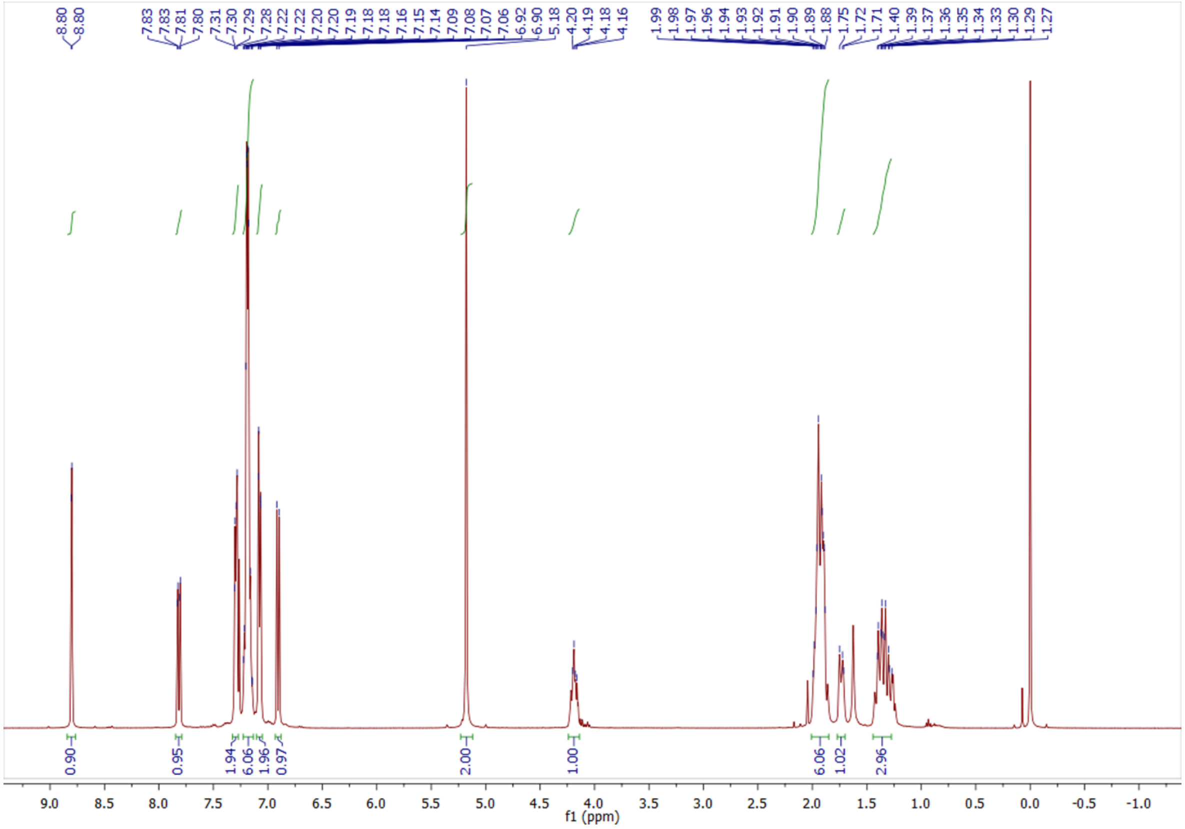

Figure S129. ^{1}H NMR (400 MHz, $CDCl_3$) spectrum of compound **4m**.

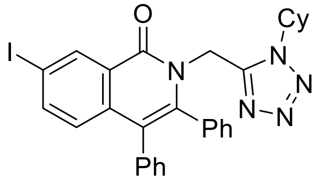

4m

2-((1-cyclohexyl-1*H*-tetrazol-5-yl)methyl)-7-iodo-3,4-diphenylisoquinolin-1(2*H*)-one

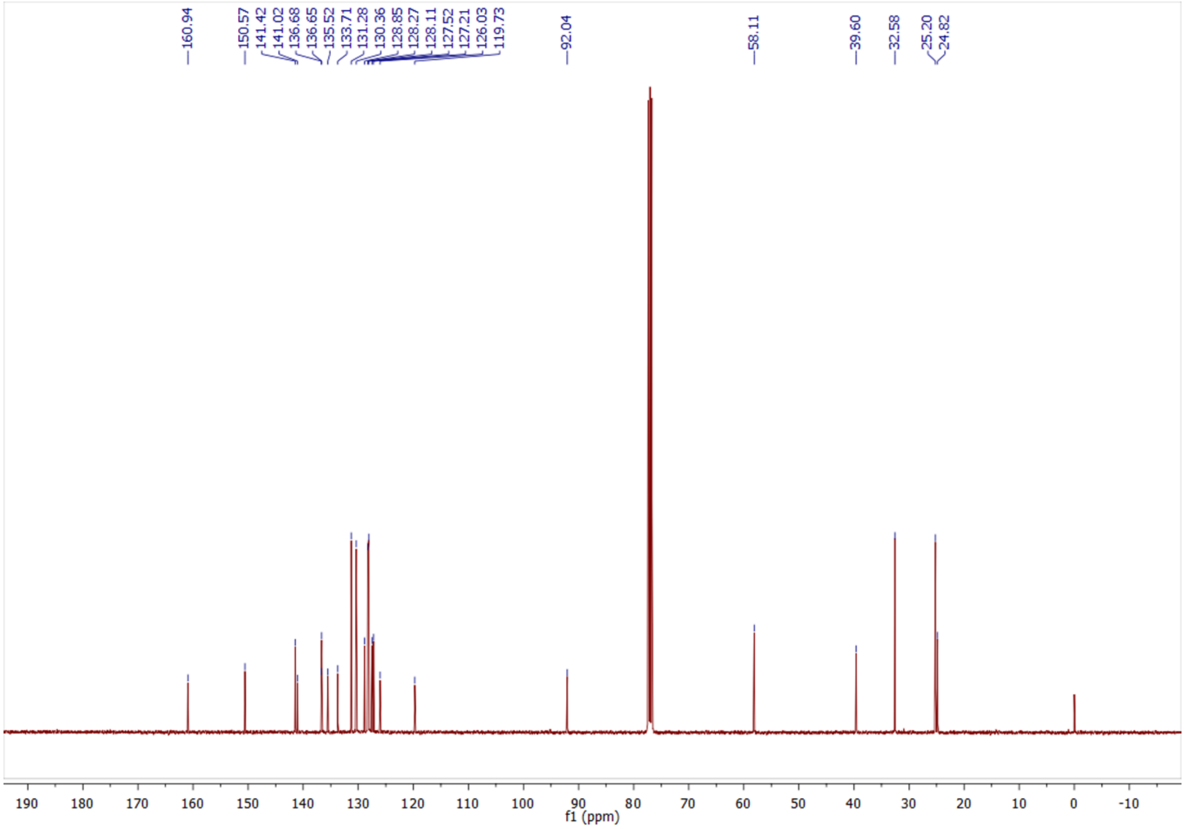

Figure S130. $^{13}C\ \{^{1}H\}$ NMR (101 MHz, $CDCl_3$) spectrum of compound **4m**.

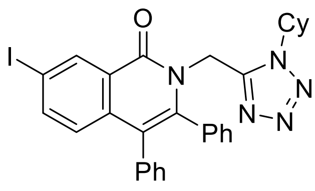

4m

2-((1-cyclohexyl-1*H*-tetrazol-5-yl)methyl)-7-iodo-3,4-diphenylisoquinolin-1(2*H*)-one

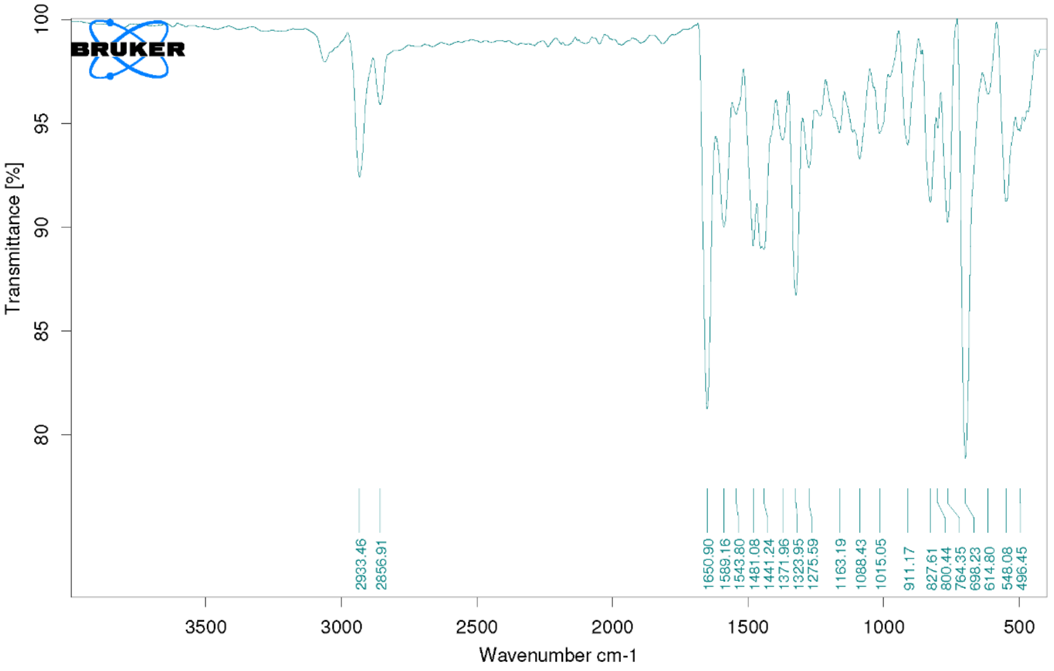

C:\Users\hvs\Documents\IR spectra\Erik Van Der Eycken\Felix\Gerardo\GM-080-A.0 GM-080-A Instrument type and / or accessory	10/12/2018

Page 1/1

Figure S131. FT-IR (KBr) spectrum of compound **4m**.

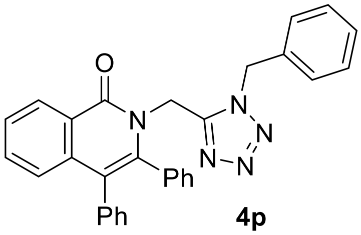

4p

2-((1-benzyl-1*H*-tetrazol-5-yl)methyl)-3,4-diphenylisoquinolin-1(2*H*)-one

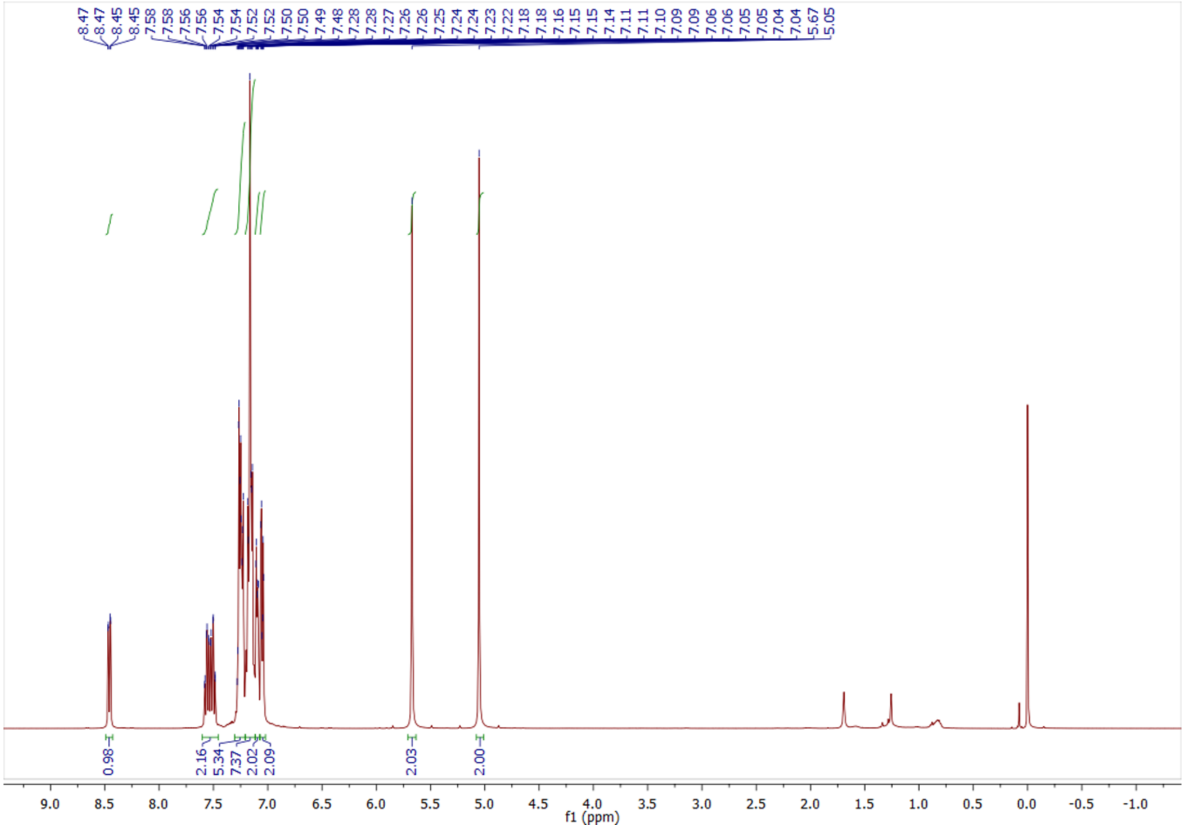

Figure S132. ^{1}H NMR (400 MHz, $CDCl_3$) spectrum of compound **4p**.

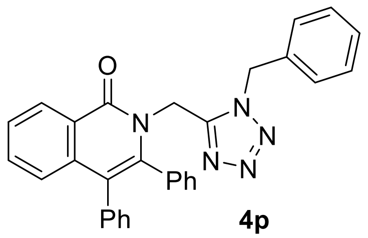

4p

2-((1-benzyl-1*H*-tetrazol-5-yl)methyl)-3,4-diphenylisoquinolin-1(2*H*)-one

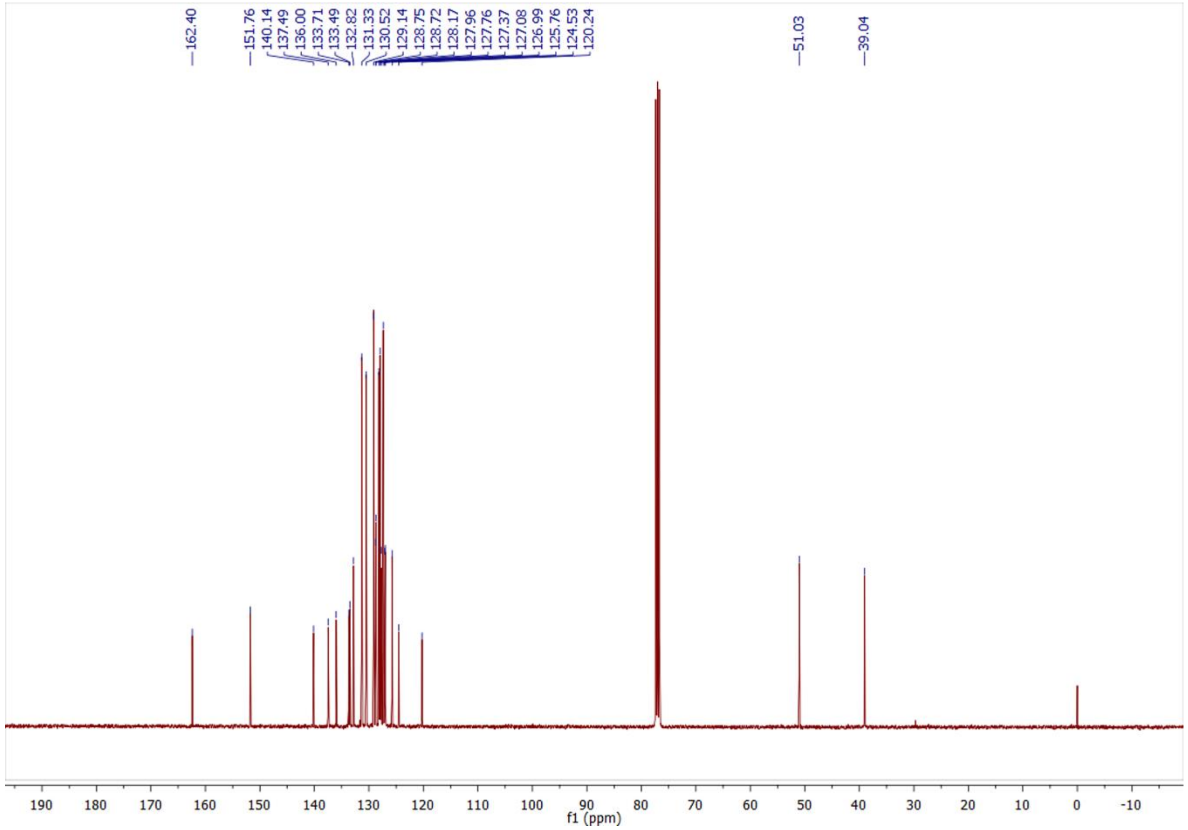

Figure S133. $^{13}C\ \{^1H\}$ NMR (101 MHz, $CDCl_3$) spectrum of compound **4p**.

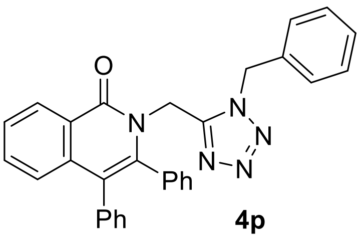

4p

2-((1-benzyl-1*H*-tetrazol-5-yl)methyl)-3,4-diphenylisoquinolin-1(2*H*)-one

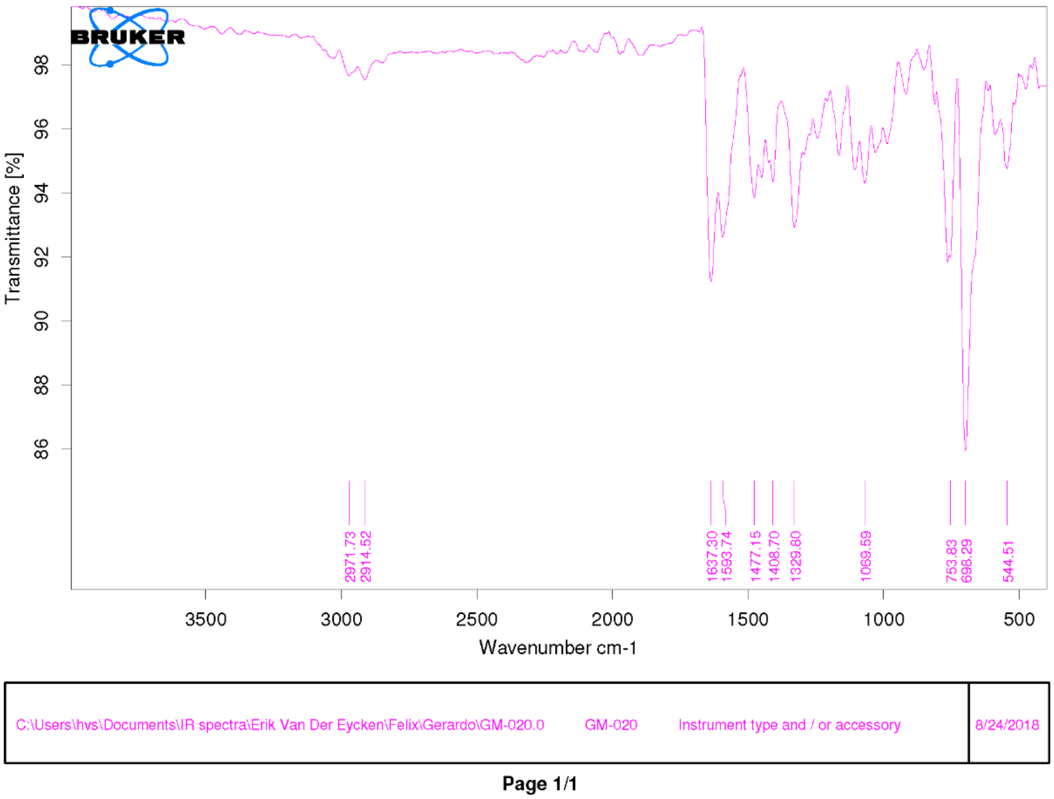

Figure S134. FT-IR (KBr) spectrum of compound **4p**.

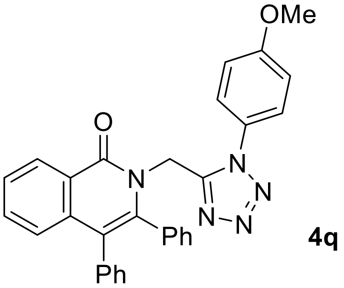

2-((1-(4-methoxyphenyl)-1*H*-tetrazol-5-yl)methyl)-3,4-diphenylisoquinolin-1(2*H*)-one

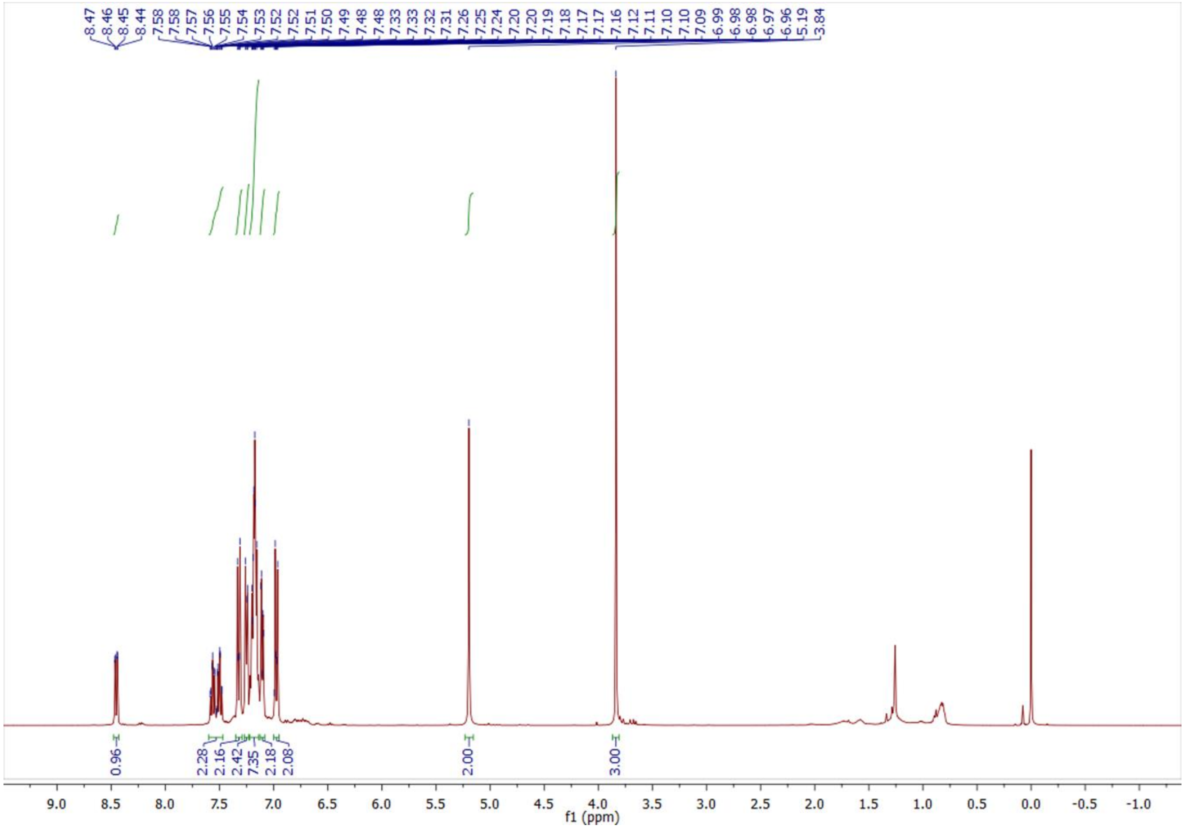

Figure S135. ^{1}H NMR (400 MHz, $CDCl_3$) spectrum of compound **4q**.

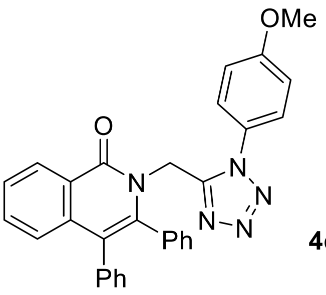

4q

2-((1-(4-methoxyphenyl)-1*H*-tetrazol-5-yl)methyl)-3,4-diphenylisoquinolin-1(2*H*)-one

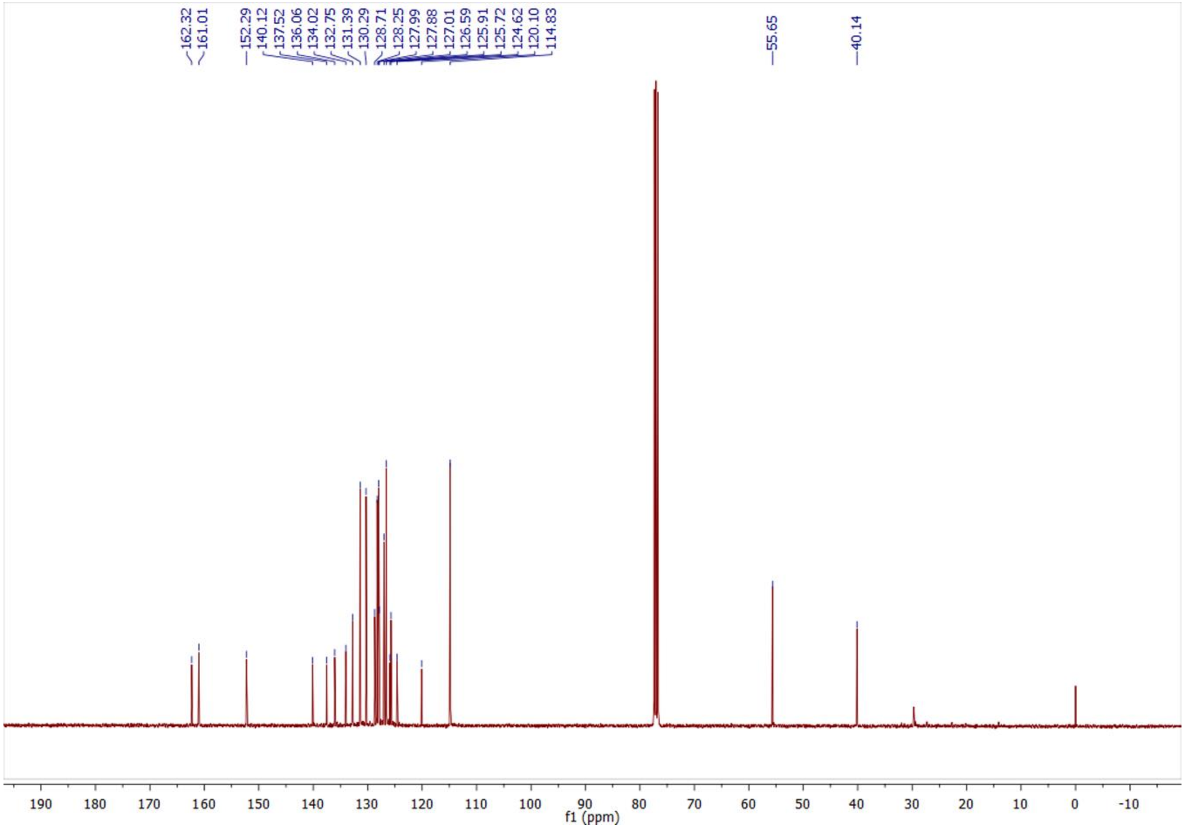

Figure S136. ^{13}C {^{1}H} NMR (101 MHz, $CDCl_3$) spectrum of compound **4q**.

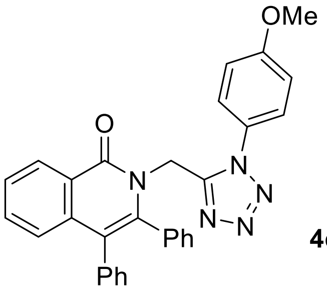

4q

2-((1-(4-methoxyphenyl)-1*H*-tetrazol-5-yl)methyl)-3,4-diphenylisoquinolin-1(2*H*)-one

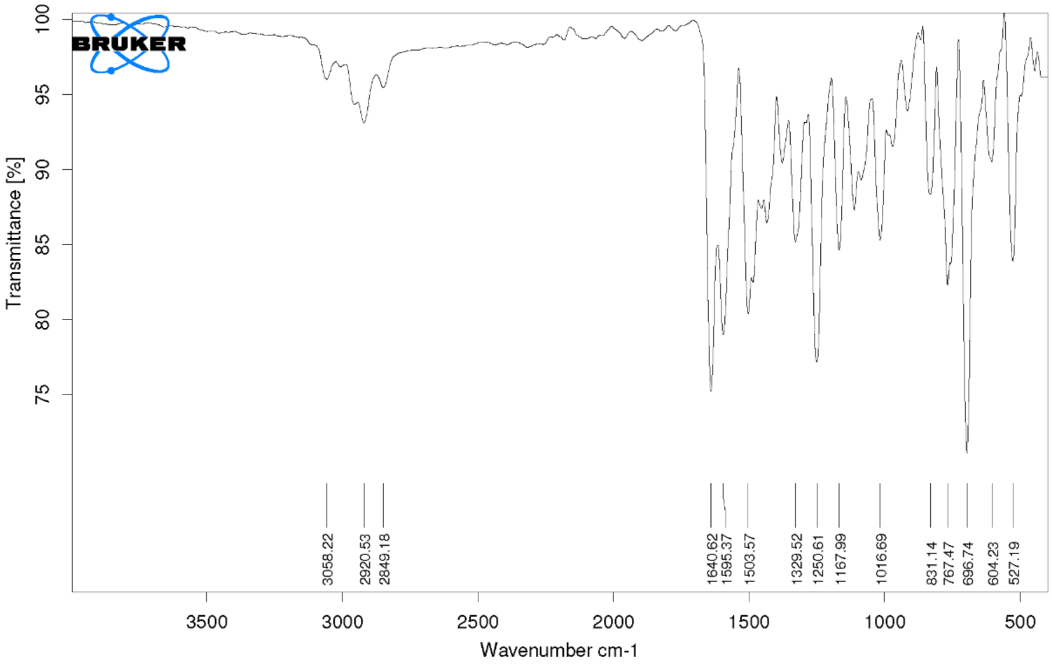

C:\Users\hvs\Documents\IR spectra\Erik Van Der Eycken\Felix\Gerardo\GM-022.0	GM-022	Instrument type and / or accessory	8/24/2018

Page 1/1

Figure S137. FT-IR (KBr) spectrum of compound **4q**.

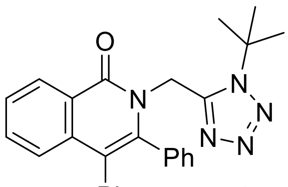

4r

2-((1-(*tert*-butyl)-1*H*-tetrazol-5-yl)methyl)-3,4-diphenylisoquinolin-1(2*H*)-one

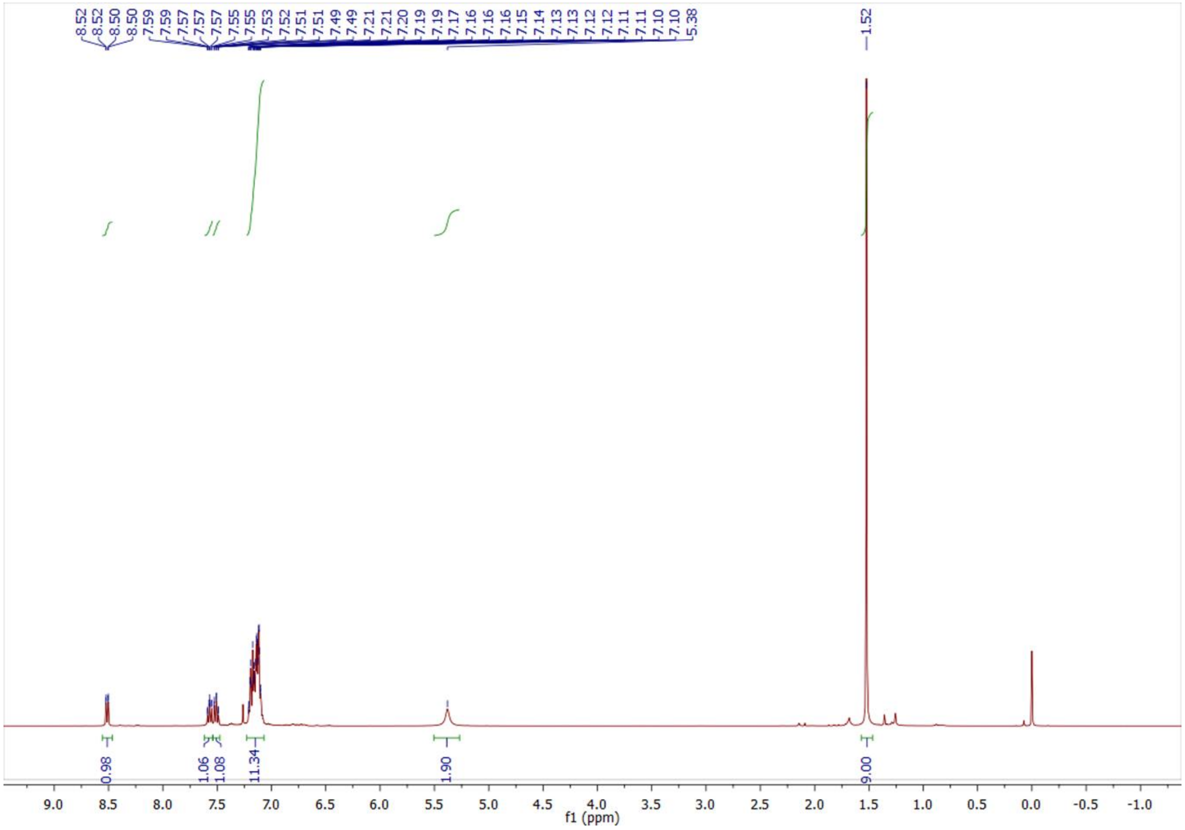

Figure S138. ^1H NMR (400 MHz, $CDCl_3$) spectrum of compound **4r**.

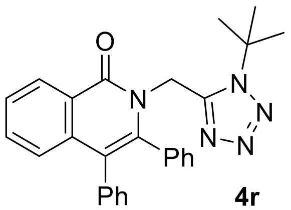

4r

2-((1-(*tert*-butyl)-1*H*-tetrazol-5-yl)methyl)-3,4-diphenylisoquinolin-1(2*H*)-one

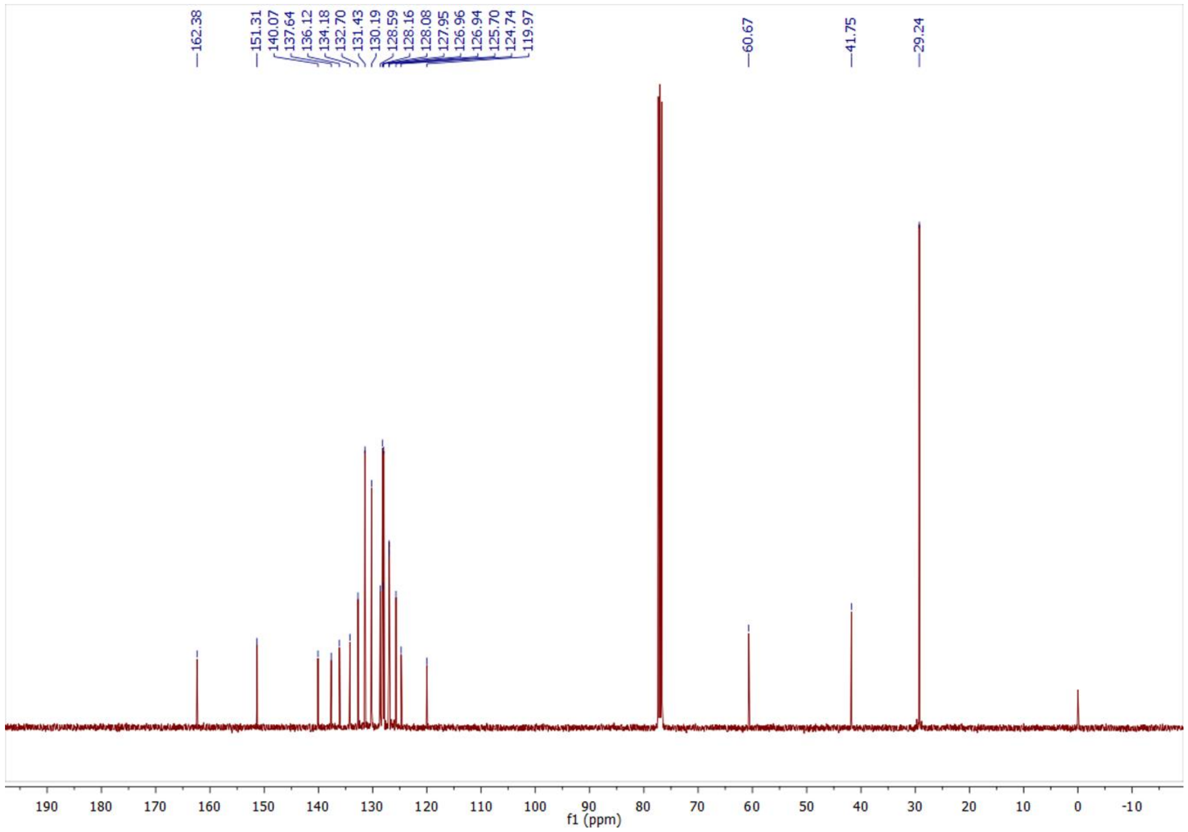

Figure S139. ^{13}C {^{1}H} NMR (101 MHz, $CDCl_3$) spectrum of compound **4r**.

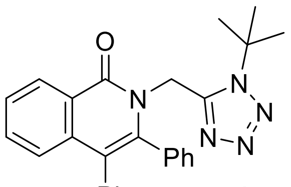

4r

2-((1-(*tert*-butyl)-1*H*-tetrazol-5-yl)methyl)-3,4-diphenylisoquinolin-1(2*H*)-one

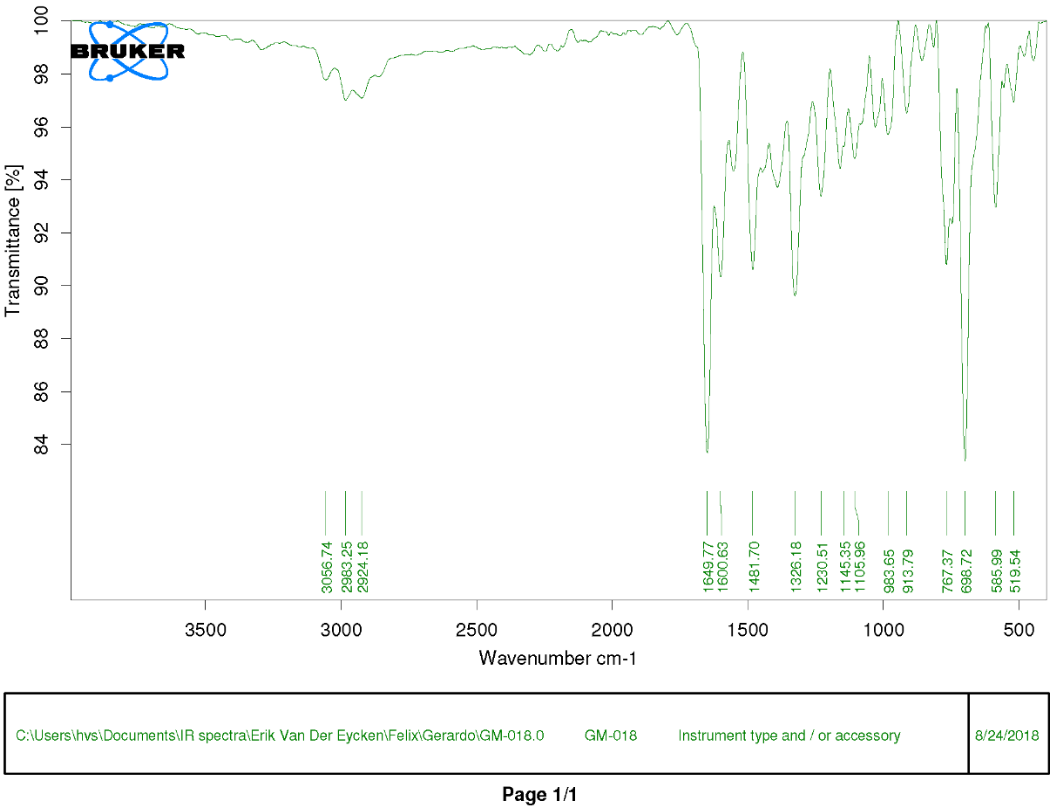

Figure S140. FT-IR (KBr) spectrum of compound **4r**.

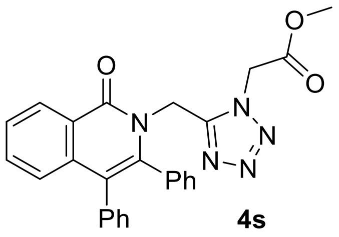

methyl 2-(5-((1-oxo-3,4-diphenylisoquinolin-2(1*H*)-yl)methyl)-1*H*-tetrazol-1-yl)acetate

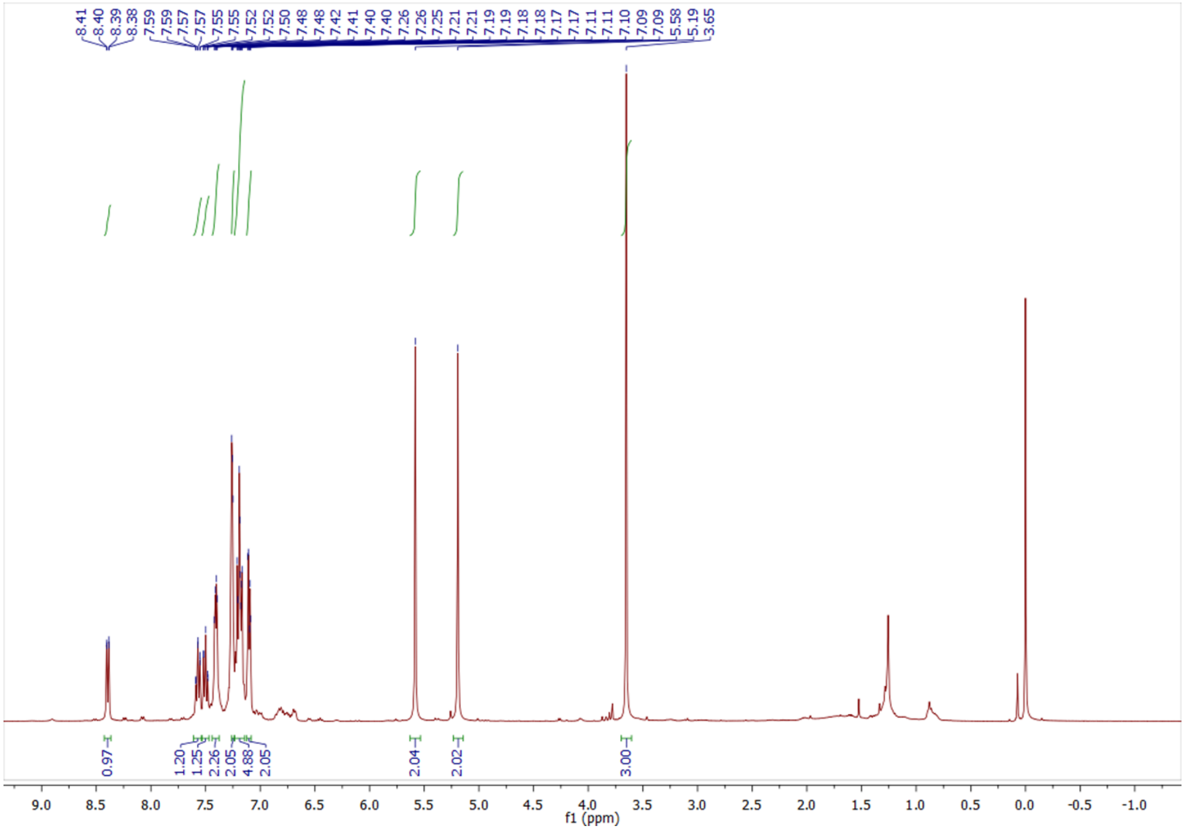

Figure S141 ^{1}H NMR (400 MHz, CDCl$_3$) spectrum of compound **4s**.

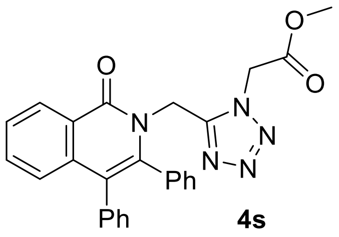

4s

methyl 2-(5-((1-oxo-3,4-diphenylisoquinolin-2(1*H*)-yl)methyl)-1*H*-tetrazol-1-yl)acetate

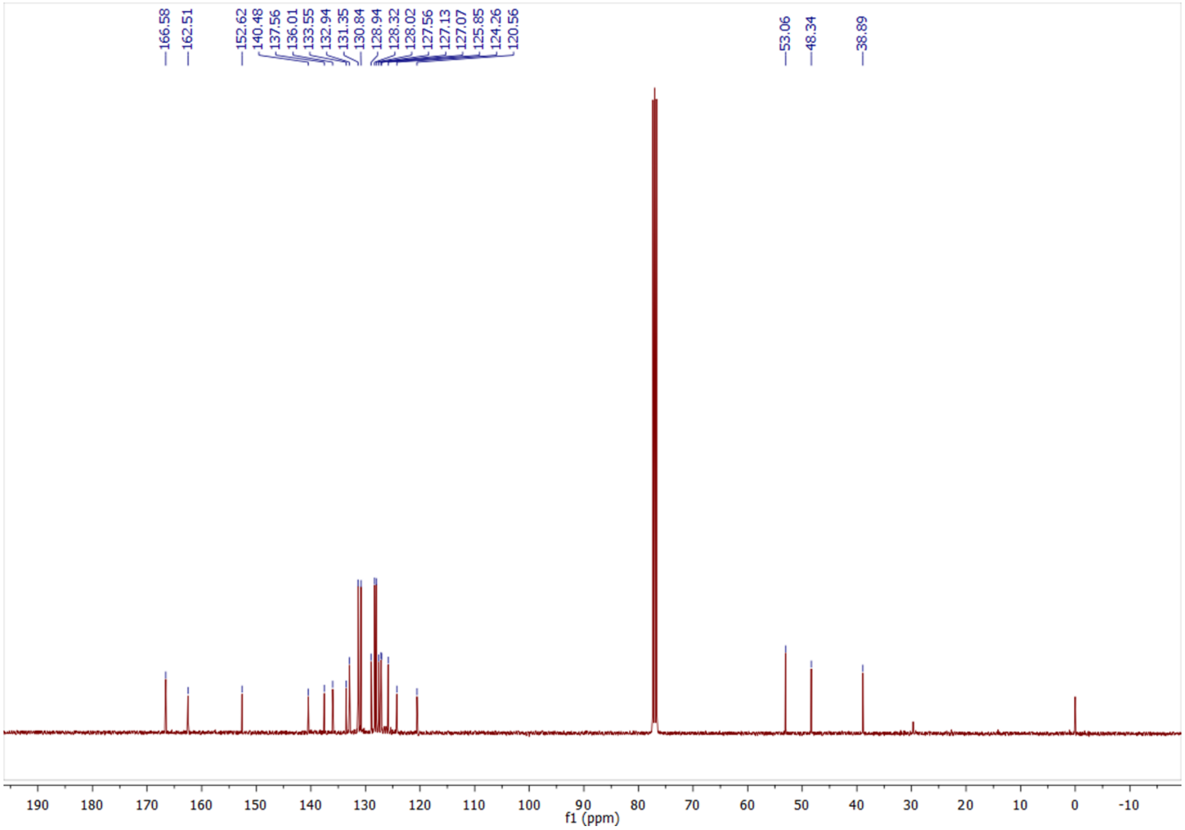

Figure S144. ^{13}C {^{1}H} NMR (101 MHz, $CDCl_3$) spectrum of compound **4s**.

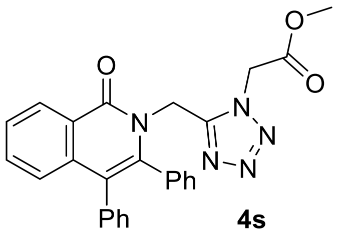

4s

methyl 2-(5-((1-oxo-3,4-diphenylisoquinolin-2(1*H*)-yl)methyl)-1*H*-tetrazol-1-yl)acetate

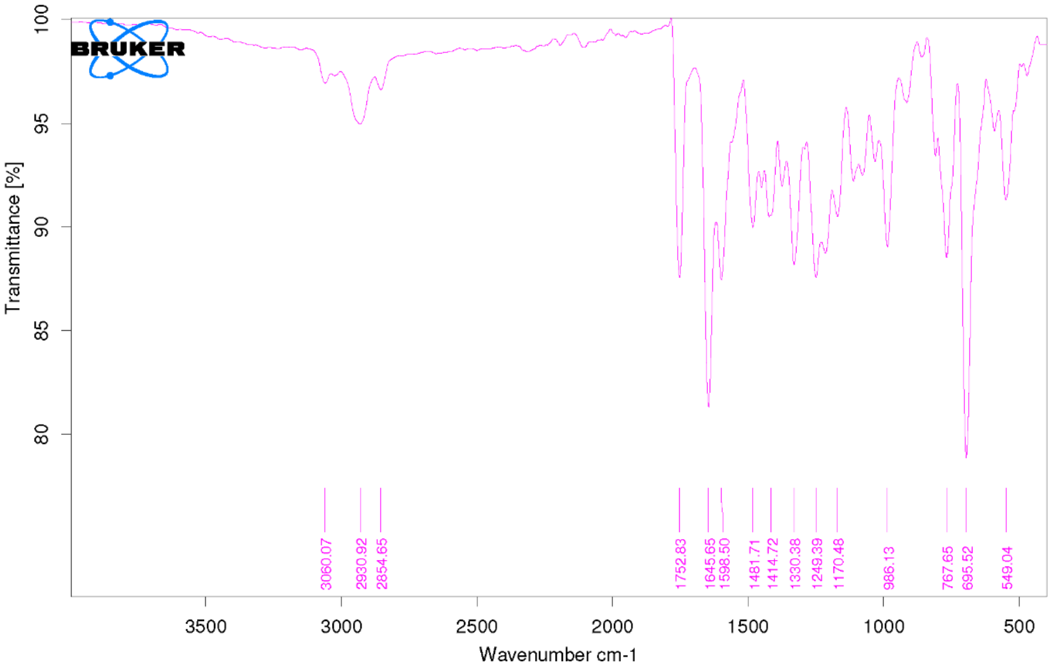

C:\Users\hvs\Documents\IR spectra\Erik Van Der Eycken\Felix\Gerardo\GM-024.0	GM-024	Instrument type and / or accessory	8/24/2018

Page 1/1

Figure S143. FT-IR (KBr) spectrum of compound **4s**.

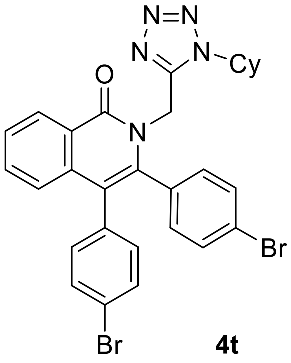

4t

3,4-bis(4-bromophenyl)-2-((1-cyclohexyl-1*H*-tetrazol-5-yl)methyl)isoquinolin-1(2*H*)-one

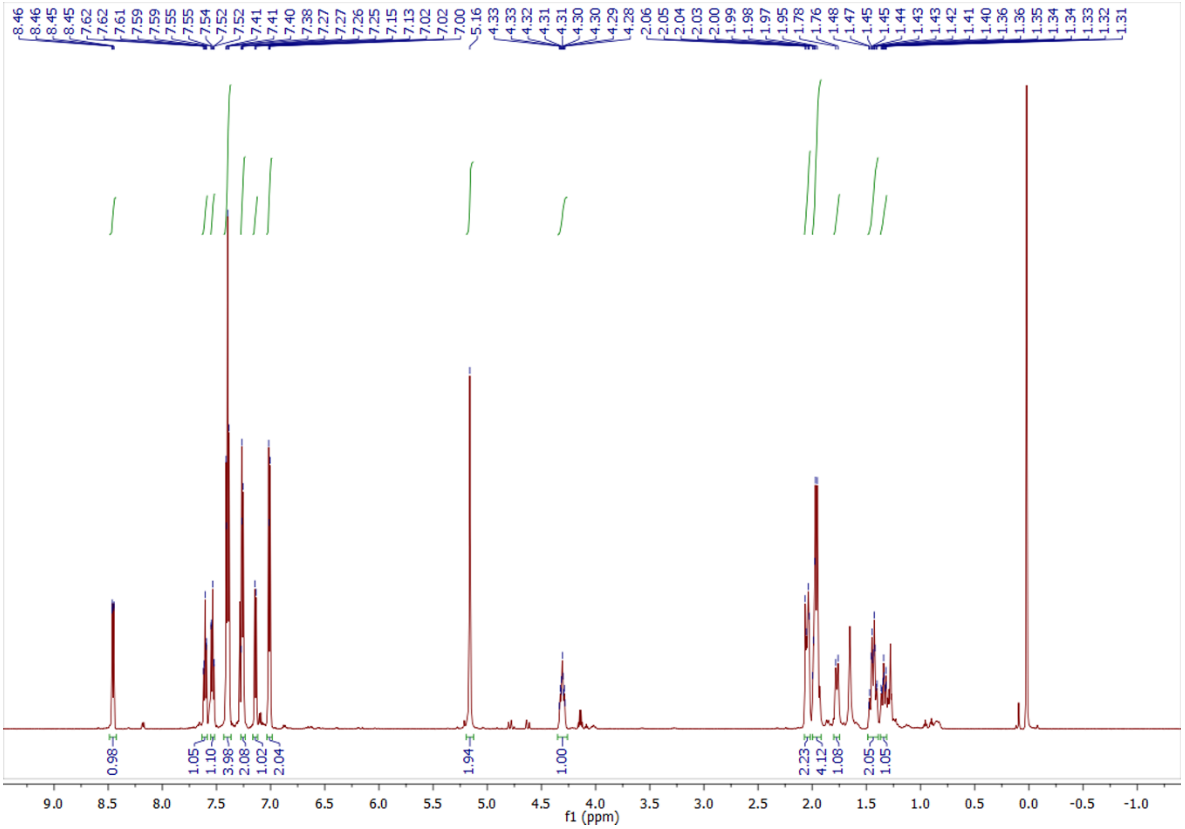

Figure S144. ^{1}H NMR (600 MHz, $CDCl_3$) spectrum of compound **4t**.

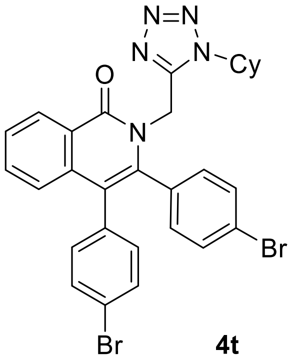

4t

3,4-bis(4-bromophenyl)-2-((1-cyclohexyl-1*H*-tetrazol-5-yl)methyl)isoquinolin-1(2*H*)-one

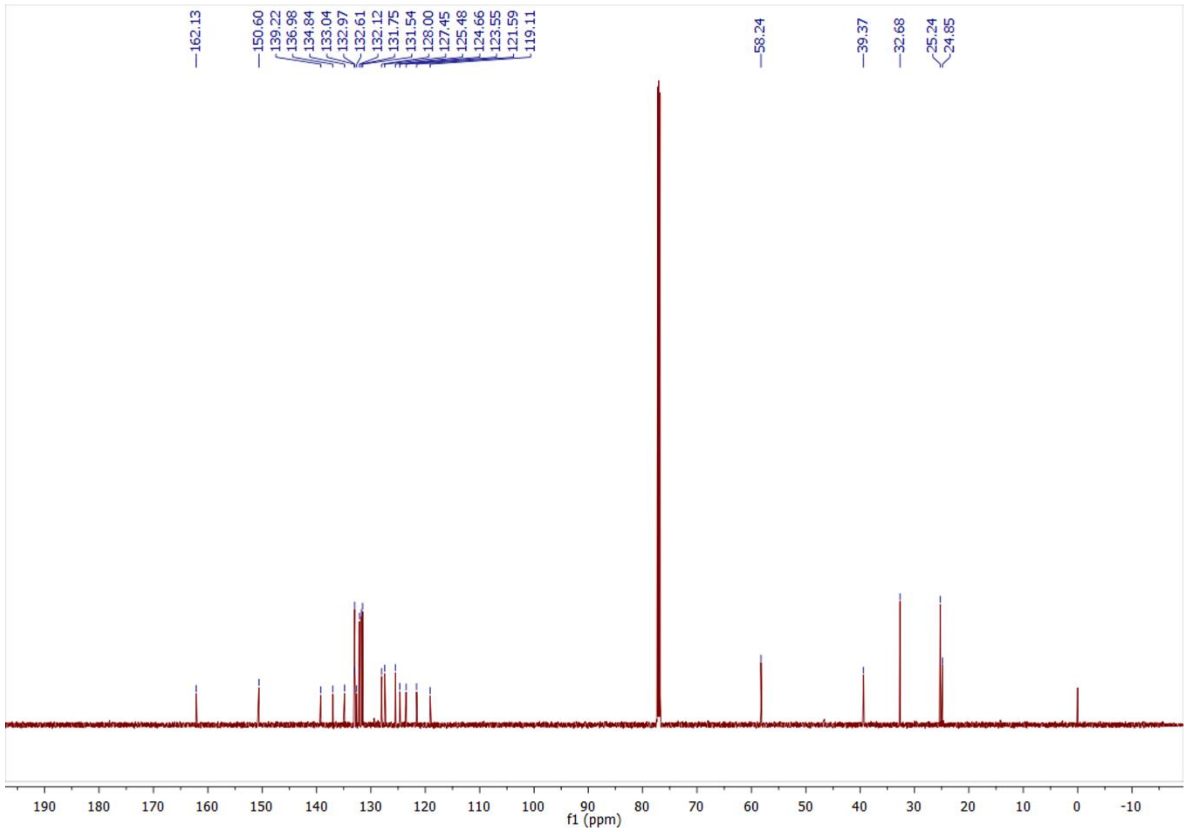

Figure S145. ^{13}C {^{1}H} NMR (151 MHz, $CDCl_3$) spectrum of compound **4t**.

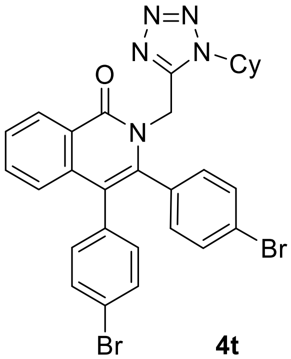

4t

3,4-bis(4-bromophenyl)-2-((1-cyclohexyl-1*H*-tetrazol-5-yl)methyl)isoquinolin-1(2*H*)-one

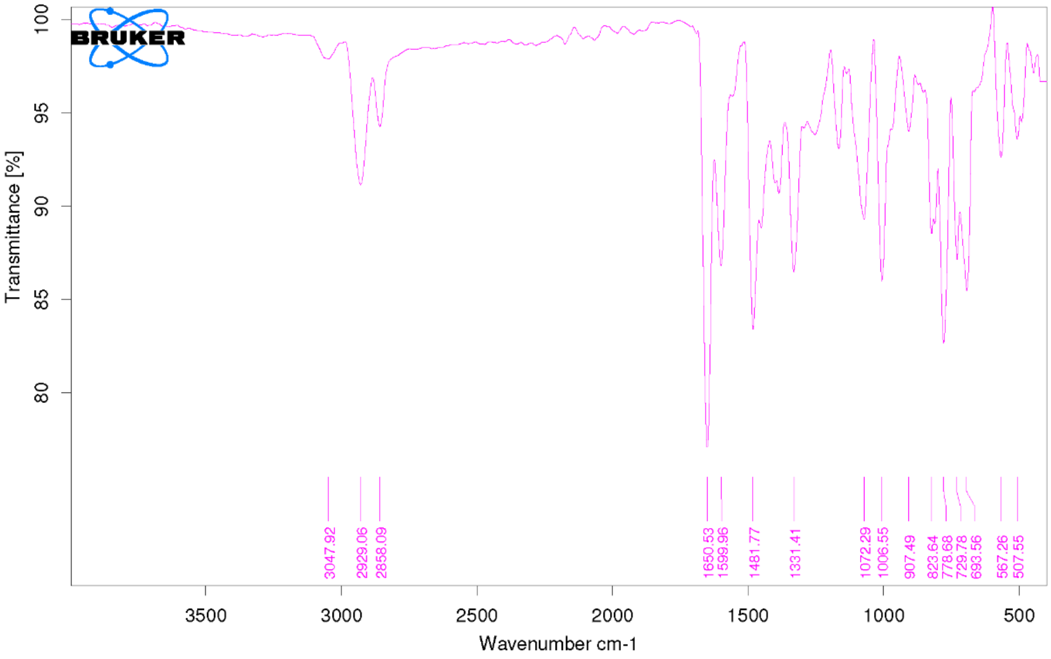

C:\Users\hvs\Documents\IR spectra\Erik Van Der Eycken\Felix\Gerardo\GM-083.0	GM-083	Instrument type and / or accessory	8/24/2018

Page 1/1

Figure S146. FT-IR (KBr) spectrum of compound **4t**.

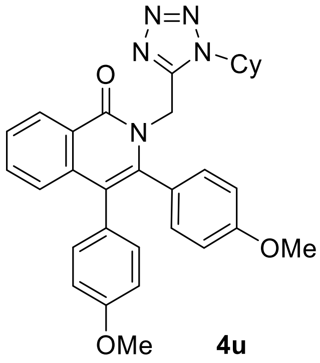

4u

2-((1-cyclohexyl-1*H*-tetrazol-5-yl)methyl)-
3,4-bis(4-methoxyphenyl)isoquinolin-1(2*H*)-one

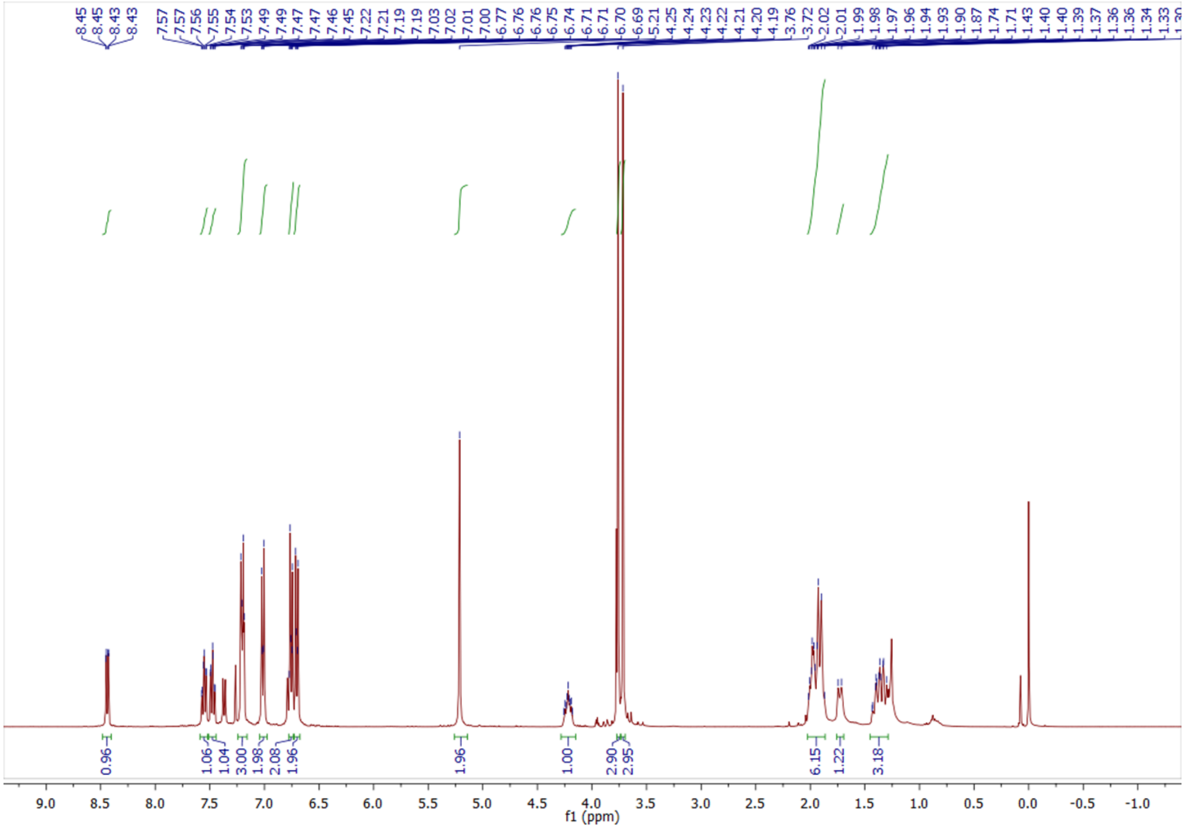

Figure S147. ^{1}H NMR (400 MHz, $CDCl_3$) spectrum of compound **4u**.

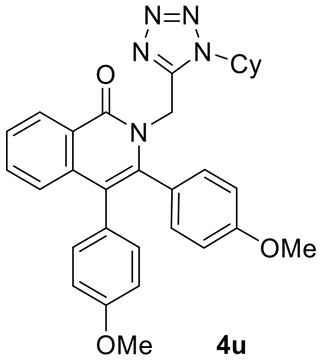

4u

2-((1-cyclohexyl-1*H*-tetrazol-5-yl)methyl)-
3,4-bis(4-methoxyphenyl)isoquinolin-1(2*H*)-one

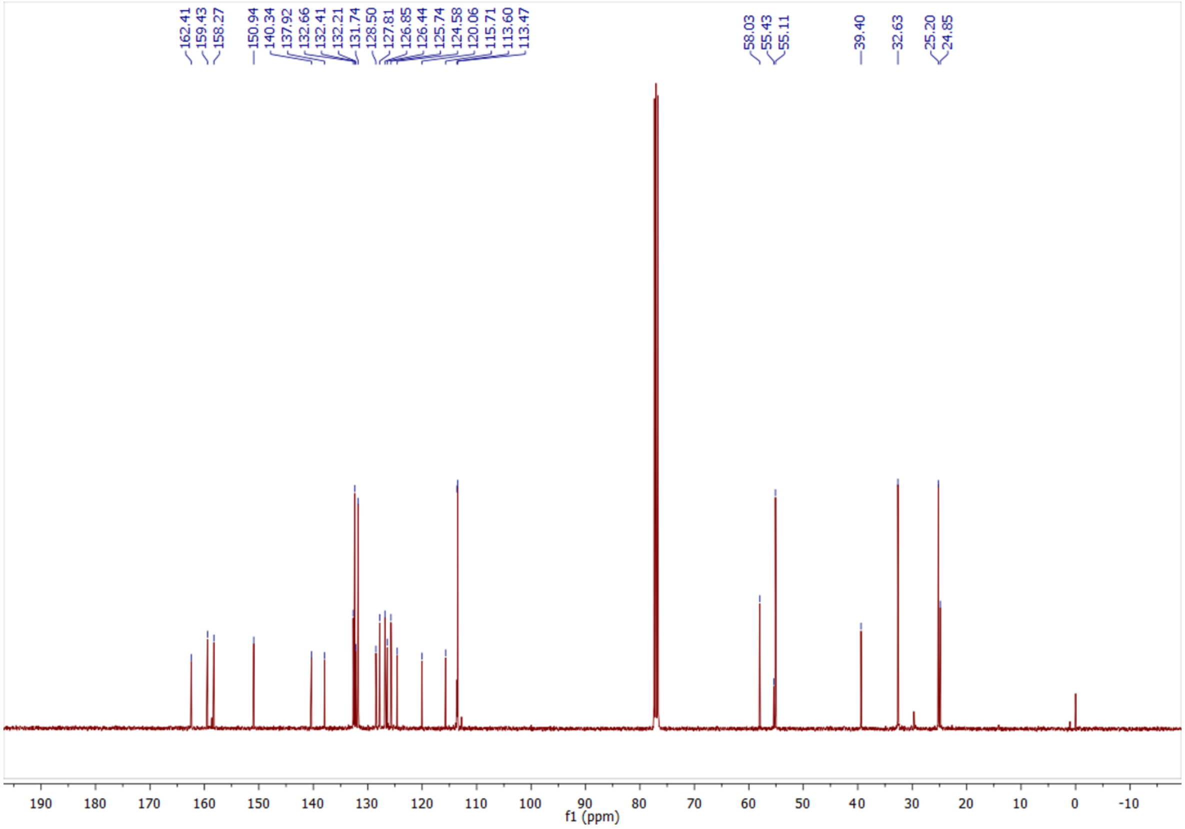

Figure S148. ^{13}C {^{1}H} NMR (101 MHz, $CDCl_3$) spectrum of compound **4u**.

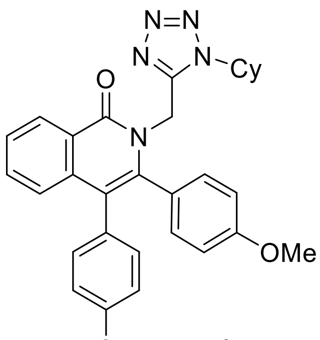

4u

2-((1-cyclohexyl-1*H*-tetrazol-5-yl)methyl)-
3,4-bis(4-methoxyphenyl)isoquinolin-1(2*H*)-one

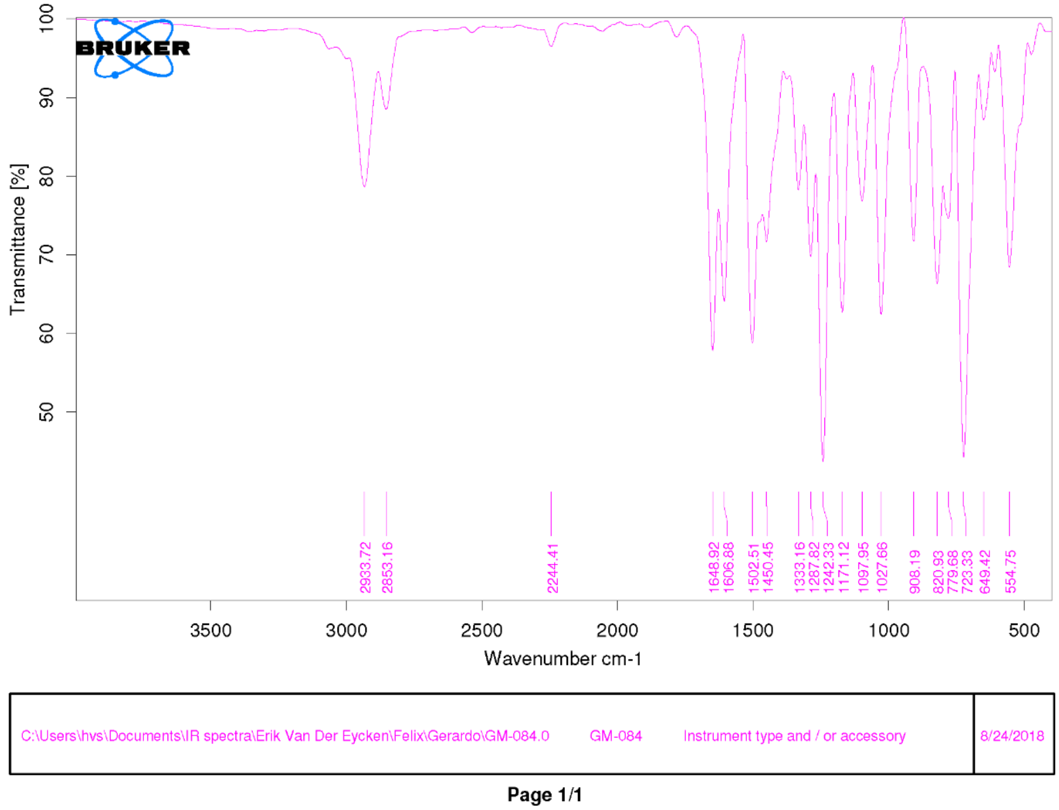

Figure S149. FT-IR (KBr) spectrum of compound **4u**.

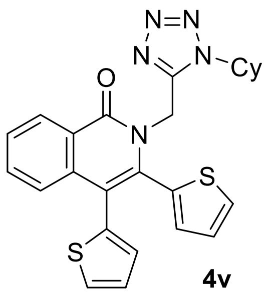

4v

2-((1-cyclohexyl-1*H*-tetrazol-5-yl)methyl)-
3,4-di(thiophen-2-yl)isoquinolin-1(2*H*)-one

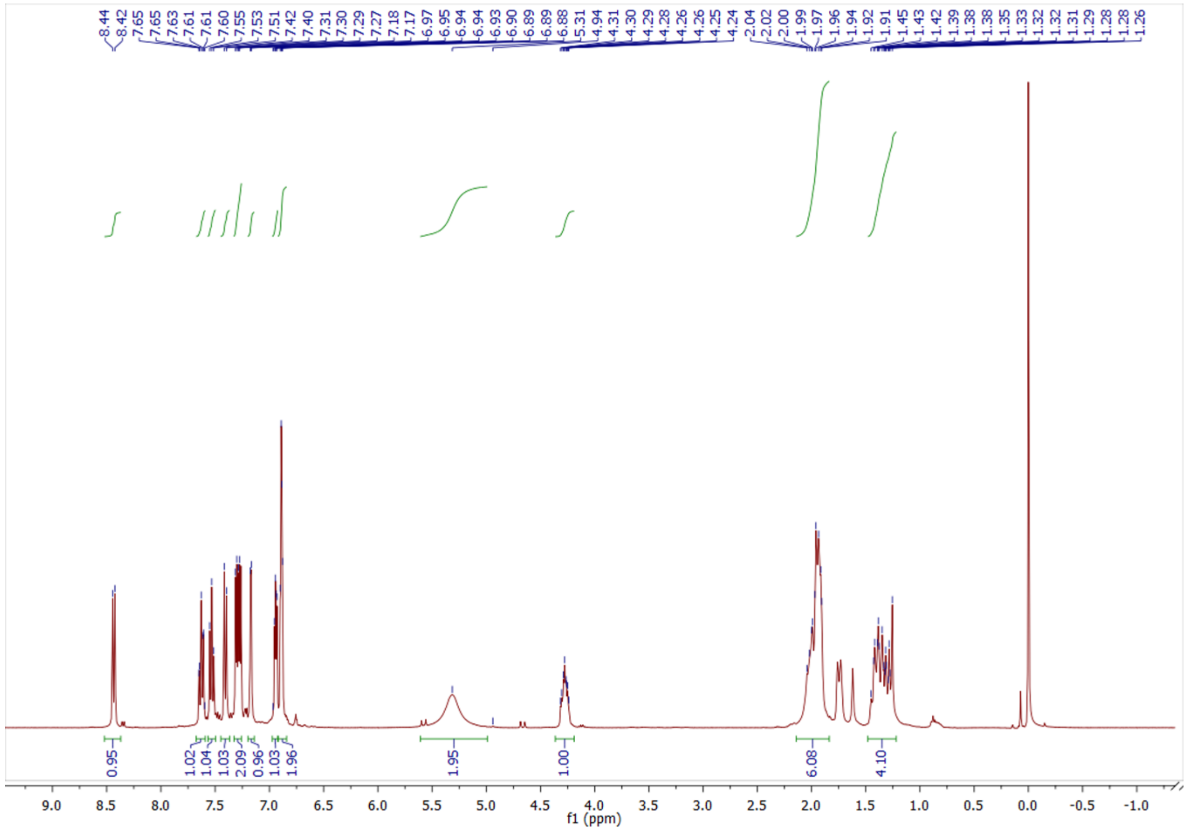

Figure S150. ^{1}H NMR (400 MHz, $CDCl_3$) spectrum of compound **4v**.

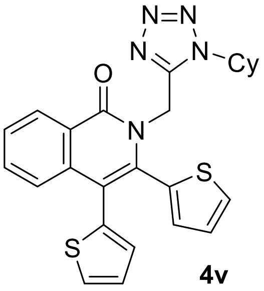

4v

2-((1-cyclohexyl-1*H*-tetrazol-5-yl)methyl)-
3,4-di(thiophen-2-yl)isoquinolin-1(2*H*)-one

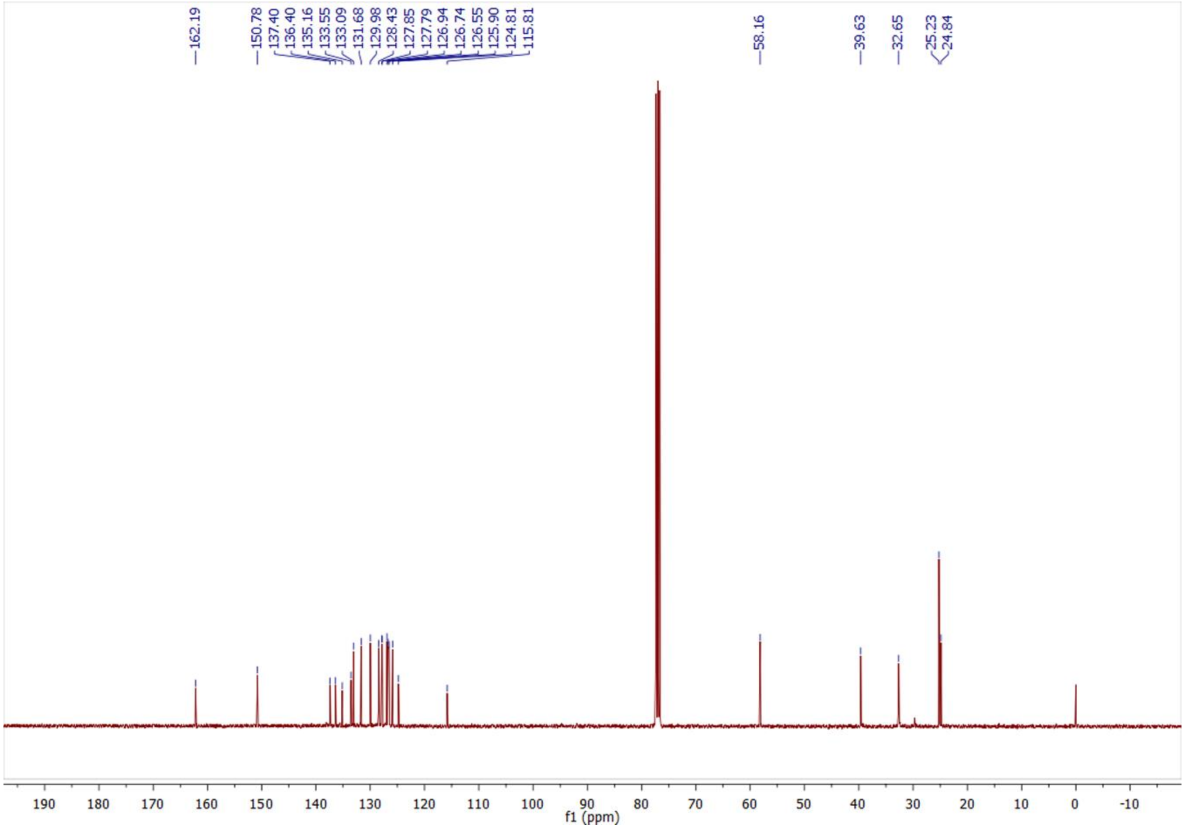

Figure S151. ^{13}C {^{1}H} NMR (101 MHz, $CDCl_3$) spectrum of compound **4v**.

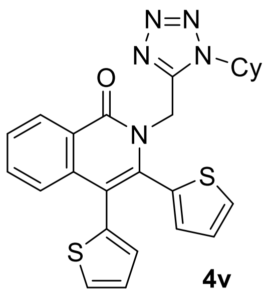

4v

2-((1-cyclohexyl-1*H*-tetrazol-5-yl)methyl)-
3,4-di(thiophen-2-yl)isoquinolin-1(2*H*)-one

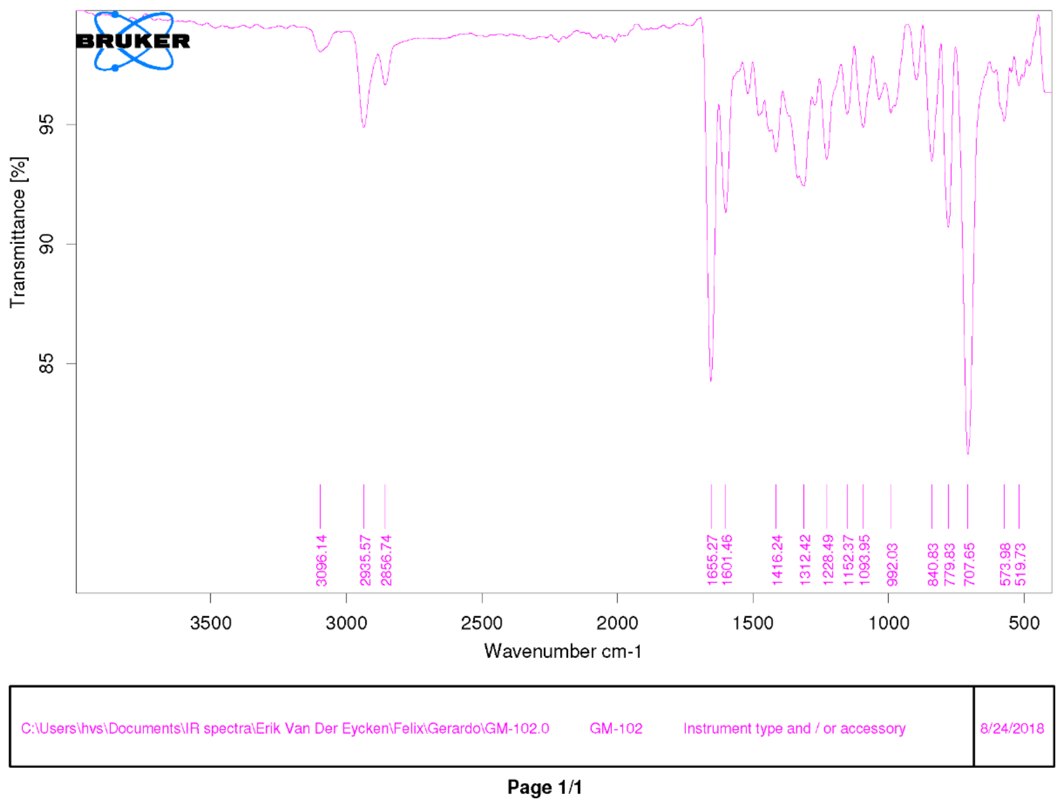

Figure S152. FT-IR (KBr) spectrum of compound **4v**.

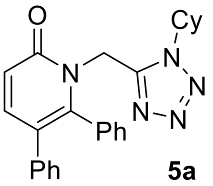

5a

1-((1-cyclohexyl-1*H*-tetrazol-5-yl)methyl)-5,6-diphenylpyridin-2(1*H*)-one

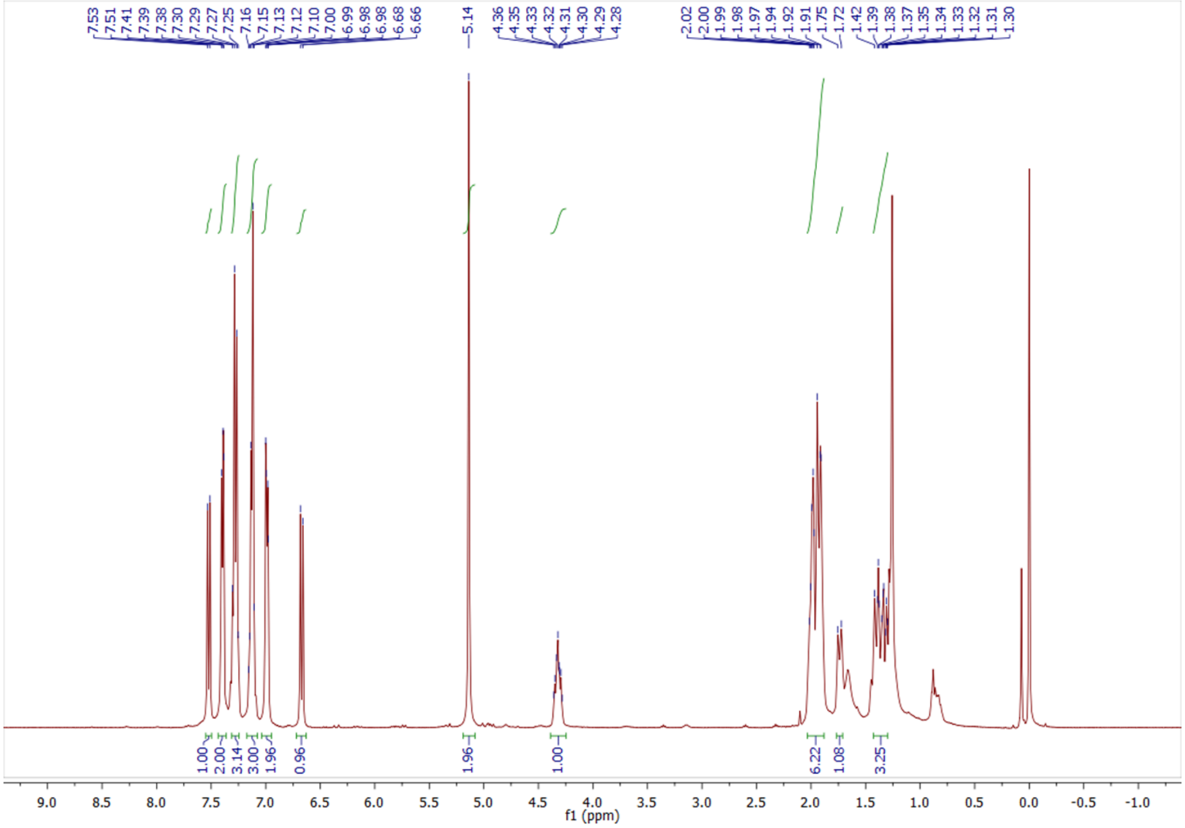

Figure S153. ^{1}H NMR (400 MHz, $CDCl_3$) spectrum of compound **5a**.

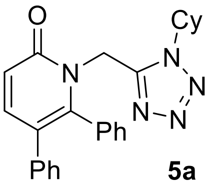

5a

1-((1-cyclohexyl-1*H*-tetrazol-5-yl)methyl)-5,6-diphenylpyridin-2(1*H*)-one

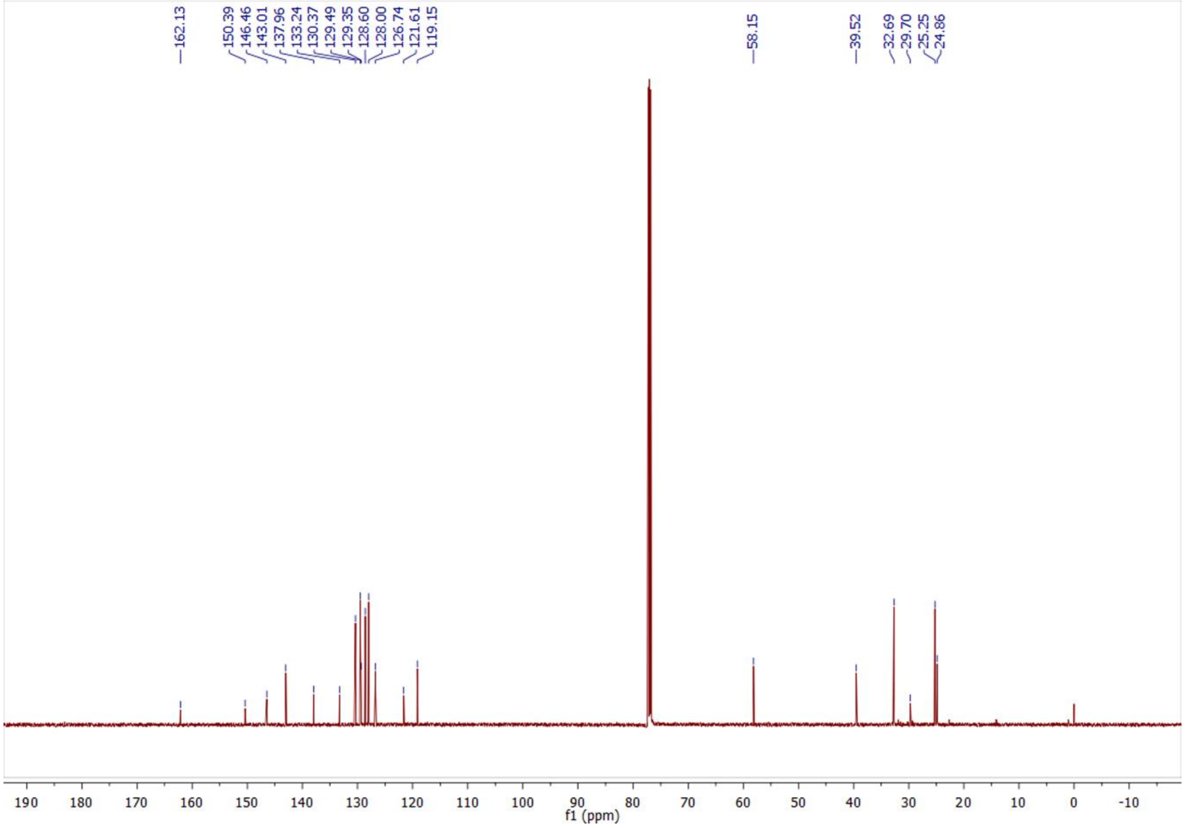

Figure S154. $^{13}C\ \{^{1}H\}$ NMR (151 MHz, $CDCl_3$) spectrum of compound **5a**.

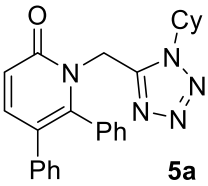

5a

1-((1-cyclohexyl-1*H*-tetrazol-5-yl)methyl)-5,6-diphenylpyridin-2(1*H*)-one

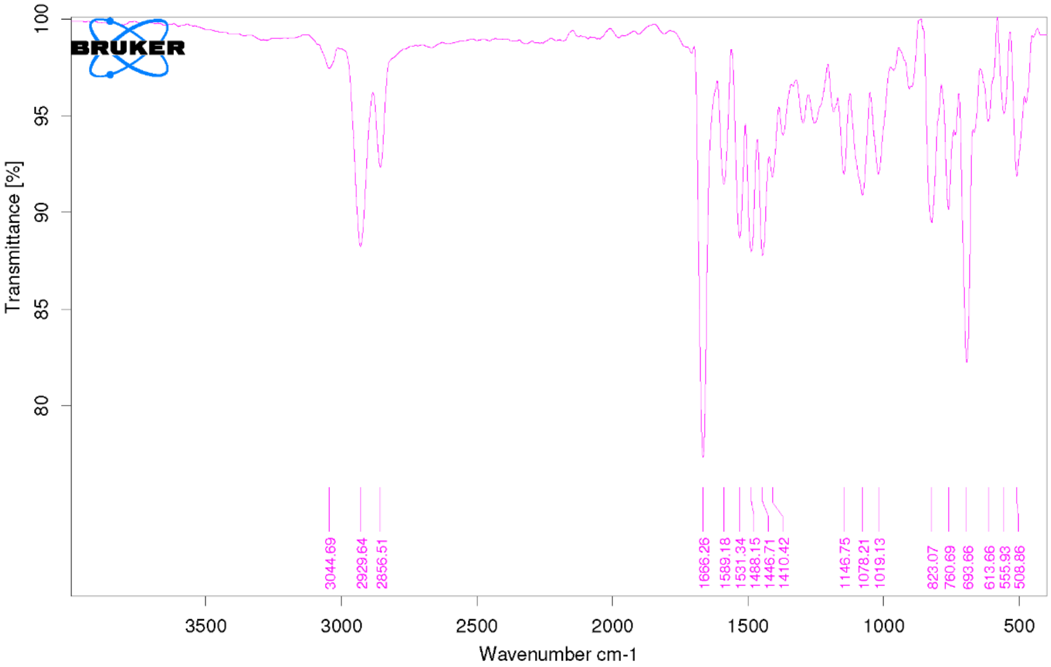

C:\Users\hvs\Documents\IR spectra\Erik Van Der Eycken\Felix\Gerardo\GM-044.0	GM-044	Instrument type and / or accessory	8/24/2018

Page 1/1

Figure S155. FT-IR (KBr) spectrum of compound **5a**.

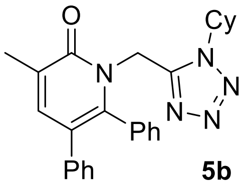

5b

1-((1-cyclohexyl-1*H*-tetrazol-5-yl)methyl)-3-methyl-5,6-diphenylpyridin-2(1*H*)-one

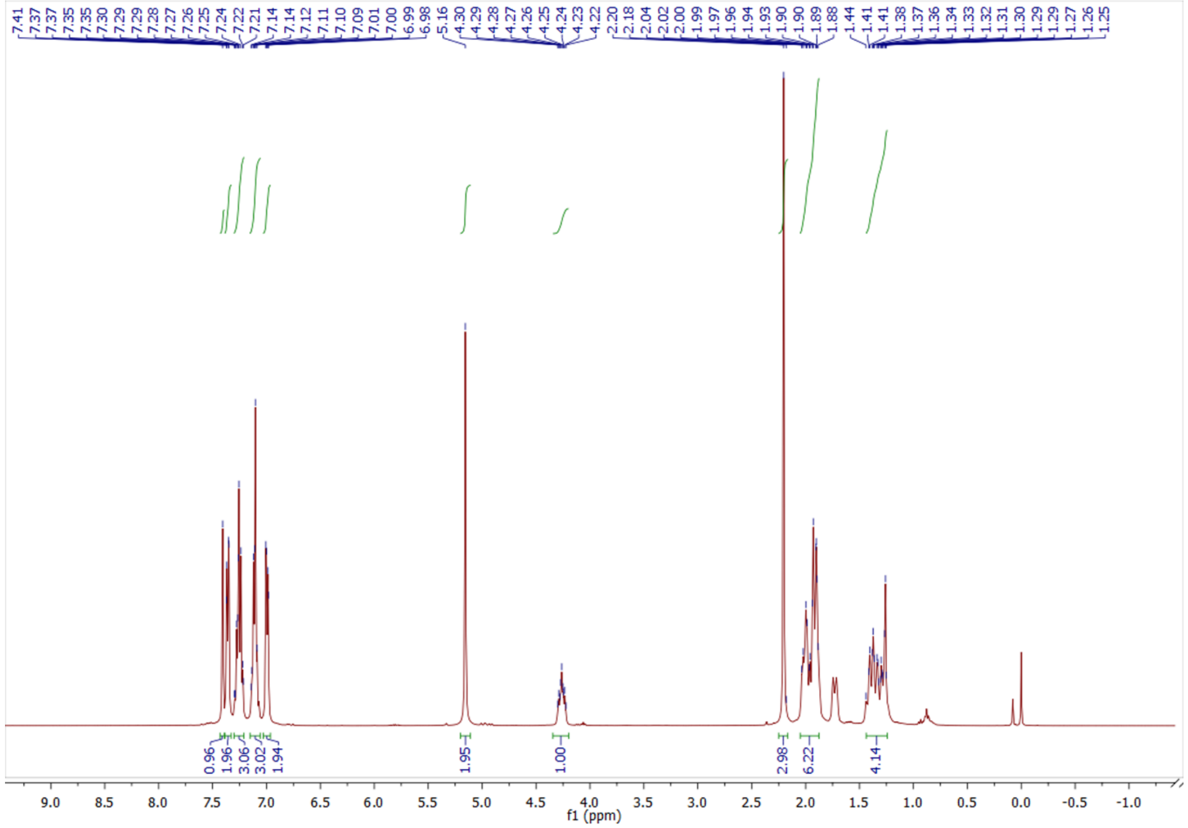

Figure S156. ^{1}H NMR (400 MHz, $CDCl_3$) spectrum of compound **5b**.

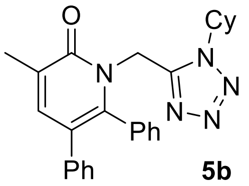

5b

1-((1-cyclohexyl-1*H*-tetrazol-5-yl)methyl)-3-methyl-5,6-diphenylpyridin-2(1*H*)-one

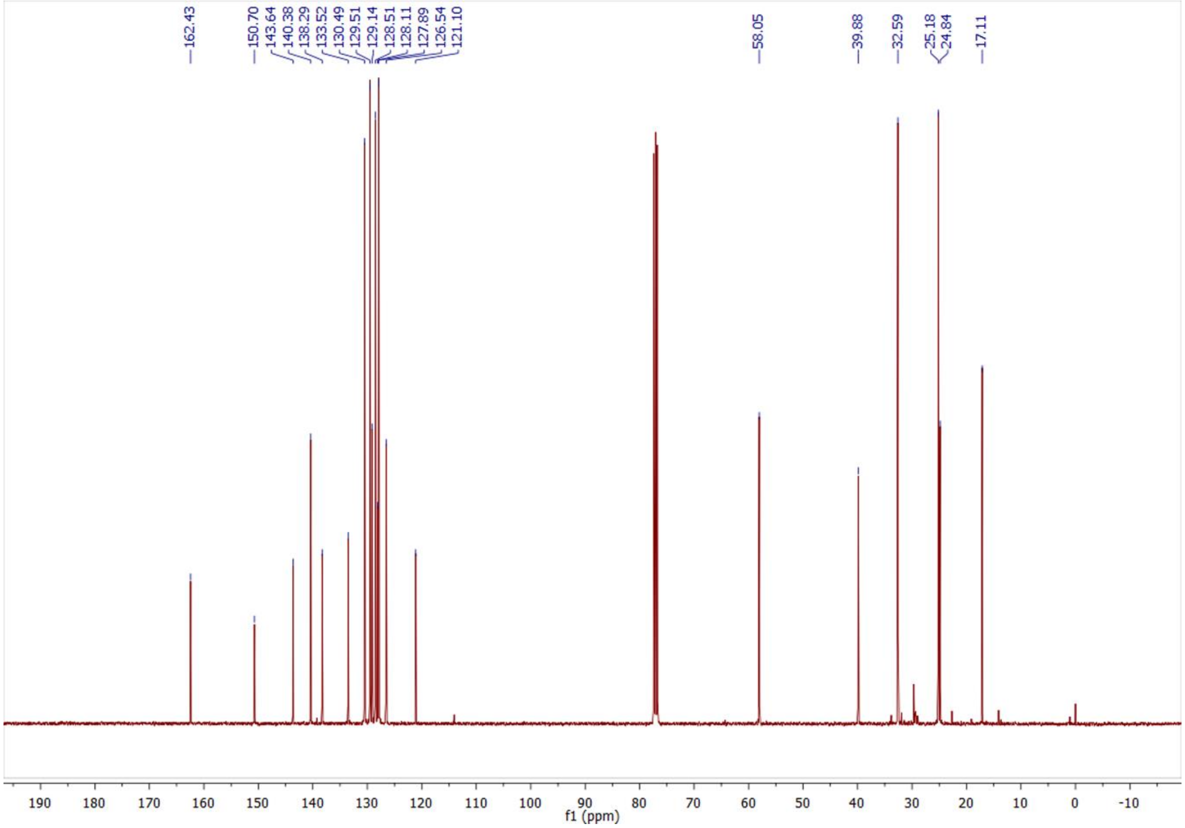

Figure S157. $^{13}C\ \{^{1}H\}$ NMR (101 MHz, $CDCl_3$) spectrum of compound **5b**.

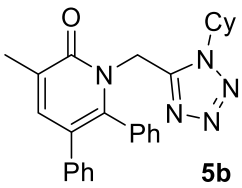

5b

1-((1-cyclohexyl-1*H*-tetrazol-5-yl)methyl)-3-methyl-5,6-diphenylpyridin-2(1*H*)-one

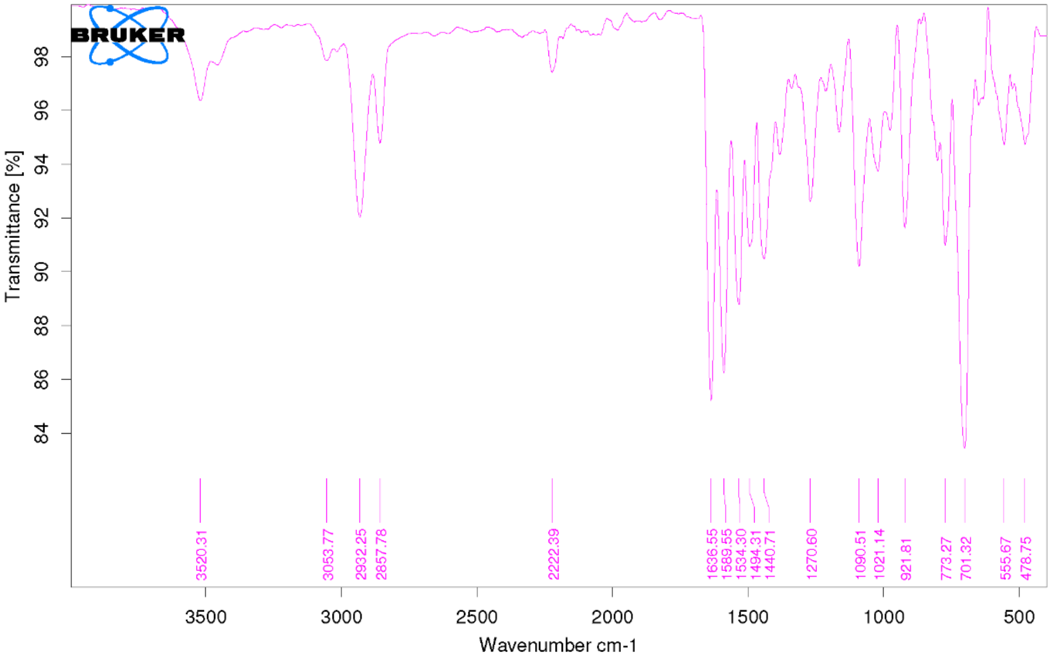

C:\Users\hvs\Documents\IR spectra\Erik Van Der Eycken\Felix\Gerardo\GM-046.0 GM-046 Instrument type and / or accessory	8/24/2018

Page 1/1

Figure S158. FT-IR (KBr) spectrum of compound **5b**.

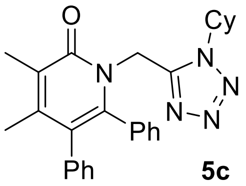

5c

1-((1-cyclohexyl-1*H*-tetrazol-5-yl)methyl)-3,4-dimethyl-5,6-diphenylpyridin-2(1*H*)-one

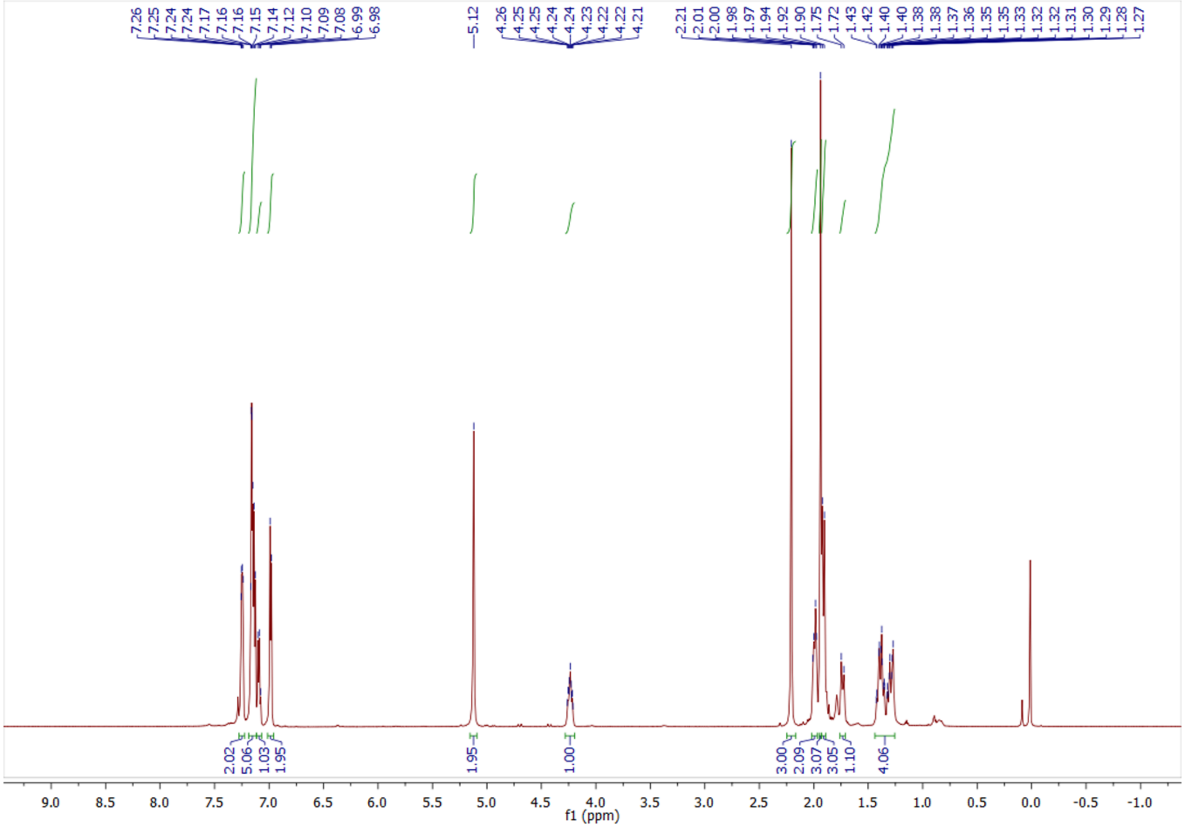

Figure S159. ^{1}H NMR (600 MHz, $CDCl_3$) spectrum of compound **5c**.

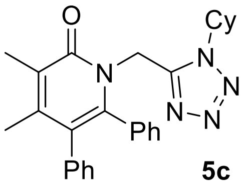

5c

1-((1-cyclohexyl-1*H*-tetrazol-5-yl)methyl)-3,4-dimethyl-5,6-diphenylpyridin-2(1*H*)-one

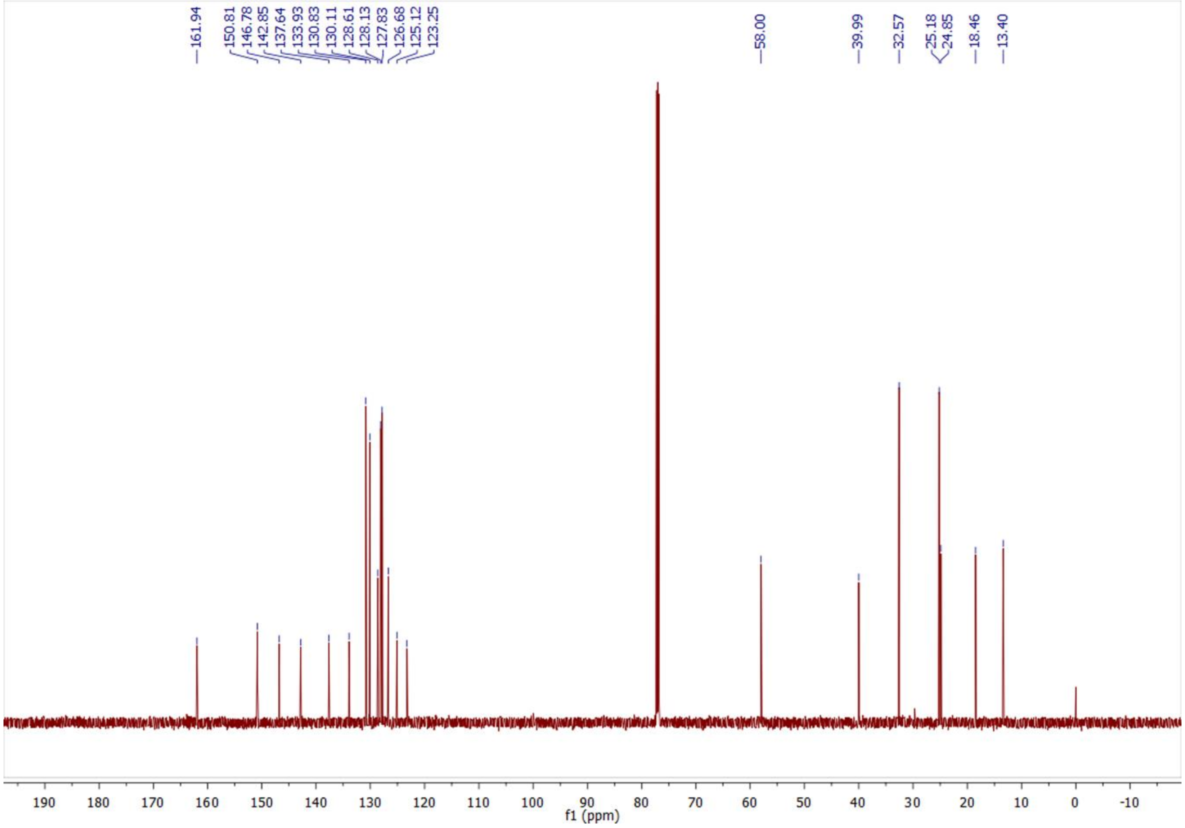

Figure S160. ^{13}C {^{1}H} NMR (151 MHz, $CDCl_3$) spectrum of compound **5c**.

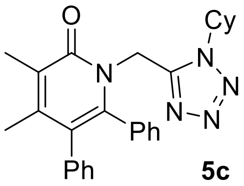

1-((1-cyclohexyl-1*H*-tetrazol-5-yl)methyl)-3,4-dimethyl-5,6-diphenylpyridin-2(1*H*)-one

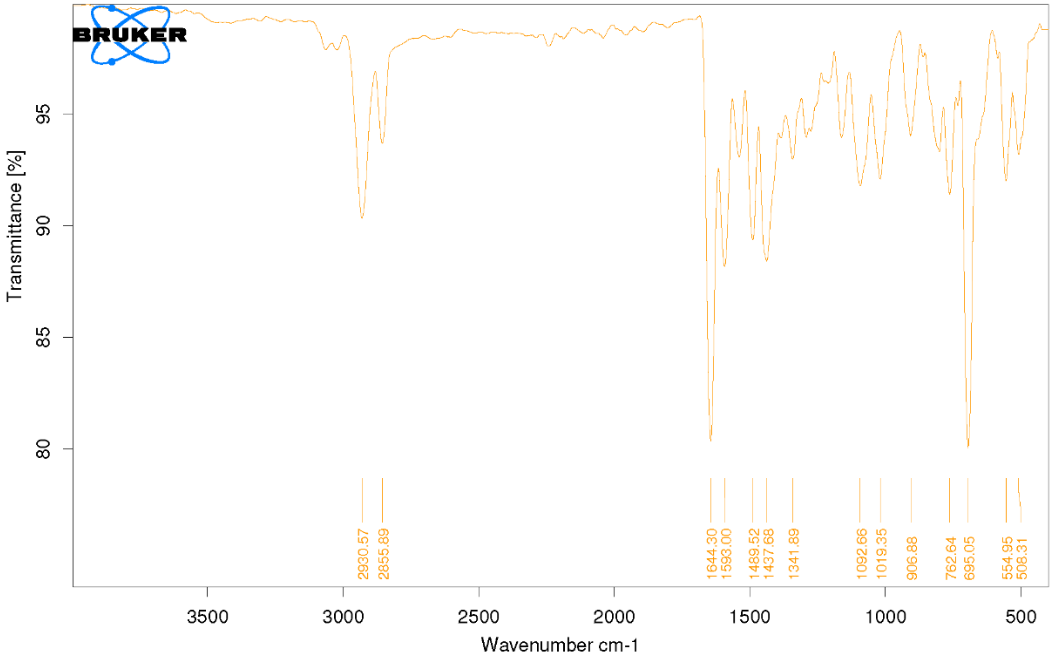

C:\Users\hvs\Documents\IR spectra\Erik Van Der Eycken\Felix\Gerardo\GM-086.0	GM-086	Instrument type and / or accessory	8/24/2018

Page 1/1

Figure S161. FT-IR (KBr) spectrum of compound **5c**.

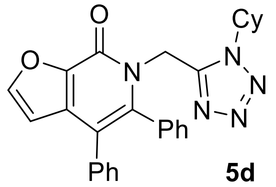

5d

6-((1-cyclohexyl-1*H*-tetrazol-5-yl)methyl)-
4,5-diphenylfuro[2,3-*c*]pyridin-7(6*H*)-one

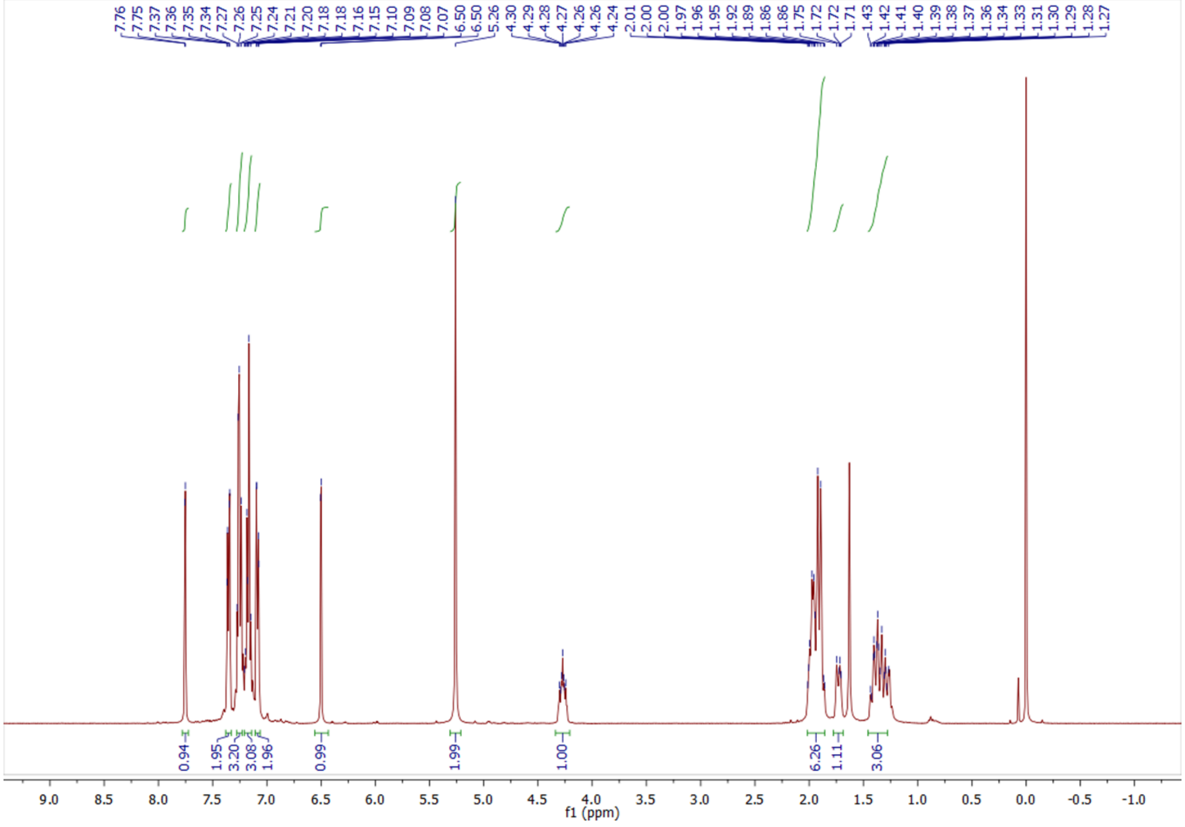

Figure S162. ^{1}H NMR (400 MHz, $CDCl_3$) spectrum of compound **5d**.

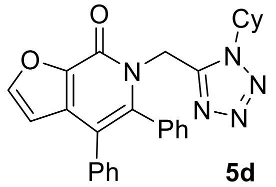

5d

6-((1-cyclohexyl-1*H*-tetrazol-5-yl)methyl)-
4,5-diphenylfuro[2,3-*c*]pyridin-7(6*H*)-one

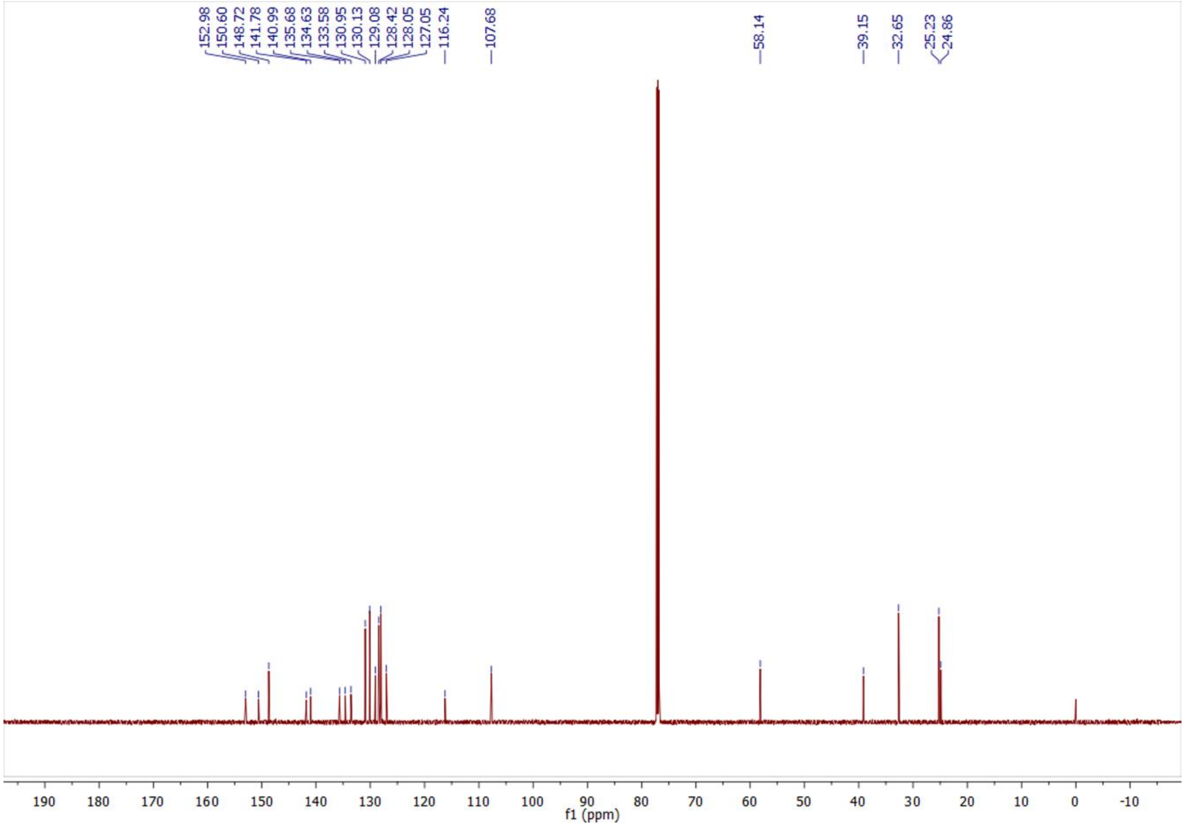

Figure S163. $^{13}C\ \{^{1}H\}$ NMR (151 MHz, $CDCl_3$) spectrum of compound **5d**.

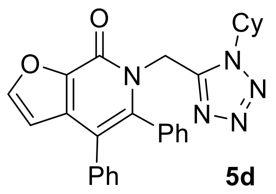

5d

6-((1-cyclohexyl-1*H*-tetrazol-5-yl)methyl)-
4,5-diphenylfuro[2,3-*c*]pyridin-7(6*H*)-one

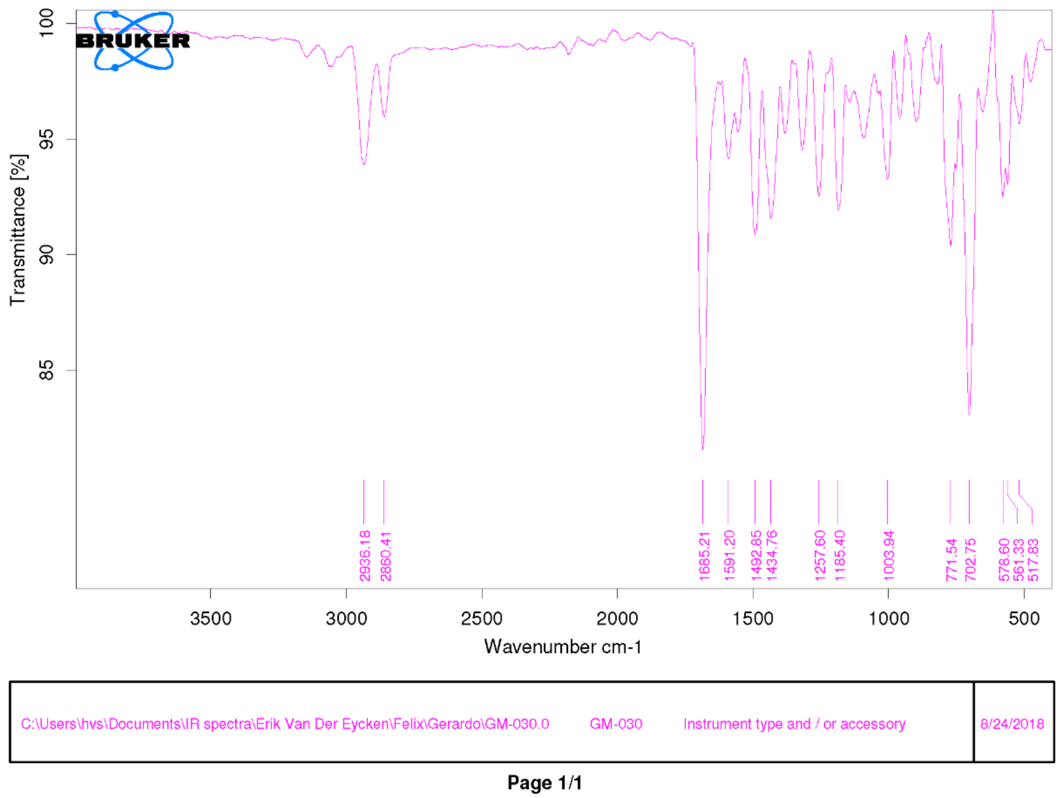

Figure S164. FT-IR (KBr) spectrum of compound **5d**.

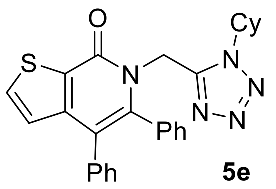

6-((1-cyclohexyl-1*H*-tetrazol-5-yl)methyl)-
4,5-diphenylthieno[2,3-*c*]pyridin-7(6*H*)-one

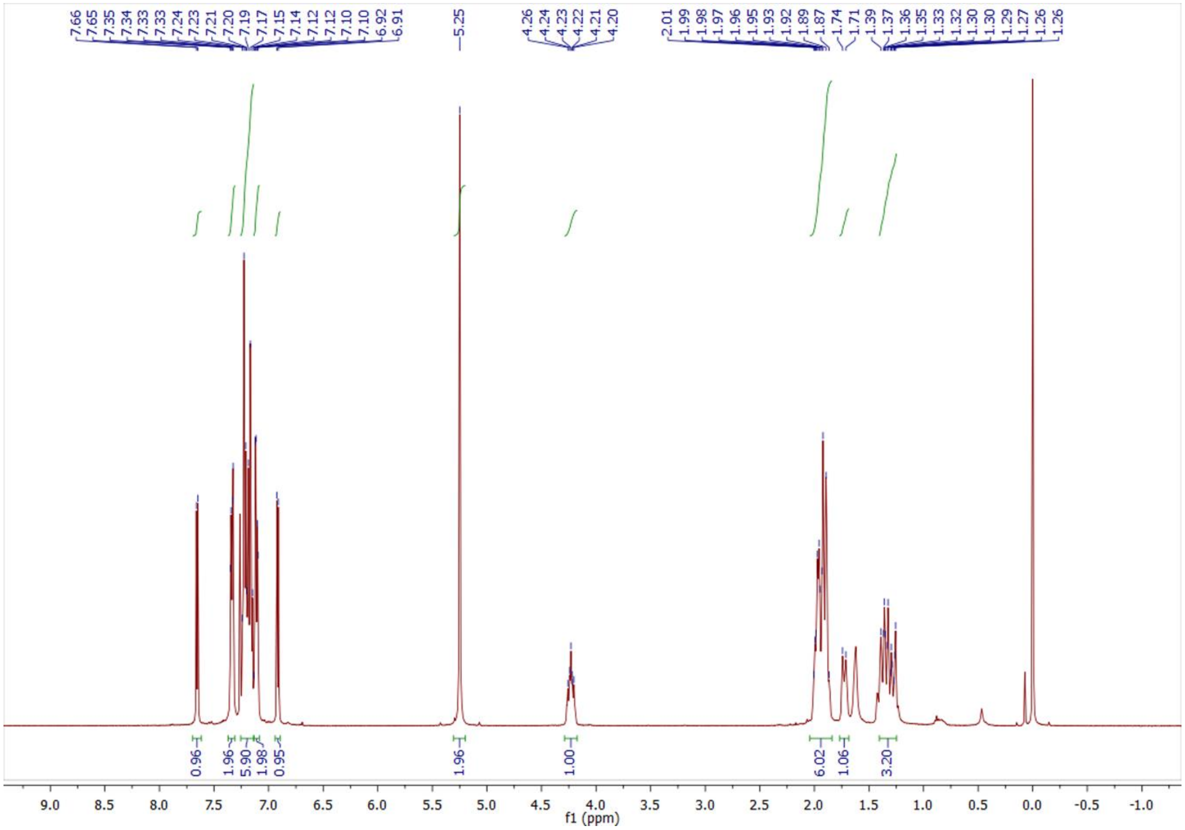

Figure S165. ^{1}H NMR (400 MHz, $CDCl_3$) spectrum of compound **5e**.

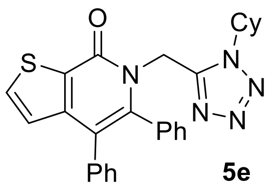

5e

6-((1-cyclohexyl-1*H*-tetrazol-5-yl)methyl)-
4,5-diphenylthieno[2,3-*c*]pyridin-7(6*H*)-one

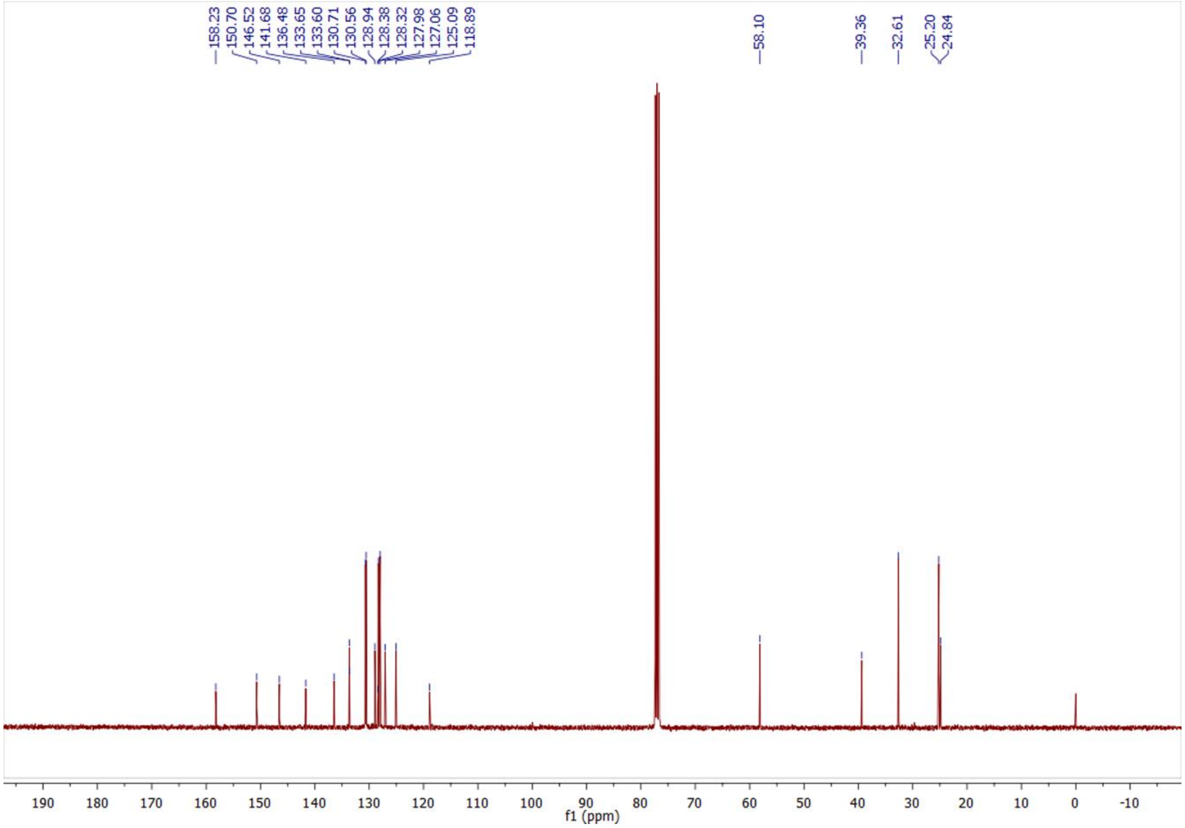

Figure S166. $^{13}C\ \{^{1}H\}$ NMR (101 MHz, $CDCl_3$) spectrum of compound **5e**.

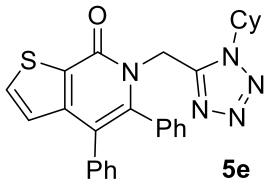

5e

6-((1-cyclohexyl-1*H*-tetrazol-5-yl)methyl)-
4,5-diphenylthieno[2,3-*c*]pyridin-7(6*H*)-one

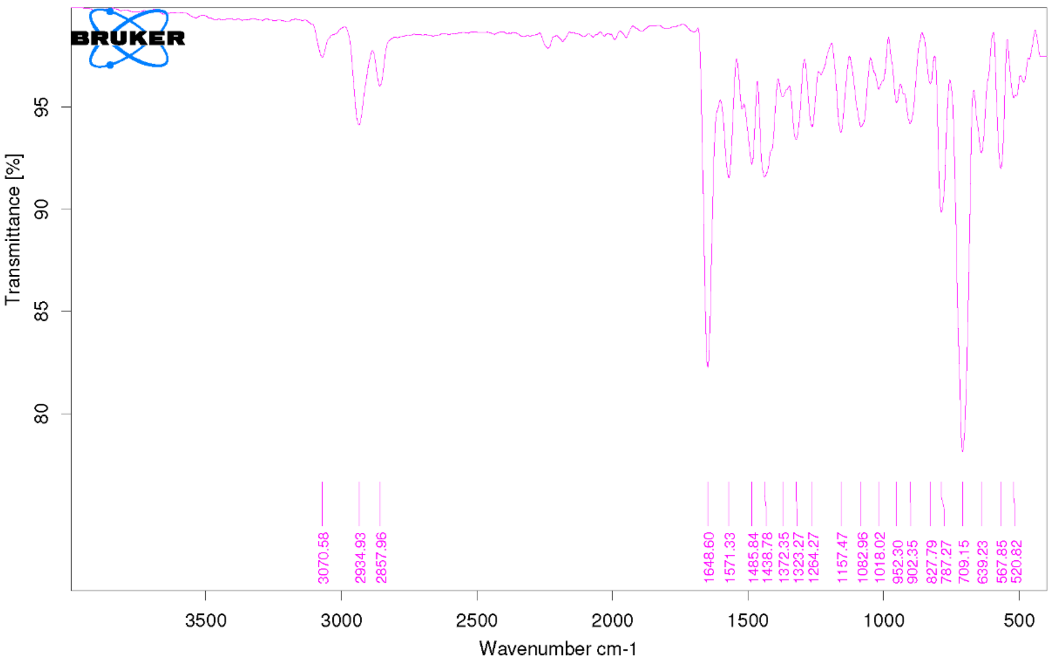

C:\Users\hvs\Documents\IR spectra\Erik Van Der Eycken\Felix\Gerardo\GM-050.0	GM-050	Instrument type and / or accessory	10/12/2018

Page 1/1

Figure S167. FT-IR (KBr) spectrum of compound **5e**.

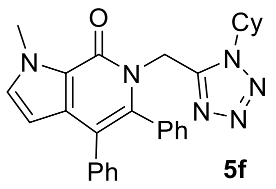

5f

6-((1-cyclohexyl-1*H*-tetrazol-5-yl)methyl)-1-methyl-4,5-diphenyl-1,6-dihydro-7*H*-pyrrolo[2,3-*c*]pyridin-7-one

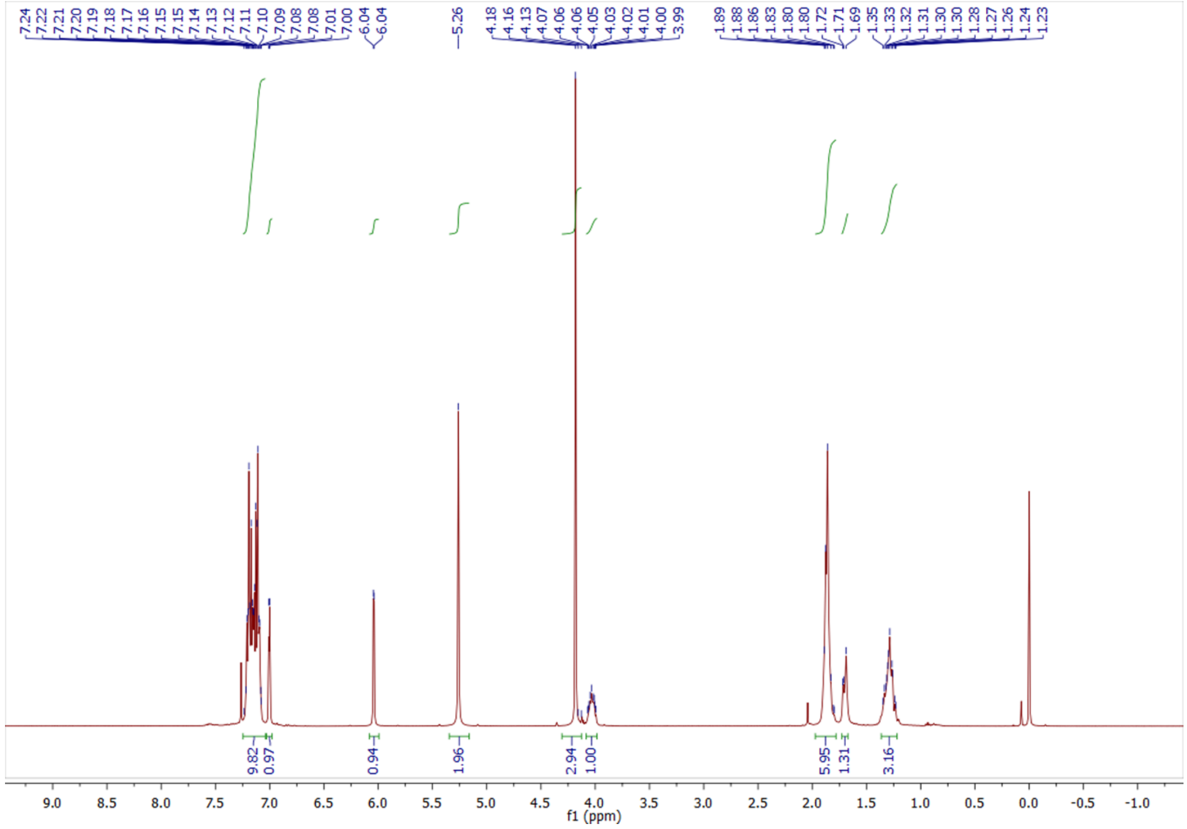

Figure S168. ^{1}H NMR (400 MHz, $CDCl_3$) spectrum of compound **5f**.

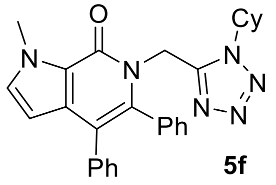

5f

6-((1-cyclohexyl-1*H*-tetrazol-5-yl)methyl)-1-methyl-4,5-diphenyl-1,6-dihydro-7*H*-pyrrolo[2,3-*c*]pyridin-7-one

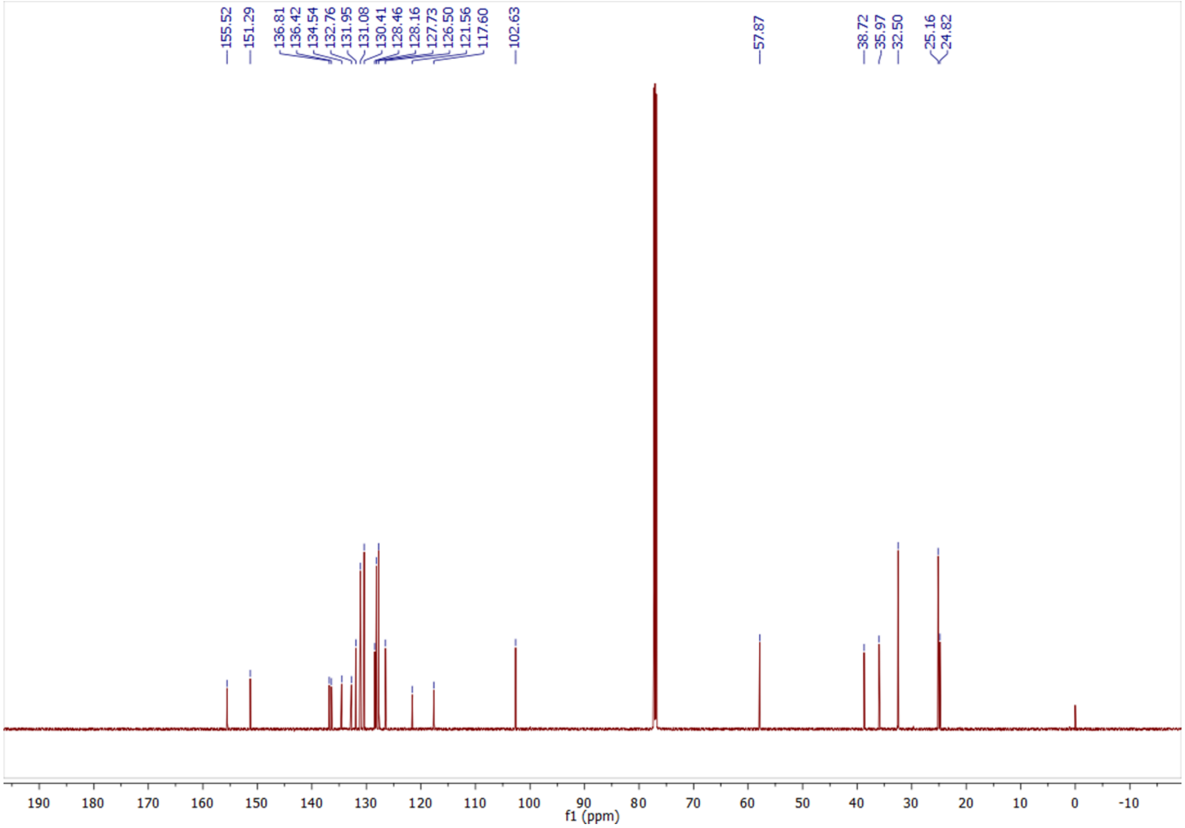

Figure S169. ^{13}C {^{1}H} NMR (151 MHz, $CDCl_3$) spectrum of compound **5f**.

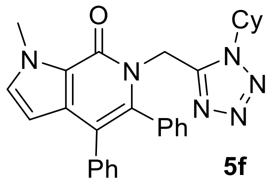

5f

6-((1-cyclohexyl-1*H*-tetrazol-5-yl)methyl)-1-methyl-4,5-diphenyl-1,6-dihydro-7*H*-pyrrolo[2,3-*c*]pyridin-7-one

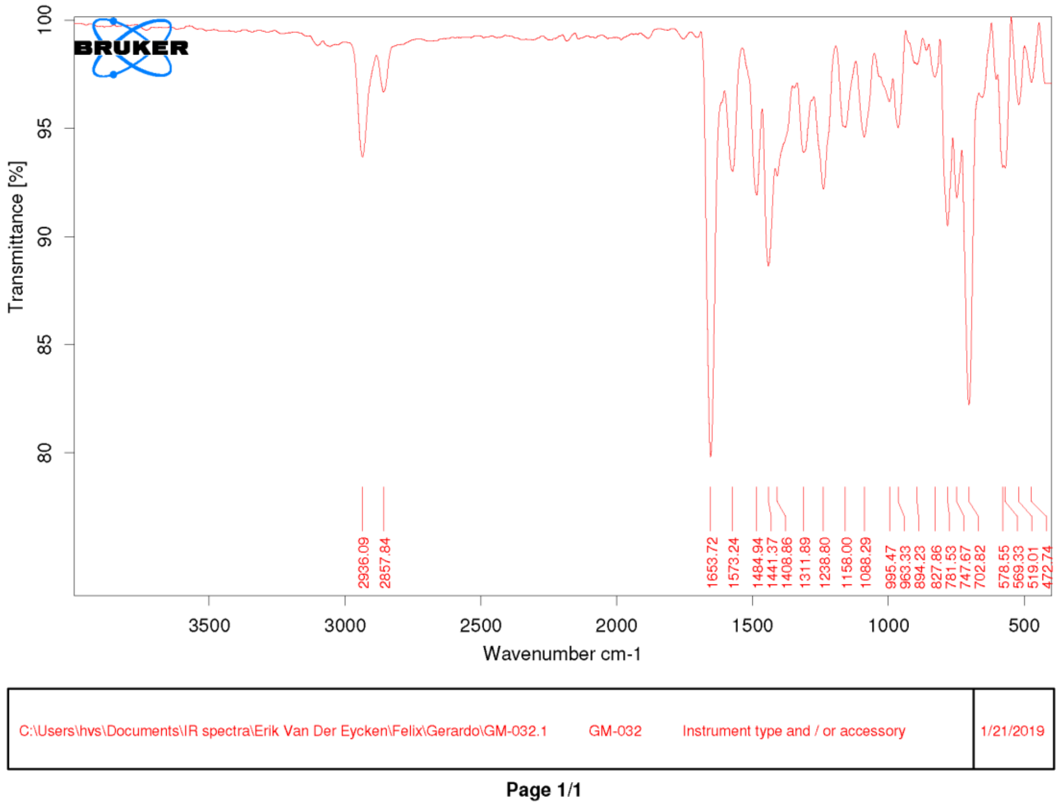

Figure S170. FT-IR (KBr) spectrum of compound **5f**.

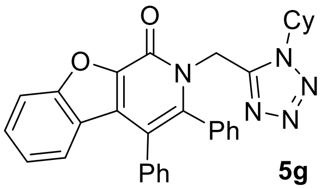

5g

2-((1-cyclohexyl-1*H*-tetrazol-5-yl)methyl)-3,4-diphenylbenzofuro[2,3-*c*]pyridin-1(2*H*)-one

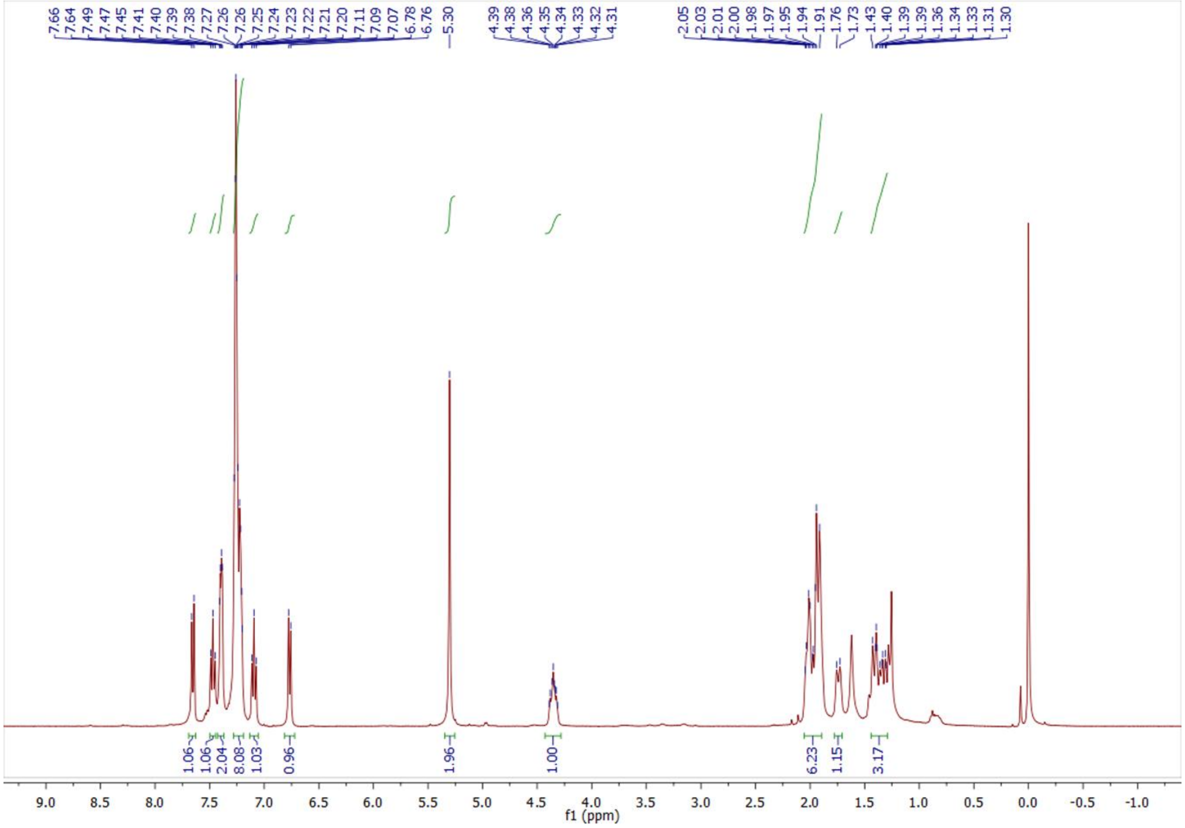

Figure S171. ^{1}H NMR (400 MHz, $CDCl_3$) spectrum of compound **5g**.

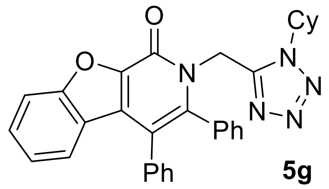

2-((1-cyclohexyl-1*H*-tetrazol-5-yl)methyl)-
3,4-diphenylbenzofuro[2,3-*c*]pyridin-1(2*H*)-one

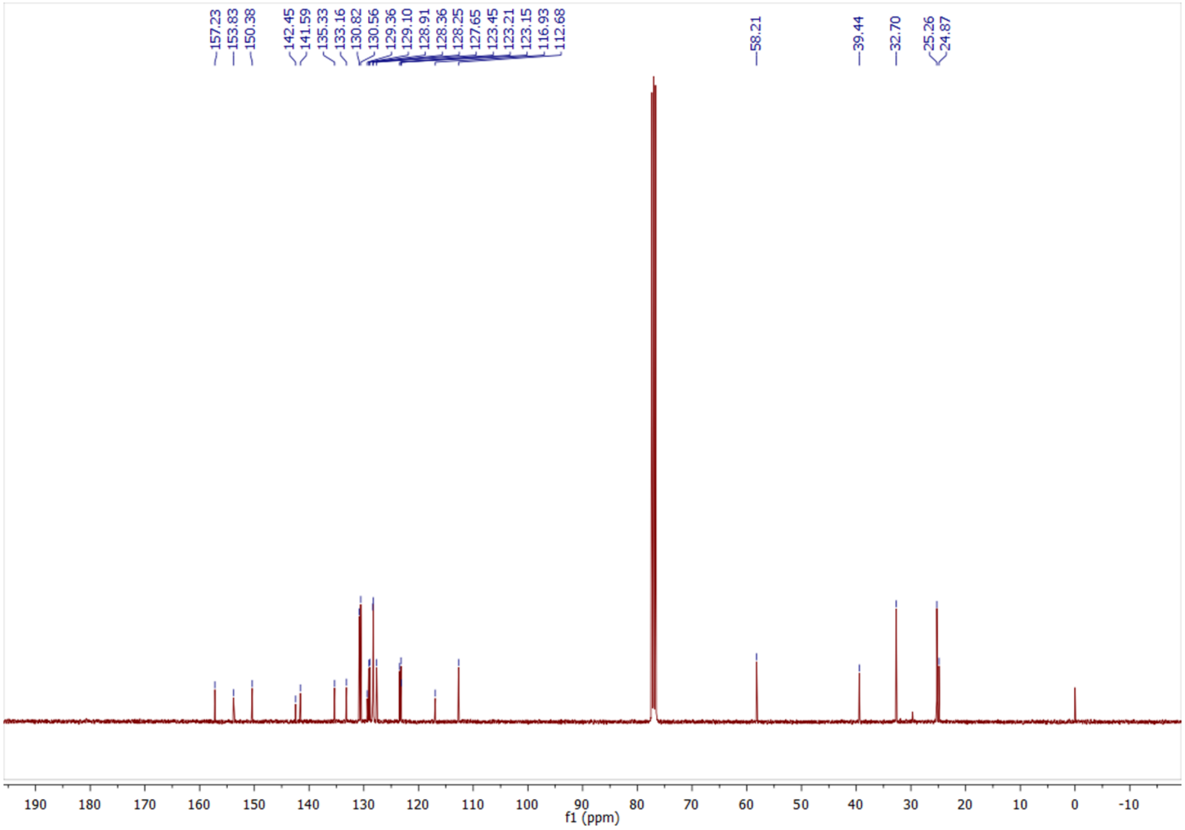

Figure S172. $^{13}C\{^{1}H\}$ NMR (101 MHz, $CDCl_3$) spectrum of compound **5g**.

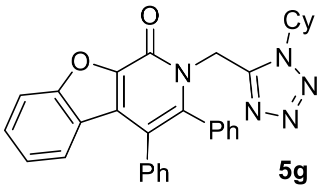

5g

2-((1-cyclohexyl-1*H*-tetrazol-5-yl)methyl)-
3,4-diphenylbenzofuro[2,3-*c*]pyridin-1(2*H*)-one

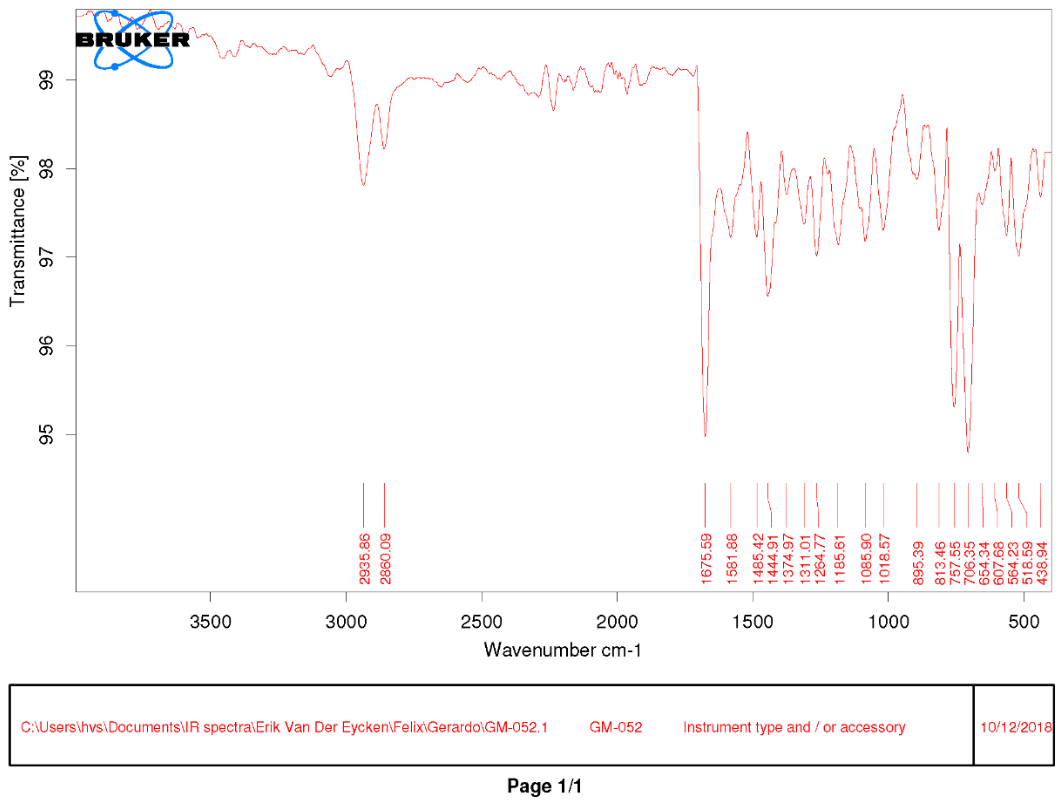

Figure S173. FT-IR (KBr) spectrum of compound **5g**.

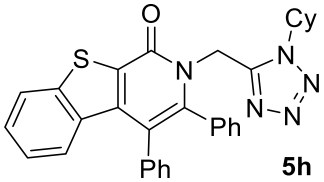

5h

2-((1-cyclohexyl-1*H*-tetrazol-5-yl)methyl)-
3,4-diphenylbenzo[4,5]thieno[2,3-*c*]pyridin-1(2*H*)-one

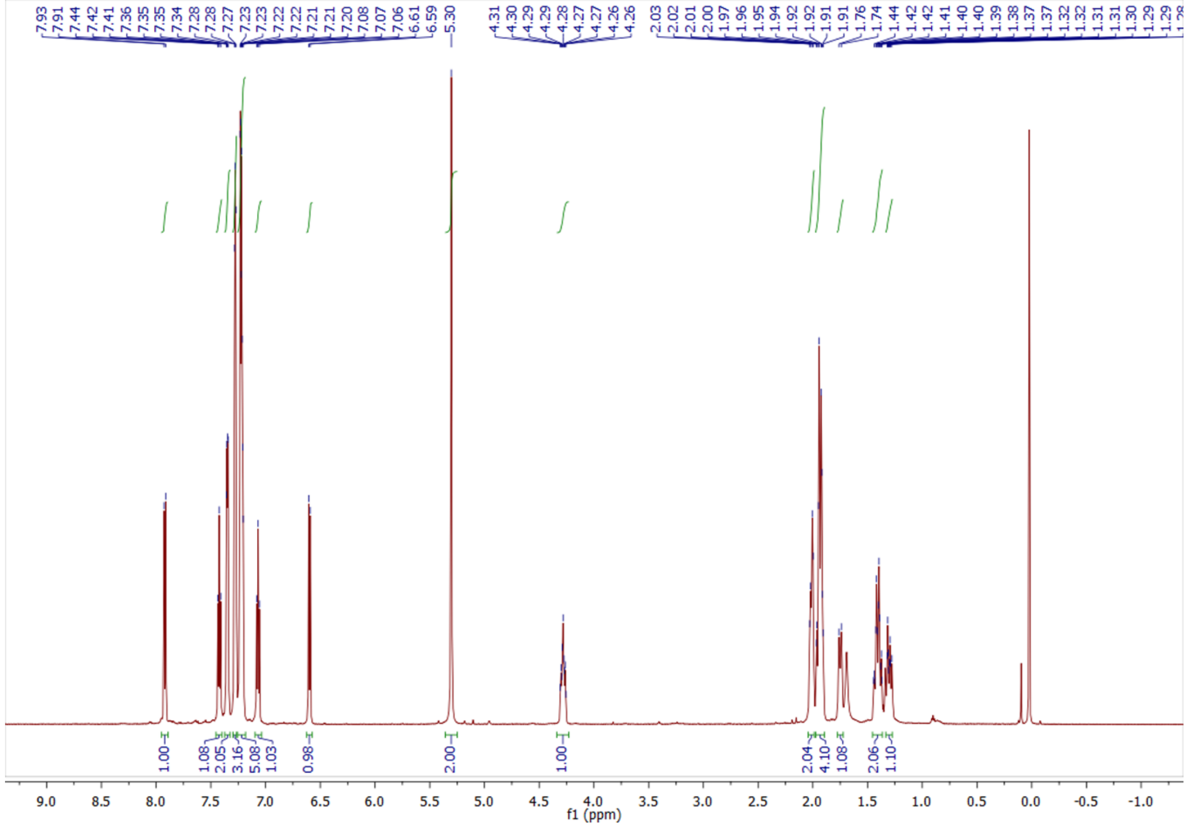

Figure S174. ^{1}H NMR (600 MHz, $CDCl_3$) spectrum of compound **5h**.

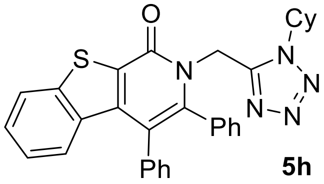

5h

2-((1-cyclohexyl-1*H*-tetrazol-5-yl)methyl)-
3,4-diphenylbenzo[4,5]thieno[2,3-*c*]pyridin-1(2*H*)-one

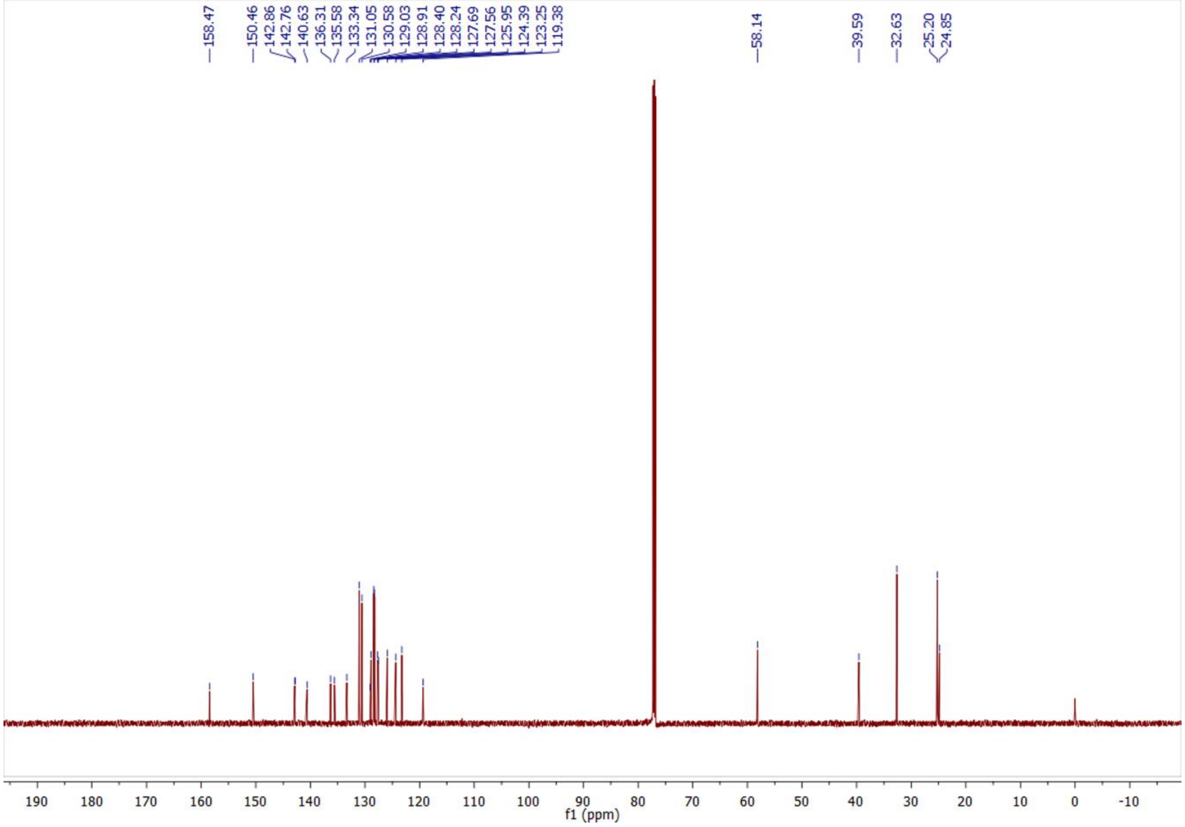

Figure S175. ^{13}C {^{1}H} NMR (151 MHz, $CDCl_3$) spectrum of compound **5h**.

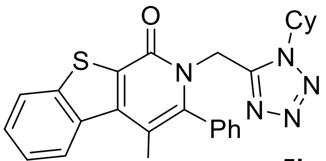

5h

2-((1-cyclohexyl-1*H*-tetrazol-5-yl)methyl)-
3,4-diphenylbenzo[4,5]thieno[2,3-*c*]pyridin-1(2*H*)-one

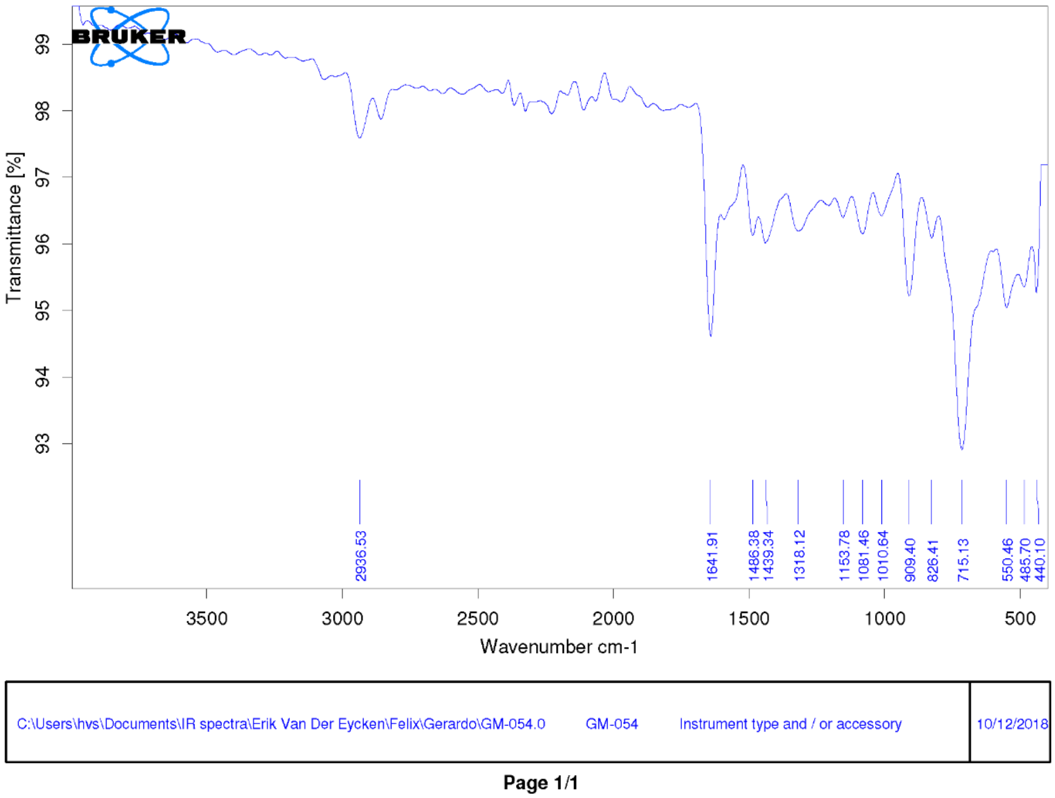

Figure S176. FT-IR (KBr) spectrum of compound **5h**.

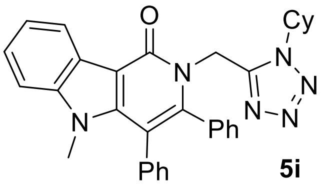

5i

2-((1-cyclohexyl-1*H*-tetrazol-5-yl)methyl)-5-methyl-3,4-diphenyl-2,5-dihydro-1*H*-pyrido[4,3-*b*]indol-1-one

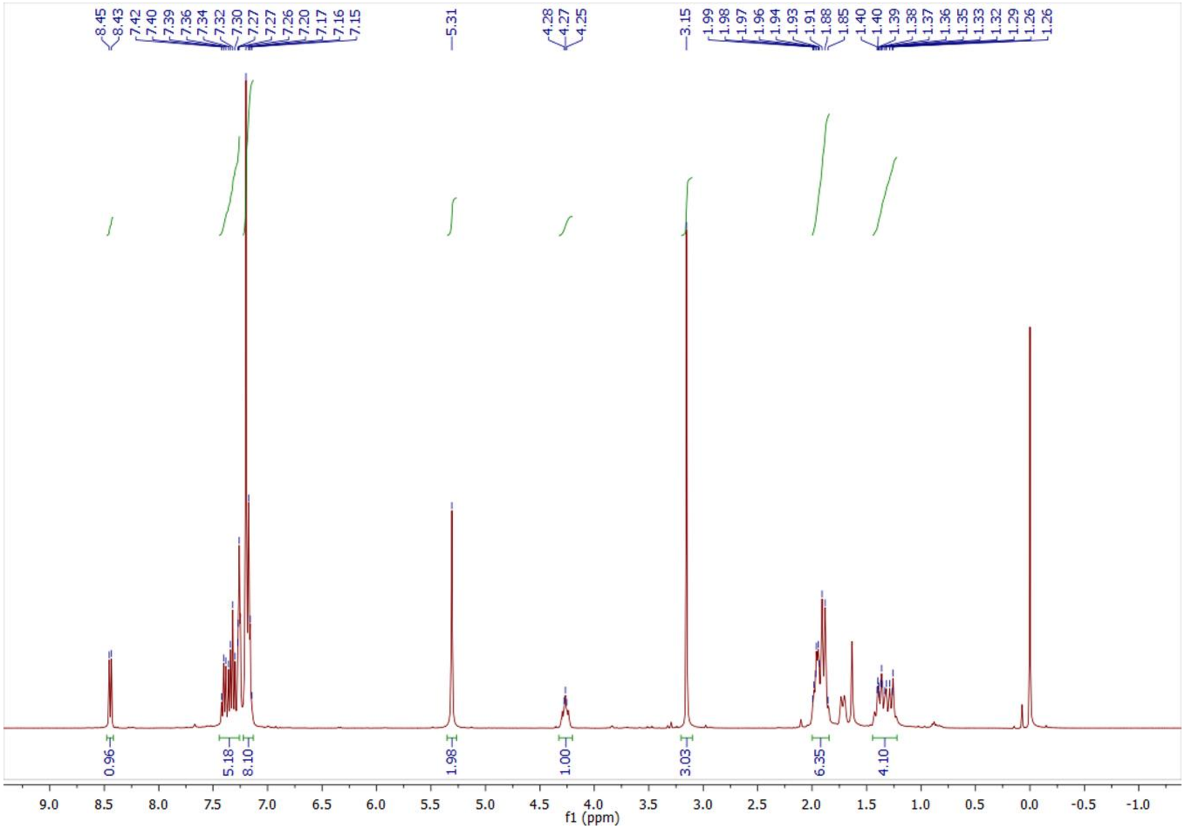

Figure S177. ^{1}H NMR (400 MHz, $CDCl_3$) spectrum of compound **5i**.

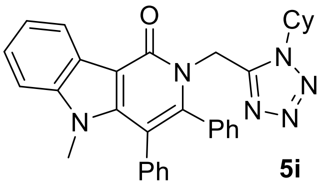

5i

2-((1-cyclohexyl-1*H*-tetrazol-5-yl)methyl)-5-methyl-3,4-diphenyl-2,5-dihydro-1*H*-pyrido[4,3-*b*]indol-1-one

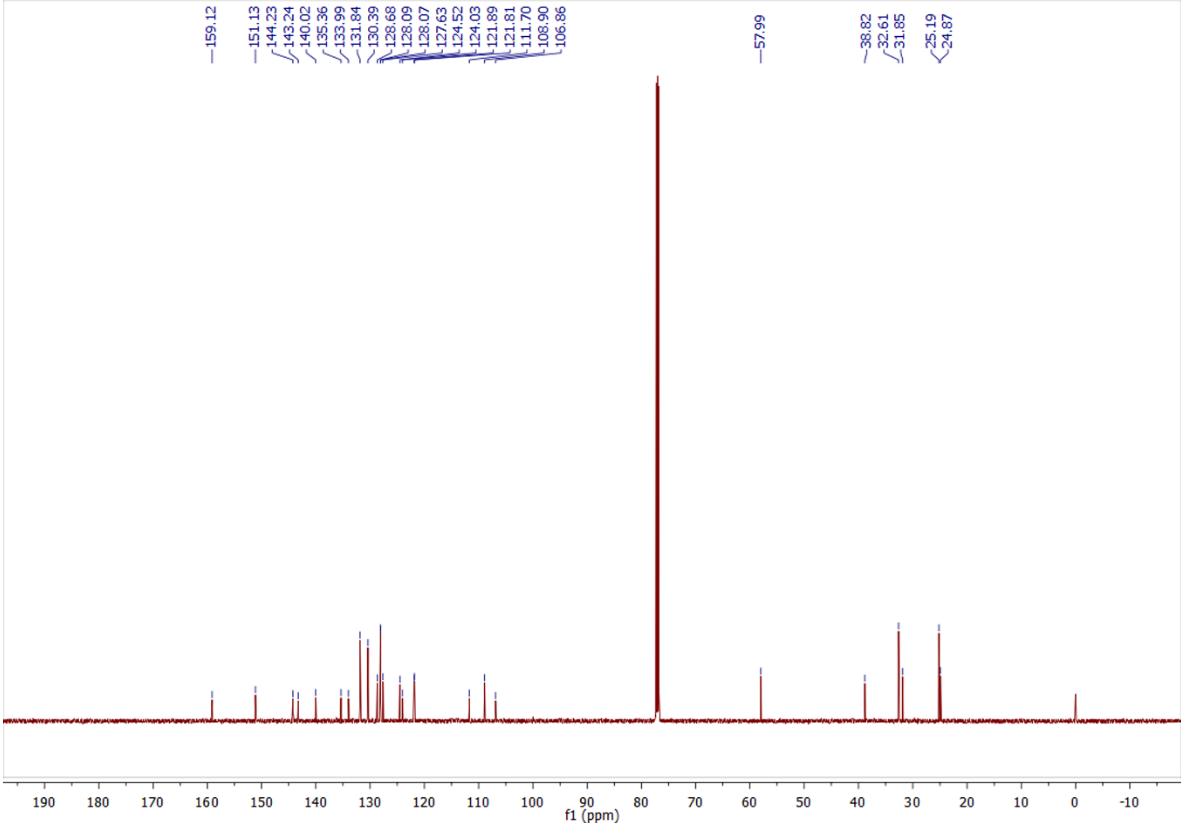

Figure S178. ^{13}C {^{1}H} NMR (151 MHz, $CDCl_3$) spectrum of compound **5i**.

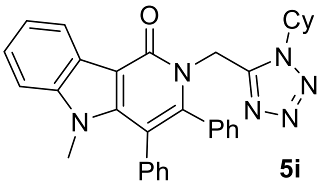

5i

2-((1-cyclohexyl-1*H*-tetrazol-5-yl)methyl)-5-methyl-3,4-diphenyl-2,5-dihydro-1*H*-pyrido[4,3-*b*]indol-1-one

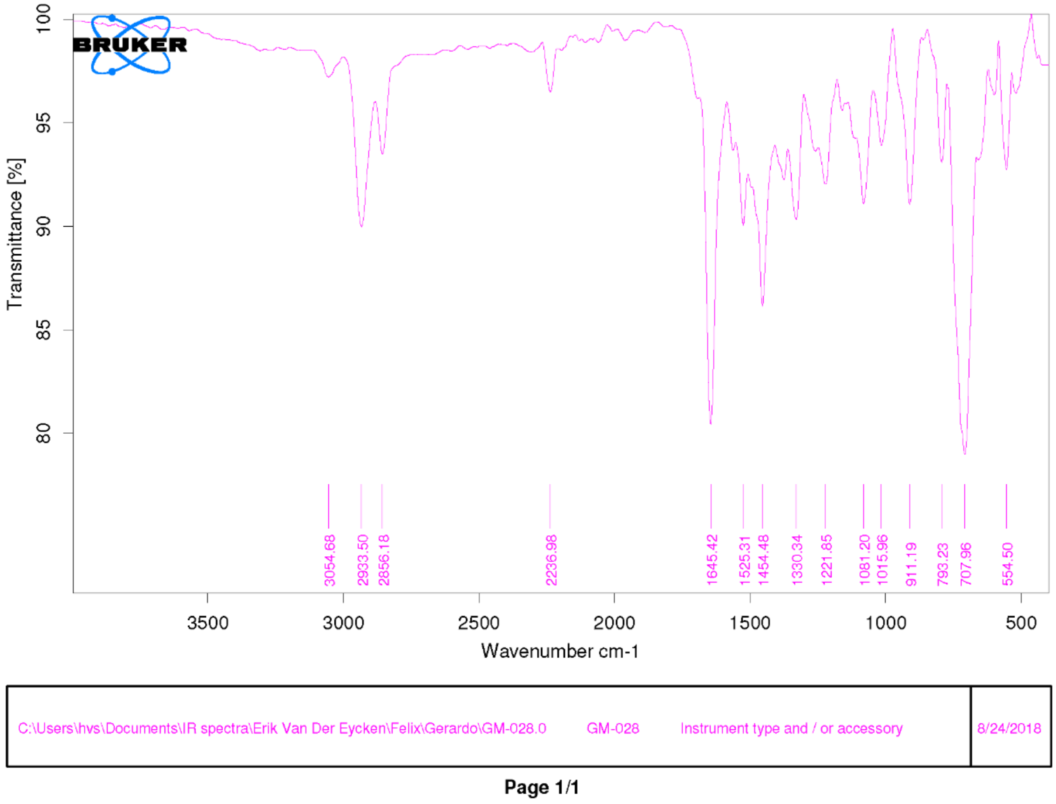

Figure S179. FT-IR (KBr) spectrum of compound **5i**.

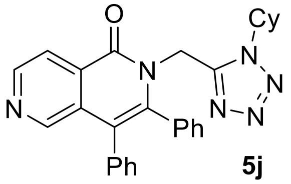

5j

2-((1-cyclohexyl-1*H*-tetrazol-5-yl)methyl)-3,4-diphenyl-2,6-naphthyridin-1(2*H*)-one

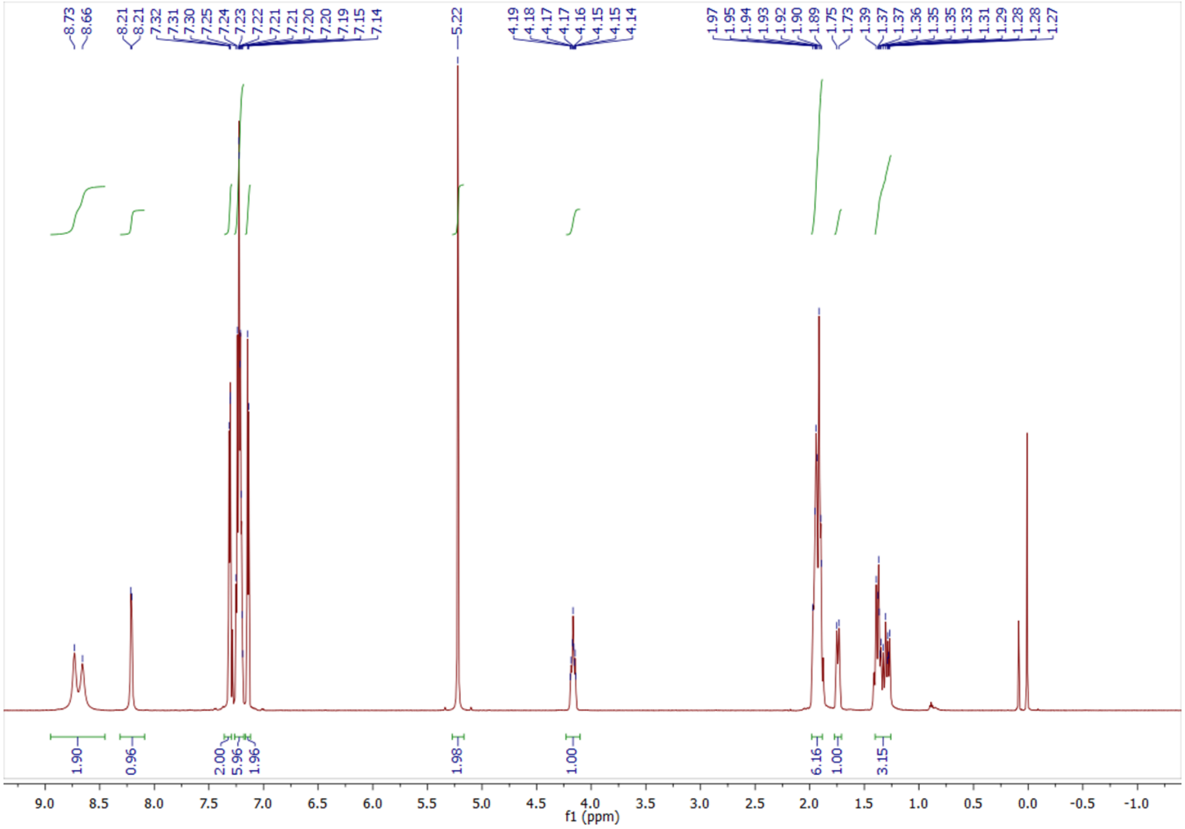

Figure S180. ^{1}H NMR (600 MHz, $CDCl_3$) spectrum of compound **5j**.

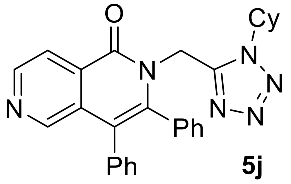

5j

2-((1-cyclohexyl-1*H*-tetrazol-5-yl)methyl)-3,4-diphenyl-2,6-naphthyridin-1(2*H*)-one

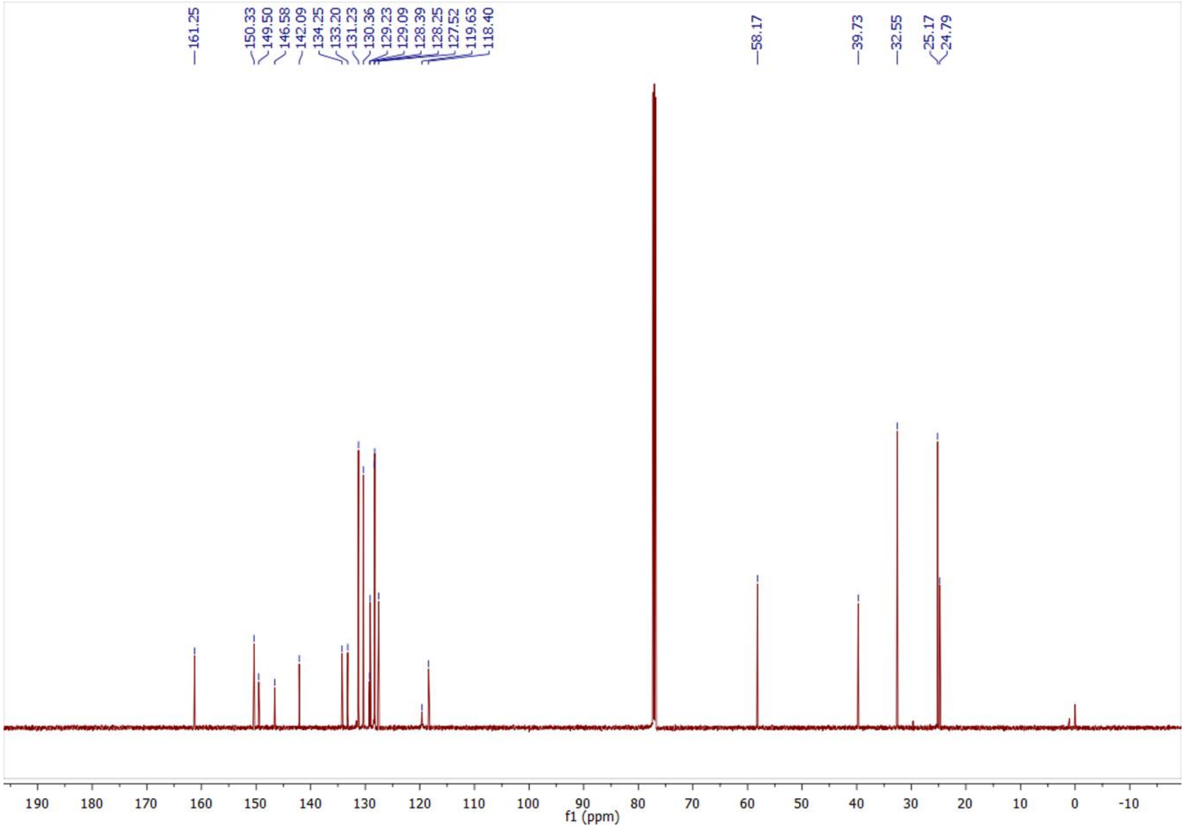

Figure S181. ^{13}C {^{1}H} NMR (151 MHz, $CDCl_3$) spectrum of compound **5j**.

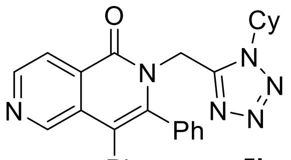

5j

2-((1-cyclohexyl-1*H*-tetrazol-5-yl)methyl)-3,4-diphenyl-2,6-naphthyridin-1(2*H*)-one

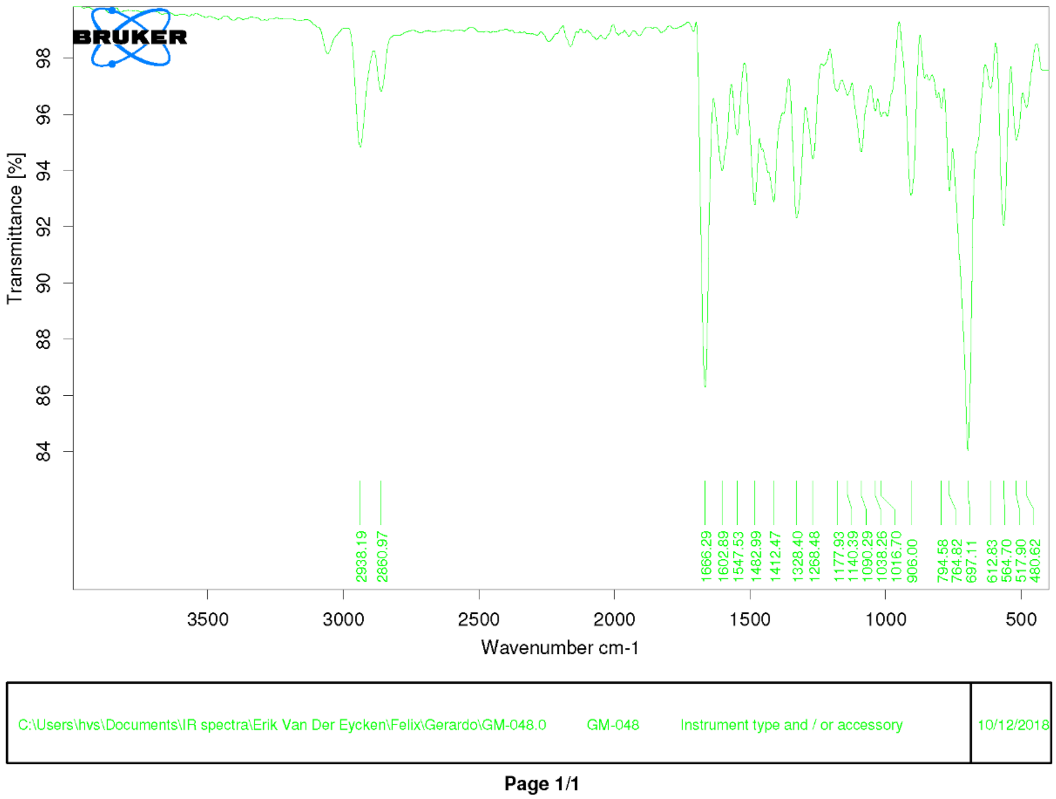

Figure S182. FT-IR (KBr) spectrum of compound **5j**.

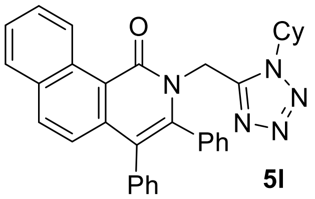

5l

2-((1-cyclohexyl-1*H*-tetrazol-5-yl)methyl)-
3,4-diphenylbenzo[*h*]isoquinolin-1(2*H*)-one

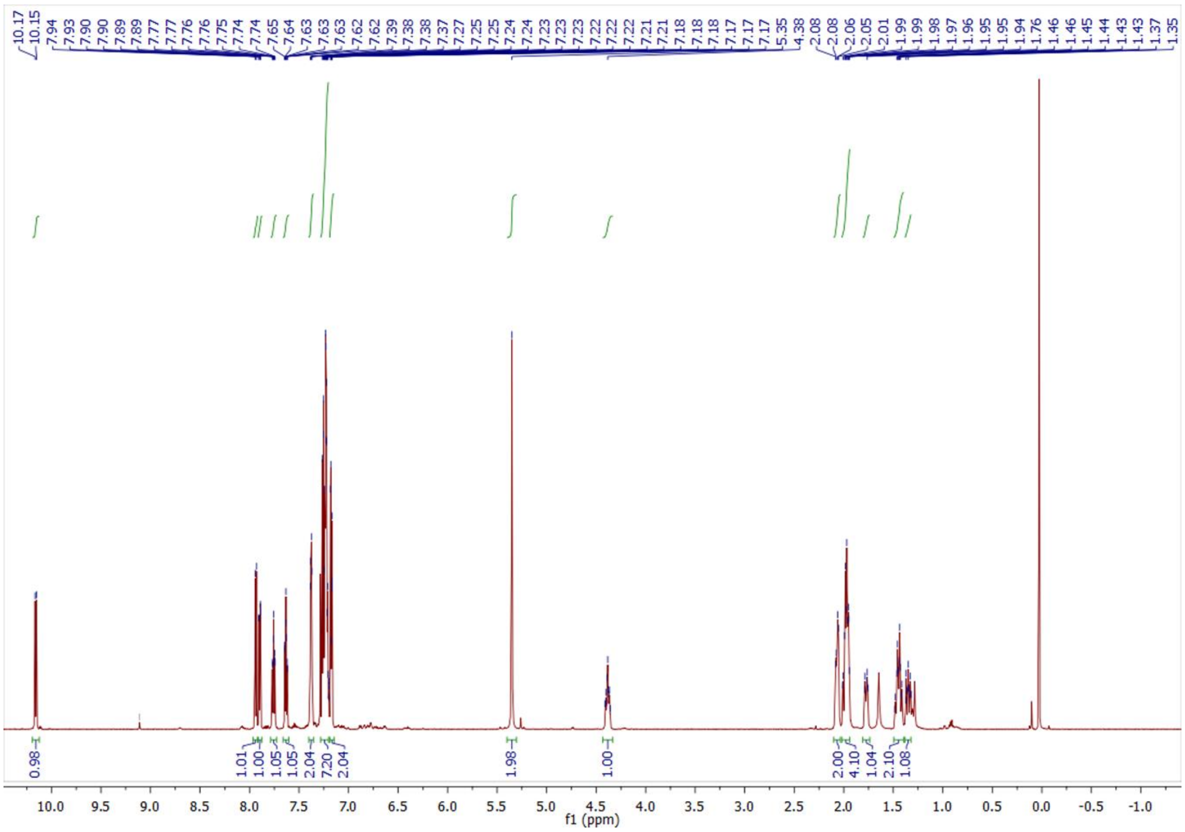

Figure S183. ^{1}H NMR (600 MHz, $CDCl_3$) spectrum of compound **5l**.

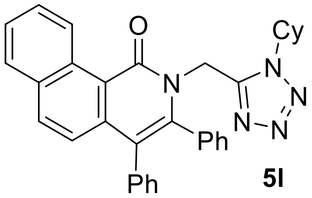

5l

2-((1-cyclohexyl-1*H*-tetrazol-5-yl)methyl)-
3,4-diphenylbenzo[*h*]isoquinolin-1(2*H*)-one

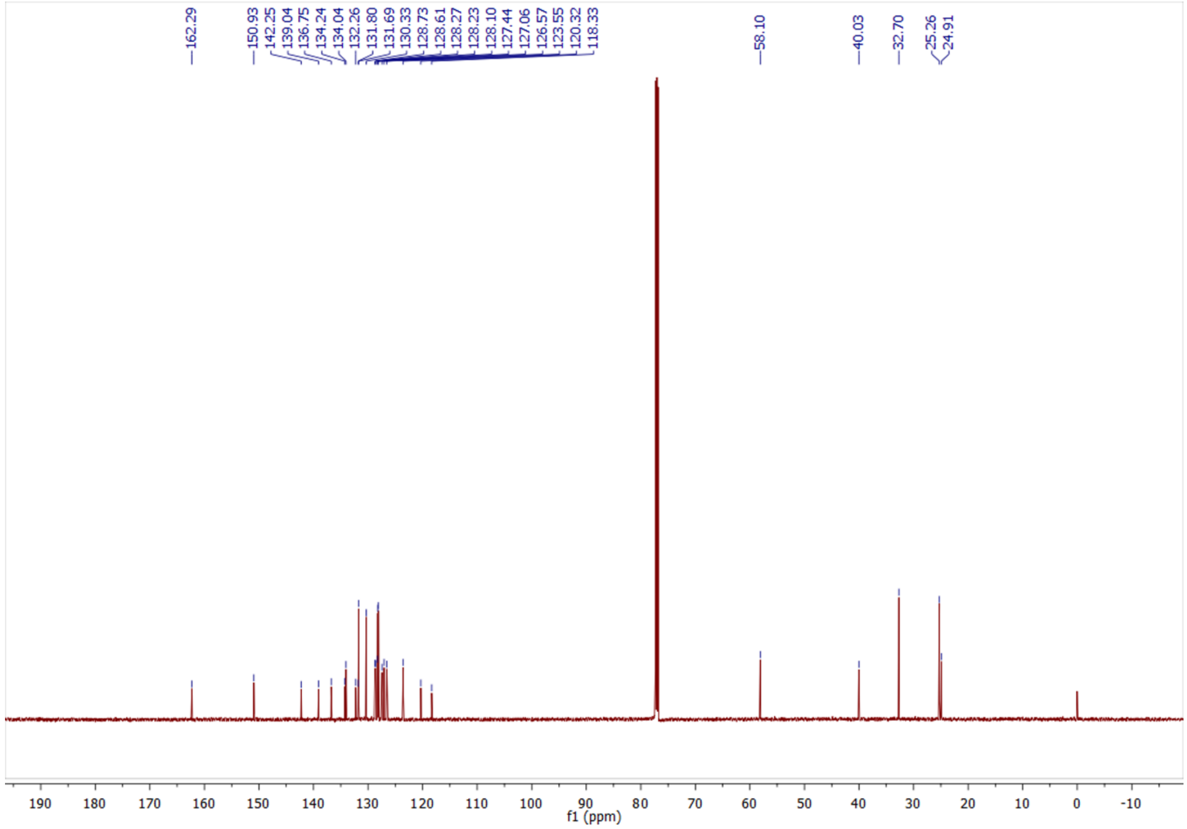

Figure S184. $^{13}C\ \{^{1}H\}$ NMR (151 MHz, $CDCl_3$) spectrum of compound **5l**.

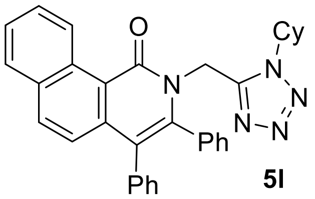

5l

2-((1-cyclohexyl-1*H*-tetrazol-5-yl)methyl)-3,4-diphenylbenzo[*h*]isoquinolin-1(2*H*)-one

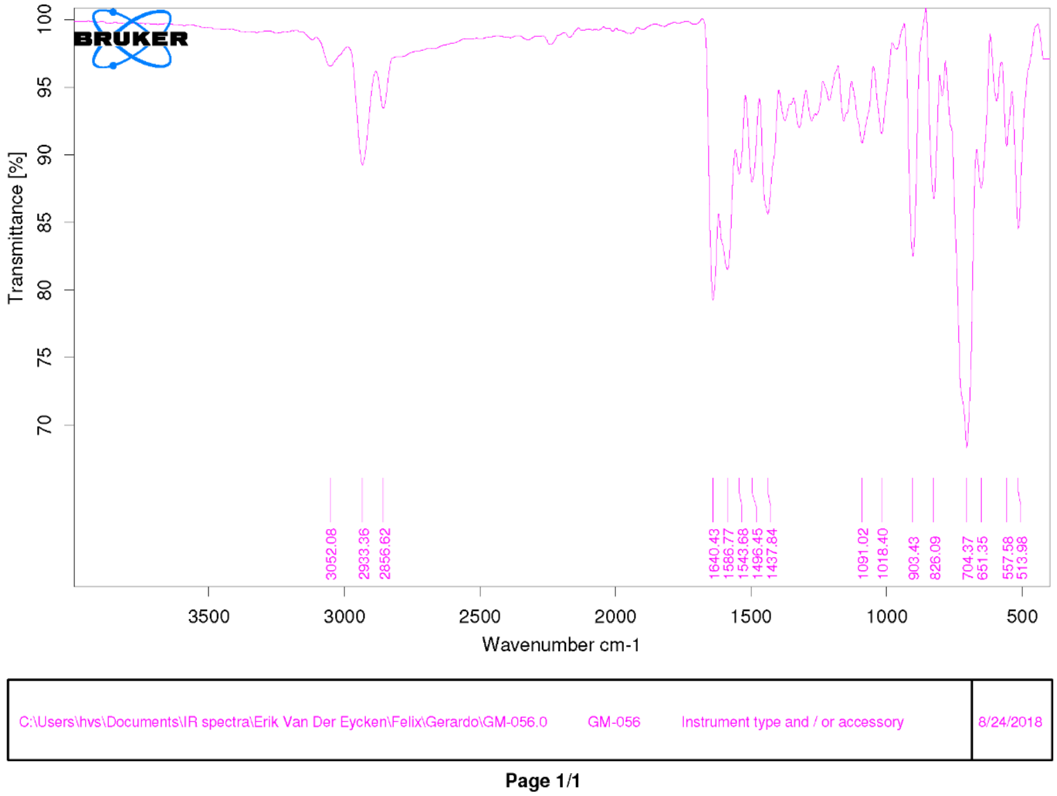

Figure S185. FT-IR (KBr) spectrum of compound **5l**.
